# Dedifferentiated fat cells administration ameliorates abnormal expressions of fatty acids metabolism-related protein expressions and intestinal tissue damage in experimental necrotizing enterocolitis

**DOI:** 10.1038/s41598-023-34156-1

**Published:** 2023-05-22

**Authors:** Haruka Mimatsu, Atsuto Onoda, Tomohiko Kazama, Koji Nishijima, Yoshie Shimoyama, Shoji Go, Kazuto Ueda, Yoshiyuki Takahashi, Taro Matsumoto, Masahiro Hayakawa, Yoshiaki Sato

**Affiliations:** 1 Division of Neonatology, Center for Maternal-Neonatal Care, Nagoya University Hospital, 65 Tsurumai-Cho Showa-Ku, Nagoya, 466-8550 Japan; 2 Department of Pediatrics, Nagoya University Graduate School of Medicine, Nagoya, Japan; 3 Faculty of Pharmaceutical Sciences, Sanyo-Onoda City University, Sanyo-Onoda, Yamaguchi, Japan; 4 Department of Functional Morphology, Division of Cell Regeneration and Transplantation, Nihon University School of Medicine, Tokyo, Japan; 5 Center for Perinatal, Maternal and Neonatal Medicine, Niigata University Medical and Dental Hospital, Niigata, Japan; 6 Department of Pathology, Nagoya University Graduate School of Medicine, Nagoya, Japan

**Keywords:** Neonatology, Infant necrotizing enterocolitis, Preterm birth, Paediatric research

## Abstract

Neonatal necrotizing enterocolitis (NEC) is a serious disease of premature infants that necessitates intensive care and frequently results in life-threatening complications and high mortality. Dedifferentiated fat cells (DFATs) are mesenchymal stem cell-like cells derived from mature adipocytes. DFATs were intraperitoneally administrated to a rat NEC model, and the treatment effect and its mechanism were evaluated. The NEC model was created using rat pups hand fed with artificial milk, exposed to asphyxia and cold stress, and given oral lipopolysaccharides after cesarean section. The pups were sacrificed 96 h after birth for macroscopic histological examination and proteomics analysis. DFATs administration significantly improved the survival rate from 25.0 (vehicle group) to 60.6% (DFAT group) and revealed a significant reduction in macroscopical, histological, and apoptosis evaluation compared with the vehicle group. Additionally, the expression of C–C motif ligand 2 was significantly decreased, and that of interleukin-6 decreased in the DFAT group. DFAT administration ameliorated 93 proteins mainly related to proteins of fatty acid metabolism of the 436 proteins up-/down-regulated by NEC. DFATs improved mortality and restored damaged intestinal tissues in NEC, possibly by improving the abnormal expression of fatty acid-related proteins and reducing inflammation.

## Introduction

Neonatal necrotizing enterocolitis (NEC) is a major cause of morbidity and mortality in premature infants. Immature intestine and atypical immunity induced by microbial colonization due to cesarean section, enteral feeding with artificial milk, bacterial infection, and cardiorespiratory instability are factors associated with NEC^1,2^. Currently, parental nutrition without enteral feeding, antibiotics, and surgery (necrotic intestinal resection) are the only therapies for NEC. Some patients with serious conditions cannot undergo intestinal resection and must receive conservative therapy with or without intraperitoneal drainage. Survivors who develop short bowel syndrome after long segment resection of the necrotic intestine present poor nutrition that causes psychomotor retardation^3^. Therefore, developing a novel therapy to improve the outcome in neonates with NEC is necessary. Some stem cell therapies for several diseases have recently been reported^4^. Some animal experiences with stem cell therapy in an NEC model were reported, including bone marrow-derived mesenchymal stem cells (MSCs), human amniotic epithelial cells, human chorionic MSCs, human amniotic fluid stem cells (AF-derived stem cells), neonatal enteric neural stem cells, and others^5–8^. These stem cells decreased the survival rate and the severity of the NEC animal model, especially bone marrow-derived MSC and AF-derived stem cells^6^. However, it is invasive and/or obtaining these cells is limited and the mechanism of these treatments was unclear. Several reports have indicated the efficacy of adipose tissue-derived stem cells (ASCs)^9,10^. These cells have great advantages for preparation due to easy and safe adipose tissue access^9^. Dedifferentiated fat cells (DFATs) are derived from mature adipocytes via ceiling culture, thereby providing a large number of highly pure cells from a small amount of adipose tissue. ASCs are a heterogeneous population that contains smooth muscle cells, monocytes, vascular endothelial cells, and others, whereas DFATs are a highly homogeneous population of MSCs. Furthermore, DFATs produce a larger number of cells than ASCs^11–15^. Our research group revealed that DFATs exert a therapeutic effect on neonatal hypoxic–ischemic encephalopathy^16^, but ASCs do not^17^. A large number of homogeneous cells collected with easy techniques is necessary for regenerative medicine. Considering these advantages, DFATs may be ideal stem cells for NEC treatment among various kinds of stem cells. The present study aimed to evaluate the treatment effects of DFAT administration and revealed its mechanism using an NEC rat model.

## Results

### Survival rate and body weight

**Figure 1:**
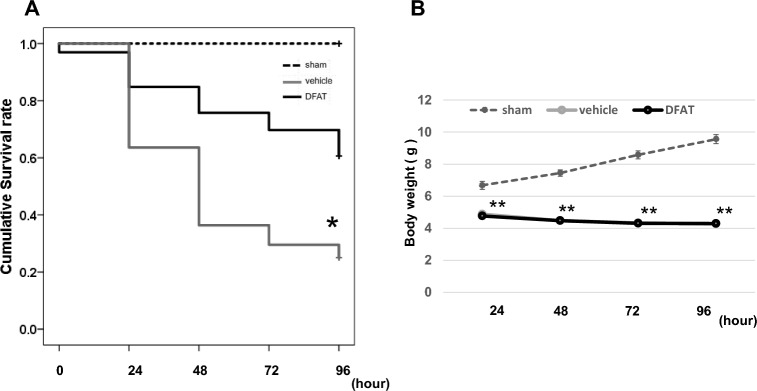
(**A**) Cumulative survival rate. Dotted line indicates the control group (sham), the gray line indicates the NEC model (vehicle), and the black line indicates the NEC model with DFAT. The cumulative survival rate of the vehicle was 25.0%, and DFAT was 60.6%. * *p* < 0.05 (sham vs vehicle, DFAT vs vehicle, sham vs DFAT). *NEC* necrotizing enterocolitis, *DFAT* dedifferentiated fat. (**B**) Body weight of rat pups. Dotted line indicates the control group (sham), the gray line indicates the NEC model (vehicle), and the black line indicates the NEC model with DFAT. Significant differences were found in the sham and vehicle and DFAT groups, but no difference between the vehicle and DFAT groups for the 96-h observation period. ** *p* < 0.01; n = 21 for sham, 37 for vehicle, and 30 for DFAT in (**A**,**B**). Data represent the mean ± S.E.M. *NEC* necrotizing enterocolitis, *DFAT* dedifferentiated fat.

We calculated the survival rate until 96 h after birth. All rats in the sham group survived until the end of the experiment. The survival rate in the NEC model (vehicle) was 25.0%, which was lower than that in the sham group, but DFAT administration significantly improved the survival rate to 60.6% (Fig. 1A, *P*< 0.05). Body weights were not significantly different between the NEC model administered DFAT cells and Ringer’s solution (Fig. 1B).

### Macroscopic evaluation

**Figure 2:**
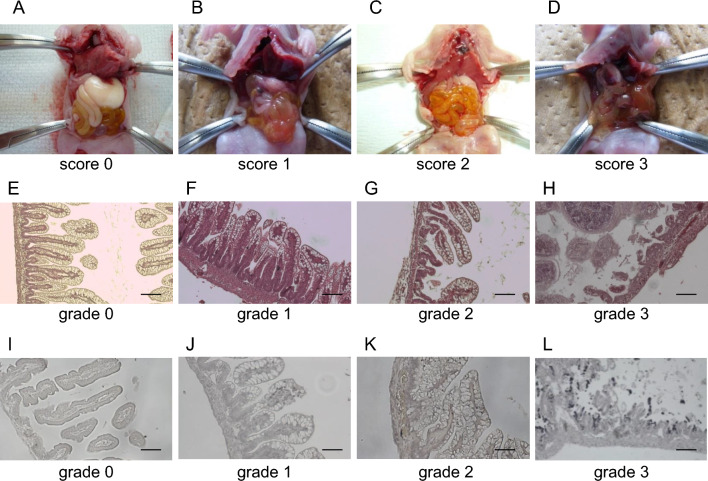
Evaluations using the scoring system. (**A**–**D**) Pictures representing the macroscopic evaluation of the intestine in each score: (**A**) score 0; normal intestine, (**B**) score 1; mild hemorrhage, (**C**) score 2; moderate hemorrhage, (**D**) score 3; severe hemorrhage. Representative pictures of the intestine in each group. (**E**–**H**) Pictures representing the evaluation of HE staining in each grade: (**E**) grade 0, no damage; (**F**) grade1, separation of villous core, without other abnormalities; (**G**) grade 2, villous core separation, submucosal edema, and epithelium sloughing; (**H**) grade 3, denudation of epithelium with loss of villous, full-thickness necrosis, or perforation. Scale bars indicate 100 µm. (**I**–**L**) Pictures representing H&E staining with active caspase-3 antibody in each grade. The tissues were graded as follows: (**I**) grade 0 (normal), no damage; (**J**) grade 1 (mild), apoptotic nuclei present at villous tips; (**K**) grade 2 (moderate), apoptotic nuclei covering all villous tips but crypts protected; (**L**) grade 3 (severe), the transmural spread of apoptotic nuclei. Scale bars indicate 100 µm. *H&E* hematoxylin and eosin.

**Figure 3:**
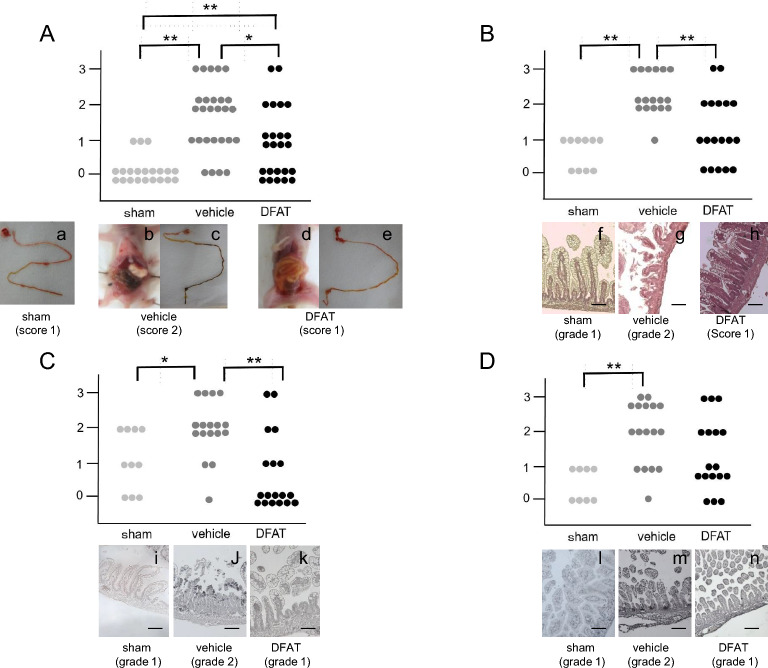
Macroscopic and histological evaluations. (**A**) Representative result of the macroscopic evaluation. (a) The sham (score 0) and (b,c) vehicle groups showed the color was changed from yellow to brown (score 2). (d,e) DFAT administration group (score 1). Significant differences were found between sham and vehicle, sham and DFAT, and vehicle and DFAT. * *p* < 0.05, ** *p* < 0.01; n = 21 for sham, 27 for vehicle, and 24 for DFAT. *DFAT* dedifferentiated fat. (**B**) Representative result of H&E staining in each grade. (f) Sham (grade1), (g) vehicle (grade2), and (h) DFAT administration groups (grade1). Significant differences were found between sham and vehicle, vehicle and DFAT. ** *p* < 0.01; n = 10 for sham, 17 for vehicle, and 18 for DFAT. *DFAT* dedifferentiated fat. (**C**) Representative pictures of H&E staining with active caspase-3 antibody in each grade. (i) Sham (grade 1), (j) vehicle (grade 2), and (k) DFAT administration group (grade 0). A significant difference was found between the vehicle and DFAT groups in caspase-3. *p < 0.05, ** *p* < 0.01; n = 10 for sham, 17 for vehicle, and 18 for DFAT. *DFAT* dedifferentiated fat. (**D**) Representative pictures of TUNEL staining in each grade. (l) Sham (grade 0), (m) vehicle (grade 2), and (h) DFAT administration groups (grade 1). A different trend was found between the vehicle and DFAT groups. *p < 0.05, ** *p* < 0.01; n = 8 for sham, 17 for vehicle, and 17 for DFAT. *DFAT* dedifferentiated fat. *H&E* hematoxylin and eosin. Scale bars indicate 100 µm.

We visually evaluated the intestine when we opened the rats’ abdomens. A normal intestine or score of 1 (Fig. 2) was found in the sham group. The intestine changed to brown or black in more than half of the vehicle group because of necrosis and/or hemorrhage (scores of 2 or 3, Fig. 3A). A significant reduction was found in the median histological score in the DFAT group compared with that in the vehicle group (*P* < 0.05; Fig. 3A).

### Histological evaluation

We evaluated the intestinal tissues using hematoxylin and eosin (H&E) staining. Histological analysis was performed using the grading system. Moderate (grade 2) or severe (grade 3) damage was seen in the intestine of all except one pup in the vehicle group (Fig. 3B). However, more than half the pups showed mild or no damage in the DFAT group. The degree of intestinal damage in the DFAT group was significantly lower than that in the vehicle group (*P* < 0.01; Fig. 3B). Apoptosis evaluation was performed using a grading system according to immunohistochemical procedures (active caspase-3 antibody) and TUNEL staining. The immunohistochemical staining with active caspase-3 antibody revealed a significantly lower grade in the DFAT group than in the vehicle group (*P* < 0.01; Fig. 3C). The DFAT group revealed a lower grade of apoptosis evaluation with TUNEL staining compared to the vehicle group (Fig. 3D).

### Proteomics

**Figure 4:**
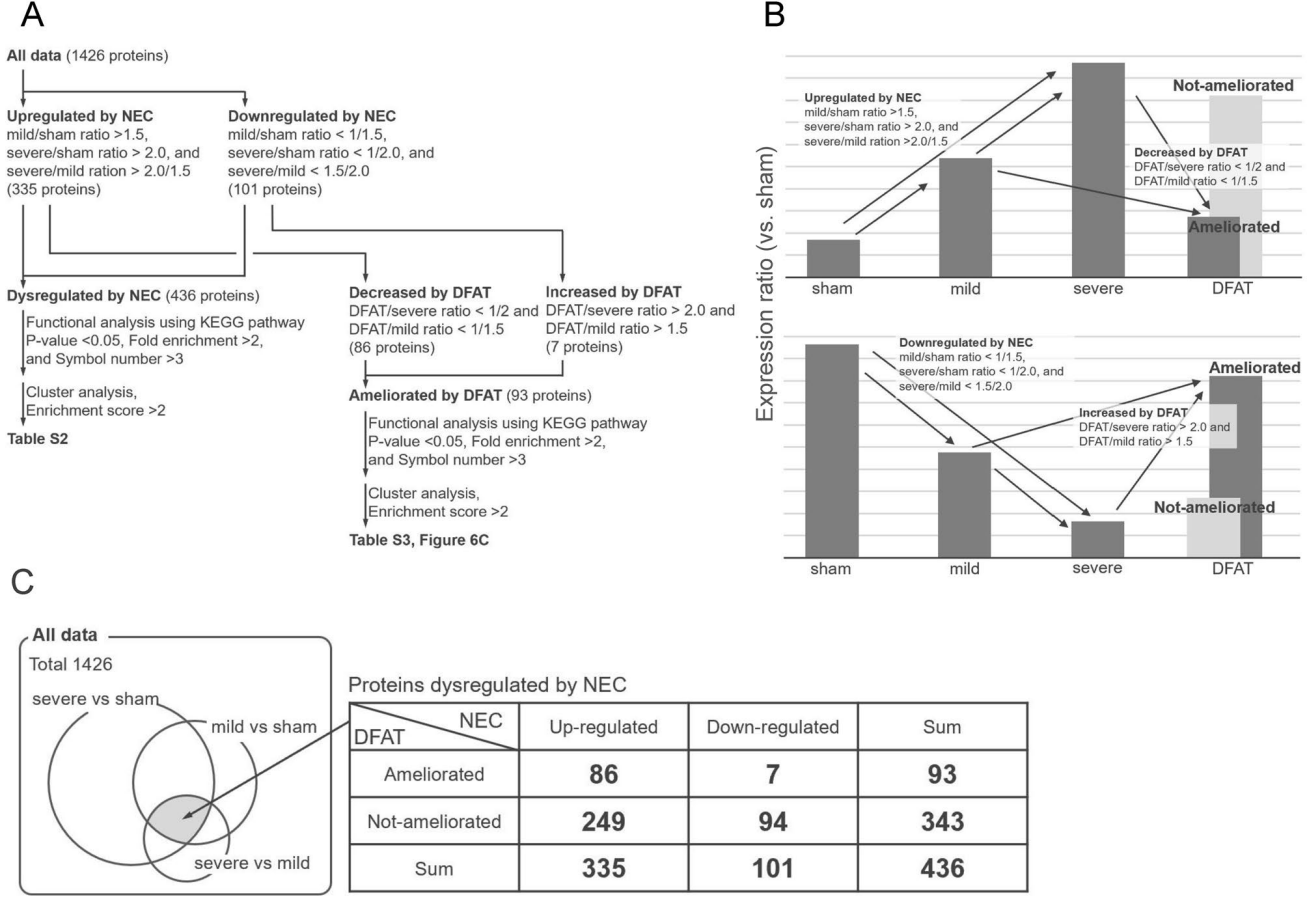
Visualization of the analytical process of proteomics. (**A**) Flowchart of the analytical process and extraction criteria for the ileum proteins that were abnormally expressed by NEC and were ameliorated by DFAT. (**B**) Visualization of the extraction criteria. (**C**) Venn diagram and table of the number of extracted proteins. n = 3 for each group. *NEC* necrotizing enterocolitis, *DFAT* dedifferentiated fat.

Protein profiles of ileum tissue 96 h after birth were obtained by proteomics to comprehensively evaluate the changes in protein expression patterns caused by NEC (Fig. 4A,B). A total of 1,426 proteins revealed a high-quality signal and were quantified by liquid chromatography/tandem mass spectrometry (LC/MS/MS). Among the 1,426 proteins, 335 exhibited up-regulation (Fig. 4A,B; mild/sham ratio of > 1.5, severe/sham ratio of > 2.0, and severe/mild ratio of > 2.0/1.5) and 101 exhibited downregulation (Fig. 4A,B; mild/sham ratio of < 1/1.5, severe/sham ratio of < 1/2.0, and severe/mild ratio of < 1.5/2.0). A total of 436 proteins revealed abnormal expression depending on NEC severity (Table S2).

We refined the fluctuations in the protein profiles of ileum tissue in response to NEC using the data of the DFAT cell group to detect proteins with expression ameliorated by DFAT cell administration (Fig. 4). The ileum proteins dysregulated by NEC were divided into ameliorated and non-ameliorated proteins by DFAT cell administration. Regarding the 335 proteins exhibiting up-regulation by NEC, ameliorated proteins are those that showed a downregulation of 1/twofold in the DFAT cell group with the severe NEC group and 1/1.5-fold in the DFAT cell group with the mild NEC group (Fig. 4A–C). Likewise, regarding the 101 proteins exhibiting downregulation by NEC, ameliorated proteins are those that showed a twofold up-regulation in the DFAT cell group compared with the severe NEC group and 1.5-fold in the DFAT cell group compared with the mild NEC group (Fig. 4A–C). Proteins that did not fulfill the aforementioned conditions were classified as non-ameliorated proteins. Of 436 proteins extracted from the protein profile as protein severity-dependently dysregulated by NEC, 93 proteins (Fig. 4C; proteins upregulated by NEC, 86; proteins down-regulated by NEC, 7) were ameliorated by DFAT cell injection (Table S3), whereas 343 proteins (Fig. 4C; proteins upregulated by NEC, 249; proteins down-regulated by NEC, 94) were not ameliorated by DFAT cell injection (Table S4).

### Functional analysis

**Figure 5:**
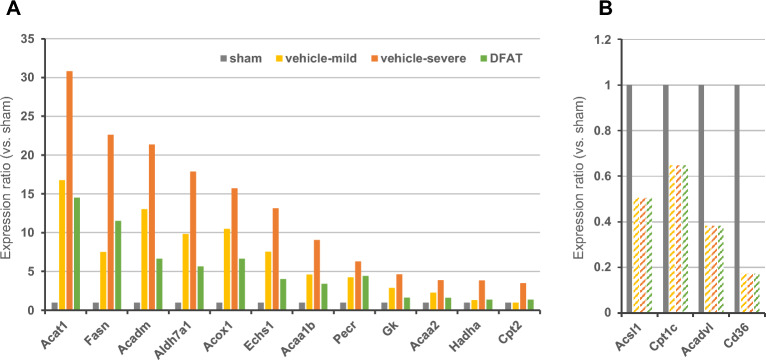
Ileum proteins extracted by functional analysis using the KEGG pathway^18^. Relative expression levels of proteins significantly enriched in the 3 terms associated with “fatty acid metabolism,” “fatty acid degradation,” and “PPAR signaling pathway.” (**A**) Upregulated proteins and (**B**) down-regulated proteins by neonatal necrotizing. The bar of vehicle-mild, vehicle-severe, and DFAT (diagonal lines) indicate the detection limit. *DFAT* dedifferentiated fat.

We performed the KEGG pathway^18^ with clustering analysis using the DAVID bioinformatics database to reveal adverse outcome pathways of NEC and the relationship between ileum functional denaturation recovery and protein expression amelioration by DFAT cells. The functional analysis of the ileum proteins dysregulated by NEC (436) was significantly enriched in three terms associated with “Fatty acid metabolism,” “Fatty acid degradation,” and “PPAR signaling pathway” (Table 1) in one cluster. In the three terms, 12 proteins (Acat1, Fasn, Acadm, Aldh7a1, Acox1, Echs1, Acaa1b, Pecr, Gk, Acaa2, Hadha, and Cpt2) revealed severity-dependent upregulation (Fig. 5A), whereas four proteins (Acsl1, Cpt1c, Acadvl, and Cd36) revealed severity-dependent downregulation caused by NEC (Fig. 5B). Furthermore, the functional analysis with the KEGG pathway revealed that the protein group ameliorated by DFAT cells (93 proteins) was significantly enriched in three terms related to “valine, leucine, and isoleucine degradation,” “butanoate metabolism,” and “synthesis and degradation of ketone bodies” in cluster 1, and four terms related to “valine, leucine, and isoleucine degradation,” “fatty acid degradation,” “beta-Alanine metabolism,” and “biosynthesis of antibiotics” in cluster 2 (Table 2). Notably, the term “fatty acid degradation” was commonly extracted from the protein group dysregulated by NEC and the protein group ameliorated by DFAT cells. The term included four proteins (Acadm, Aldh7a1, Acox1, and Echs1) (Table 2 and Fig. 5A).

**Table 1:** KEGG pathway^18^　terms related to proteins dysregulated by neonatal necrotizing enterocolitis.

Cluster	Term ID	Term	*P*-value	Fold enrichment	Proteins
1 (Enrichment score: 4.54)	rno01212	Fatty acid metabolism	0.0000007	6.22	Acat1, Fasn, Acadm, Acox1, Echs1, Acaa1b, Pecr, Acaa2, Hadha, Cpt2, Acsl1, Cpt1c, Acadvl
	rno00071	Fatty acid degradation	0.0000012	6.60	Acat1, Acadm, Aldh7a1, Acox1, Echs1, Acaa1b, Acaa2, Hadha, Cpt2, Acsl1, Cpt1c, Acadvl
	rno03320	PPAR signaling pathway	0.028	2.68	Acadm, Acox1, Acaa1b, Gk, Cpt2, Acsl1, Cpt1c, Cd36

**Table 2:** KEGG pathway^18^　terms related to proteins with abnormal expression due to neonatal necrotizing enterocolitis ameliorated by the administration of dedifferentiated fat cells.

Cluster	Term ID	Term	*P*-value	Fold enrichment	Proteins
1 (Enrichment score: 3.47)	rno00280	Valine, leucine, and isoleucine degradation	0.0000063	14.72	Hmgcl, Hmgcs2, Oxct1, Aldh7a1, Echs1, Ivd, Acadm
	rno00650	Butanoate metabolism	0.0017	16.52	Hmgcl, Hmgcs2, Oxct1, Echs1
	rno00072	Synthesis and degradation of ketone bodies	0.0037	31.54	Hmgcl, Hmgcs2, Oxct1
2 (Enrichment score: 2.56)	rno00280	Valine, leucine, and isoleucine degradation	0.0000063	14.72	Hmgcl, Hmgcs2, Oxct1, Aldh7a1, Echs1, Ivd, Acadm
	rno00071	Fatty acid degradation	0.0073	9.84	Acadm, Aldh7a1, Acox1, Echs1
	rno00410	Beta-alanine metabolism	0.034	10.21	Acadm, Aldh7a1, Echs1
	rno01130	Biosynthesis of antibiotics	0.038	3.17	Fnta, Hmgcs2, Aldh7a1, Echs1, Acadm, Aco2

Among these proteins upregulated by NEC and ameliorated by DFAT, three proteins (Acat1, Fasn, and Acadm) were confirmed by electrophoresis immunoassay for their upregulated expression and DFAT amelioration (Supplemental Figure).

### Cytokines/chemokines

**Figure 6:**
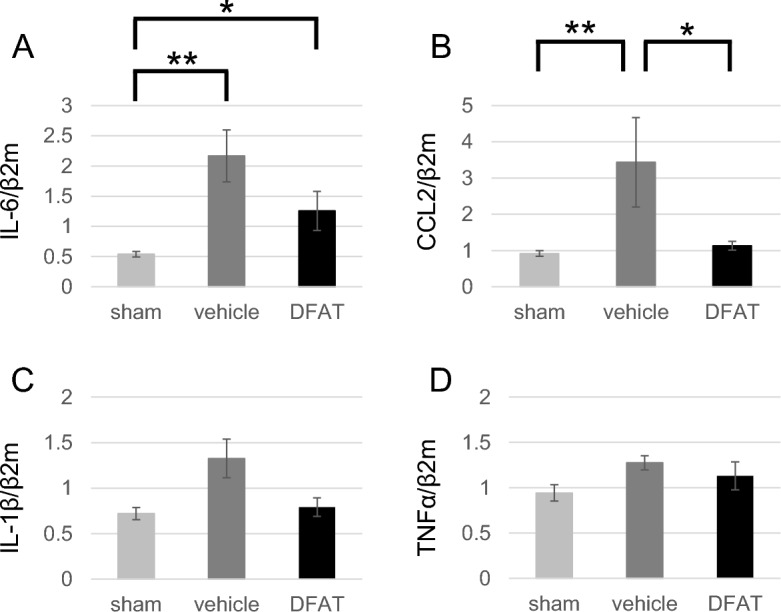
Inflammatory cytokine/chemokine. Gene expression of IL-6 (**A**), CCL2 (**B**), IL-1β (**C**), and TNFα (**D**). Both IL-6 and CCL2 expressions were significantly increased in the vehicle group, CCL2 expression was significantly decreased in the DFAT group, and IL-6 expression tended to decrease. * *p* < 0.05, ** *p* < 0.01; n = 17 for sham, 27 for vehicle, 13 for DFAT in A. n = 17 for sham, 28 for vehicle, 13 for DFAT in B n = 17 for sham, 27 for vehicle, 12 for DFAT in C. n = 10 for sham, 16 for vehicle, 7 for DFAT in D. Data represent the mean ± S.E.M. *CCL2* C–C Motif Chemokine Ligand 2, *DFAT* dedifferentiated fat, *IL* interleukin, *TNFα* tumor necrosis factor-alpha.

Cytokines and chemokines regulating inflammation in this disease were measured using real-time reverse transcription polymerase chain reaction (RT-PCR). Interleukin (IL)-6 is a key inflammation regulator of sepsis in NEC, and the C–C Motif Chemokine Ligand 2 (CCL2) is induced from the bone marrow to migrate white blood cells. Both IL-6 and CCL2 expressions were significantly increased in the vehicle group (*P* < 0.01), and CCL2 expression was significantly decreased in the DFAT group (*P* < 0.05). IL-6 expression decreased, but with no significant difference between the vehicle and DFAT groups (Fig. 6A,B). IL-1β and tumor necrosis factor-alpha (TNFα) in other inflammatory cytokines were higher in the vehicle group than in the sham group and decreased in the DFAT group, although with no significant differences among the three groups (Fig. 6C,D).

**Figure 7:**
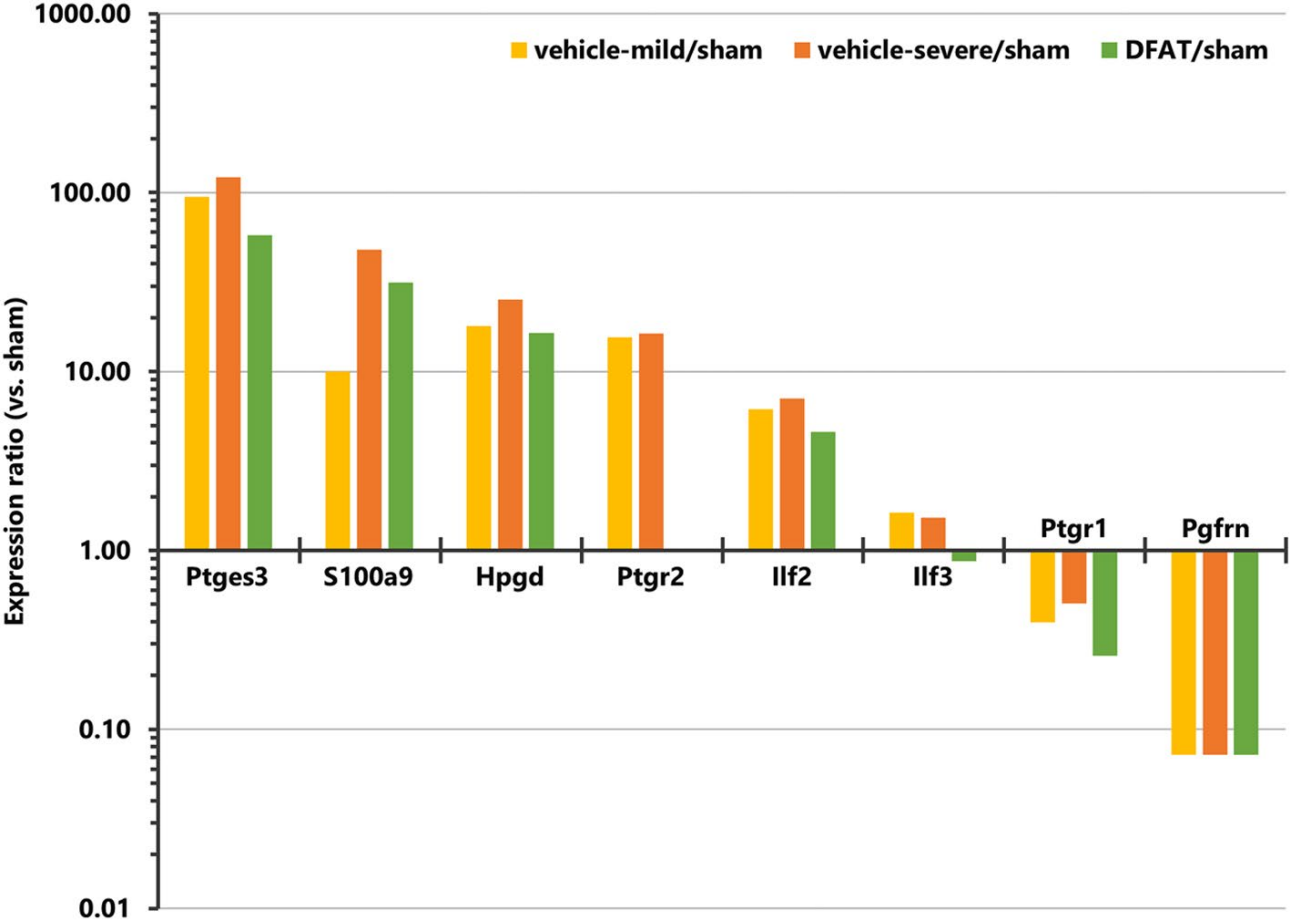
Inflammatory response-related proteins extracted from proteomics data. Relative expression levels of the proteins are categorized in inflammatory response-related protein terms of Gene Ontology when the expression levels of the sham group are set as 1.

Moreover, the expression levels of intestinal proteins, categorized in the inflammatory response-related protein term of Gene Ontology, were confirmed by proteomic data. Prostaglandin E synthase 3 (Ptges3/p23), S100a9 (MRP14), 15-Hydroxyprostaglandin dehydrogenase (Hpgd), prostaglandin reductase 2 (Ptgr2), IL enhancer-binding factor 2 (Ilf2), and IL enhancer-binding factor 3 (Ilf3), which promote inflammation and/or increase with inflammation^19–24^, were increased in the vehicle (NEC) group compared to the control group (Fig. 7). Additionally, Ptgr1, which has an anti-inflammatory function^25^, and prostaglandin F2 receptor negative regulator (Ptgfrn), which acts as an inhibitor of prostaglandin signaling^26^, were decreased in the vehicle (NEC) group. The abnormal expression of Ptges3, Hpgd, Ptgr2, and Ilf3 in the eight proteins dysregulated by NEC was improved by DFAT administration. Furthermore, the abnormal expression of four proteins (Ptges3, Hpgd, Ptgr2, and Ilf3) in the eight inflammatory response-related proteins that were abnormally expressed by NEC was improved by DFAT treatment (Fig. 7).

### DFAT cells engraftment

We performed immunohistostaining with Green fluorescent protein (GFP) antibody using frozen sections of the NEC intestine to evaluate DFAT cell engraftment labeled with GFP. However, GFP-positive cells were not observed in the intestine of the DFAT group.

## Discussion

This study revealed the treatment effects of DFAT cells in an experimental NEC model of newborn rats. The findings supported improvement in survival and macroscopic and histological immunohistochemical evaluations in intestinal damage. The proteomics and functional analysis revealed that proteins related to fatty acid metabolism were mostly fluctuated by NEC and may be key molecules of the biological pathways in NEC pathogenesis. Abnormal expression of fatty acid metabolism-related proteins was mostly ameliorated by DFAT administration. Additionally, inflammatory cytokines/chemokines were inhibited by DFAT therapy. The effect of DFATs was considered via trophic effects, which was supported but not found in the GFP-positive DFAT cells in the intestine of the NEC model.

We performed proteomics analysis with the injured intestine of the NEC model rat (vehicle, DFAT administration, and sham groups) using LC–MS/MS and revealed that changes in fatty acid-related protein expression were most prominent in NEC, and many were ameliorated by DFAT (Fig. 5A). A previous study revealed that enterobacteria used unavailable carbohydrates (dietary fiber) as an important nutrient. Short-chain fatty acids made by metabolism handle the host immune and metabolism systems as important signaling molecules. For example, acetic acid, a short-chain fatty acid that prevents pathogenic *Escherichia coli* O-157 infection, improves enteric barrier function^27^ and promotes FoxP3 expression of a Treg cell translation factor, thereby improving enterocolitis^28^. Furthermore, enterobacteria produce several fatty acids, hydroxyl fatty acid, oxo fatty acid, partially carbonated non-methylene fatty acids, and conjugated linoleic acid in mice tissues^27^. These associated hydrated fatty acids prevent enteric barrier function^27^, fatty acid synthesis^29^, and immune system control. Their function indicates the possibility that fatty acids produced by enterobacterium affect host health as a signaling factor.

Intestinal fatty acid-binding protein (IFABP), which is secreted by small intestine simple columnar epithelial cells, was highly expressed in the NEC model, and its expression correlated with the degree of tissue injury^30^. Polyunsaturated fatty acid supplementation alters proinflammatory gene expression and reduces NEC incidence^31^. Additionally, systematic reviews indicated the possibility of IFABP as a biomarker for early NEC diagnosis^32,33^. Therefore, fatty acids and their related protein play very critical roles in the pathophysiology of NEC and are targets for developing novel NEC therapies. Furthermore, the mitigation of fatty acid metabolism-related protein dysregulation by DFAT administration suggests that these proteins are likely to be key factors in understanding the therapeutic mechanisms of DFATs at the molecular level.

Additionally, the inflammatory response plays an important role in developing NEC^30^. We evaluated the inflammatory cytokines/chemokines, IL-6 and CCL2, using real-time PCR. CCL2 and IL-6 were reduced in the NEC model by DFAT administration. IL-6 is related to various effects, including (1) blood stem cell differentiation and proliferation, (2) mature megakaryocyte promotion for platelet production, (3) T-cell and neural stem cell differentiation, and (4) acute activation proteins (CRP and haptoglobin) and acute phase response factor (STAT3) production^34^. Conversely, CCL2, which is induced in bone marrow, belongs to the monocyte chemotactic protein family that affects monocyte migration^35^. The finding that DFATs suppressed the IL-6 and CCL2 levels indicated that DFAT cells inhibited intestinal inflammation and/or promoted intestinal regeneration in the NEC rat model, leading to intestinal damage amelioration/prevention and survival rate improvement.

Other inflammatory response-related proteins extracted from proteomics data supported that inflammation is important in developing NEC and DFAT cells, partially inhibiting inflammation (Fig. 7). Additionally, this analysis indicated that NEC particularly induced protein dysregulation in prostaglandin metabolism. Prostaglandin is stimulated by inflammatory conditions, has high pharmacological activity in inducing acute inflammation, and is one of the types of unsaturated fatty acids synthesized through fatty acid metabolism^26,36^. Inflammation and fatty acid metabolism are strongly linked via prostaglandin. The functional analysis of the proteomic data using KEGG pathway in the present study revealed that expression of fatty acid metabolism-, fatty acid degradation-, and PPAR signaling pathway-related proteins are most significantly affected by NEC. Thus, NEC-induced abnormalities in fatty acid metabolism may be associated with altered expression of prostaglandin-related proteins. Moreover, the results revealed that NEC-induced expression changes of these fatty acid metabolism-related and prostaglandin-related proteins were ameliorated by DFAT. Therefore, both inflammation and abnormal fatty acid metabolism may be important biological responses in understanding the pathogenesis of NEC and the efficacy of DFAT cells at the molecular level, suggesting that prostaglandin may be the key molecule linking them. However, future studies are needed to evaluate prostaglandin expression in NEC and investigate its role.

The results related to survival, intestine structure, and apoptosis evaluations revealed significant improvement with DFAT therapy in the NEC model. NEC severity (score of > 2 or grade 2) was macroscopically reduced from 54 to 29%, from 94 to 39% in HE staining, and from 65 to 28% in apoptosis evaluations. A previous study revealed the reduced NEC incidence and severity from 61 to 19–23% histologically with other stem cells, including bone marrow-derived MSCs^6^, which indicates the same degree of treatment effect as ours. However, DFATs have several advantages compared with other stem cells. First, DFATs provide us with a large number of highly pure cells from a small amount of adipose tissue in a short period^12–15^. Second, DFATs are procured from adipose tissue of all donor ages because the proliferation ability of DFATs does not change depending on the donor’s age^12^. In contrast, the proliferation rate of other MSCs changes by donor age^37^. Third, DFATs are more purified cells than ASCs from adipose tissue with an expected clinically consistent effect. Fourth, DFATs were more easily obtained than bone marrow-derived-MSCs, and DFATs were obtained from subcutaneous fat cells that would be discarded. These facts indicate the superiority of DFATs in clinical use, even with the same treatment effect as that of other stem cells. Regarding tissue-derived stem cells, including DFAT, interdonor variability likely occurs, and avoiding it will require a rigorous quality control system. MSC derived from induced pluripotent stem cells may overcome the problem due to their capacity for multilineage differentiation and indefinite proliferation^38,39^, and a phase 1 clinical trial for acute steroid-resistant graft versus host disease has already been performed^40^. However, currently, the cost of preparation for clinical-grade cells remained very high and much higher than those for stem cells without gene transfer, including DFAT^41^. Therefore, development using DFAT would be important to establish a treatment as general medicine as soon as possible.

This study has some limitations. First, we did not evaluate the use of fatty acid expression changes for the treatment effect of DFAT although fatty acid expression changes induced by NEC were less in the DFAT group. The function/role of the fatty acids remained unknown in DFAT treatment. Therefore, further studies are needed to clarify the function/role of fatty acids. Second, we could not evaluate the long-term effect of DFAT treatment. Our model will hardly survive for long periods due to technical difficulties. Additionally, we were unable to examine the effect of treatment started at later time points due to the model limitation. Identifying the long-term effect and the therapeutic windows is important for clinical application. Therefore, we need to develop another NEC model, which survives for long periods.

In conclusion, DFATs significantly reduce the incidence and severity of NEC with beneficial effects on ameliorating abnormal fatty acid-related protein expressions and reducing inflammation. DFATs represent a possibility as a novel therapy to improve mortality and repair the damaged intestinal tissues in NEC. Further studies are required to investigate the long-term results of intestinal tissue and safety with DFAT therapy in NEC.

## Materials and methods

An expanded version of the Materials and Methods is available in the online Supplement.

All animal experiments were approved by the Institutional Review Board of Nagoya University School of Medicine (permit No. 28139, 29383, 30078), and conducted following the Regulations on Animal Experiments in Nagoya University. This study is reported in compliance with the Animal Research: Reporting in *Vivo* Experiments guidelines.

### DFAT cell preparation

GFP-labeled DFATs^42^ were prepared from subcutaneous adipose tissue in GFP transgenic rats using a ceiling culture method^11^ (Sprague–Dawley YgN [act-EGFP] OsbCZ-004). Cells were cultured in Dulbecco’s modified Eagle’s medium (Invitrogen, Carlsbad, CA, USA) with 20% (in the dedifferentiated phase) or 10% (in the proliferation phase) fetal bovine serum, where cells were incubated at 37 °C with 5% CO_2_.

### Animals and experimental design

Neonatal rats were delivered from pregnant Sprague–Dawley dams via cesarean section on embryonic day 21. Then, they were housed in an incubator (temperature: 34 °C, humidity: 70%–90%). Rat pups were hand-fed three times daily with 0.2 mL high osmotic condition milk, and were subjected to asphyxia (100% CO_2_ gas for 10 min) and cold stress (4 °C for 5 min), followed by recovery (100% oxygen gas for 5 min) two times daily. Furthermore, lipopolysaccharides from *Escherichia coli* 0111:B4 (Sigma-Aldrich, St. Louis, MO, USA) at 3 mg/kg were administered by feeding at 2 and 38 h after birth.

The rat pups were allocated to three groups as follows: NEC + Ringer’s solution at 50 µL intraperitoneal injection (IP) (vehicle); NEC + DFAT, 1.0 × 10^6^ cells in 50 µL of Ringer’s solution IP at 32 and 52 h after birth (DFAT); natural vaginal delivered rat pups were fed mother’s milk as a control (sham). The pups were weighed daily and recorded alive or dead and sacrificed 96 h after birth.

### Macroscopic evaluation

Pups were euthanized with pentobarbital sodium. The intestine was visually evaluated upon abdominal cavity opening. Typical signs of NEC were evaluated using a scoring system reported by Yan-Nan Jiang et al.^43^ with minor modification as follows: 0, indicating normal intestine (absence of macroscopic hemorrhage, edema, or mucosal abnormality); 1, indicating local hyperemia and hyperemia, extensive edema, and local hemorrhage; 2, indicating extensive hemorrhage and local necrosis and pneumatosis intestinalis; and 3, indicating extensive transmural necrosis and pneumatosis intestinalis. The intestinal color that changed from yellow to dark brown was allocated a score of 2 (Fig. 2A–D).

### Tissue preparation

Sections of 2 cm at the end of the ileum were fixed overnight in 4% paraformaldehyde (PFA) and embedded in paraffin. After fixed PFA, tissues were followed by 20%–30% sucrose for each 24 h and were embedded in an OTC compound to make frozen sections for immunohistostaining with GFP antibody.

### Histological and immunohistochemical procedures

Tissue structure evaluation was performed with H&E staining. We modified the scoring of Guven et al*.*^44^ as follows: 0 (normal), no damage; 1 (mild), separation of the villous core with no other abnormalities; 2 (moderate) villous core separation, submucosal edema, and epithelium sloughing; and 3 (severe), denudation of the epithelium with loss of villous, full-thickness necrosis, or perforation (Fig. 2E–H).

Additionally, apoptosis was evaluated using an active caspase-3 antibody and TUNEL staining. Tissues were graded 0 (normal, no apoptotic change), 1 (mild, apoptotic nuclei present at villous tips), 2 (moderate, apoptotic nuclei covering all villous tips but crypts protected), and 3 (severe, the transmural spread of apoptotic nuclei) (Fig. 2I–L)^44^.

Antigen retrieval was performed by incubation with 0.01-M citric acid monohydrate (Fujifilm Wako Pure Chemical Corporation, Osaka, Japan) for 10 min at 90 °C with heating to evaluate apoptosis using an active caspase-3 antibody. Sections were incubated with the primary antibody, Purified Rabbit Anti Active Caspase-3 (dilution 1:200; BD Biosciences, Franklin Lakes, NJ, USA) in a blocking solution at 4 °C overnight, after incubation with a blocking solution of 4% normal donkey serum (Jackson Immuno Research, Baltimore, PA, USA) with 10% Triton-100 (MP Biomedicals, Inc., Sanata Ana, CA, USA) for 30 min. Sections were incubated with a secondary antibody, Biotin-SP-Conjugated AffiniPure donkey Anti Rabbit IgG (dilution 1:400; Jackson Immuno Research), on the second day. Sections were incubated with 3% H_2_O_2_ (Takara Bio Inc., Shiga, Japan) for 10 min to quench endogenous peroxidase activity. The binding was visualized with the Vectastain Elite ABC Standard Kit (Vector Laboratories, Burlingame, CA, USA), followed by peroxidase detection for 10 min (0.12 mg/mL 3,3′-diaminobenzidine, 0.01% H_2_O_2_, and 0.04% NiCl_2_). Sections were mounted with NEW M・X (Misumi Corporation, Tokyo, Japan).

Sections were incubated with 20 µg/mL of proteinase K (Roche, Mannheim, Germany) and 3% H_2_O_2_ to evaluate apoptosis using TUNEL stain. In Situ Cell Death Detection Kit, POD (Roche Diagnostics, Mannheim, Germany) was used, and the reaction solution, which was prepared by mixing the enzyme and label solutions, was added to the sample. Then, they were incubated with Converter-POD. The slides were stained with 3,3′-Diaminobenzidine solution (0.12 mg/mL 3,3′-diaminobenzidine, 0.01% H_2_O_2_, and 0.04% NiCl2), then mounted with NEW M・X. Frozen sections were incubated with a monoclonal antibody Rb anti-GFP (1:200, Medical & Biological Laboratories, Tokyo, Japan), followed by donkey anti-Rb IgG-555 (1:500). Sections were mounted using Prolong Gold with DAPI (ProLong Gold antifade reagent with DAPI by Life Technologies) to evaluate DFAT engraftment.

### Proteomics

Multi-beads shocker® (cell disruptor: Yasui Kikai Corporation, Osaka, Japan) with liquid N_2_ was used to homogenize the ileum tissue at 0.05 g. These samples were put in detergent-free lysis buffer (Minute TM Detergent-Free Protein Extraction Kit for Animal Cultured Cells and Tissues: Funakoshi Co., Ltd., Tokyo, Japan) with protease inhibitor (Complete Mini-EDTA-free: Sigma-aldrich, MO, USA). The homogenates were centrifuged at 21,130 rcf for 1 min to remove debris, and then, the supernatants were collected as protein lysates. The total protein concentration was quantified using the Pierce BCA Protein Assay Kit (Thermo Fisher Scientific). They were pooled and adjusted to 100 µg/200 µL for LC/MS/MS and stored at − 80 °C in a deep freezer until use.

The concentration of all types of proteins was quantified via LC/MS/MS. An Orbitrap Fusion mass spectrometry system (Thermo Fisher Scientific) was used in combination with UltiMate3000 RSLCnano LC system (Dionex Co., Amsterdam, the Netherlands) with a nano HPLC capillary column (150 mm × 75 μm i.d., Nikkyo Technos Co., Tokyo, Japan) via a nanoelectrospray ion source. A linear gradient flow rate (0 min, 5% B; 100 min, 40% B) of solvent A (2% acetonitrile with 0.1% formic acid) and solvent B (95% acetonitrile with 0.1% formic acid) was set at 300 nL/min. A 400–1600 mass-to-charge ratio (m/z) was used to perform precursor ion scans. MS/MS was performed via quadrupole isolation at 0.8 Th, HCD fragmentation at 30% normalized collision energy, and rapid scan MS analysis in an ion trap. Only precursors with charge states 2–6 were sampled for MS2. The dynamic exclusion time was set to 15 s with a tolerance of 10 ppm. The instrument was run in maximum speed mode with a 3-s cycle. The proteome software Scaffold (version 4.4.8, Proteome Software Inc., Portland, OR, USA) was used to validate peptide and protein identifications. Proteome Discoverer 1.4 (Thermo Fisher Scientific) and the MASCOT search engine (version 2.6.0, Matrix Science Inc., Boston, MA, USA) was used to analyze data. We referred to the protein database in UniProt (release 2019_06) and set a precursor mass tolerance and a fragment ion mass tolerance as 10 ppm and 0.8 Da, respectively. The primary proteome data (Table S1) were submitted to the Japan Proteome Standard Repository/Database^45^. The accession number is jPOST: JPST001143 (PXD025647).

### Protein extraction and functional analysis

Proteins dysregulated by NEC were extracted by threshold levels as follows: protein severity-dependently upregulated by NEC (mild/sham: > 1.5-fold, severe/sham: > 2.0-fold, and severe/mild ratio: > 2.0/1.5) and protein severity-dependently down-regulated by NEC (mild/sham ratio: < 1/1.5, severe/sham ratio: < 1/2.0, and severe/mild: < 1.5/2.0). Dysregulated proteins were analyzed to identify the proteins ameliorated by DFAT cells as follows: proteins in which NEC up-regulation was ameliorated (DFAT/severe ratio: < 1/2 and DFAT/mild ratio: < 1/1.5) and proteins in which NEC downregulation was ameliorated (DFAT/severe ratio: > 2.0 and DFAT/mild ratio: > 1.5). The extracted proteins were annotated with the KEGG pathway using DAVID 6.8 bioinformatics resources (https://david.ncifcrf.gov/home.jsp; 2019_06)^46^. The flagged KEGG pathway term was processed with a functional annotation clustering on the DAVID. Finally, we extracted the KEGG pathway terms with a *P*-value of < 0.05, fold enrichment of > 2, symbol number of > 3, and cluster enrichment score of > 2 as significant terms.

### Capillary electrophoresis immunoassay

Protein expression was evaluated by electrophoresis immunoassay (JESS) using the same proteins found by proteomics with functional analysis. The immunoassay was performed on the JESS system (ProteinSimple, San Jose, CA, USA). Proteins were identified by specific antibodies: rabbit anti-Acat1 (dilution 1:50; Proteintech, Rosemont, IL, USA), rabbit anti- Fasn (dilution 1:10; Proteintech), and rabbit anti-Acadm (dilution 1:10; Proteintech), in the capillary system, and the system measured and captured their chemiluminescence reactions as digital blot images. The expression of each protein was normalized with total protein abundance in the same capillary.

### Real-time PCR

Total RNA was extracted from the end of the ileum tissue at 0.05 g (snap frozen in liquid N_2_) after homogenization using the Multi-beads shocker®. The RNA concentration was quantified using ultraviolet spectrophotometry at A260, and the purity was determined by the 260/A280 ratio using NanoDrop™ One/One^C^ (Thermo Fisher Scientific, Waltham, MA, USA). Complementary DNA (cDNA) was synthesized from total RNA, and a quantitative real-time PCR was performed.

### Statistical analysis

Statistical analyses were conducted using IBM Statistical Package for the Social Sciences version 24 (IBM Japan, Tokyo, Japan) or Statcel 4 (Seiun-sha, Tokyo, Japan). Survival was estimated with the Kaplan–Meier method and compared using the log-rank test. The Kruskal–Wallis analysis, followed by the Steel–Dwass test, was used when three groups were compared for body weight, macroscopic, histological evaluation, and real-time PCR. One-way analysis of variance, followed by Holm–Šídák’s multiple comparisons test, was used to assess protein expression in electrophoresis immunoassay. The significance threshold was *P*-values of < 0.05.

## Supplementary Information

Supplementary Information 1.

Supplementary Information 2.

Supplementary Table S1.

Supplementary Table S2.

Supplementary Table S3.

Supplementary Table S4.

## Data Availability

The datasets of proteomics generated and/or analyzed during the current study are available in the jPOST, JPST001143 (PXD025647). The other datasets and materials are available from the corresponding author upon reasonable request.

## References

[1] Hunter CJ, Upperman JS, Ford HR, Camerini V (2008). Understanding the susceptibility of the premature infant to necrotizing enterocolitis (NEC). Pediatr. Res..

[2] Gregory KE, Deforge CE, Natale KM, Phillips M, Van Marter LJ (2011). Necrotizing enterocolitis in the premature infant: Neonatal nursing assessment, disease pathogenesis, and clinical presentation. Adv. Neonatal. Care.

[3] Blakely ML, Gupta H, Lally KP (2008). Surgical management of necrotizing enterocolitis and isolated intestinal perforation in premature neonates. Semin. Perinatol..

[4] Hattori T (2015). Administration of umbilical cord blood cells transiently decreased hypoxic-ischemic brain injury in neonatal rats. Dev. Neurosci..

[5] McCulloh CJ (2017). Evaluating the efficacy of different types of stem cells in preserving gut barrier function in necrotizing enterocolitis. J. Surg. Res..

[6] Drucker NA (2018). Stem cell therapy in necrotizing enterocolitis: Current state and future directions. Semin. Pediatr. Surg..

[7] Tayman C (2011). Mesenchymal stem cell therapy in necrotizing enterocolitis: A rat study. Pediatr. Res..

[8] McCulloh CJ (2018). Treatment of experimental necrotizing enterocolitis with stem cell-derived exosomes. J. Pediatr. Surg..

[9] Ikegame Y (2011). Comparison of mesenchymal stem cells from adipose tissue and bone marrow for ischemic stroke therapy. Cytotherapy.

[10] Katsuno T (2012). Low serum cultured adipose tissue-derived stromal cells ameliorate acute kidney injury in rats. Cell Transpl..

[11] Matsumoto T (2008). Mature adipocyte-derived dedifferentiated fat cells exhibit multilineage potential. J. Cell. Physiol..

[12] Yagi K, Kondo D, Okazaki Y, Kano K (2004). A novel preadipocyte cell line established from mouse adult mature adipocytes. Biochem. Biophys. Res. Commun..

[13] Oki Y, Watanabe S, Endo T, Kano K (2008). Mature adipocyte-derived dedifferentiated fat cells can trans-differentiate into osteoblasts in vitro and in vivo only by all-trans retinoic acid. Cell Struct. Funct..

[14] Kazama T, Fujie M, Endo T, Kano K (2008). Mature adipocyte-derived dedifferentiated fat cells can transdifferentiate into skeletal myocytes in vitro. Biochem. Biophys. Res. Commun..

[15] Sakuma T (2009). Mature, adipocyte derived, dedifferentiated fat cells can differentiate into smooth muscle-like cells and contribute to bladder tissue regeneration. J. Urol..

[16] Mikrogeorgiou A (2017). Dedifferentiated fat cells as a novel source for cell therapy to target neonatal hypoxic-ischemic encephalopathy. Dev. Neurosci..

[17] Sugiyama Y (2018). Intravenous administration of bone marrow-derived mesenchymal stem cell, but not adipose tissue-derived stem cell, ameliorated the neonatal hypoxic-ischemic brain injury by changing cerebral inflammatory state in rat. Front. Neurol..

[18] Kanehisa M, Goto S (2000). KEGG: Kyoto encyclopedia of genes and genomes. Nucleic Acid Res..

[19] Gao P (2022). High expression of PTGES3 is an independent predictive poor prognostic biomarker and correlates with immune infiltrates in lung adenocarcinoma. Int. Immunopharmacol..

[20] Wang S (2018). S100A8/A9 in Inflammation. Front Immunol..

[21] Kim HJ (2015). 15-hydroxyprostaglandin dehydrogenase is upregulated by hydroxychloroquine in rheumatoid arthritis fibroblast-like synoviocytes. Mol. Med. Rep..

[22] Chen IJ (2018). Targeting the 15-keto-PGE2-PTGR2 axis modulates systemic inflammation and survival in experimental sepsis. Free Radic. Biol. Med..

[23] Yin X (2022). ILF2 contributes to hyperproliferation of keratinocytes and skin inflammation in a KLHDC7B-DT-dependent manner in psoriasis. Front Genet..

[24] Watson SF, Bellora N, Macias S (2020). ILF3 contributes to the establishment of the antiviral type I interferon program. Nucleic Acids Res..

[25] Tobin DM, Roca FJ, Ray JP, Ko DC, Ramakrishnan L (2013). An enzyme that inactivates the inflammatory mediator leukotriene b4 restricts mycobacterial infection. PLoS ONE.

[26] Fussbroich D (2020). A combination of LCPUFAs regulates the expression of miRNA-146a-5p in a murine asthma model and human alveolar cells. Prostaglandins Other Lipid Mediat..

[27] Miyamoto J (2015). A gut microbial metabolite of linoleic acid, 10-hydroxy-cis-12-octadecenoic acid, ameliorates intestinal epithelial barrier impairment partially via GPR40-MEK-ERK pathway. J. Biol. Chem..

[28] Furusawa Y (2013). Commensal microbe-derived butyrate induces the differentiation of colonic regulatory T cells. Nature.

[29] Goto T (2015). 10-oxo-12(Z)-octadecenoic acid, a linoleic acid metabolite produced by gut lactic acid bacteria, potently activates PPARgamma and stimulates adipogenesis. Biochem. Biophys. Res. Commun..

[30] Simões AL (2016). Temporal profile of intestinal tissue expression of intestinal fatty acid-binding protein in a rat model of necrotizing enterocolitis. Clinics.

[31] Lu J, Jilling T, Li D, Caplan MS (2007). Polyunsaturated fatty acid supplementation alters proinflammatory gene expression and reduces the incidence of necrotizing enterocolitis in a neonatal rat model. Pediatr. Res..

[32] Terrin G, Stronati L, Cucchiara S, De Curtis M (2017). Serum markers of necrotizing enterocolitis: A systematic review. J. Pediatr. Gastroenterol. Nutr..

[33] Yang G, Wang Y, Jiang X (2016). Diagnostic value of intestinal fatty-acid-binding protein in necrotizing enterocolitis: A systematic review and meta-analysis. Indian J. Pediatr..

[34] Castell JV (1988). Recombinant human interleukin-6 (IL-6/BSF-2/HSF) regulates the synthesis of acute phase proteins in human hepatocytes. FEBS Lett..

[35] Xie Z (2017). MCP1 triggers monocyte dysfunctions during abnormal osteogenic differentiation of mesenchymal stem cells in ankylosing spondylitis. J. Mol. Med. (Berl).

[36] Arisaka M, Arisaka O, Yamashiro Y (1991). Fatty acid and prostaglandin metabolism in children with diabetes mellitus. II. The effect of evening primrose oil supplementation on serum fatty acid and plasma prostaglandin levels. Prostaglandins Leukot Essent Fatty Acids.

[37] D'Ippolito G, Schiller PC, Ricordi C, Roos BA, Howard GA (1999). Age-related osteogenic potential of mesenchymal stromal stem cells from human vertebral bone marrow. J. Bone Miner Res..

[38] Li X, Zhang Y (2017). iPSC-derived mesenchymal stem cells exert SCF-dependent recovery of cigarette-smoke-induced apoptosis/proliferation imbalance in airway cells. J. Cell Mol. Med..

[39] Lian Q (2016). Directed dhfferentiation of human-Induced pliripotent stem cells to mesenchymal stem cells. Methods Mol. Biol..

[40] Bloor AJC (2020). Production, safety and efficacy of iPSC-derived mesenchymal stromal cells in acute steroid-resistant graft versus host disease: A phase I, multicenter, open-label, dose-escalation study. Nat. Med..

[41] Jenkins MJ, Farid SS (2015). Human pluripotent stem cell-derived products: Advances towards robust, scalable and cost-effective manufacturing strategies. Biotechnol. J..

[42] Jumabay M (2009). Dedifferentiated fat cells convert to cardiomyocyte phenotype and repair infarcted cardiac tissue in rats. J. Mol. Cell Cardiol..

[43] Jiang YN (2020). Early protein markers of necrotizing enterocolitis in plasma of preterm pigs exposed to antibiotics. Front Immunol..

[44] Guven A (2009). Hyperbaric oxygen therapy reduces the severity of necrotizing enterocolitis in a neonatal rat model. J. Pediatr. Surg..

[45] Okuda S (2017). jPOSTrepo: An international standard data repository for proteomes. Nucleic Acids Res..

[46] da Huang W, Sherman BT, Lempicki RA (2009). Systematic and integrative analysis of large gene lists using DAVID bioinformatics resources. Nat. Protoc..

