# Supplementary material for: Dedifferentiated fat cells administration ameliorates abnormal expressions of fatty acids metabolism-related protein expressions and intestinal tissue damage in experimental necrotizing enterocolitis
Source: Sci Rep. 2023 May 22;13:8266. doi: 10.1038/s41598-023-34156-1 (PMC10203254; doi:10.1038/s41598-023-34156-1)

a

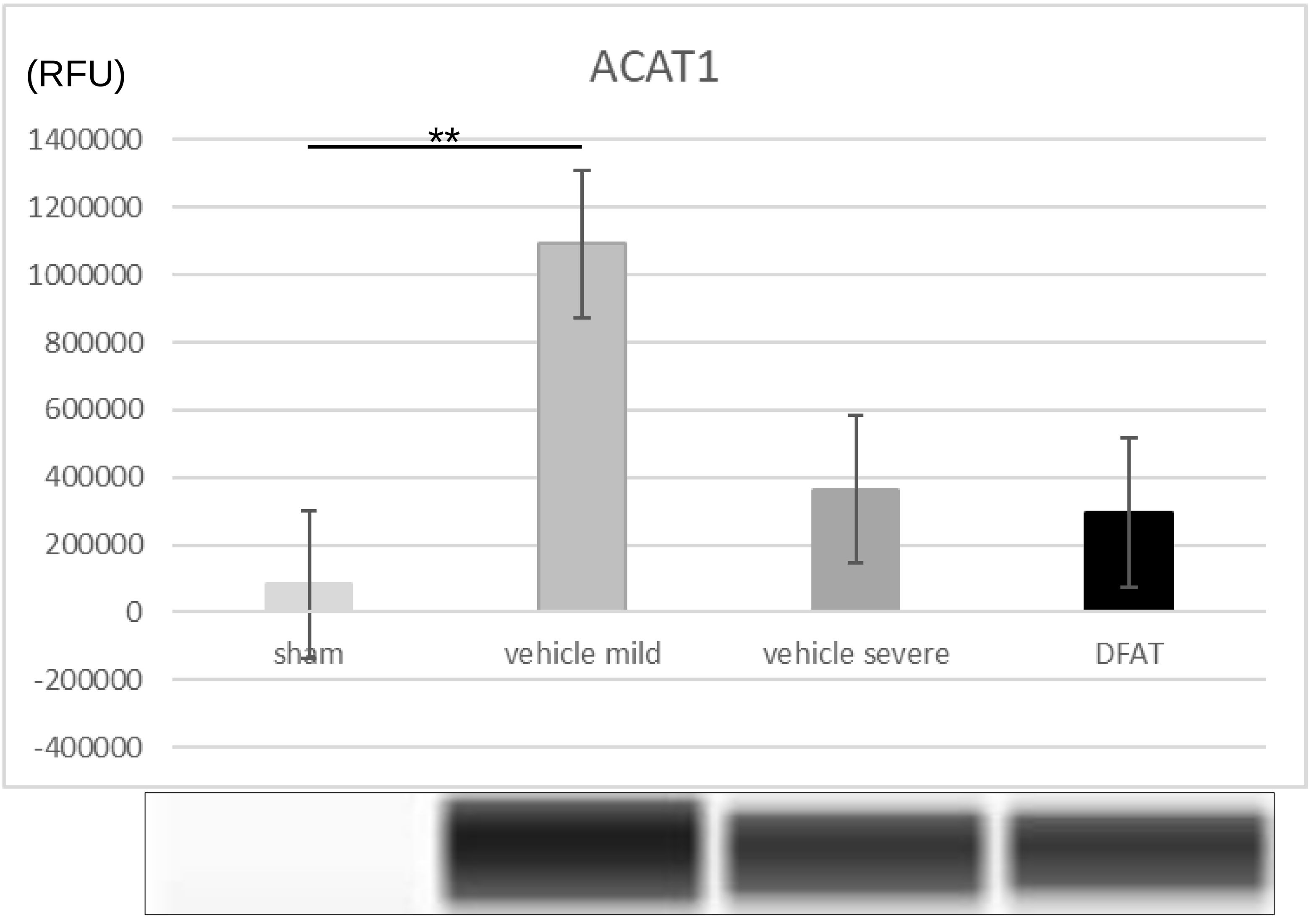

b

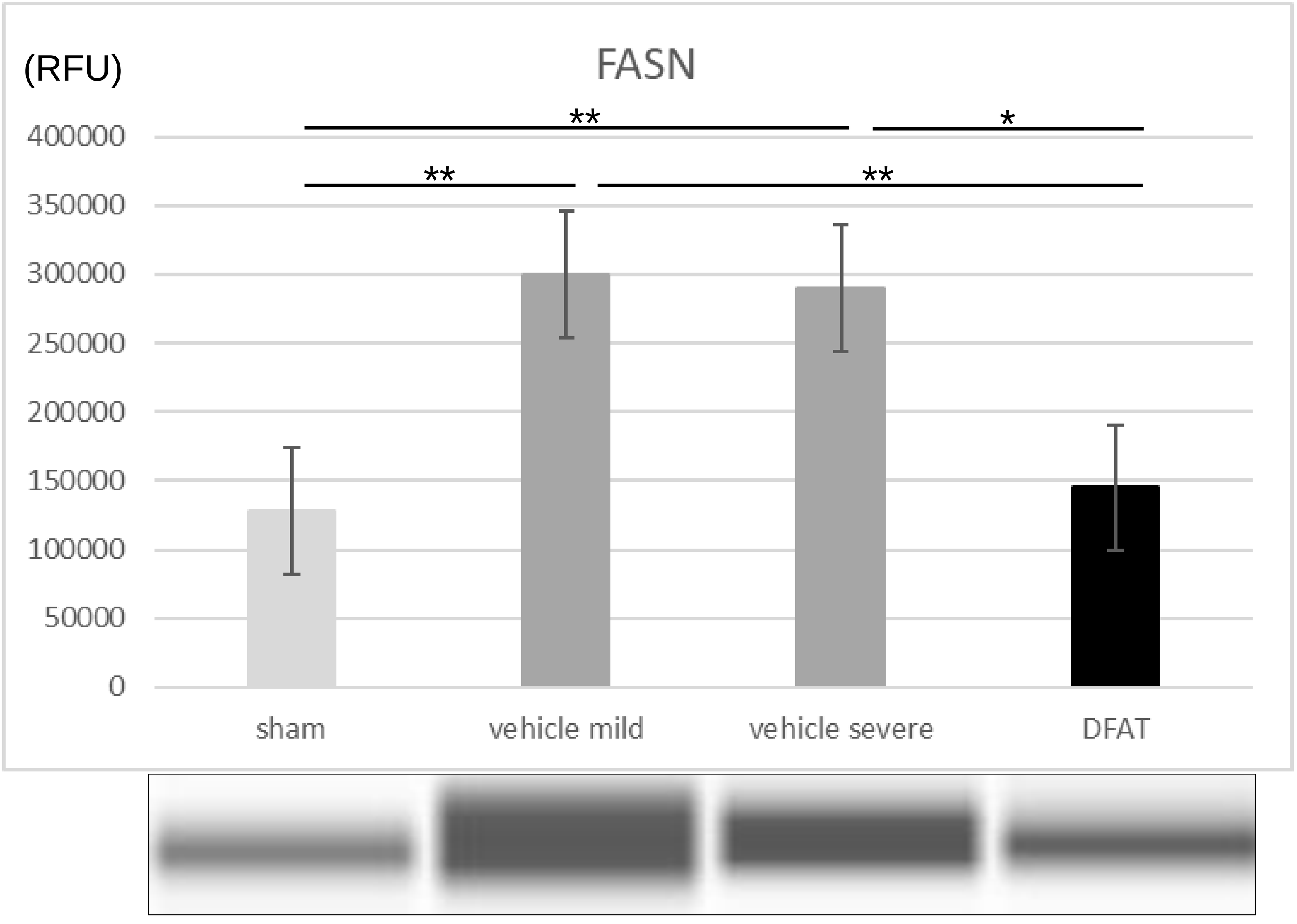

c

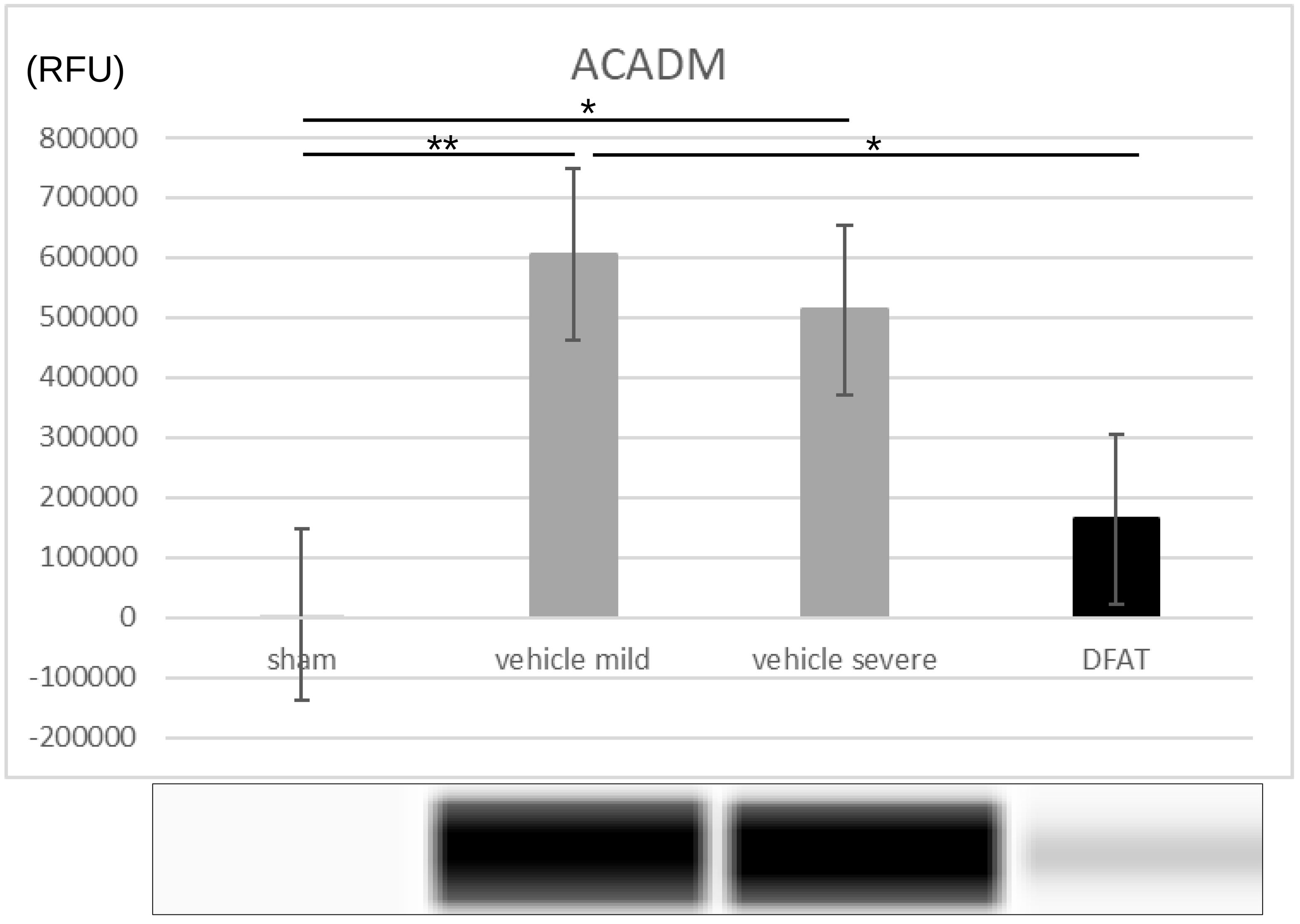

Supplemental figure

Protein expression was evaluated by electrophoresis immunoassay (JESS) (a) Acat1 (b) Fasn (c) Acadm

The expression of each protein was normalized with total protein abundance in the same capillary.

*P < 0.05, **P < 0.01; n = 3 for each group

RFU: relative fluorescent unit

Full images of electrophoresis immunoassay (JESS)

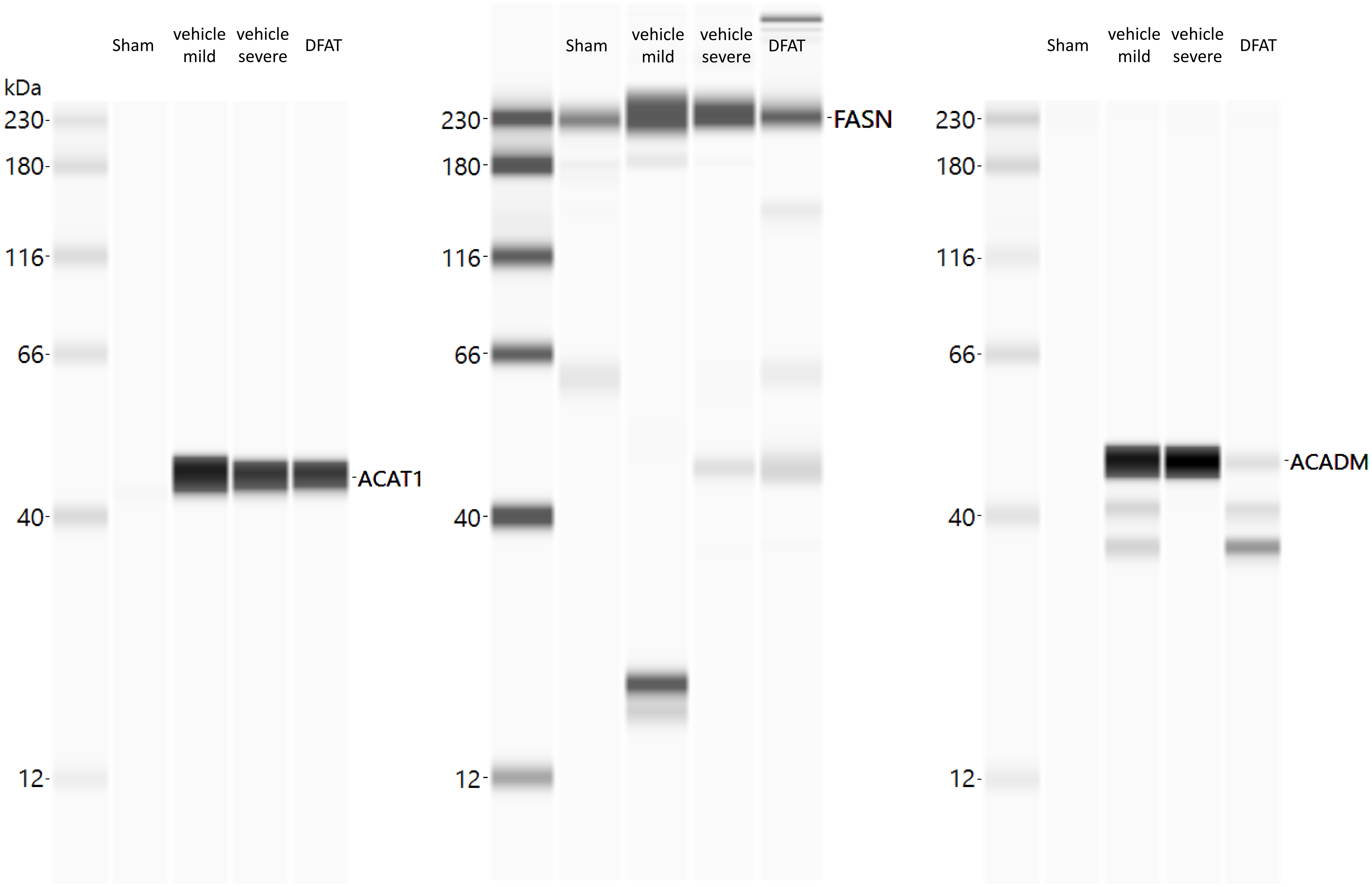

Supplement: Supplementary file 1 — Supplementary Information 1. [file 41598_2023_34156_MOESM1_ESM.pdf]
