# Supplementary material for: Dedifferentiated fat cells administration ameliorates abnormal expressions of fatty acids metabolism-related protein expressions and intestinal tissue damage in experimental necrotizing enterocolitis
Source: Sci Rep. 2023 May 22;13:8266. doi: 10.1038/s41598-023-34156-1 (PMC10203254; doi:10.1038/s41598-023-34156-1)
Supplement: Supplementary file 2 — Supplementary Information 2. [file 41598_2023_34156_MOESM2_ESM.docx]

**Supplemental Materials and Methods**

**DFAT cell preparation**

Green fluorescent protein (GFP)-labeled DFATs[*40*] were prepared from subcutaneous adipose tissue in GFP transgenic rats using a previously described ceiling culture method[*12*] (Sprague-Dawley YgN [act-EGFP] OsbCZ-004). Cells were cultured in Dulbecco's modified Eagle's medium (Invitrogen, Carlsbad, CA, USA), which was supplemented with 20% (v/v) fetal bovine serum (SAFC Biosciences, lot 6G2146, Lenexa, KS, USA) in the dedifferentiation phase and 10% (v/v) fetal bovine serum (USDA-tested fetal bovine serum; Thermo Fisher Scientific Inc., lot AWD12936, Waltham, MA, USA) in the proliferation phase, where cells were incubated at 37°C with 5% CO_2_. The medium was changed every 3 to 4 days．

**Animals and experimental design**

NEC was induced by modifying a well-established model described by Guven et al. [*44*]. In this model, pups were subjected to major risk factors for human NEC, prematurity, formula feeding, bacterial colonization, and hypoxia–ischemia, which lead to ileum and proximal colon intestinal injury. Neonatal rats were delivered from pregnant Sprague-Dawley dams *via* cesarean section on embryonic day 21. They were then housed in an incubator (temperature: 34°C, humidity: 70%–90%). Rat pups were hand fed three times daily with 0.2 mL high osmotic condition milk, Pets Own Milk (Doggy Man, H.A. Co., Ltd., Osaka, Japan), mixed 15% Meiji LW (Meiji Co., Ltd., Tokyo, Japan), using a flexible plastic feeding tube (22 gauge, Instech Laboratories Inc., Montgomery, PA, USA). The first feeding was started 2 h after delivery. All rat pups were also subjected to asphyxia (100% CO_2_ gas for 10 min) and cold stress (4°C for 5 min), followed by recovery (100% oxygen gas for 5 min) twice daily. Furthermore, lipopolysaccharides from *Escherichia coli* 0111:B4 (Sigma-Aldrich, St. Louis, MO, USA), 3 mg/kg, were administered by feeding at 2 and 38 h after birth.

The rat pups were allocated to three groups as follows: NEC + Ringer's solution (AY pharma, Tokyo, Japan) 50 µL intraperitoneal injection (IP) (vehicle), NEC + DFAT, 1.0 × 10^6^ cells in 50 µL Ringer's solution) IP at 32 and 52 h after birth (DFAT); natural vaginal delivered rat pups were fed mother's milk as a control (sham).

The pups were weighed daily and recorded alive or dead. The pups were sacrificed 96 h after birth.

**Tissue preparation**

All pups were euthanized with a pentobarbital sodium overdose. The abdominal cavity was opened, and the small intestine was visually evaluated for typical signs of NEC (macroscopic evaluation). Then 2-cm sections of the end of the ileum were collected and fixed overnight in 4% paraformaldehyde at room temperature (RT; about 25–28°C). After graded dehydration with ethanol and xylene, tissues were embedded in paraffin and cut into 5-µm sections for histological evaluation. Tissues were fixed with PFA overnight for immunohistostaining with GFP antibody, followed by 20% sucrose for 24 h and 30% sucrose for 24 h. These fixed tissues were embedded in OTC compound to make 8-µm frozen sections.

**Histological and immunohistochemical procedures**

We performed a tissue structure evaluation with hematoxylin and eosin (H&E) staining and apoptosis evaluation with active caspase-3 antibody and TUNEL using paraffin sections.

For H&E evaluation, the sections were deparaffinized and rehydrated with xylene and graded alcohols to water. The sections were stained with hematoxylin (Mayer's hematoxylin; Wako, Osaka, Japan) and eosin (Wako), dehydrated with ethanol and xylene, and mounted.

For evaluation of apoptosis using an active caspase-3 antibody, the sections were deparaffinized and rehydrated in the same way for H&E staining. Antigen retrieval was performed by incubation with 0.01 M citric acid monohydrate (Fujifilm Wako Pure Chemical Corporation, Osaka, Japan) in distilled water (pH 6) for 10 min at 90°C with heating. After incubation with blocking solution, 4% normal donkey serum (Jackson Immuno Research, Baltimore, PA, USA) with 10% Triton-100 (MP Biomedicals, Inc., Sanata Ana, CA, USA) in phosphate-buffered saline (PBS; Takara Bio Inc., Shiga, Japan) for 30 min, the sections were incubated with the primary antibody, Purified Rabbit Anti Active Caspase-3 (dilution 1:200; BD Biosciences, Franklin Lakes, NJ, USA) in blocking solution at 4°C overnight．On the second day, the sections were incubated with secondary antibody, Biotin-SP-Conjugated AffiniPure donkey Anti Rabbit IgG (dilution 1:400; Jackson Immuno Research) in PBS at RT for 60 min. Then, the sections were incubated with 3% H_2_O_2_ (Takara Bio Inc.) in PBS for 10 min to quench endogenous peroxidase activity. The binding was visualized with the Vectastain Elite ABC Standard Kit (Vector Laboratories, Burlingame, CA, USA), followed by peroxidase detection for 10 min (0.12 mg/mL 3,3′-diaminobenzidine, 0.01% H_2_O_2_, and 0.04% NiCl_2_). The sections were dehydrated with ethanol and xylene and then mounted with NEW M・X (Misumi Corporation, Tokyo, Japan).

For evaluation of engraftment of DFAT, frozen sections (8 µm) were used. The sections were heated in boiling 0.01 M citric acid (pH 6.0) for 10 min and then blocked with 4% normal donkey serum (with 0.1% Triton-X-100) in PBS for 60 min. Tissue sections were incubated with a monoclonal antibody Rb anti GFP (1:200, Medical & Biological Laboratories, Tokyo,Japan) in 4% normal donkey serum with 0.1% Triton-X100 overnight at 4°C, followed by incubation with donkey anti Rb IgG-555 (1:500) in 4% normal donkey serum (with 0.1% Triton-100) in PBS for 1 h at RT in the dark. Then, sections were mounted using Prolong Gold with DAPI (ProLong Gold antifade reagent with DAPI by Life Technologies).

**TUNEL staining**

Sections were deparaffinized and rehydrated with graded alcohols to water to evaluate apoptosis. The sections were incubated with 20 µg/mL of proteinase K (Roche, Mannheim, Germany) for 30 min at RT. Then, they were incubated with 3% H_2_O_2_ in PBS for 5　min. The In Situ Cell Death Detection Kit, POD (Roche) was used, and the reaction solution, which was prepared by mixing the enzyme and label solutions, was added to the sample at RT in a humidified chamber and allowed to stand for 60 min. They were then incubated with Converter-POD for 30 min in a humidity box. The slides were stained with 3,3′-Diaminobenzidine solution (0.12mg/mL 3,3′-diaminobenzidine, 0.01% H_2_O_2_, and 0.04% NiCl_2_), then dehydrated with ethanol, and mounted with NEW M・X.

**Macroscopic and histological evaluation**
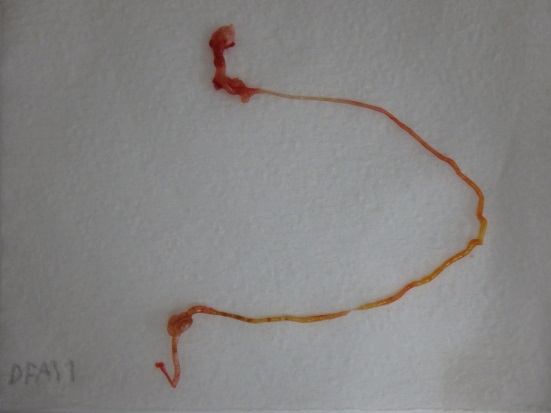


The macroscopic evaluation was performed on the open rat abdominal cavity. Typical signs of NEC were evaluated using a scoring system reported by Yan-Nan Jiang et al.[*43*] with minor modification as follows: 0, indicating normal intestine (absence of macroscopic hemorrhage, edema, or mucosal abnormality); 1, indicating local hyperemia and hyperemia, extensive edema, and local hemorrhage; 2, indicating extensive hemorrhage and local necrosis and pneumatosis intestinalis; and 3, indicating extensive transmural necrosis and pneumatosis intestinalis. The intestinal color that changed from yellow to dark brown was allocated a score of 2 (Figures 2A–D).

Tissue structure evaluation was performed with H&E staining. We modified the scoring of Guven *et al.*[*44*] as follows: 0 (normal), no damage; 1 (mild), separation of the villous core with no other abnormalities; 2 (moderate) villous core separation, submucosal edema, and epithelium sloughing; and 3 (severe), denudation of the epithelium with loss of villous, full-thickness necrosis, or perforation (Figures 2E–H).

**Apoptosis evaluation**

We evaluated apoptosis of the intestine using immunohistochemical staining with active caspase-3 antibody and TUNEL staining with the grading system. Tissues were graded 0 (normal, no apoptotic change), 1 (mild, apoptotic nuclei present at villous tips), 2 (moderate, apoptotic nuclei covering all villous tips but crypts protected), and 3 (severe, the transmural spread of apoptotic nuclei) (Figures 2I–L)[*44*].

**Proteomics preparation**

We obtained the end of ileum tissue 0.05 g (snap frozen in liquid N_2_) from the NEC model, NEC model with DFATs, and sham using the Multi-beads shocker® (cell disruptor: Yasui Kikai Corporation, Osaka, Japan) with liquid nitrogen. We separated these into four groups: sham, vehicle mild (scores 0 and 1 in the macroscopic evaluation), vehicle severe (scores 2 and 3 in the macroscopic evaluation), and DFAT groups. These samples were put in detergent-free lysis buffer (Minute TM Detergent-Free Protein Extraction Kit for Animal Cultured Cells and Tissues: Funakoshi Co., Ltd., Tokyo, Japan) with protease inhibitor (Complete Mini-EDTA-free: Sigma-aldrich, MO, USA) and mixed. The homogenates were centrifuged at 21130 rcf for 1 min to remove debris, and then, the supernatants were collected as protein lysates. The total protein concentration of the lysates was quantified using the bicinchoninic acid method using the Pierce BCA Protein Assay Kit (Thermo Fisher Scientific Inc.). After protein quantification, lysates of three animals were pooled within each group and adjusted to 100 µg/200 µL for liquid chromatography/tandem mass spectrometry (LC/MS/MS) and stored at -80°C in a deep freezer until use.

**Proteomics**

The concentration of all types of proteins in the ileum of each group was comprehensively quantified *via* LC/MS/MS. For LC/MS/MS, we used an Orbitrap Fusion mass spectrometry system (Thermo Fisher Scientific) combined with UltiMate3000 RSLCnano LC system (Dionex Co., Amsterdam, the Netherlands) with a nano HPLC capillary column (150 mm × 75 μm i.d., Nikkyo Technos Co., Tokyo, Japan) *via* a nanoelectrospray ion source. In reversed-phase chromatography, a linear gradient flow rate (0 min, 5% B; 100 min, 40% B) of solvent A (2% acetonitrile with 0.1% formic acid) and solvent B (95% acetonitrile with 0.1% formic acid) was set at 300 nL/min. Before MS/MS analysis, a precursor ion scan was performed using a 400–1600 mass-to-charge ratio (m/z). MS/MS was performed *via* quadrupole isolation at 0.8 Th, HCD fragmentation at 30% normalized collision energy, and rapid scan MS analysis in an ion trap. Only precursors with charge states 2–6 were sampled for MS2. The dynamic exclusion time was set to 15 s with a tolerance of 10 ppm. The instrument was run in maximum speed mode with a 3-second cycle. After quantification, the proteome software Scaffold (version Scaffold_4.4.8, Proteome Software Inc., Portland, OR, USA) was used to validate MS/MS-based peptide and protein identifications. The proteome data were analyzed using Proteome Discoverer 1.4 (Thermo Fisher Scientific) and the MASCOT search engine (version 2.6.0, Matrix Science Inc., Boston, MA, USA) to identify the proteins and peptides. For identification, we referred to the protein database in UniProt (release 2019_06) and set a precursor mass tolerance and a fragment ion mass tolerance as 10 ppm and 0.8 Da, respectively. The primary proteome data (Table S1) were submitted to the Japan Proteome Standard Repository/Database [*45*]. The proteomics data accession number for data in the present study is jPOST: JPST001143 (PXD025647).

**Protein extraction and functional analysis**

From the proteome data, proteins dysregulated by NEC were extracted by threshold levels as follows: protein severity-dependently up-regulated by NEC (mild/sham > 1.5-fold, severe/sham > 2.0-fold, and severe/mild ratio > 2.0/1.5) and protein severity-dependently down-regulated by NEC (mild/sham ratio < 1/1.5, severe/sham ratio < 1/2.0, and severe/mild < 1.5/2.0). Furthermore, we analyzed the dysregulated proteins to identify the proteins ameliorated by DFAT cell injection as follows: proteins in which up-regulation by NEC was ameliorated (DFAT/severe ratio < 1/2 and DFAT/mild ratio < 1/1.5) and proteins in which down-regulation by NEC was ameliorated (DFAT/severe ratio > 2.0 and DFAT/mild ratio > 1.5). To understand the biological significance of the proteome results, the extracted protein lists (dysregulated proteins by NEC and ameliorated proteins by DFAT cells) were processed for functional analysis using the KEGG pathway database. Proteins were annotated with the KEGG pathway database for annotation, visualization, and integrated discovery version 6.8 (DAVID 6.8) bioinformatics resources (<https://david.ncifcrf.gov/home.jsp>; 2019_06)[*46*]. We used the annotation database on June 14, 2019. The flagged KEGG pathway term was processed with a functional annotation clustering on the DAVID to remove redundant annotation terms. Finally, we extracted the KEGG pathway terms with a P-value <0.05, fold enrichment >2, symbol number >3, and cluster enrichment score >2 as significant terms.

**Capillary electrophoresis immunoassay**

Protein expression was evaluated by electrophoresis immunoassay (JESS) using the same proteins found by proteomics with functional analysis. The immunoassay was performed on the JESS system (ProteinSimple, San Jose, CA) using a 12–230 kDa Separation Module (SM-FL004, ProteinSimple). The proteins were identified by specific antibodies in the capillary system, and the system measured and captured their chemiluminescence reactions as digital blot images. The specific antibodies used for the capillary electrophoresis immunoassay were rabbit anti- Aacat1 (dilution 1:50; Proteintech, Rosemont, IL, USA), rabbit anti- Fasn (dilution 1:10, Proteintech), and rabbit anti- Acadm (dilution 1:10; Proteintech). Capillary images were analyzed by the manufacturer Compass software (ProteinSimple). The expression of each protein was normalized with total protein abundance in the same capillary.

**Real-time polymerase chain reaction**

Total RNA was extracted from the end of the ileum tissue 0.05 g (snap frozen in liquid N_2_) after homogenization using the Multi-beads shocker® (cell disruptor: Yasui Kikai Corporation) and TRI Reagent® (Cosmo Bio Co., Ltd., Tokyo, Japan) with the RNeasy Mini Kit (Qiagen Inc., PL, Venlo, the Netherlands) according to the manufacturer's protocol. The RNA concentration was quantified using ultraviolet spectrophotometry at A260, and the purity was determined by the 260/A280 ratio using NanoDrop^TM^ One/One^C^ (Thermo Fisher Scientific). The RNA integrity number (RIN) was measured with Agilent 2100 bioanalyzer (Agilent Technologies, Santa Clara, CA, USA) and selected the samples above RIN 6. Complementary DNA (cDNA) was synthesized from 0.5 µg total RNA using the SuperScript VILO cDNA Synthesis Kit (Thermo Fisher Scientific). After that, quantitative real-time polymerase chain reaction was performed using Brilliant III Ultra-Fast SYBR Green QPCR Master Mix (Agilent Technologies) on an Mx3005P RT QPCR System (Agilent Technologies). Amplification was initiated at 95°C for 3 min, followed by 40 cycles of 95°C for 10 s and 60°C for 22 s. Correlation coefficients were above 0.94 for each standard curve. All measurements were performed in triplicate. The primer sequences were as follows;

IL-6 forward, ATATGTTCTCAGGGAGATCTTGGA;

reverse, TGCATCATCGCTGTTCATACAA;

CCL2 forward, CTATGCAGGTCTCTGTCACGCTTC;

reverse, CAGCCGACTCATTGGGATCA;

IL-1β forward, TCTGATGTTCCCATTAGAC;

reverse, AATACCACTTGTTGGCTTA.

TNFα forward, GAAATCCAGGAATTGATGGCTGA;

reverse, GTTGCACACAGGCTGGTAGGAG.

β2-microglobulin (housekeeping gene)

forward, CCTGGCTCACACACTGAATTCACAC;

reverse, AACCGGATCTGGAGTTAAACTGGT.

**Statistical analysis**

Statistical analyses were conducted using IBM Statistical Package for the Social Sciences version 24 (IBM Japan, Tokyo, Japan) or Statcel 4 (Seiun-sha, Shiga, Tokyo, Japan). Survival was estimated with the Kaplan–Meier method and compared using the log-rank test. The Kruskal-Wallis analysis, followed by the Steel-Dwass test, was used when three groups were compared for body weight, macroscopic, histological evaluation, and real-time PCR. One-way analysis of variance, followed by Holm-Šídák’s multiple comparisons test, was used to assess protein expression in electrophoresis immunoassay. The significance threshold was *P*-values of <0.05.
