# Supplementary material for: Dedifferentiated fat cells administration ameliorates abnormal expressions of fatty acids metabolism-related protein expressions and intestinal tissue damage in experimental necrotizing enterocolitis
Source: Sci Rep. 2023 May 22;13:8266. doi: 10.1038/s41598-023-34156-1 (PMC10203254; doi:10.1038/s41598-023-34156-1)
Supplement: Supplementary file 3 — Supplementary Table S1. [file 41598_2023_34156_MOESM3_ESM.pdf]

Table S1. Raw data

Accession	Description	MW [kDa]	Area				Score				Coverage				# Peptides				# PSM			
			sham	vehicle-mild	vehicle-severe	DFAT	sham	vehicle-mild	vehicle-severe	DFAT	sham	vehicle-mild	vehicle-severe	DFAT	sham	vehicle-mild	vehicle-severe	DFAT	sham	vehicle-mild	vehicle-severe	DFAT
A0A0G2K0D3	Leiomodin-1 OS=Rattus norvegicus OX=10116 GN=Lmod1 PE=1 SV=1 - [LMOD1_RAT]	66.2	4.457E-5	9.169E-5	7.381E-5	7.297E-5	27.08	138.95	133.72	127.38	1.85	7.23	7.23	7.39	1	4	4	4	1	7	6	6
A0A0G2K2P5	Tight junction protein ZO-1 OS=Rattus norvegicus OX=10116 GN=Tjp1 PE=1 SV=1 - [ZO1_RAT]	197.0	6.283E-5	1.352E-4	1.244E-4	1.163E-4	162.07	374.80	364.12	230.57	7.42	7.20	8.33	6.91	8	8	10	9	11	16	19	17
A0JPJ7	Obg-like ATPase 1 OS=Rattus norvegicus GN=Ola1 PE=2 SV=1 - [OLA1_RAT]	44.5	0.000E0	0.000E0	2.006E-4	8.653E-5			314.45	65.09			25.76	17.42			8	5			15	9
A0JPN2	Zinc transporter ZIP4 OS=Rattus norvegicus OX=10116 GN=Slc39a4 PE=2 SV=1 - [S39A4_RAT]	71.1	0.000E0	1.052E-5	0.000E0	0.000E0		37.52				1.98				1				1		
A2RUV9	Adipocyte enhancer-binding protein 1 OS=Rattus norvegicus GN=Aebp1 PE=2 SV=1 - [AEBP1_RAT]	128.0	4.373E-5	0.000E0	0.000E0	0.000E0	53.72				1.24				1				2			
A2RUW1	Toll-interacting protein OS=Rattus norvegicus GN=Tollip PE=2 SV=1 - [TOLIP_RAT]	30.3	0.000E0	5.598E-5	1.039E-4	3.348E-5		99.97	57.12	28.77		8.03	4.74	13.14		2	1	3		3	2	4
A2VCX1	TIP41-like protein OS=Rattus norvegicus GN=Tiprl PE=1 SV=1 - [TIPRL_RAT]	31.2	0.000E0	3.708E-5	0.000E0	0.000E0		47.65				9.96				2				2		
A7VJC2	Heterogeneous nuclear ribonucleoproteins A2/B1 OS=Rattus norvegicus GN=Hnrnpa2b1 PE=1 SV=1 - [ROA2_RAT]	37.5	2.617E-3	6.202E-3	4.467E-3	3.986E-3	1319.48	2655.34	2270.84	1912.82	33.99	44.76	44.48	51.27	16	20	18	20	51	87	75	74
B0BN18	Prefoldin subunit 2 OS=Rattus norvegicus GN=Pfdn2 PE=2 SV=1 - [PFD2_RAT]	16.6	0.000E0	8.998E-5	1.058E-4	6.389E-5		187.06	184.56	146.30		16.88	16.88	16.88		2	2	2		4	4	3
B0BN85	Protein SGT1 homolog OS=Rattus norvegicus GN=Sugt1 PE=2 SV=1 - [SGT1_RAT]	38.1	1.355E-4	6.171E-5	5.306E-5	0.000E0	60.25	59.94	31.90		10.12	11.31	9.82		3	3	2		7	4	3	
B0BN93	26S proteasome non-ATPase regulatory subunit 13 OS=Rattus norvegicus GN=Psmd13 PE=1 SV=1 - [PSD13_RAT]	42.8	4.614E-5	1.291E-4	2.002E-4	1.710E-4	84.87	157.69	688.14	281.06	11.44	25.53	60.11	46.28	4	10	19	15	5	15	34	24
B0BN94	Protein FAM136A OS=Rattus norvegicus GN=Fam136a PE=1 SV=1 - [F136A_RAT]	15.6	8.928E-5	6.596E-5	8.595E-5	0.000E0	15.07	127.09	126.68		13.04	36.23	13.04		1	3	1		1	5	2	
B0BNA5	Coactosin-like protein OS=Rattus norvegicus GN=Cotl1 PE=1 SV=1 - [COTL1_RAT]	15.9	7.026E-4	1.918E-3	1.903E-3	1.449E-3	595.38	1209.13	1126.75	1020.16	57.04	66.90	66.90	66.90	7	9	10	9	20	35	36	30
B0BNA7	Eukaryotic translation initiation factor 3 subunit I OS=Rattus norvegicus GN=Eif3i PE=2 SV=1 - [EIF3I_RAT]	36.4	3.323E-5	1.019E-4	1.535E-4	6.180E-5	29.94	195.07	214.23	103.70	5.54	27.38	21.54	8.31	2	6	5	3	2	8	8	5
B0BNE2	DNA-directed RNA polymerases I, II, and III subunit RPABC1 OS=Rattus norvegicus GN=Polr2e PE=2 SV=1 - [RPAB1_RAT	24.6	0.000E0	4.034E-5	0.000E0	0.000E0		30.65				14.29				2				2		
B0BNE5	S-formylglutathione hydrolase OS=Rattus norvegicus GN=Esd PE=1 SV=1 - [ESTD_RAT]	31.3	0.000E0	5.055E-4	6.039E-4	3.022E-4		238.09	439.05	295.68		47.16	51.77	45.04		8	10	9		16	25	18
B0BNM1	NAD(P)H-hydrate epimerase OS=Rattus norvegicus GN=Apoa1bp PE=2 SV=1 - [NNRE_RAT]	30.9	0.000E0	4.720E-5	6.275E-5	8.469E-5		37.40	73.88	45.48		20.57	14.54	17.38		3	2	3		4	4	4
B0BNN3	Carbonic anhydrase 1 OS=Rattus norvegicus GN=Ca1 PE=1 SV=1 - [CAH1_RAT]	28.3	2.453E-4	2.642E-4	1.844E-4	2.687E-4	302.13	308.01	152.57	282.52	57.47	52.11	40.61	48.28	10	9	6	8	16	19	8	15
B0K019	BAG family molecular chaperone regulator 1 OS=Rattus norvegicus OX=10116 GN=Bag1 PE=2 SV=1 - [BAG1_RAT]	40.1	4.669E-5	4.044E-5	4.031E-5	0.000E0	49.05	72.57	72.72		8.66	6.70	8.66		2	2	2		2	3	3	
B0K020	CDGSH iron-sulfur domain-containing protein 1 OS=Rattus norvegicus OX=10116 GN=Cisd1 PE=3 SV=1 - [CISD1_RAT]	12.1	6.525E-5	0.000E0	0.000E0	0.000E0	38.52				12.04				1				1			
B0LT89	Serine/threonine-protein kinase 24 OS=Rattus norvegicus GN=Stk24 PE=2 SV=1 - [STK24_RAT]	48.0	0.000E0	0.000E0	0.000E0	2.776E-5				21.30				1.62				1				2
B1H267	Sorting nexin-5 OS=Rattus norvegicus GN=Snx5 PE=1 SV=1 - [SNX5_RAT]	46.8	2.114E-4	9.611E-5	5.094E-5	0.000E0	258.29	57.20	36.00		11.14	3.71	3.71		4	1	1		11	2	1	
B1H268	Mini-chromosome maintenance complex-binding protein OS=Rattus norvegicus GN=Mcmbp PE=2 SV=1 - [MCMBP_RAT]	73.0	0.000E0	1.600E-5	0.000E0	1.958E-5		34.99		21.31		2.02		2.02		1		1		1		1
B2GUV7	Eukaryotic translation initiation factor 5B OS=Rattus norvegicus GN=Eif5b PE=1 SV=1 - [IF2P_RAT]	137.6	0.000E0	6.109E-5	7.455E-5	5.471E-5		89.04	125.69	58.40		7.24	8.96	3.37		5	7	2		7	8	2
B2GUZ5	F-actin-capping protein subunit alpha-1 OS=Rattus norvegicus GN=Capza1 PE=1 SV=1 - [CAZA1_RAT]	32.9	1.491E-5	4.810E-4	7.117E-4	3.925E-4	27.30	571.07	625.85	389.43	2.80	64.69	64.69	54.20	1	12	12	11	1	24	27	18
B2GV06	Succinyl-CoA:3-ketoacid coenzyme A transferase 1, mitochondrial OS=Rattus norvegicus GN=Oxct1 PE=1 SV=1 - [SCOT1_	56.2	0.000E0	8.479E-5	1.387E-4	7.874E-5		118.06	380.67	105.86		26.15	31.54	13.27		7	9	4		8	16	6
B2RYD2	Epithelial splicing regulatory protein 1 OS=Rattus norvegicus OX=10116 GN=Esrp1 PE=2 SV=2 - [ESRP1_RAT]	75.0	0.000E0	3.080E-5	0.000E0	6.651E-5		26.20		33.62		2.51		2.51		2		2		3		3
B2RYG6	Ubiquitin thioesterase OTUB1 OS=Rattus norvegicus GN=Otub1 PE=1 SV=1 - [OTUB1_RAT]	31.3	0.000E0	2.145E-4	2.848E-4	2.242E-4		369.86	512.18	459.93		35.06	38.01	31.37		8	9	8		18	18	18
B2RYM5	Lys-63-specific deubiquitinase BRCC36 OS=Rattus norvegicus GN=Brcc3 PE=2 SV=1 - [BRCC3_RAT]	33.0	0.000E0	2.540E-4	1.956E-4	5.673E-4		21.75	28.80	26.92		3.44	8.59	3.44		1	2	1		1	3	2
B2RYW9	Fumarylacetoacetate hydrolase domain-containing protein 2 OS=Rattus norvegicus GN=Fahd2 PE=1 SV=1 - [FAHD2_RAT	34.6	0.000E0	4.520E-5	4.193E-5	0.000E0		51.15	120.34			13.10	15.65			3	4			5	6	
B2RZ78	Vacuolar protein sorting-associated protein 29 OS=Rattus norvegicus GN=Vps29 PE=1 SV=2 - [VPS29_RAT]	20.5	2.061E-5	2.372E-4	1.885E-4	2.380E-4	36.71	202.18	95.58	148.91	12.64	39.56	32.42	39.56	1	5	4	5	2	9	8	10
B3GNI6	Septin-11 OS=Rattus norvegicus GN=Sept11 PE=1 SV=1 - [SEP11_RAT]	49.7	1.981E-4	1.028E-4	1.588E-4	7.799E-5	292.00	131.24	245.99	184.38	13.46	5.80	16.24	5.80	6	3	6	3	11	9	15	8
B5DF91	ELAV-like protein 1 OS=Rattus norvegicus OX=10116 GN=Elavl1 PE=1 SV=1 - [ELAV1_RAT]	36.1	8.084E-4	9.596E-4	6.948E-4	5.641E-4	514.36	606.95	466.76	527.44	37.12	39.26	30.98	36.50	10	10	8	9	23	24	19	19
B5DFC8	Eukaryotic translation initiation factor 3 subunit C OS=Rattus norvegicus GN=Eif3c PE=1 SV=1 - [EIF3C_RAT]	105.4	0.000E0	1.237E-4	1.725E-4	1.667E-4		41.34	432.81	250.78		8.23	13.61	9.77		6	11	8		10	19	13
B5DFC9	Nidogen-2 OS=Rattus norvegicus GN=Nid2 PE=2 SV=1 - [NID2_RAT]	152.9	7.257E-5	2.870E-5	3.812E-5	1.788E-5	116.91	35.64	54.84	22.93	3.37	2.15	1.93	0.93	4	3	2	1	6	3	2	1
B5DFN2	Adenosylhomocysteinase 2 OS=Rattus norvegicus GN=Ahcyl1 PE=1 SV=2 - [SAHH2_RAT]	53.7	2.364E-5	1.289E-2	1.147E-2	1.684E-2	91.17	414.64	426.02	343.48	1.66	15.53	26.71	21.33	1	7	10	8	2	17	22	17
D3ZAF6	ATP synthase subunit f, mitochondrial OS=Rattus norvegicus OX=10116 GN=Atp5mf PE=1 SV=1 - [ATPK_RAT]	10.4	0.000E0	7.482E-5	0.000E0	9.129E-5		28.55		67.12		13.64		23.86		1		2		1		4
D3ZBN0	Histone H1.5 OS=Rattus norvegicus OX=10116 GN=Hist1h1b PE=1 SV=1 - [H15_RAT]	22.6	0.000E0	1.279E-4	1.004E-4	5.539E-4		201.14	136.56	338.00		18.47	18.47	26.13		4	4	6		7	7	14
D3ZBP4	[F-actin]-monooxygenase MICAL1 OS=Rattus norvegicus OX=10116 GN=Mical1 PE=1 SV=1 - [MICA1_RAT]	116.6	0.000E0	1.190E-4	1.213E-4	1.779E-4		24.44	25.65	25.48		1.34	1.34	0.57		2	2	1		4	3	4
D3ZD32	Chromodomain-helicase-DNA-binding protein 5 OS=Rattus norvegicus GN=Chd5 PE=1 SV=1 - [CHD5_RAT]	222.1	0.000E0	4.689E-5	7.336E-5	4.769E-5		51.21	80.16	50.59		1.59	2.05	2.52		3	3	4		3	3	4
D3ZDK7	Glycerol-3-phosphate phosphatase OS=Rattus norvegicus OX=10116 GN=Pgp PE=1 SV=1 - [PGP_RAT]	34.6	0.000E0	3.374E-5	5.336E-5	3.523E-5		43.33	155.01	25.24		9.03	16.82	11.84		2	4	3		3	6	3
D3ZHA0	Filamin-C OS=Rattus norvegicus GN=Flnc PE=1 SV=1 - [FLNC_RAT]	290.8	7.717E-4	1.179E-3	1.187E-3	7.193E-4	3575.03	4469.85	3730.28	2268.36	32.91	38.74	35.25	25.31	68	77	69	49	140	168	139	94
D3ZHV2	Microtubule-actin cross-linking factor 1 OS=Rattus norvegicus GN=Macf1 PE=1 SV=1 - [MACF1_RAT]	619.2	2.746E-5	0.000E0	1.201E-5	0.000E0	53.37		44.40		0.63		0.85		3		4		3		5	
D3ZW55	Inosine triphosphate pyrophosphatase OS=Rattus norvegicus GN=Itpa PE=3 SV=1 - [ITPA_RAT]	21.9	0.000E0	1.787E-4	1.915E-4	1.754E-4		331.99	278.03	324.30		38.89	59.09	64.14		5	6	7		10	12	14
D3ZZ80	Obscurin-like protein 1 OS=Rattus norvegicus GN=Obsl1 PE=2 SV=3 - [OBSL1_RAT]	197.7	4.353E-5	1.218E-4	1.848E-4	1.193E-4	36.92	0.00	0.00	0.00	2.22	0.44	0.44	0.44	2	1	1	1	3	2	2	1
D4A1J4	3-hydroxybutyrate dehydrogenase type 2 OS=Rattus norvegicus GN=Bdh2 PE=3 SV=2 - [BDH2_RAT]	26.6	2.701E-4	0.000E0	5.046E-5	0.000E0	364.71		108.95		23.27		10.20		4		2		13		3	
D4A4T9	Cysteine and histidine-rich domain-containing protein 1 OS=Rattus norvegicus GN=Chordc1 PE=3 SV=1 - [CHRD1_RAT]	37.3	0.000E0	0.000E0	0.000E0	2.788E-5				31.33				4.53				1				2
D4AE41	RNA binding motif protein, X-linked-like-1 OS=Rattus norvegicus OX=10116 GN=Rbmxl1 PE=3 SV=1 - [RMXL1_RAT]	42.2	4.686E-5	2.529E-5	4.803E-5	8.024E-5	67.52	34.25	81.61	159.13	5.41	2.06	5.41	5.41	2	1	2	2	2	1	3	4
E9PU28	Inosine-5'-monophosphate dehydrogenase 2 OS=Rattus norvegicus GN=Impdh2 PE=3 SV=1 - [IMDH2_RAT]	55.8	0.000E0	0.000E0	1.959E-4	2.056E-4			44.27	33.52			5.45	9.14			3	4			5	6
F1LM81	TOM1-like protein 1 OS=Rattus norvegicus OX=10116 GN=Tom1l1 PE=1 SV=1 - [TM1L1_RAT]	52.5	0.000E0	3.031E-5	0.000E0	0.000E0		24.33				2.74				1				2		
F1LMZ8	26S proteasome non-ATPase regulatory subunit 11 OS=Rattus norvegicus GN=Psmd11 PE=3 SV=2 - [PSD11_RAT]	47.4	0.000E0	4.410E-5	1.182E-4	8.193E-5		43.59	353.27	111.55		17.06	36.97	21.09		6	13	7		8	19	10
F1LN46	Carnitine O-palmitoyltransferase 1, brain isoform OS=Rattus norvegicus OX=10116 GN=Cpt1c PE=1 SV=1 - [CPT1C_RAT]	90.1	4.001E-5	0.000E0	0.000E0	0.000E0	26.00				0.87				1				2			
F1LNI5	Protein phosphatase 1G OS=Rattus norvegicus GN=Ppm1g PE=1 SV=2 - [PPM1G_RAT]	58.7	0.000E0	4.532E-4	7.125E-4	4.612E-4		39.65	26.17	52.53		5.54	4.06	5.35		3	2	3		5	3	5
F1LNJ2	U5 small nuclear ribonucleoprotein 200 kDa helicase OS=Rattus norvegicus GN=Snrnp200 PE=1 SV=1 - [U520_RAT]	244.7	8.564E-5	1.197E-4	1.114E-4	1.084E-4	203.79	463.09	688.40	671.90	2.43	7.53	10.94	11.59	4	12	17	18	8	21	32	31
F1LQ48	Heterogeneous nuclear ribonucleoprotein L OS=Rattus norvegicus OX=10116 GN=Hnrnpl PE=1 SV=2 - [HNRPL_RAT]	67.9	6.203E-5	5.398E-4	6.415E-4	7.038E-4	91.07	1016.07	1090.50	1102.27	7.22	37.88	45.75	54.57	3	17	18	21	5	39	41	49
F1M386	Rap guanine nucleotide exchange factor 2 OS=Rattus norvegicus GN=Rapgef2 PE=1 SV=2 - [RPGF2_RAT]	166.3	0.000E0	1.414E-4	0.000E0	0.000E0		0.00				0.47				1				1		
F1MA98	Nucleoprotein TPR OS=Rattus norvegicus GN=Tpr PE=1 SV=1 - [TPR_RAT]	267.1	1.131E-4	5.452E-4	1.016E-4	5.512E-5	594.17	646.01	699.65	240.38	7.84	8.64	7.33	3.43	16	17	15	7	28	24	25	12
G3V6S8	Serine/arginine-rich splicing factor 6 OS=Rattus norvegicus OX=10116 GN=Srsf6 PE=1 SV=1 - [SRSF6_RAT]	39.0	0.000E0	1.025E-4	4.144E-5	5.549E-4		53.36	336.47	387.76		2.65	19.17	25.07		1	7	9		1	12	17
G3V7P1	Syntaxin-12 OS=Rattus norvegicus GN=Stx12 PE=1 SV=1 - [STX12_RAT]	31.2	1.262E-4	0.000E0	0.000E0	0.000E0	141.74				26.64				5				9			
G3V7W1	Programmed cell death protein 6 OS=Rattus norvegicus OX=10116 GN=Pdcd6 PE=2 SV=1 - [PDCD6_RAT]	21.9	4.930E-5	9.927E-5	1.019E-4	8.402E-5	32.42	96.24	136.08	151.54	5.76	10.99	10.99	17.80	1	2	2	3	1	4	4	5
G3V9R8	Heterogeneous nuclear ribonucleoprotein C OS=Rattus norvegicus GN=Hnrnpc PE=1 SV=1 - [HNRPC_RAT]	32.8	0.000E0	5.020E-4	3.502E-4	6.728E-4		642.82	437.84	674.38		15.77	17.11	25.84		4	4	7		15	10	19
H1UBN0	Copine-7 OS=Rattus norvegicus GN=Cpne7 PE=1 SV=1 - [CPNE7_RAT]	61.9	6.704E-5	2.990E-5	9.170E-5	7.390E-5	39.92	65.55	58.54	68.88	3.60	1.62	3.60	3.60	2	1	2	2	2	2	3	3
M0RC99	Ras-related protein Rab-5A OS=Rattus norvegicus GN=Rab5a PE=2 SV=1 - [RAB5A_RAT]	23.6	5.910E-4	2.222E-4	1.323E-4	1.931E-4	173.16	72.70	76.08	80.49	10.23	5.12	18.60	5.12	2	1	3	1	4	2	4	2
O08557	N(G),N(G)-dimethylarginine dimethylaminohydrolase 1 OS=Rattus norvegicus GN=Ddah1 PE=1 SV=3 - [DDAH1_RAT]	31.4	1.917E-5	1.871E-4	1.780E-4	9.831E-5	21.03	295.91	256.60	173.80	2.46	32.98	38.25	21.40	1	9	9	5	1	15	15	9
O08587	Nuclear pore complex protein Nup50 OS=Rattus norvegicus GN=Nup50 PE=2 SV=2 - [NUP50_RAT]	49.8	8.833E-5	0.000E0	0.000E0	0.000E0	20.90				1.50				1				2			
O08618	Phosphoribosyl pyrophosphate synthase-associated protein 2 OS=Rattus norvegicus GN=Prpsap2 PE=2 SV=1 - [KPRB_RA	40.8	0.000E0	2.656E-5	0.000E0	0.000E0		26.53				3.52				1				1		
O08629	Transcription intermediary factor 1-beta OS=Rattus norvegicus GN=Trim28 PE=1 SV=2 - [TIF1B_RAT]	88.9	6.573E-5	2.079E-4	2.972E-4	2.940E-4	42.91	326.26	364.54	368.19	2.40	10.90	11.74	14.25	1	7	8	9	2	11	14	15
O08651	D-3-phosphoglycerate dehydrogenase OS=Rattus norvegicus GN=Phgdh PE=1 SV=3 - [SERA_RAT]	56.5	0.000E0	0.000E0	2.204E-5	2.694E-5		22.76	29.93	59.74		2.44	3.94	3.19		1	2	2		1	2	4
O08699	15-hydroxyprostaglandin dehydrogenase [NAD(+)] OS=Rattus norvegicus OX=10116 GN=Hpgd PE=2 SV=2 - [PGDH_RAT	28.9	0.000E0	3.967E-5	5.164E-5	5.183E-5		88.11	58.57	53.12		9.02	9.40	9.40		2	2	2		3	3	2
O08730	Glycogenin-1 OS=Rattus norvegicus GN=Gyg1 PE=2 SV=4 - [GLYG_RAT]	37.4	0.000E0	7.610E-5	8.555E-5	6.381E-5		51.97	37.19	35.15		3.00	3.00	3.00		1	1	1		2	1	2
O09175	Aminopeptidase B OS=Rattus norvegicus GN=Rnpep PE=1 SV=2 - [AMPB_RAT]	72.6	0.000E0	1.395E-4	2.090E-4	1.623E-4		200.57	433.32	232.61		16.15	28.00	29.23		7	12	14		13	21	22
O35077	Glycerol-3-phosphate dehydrogenase [NAD(+)], cytoplasmic OS=Rattus norvegicus GN=Gpd1 PE=1 SV=4 - [GPDA_RAT]	37.4	4.778E-5	1.676E-4	2.213E-4	1.378E-4	61.86	237.64	350.99	87.64	3.15	37.82	39.83	29.23	1	10	9	6	1	16	16	9
O35078	D-amino-acid oxidase OS=Rattus norvegicus GN=Dao PE=2 SV=1 - [OXDA_RAT]	38.8	2.154E-4	0.000E0	0.000E0	0.000E0	194.99				18.21				5				9			
O35112	CD166 antigen OS=Rattus norvegicus GN=Alcam PE=1 SV=1 - [CD166_RAT]	65.0	0.000E0	6.957E-5	0.000E0	0.000E0		39.72				6.52				3				4		
O35142	Coatomer subunit beta' OS=Rattus norvegicus GN=Copb2 PE=1 SV=3 - [COPB2_RAT]	102.5	3.735E-4	1.420E-4	1.863E-4	1.241E-4	592.94	324.89	435.38	186.23	13.48	16.80	17.35	11.93	9	11	12	7	18	18	21	11
O35217	Multiple inositol polyphosphate phosphatase 1 OS=Rattus norvegicus GN=Minpp1 PE=1 SV=3 - [MINP1_RAT]	54.6	1.289E-4	7.247E-5	9.301E-5	8.035E-5	274.78	301.03	312.50	167.97	19.54	20.17	23.28	21.21	7	7	8	7	13	14	13	11
O35244	Peroxiredoxin-6 OS=Rattus norvegicus GN=Prdx6 PE=1 SV=3 - [PRDX6_RAT]	24.8	5.733E-5	3.837E-4	5.833E-4	3.009E-4	120.11	593.29	857.12	466.76	26.34	62.50	70.09	68.75	4	11	12	12	5	22	29	23
O35263	Platelet-activating factor acetylhydrolase IB subunit gamma OS=Rattus norvegicus GN=Pafah1b3 PE=2 SV=1 - [PA1B3_RA	25.8	1.574E-4	1.978E-4	2.589E-4	2.333E-4	164.93	188.79	215.99	169.08	18.10	34.91	38.79	34.48	4	5	6	5	8	9	14	9
O35264	Platelet-activating factor acetylhydrolase IB subunit beta OS=Rattus norvegicus GN=Pafah1b2 PE=1 SV=1 - [PA1B2_RAT]	25.6	2.839E-4	3.262E-4	3.791E-4	3.148E-4	190.95	385.91	458.75	343.49	20.96	39.30	34.93	31.44	3	6	5	4	10	19	17	12
O35331	Pyridoxal kinase OS=Rattus norvegicus GN=Pdxk PE=1 SV=1 - [PDXK_RAT]	34.9	2.130E-4	9.024E-5	1.020E-4	5.162E-5	480.51	169.51	218.01	31.33	50.64	25.00	23.08	10.90	11	5	4	2	21	7	9	2
O35346	Focal adhesion kinase 1 OS=Rattus norvegicus GN=Ptk2 PE=1 SV=1 - [FAK1_RAT]	119.6	0.000E0	1.035E-4	0.000E0	0.000E0		124.22				3.70				3				6		
O35397	Caspase-6 OS=Rattus norvegicus GN=Casp6 PE=2 SV=2 - [CASP6_RAT]	31.5	5.879E-4	8.274E-4	9.364E-4	7.089E-4	54.52	244.42	211.41	153.28	13.00	15.16	15.16	13.00	4	5	5	4	9	11	9	10
O35509	Ras-related protein Rab-11B OS=Rattus norvegicus GN=Rab11b PE=1 SV=4 - [RB11B_RAT]	24.5	1.126E-3	3.234E-4	5.786E-4	4.204E-4	980.14	524.97	795.81	478.50	55.96	46.33	55.96	55.96	11	9	11	11	37	23	28	21
O35567	Bifunctional purine biosynthesis protein PURH OS=Rattus norvegicus GN=Atic PE=1 SV=2 - [PUR9_RAT]	64.2	7.900E-4	4.819E-4	5.546E-4	3.778E-4	1743.41	1410.00	1660.97	1036.78	68.24	63.51	64.53	61.32	30	26	27	27	66	58	61	51
O35760	Isopentenyl-diphosphate Delta-isomerase 1 OS=Rattus norvegicus GN=Idi1 PE=2 SV=2 - [IDI1_RAT]	26.4	1.513E-4	1.843E-4	1.563E-4	1.402E-4	126.81	210.86	180.12	192.94	21.15	27.31	34.36	33.48	4	5	6	5	7	11	9	8
O35763	Moesin OS=Rattus norvegicus GN=Msn PE=1 SV=3 - [MOES_RAT]	67.7	6.998E-4	1.287E-3	1.339E-3	1.428E-3	412.00	800.71	1073.91	695.10	18.02	37.09	39.69	36.22	12	21	24	22	27	44	56	46
O35783	Calumenin OS=Rattus norvegicus OX=10116 GN=Calu PE=1 SV=1 - [CALU_RAT]	37.0	0.000E0	0.000E0	2.658E-5	9.360E-5			21.69	117.52			4.13	13.33			1	3			1	5
O35796	Complement component 1 Q subcomponent-binding protein, mitochondrial OS=Rattus norvegicus GN=C1qbp PE=1 SV=2	31.0	5.333E-5	2.052E-4	1.456E-4	1.278E-4	87.20	162.05	148.78	151.22	8.24	9.68	15.05	9.68	2	2	3	2	3	4	4	4
O35814	Stress-induced-phosphoprotein 1 OS=Rattus norvegicus GN=Stip1 PE=1 SV=1 - [STIP1_RAT]	62.5	7.882E-5	3.370E-4	3.852E-4	3.142E-4	80.11	602.41	872.59	550.04	5.34	29.28	34.62	30.57	2	15	19	15	4	24	35	25
O35821	Myb-binding protein 1A OS=Rattus norvegicus GN=Mybbp1a PE=1 SV=2 - [MBB1A_RAT]	152.2	0.000E0	1.244E-4	1.147E-4	1.287E-4		366.65	378.05	347.97		10.34	14.58	11.61		11	16	13		20	21	22
O35824	DnaJ homolog subfamily A member 2 OS=Rattus norvegicus GN=Dnaja2 PE=2 SV=1 - [DNJA2_RAT]	45.7	0.000E0	2.600E-5	2.127E-5	2.605E-5		54.66	49.85	91.55		5.58	3.16	5.58		2	1	2		3	2	3
O35828	Coronin-7 OS=Rattus norvegicus GN=Coro7 PE=1 SV=2 - [CORO7_RAT]	100.7	1.306E-4	8.382E-5	1.406E-4	5.914E-5	176.85	201.00	392.89	87.96	8.57	10.95	13.34	5.42	5	6	7	3	10	12	13	4
O35854	Branched-chain-amino-acid aminotransferase, mitochondrial OS=Rattus norvegicus GN=Bcat2 PE=1 SV=1 - [BCAT2_RAT]	44.2	9.465E-5	6.117E-5	8.457E-5	6.493E-5	242.52	180.41	105.86	46.23	18.58	7.63	7.63	7.63	5	2	2	2	9	4	3	2
O35889	Afadin OS=Rattus norvegicus OX=10116 GN=Afdn PE=1 SV=1 - [AFAD_RAT]	207.5	4.803E-5	2.866E-5	2.133E-5	0.000E0	176.85	81.81	38.92		2.79	1.15	1.48		4	2	3		5	4	4	
O35952	Hydroxyacylglutathione hydrolase, mitochondrial OS=Rattus norvegicus GN=Hagh PE=1 SV=2 - [GLO2_RAT]	34.1	2.770E-5	3.699E-5	4.883E-5	6.664E-5	64.78	61.48	82.55	66.85	6.47	17.80	17.80	2.59	2	4	4	1	3	5	6	2
O35987	NSFL1 cofactor p47 OS=Rattus norvegicus GN=Nsfl1c PE=1 SV=1 - [NSF1C_RAT]	40.7	2.418E-4	2.316E-4	1.870E-4	1.144E-4	347.39	379.07	340.41	246.14	11.08	11.08	15.14	20.54	3	3	4	5	10	10	12	9
O54697	N-acetylated-alpha-linked acidic dipeptidase-like protein OS=Rattus norvegicus OX=10116 GN=Naaladl1 PE=1 SV=1 - [NA	80.6	7.700E-4	1.883E-4	2.885E-4	2.256E-4	1213.53	521.72	647.70	288.23	37.99	20.67	29.93	13.56	17	9	14	6	35	16	23	12
O54783	Choline/ethanolamine kinase OS=Rattus norvegicus GN=Chkb PE=1 SV=3 - [CHKB_RAT]	45.1	4.889E-5	0.000E0	3.417E-5	3.050E-5	47.68		58.62	45.99	7.36		9.90	7.36	2		3	2	3		4	2
O54975	Xaa-Pro aminopeptidase 1 OS=Rattus norvegicus GN=Xpnpep1 PE=1 SV=1 - [XPP1_RAT]	69.6	1.363E-4	3.275E-4	4.581E-4	3.000E-4	186.05	983.60	1323.85	703.27	12.52	65.01	65.65	48.15	7	25	26	22	13	48	62	37
O55012	Phosphatidylinositol-binding clathrin assembly protein OS=Rattus norvegicus GN=Picalm PE=1 SV=1 - [PICAL_RAT]	69.2	0.000E0	0.000E0	2.231E-5	3.862E-5		24.52	119.29	44.87		1.41	3.28	3.28		1	2	2		1	3	2
O55035	Peptidyl-prolyl cis-trans isomerase G OS=Rattus norvegicus GN=Ppig PE=1 SV=2 - [PPIG_RAT]	88.3	0.000E0	0.000E0	0.000E0	6.757E-5				34.15				1.33				1				1
O55096	Dipeptidyl peptidase 3 OS=Rattus norvegicus GN=Dpp3 PE=1 SV=2 - [DPP3_RAT]	83.0	1.431E-4	3.238E-4	4.004E-4	3.804E-4	139.04	1151.98	1597.32	1148.84	7.05	45.80	54.20	43.50	3	24	28	23	6	46	59	51
O55159	Epithelial cell adhesion molecule OS=Rattus norvegicus OX=10116 GN=Epcam PE=1 SV=1 - [EPCAM_RAT]	35.2	2.384E-5	2.102E-4	2.737E-4	1.095E-4	27.95	204.93	455.20	65.21	4.13	40.63	40.63	25.08	1	7	8	5	2	12	16	7
O55165	Kinesin-like protein KIF3C OS=Rattus norvegicus OX=10116 GN=Kif3c PE=2 SV=1 - [KIF3C_RAT]	89.8	0.000E0	1.665E-4	2.414E-4	1.687E-4		25.18	21.86	22.70		2.01	2.01	1.13		2	2	1		4	4	1
O55171	Acyl-coenzyme A thioesterase 2, mitochondrial OS=Rattus norvegicus GN=Acot2 PE=1 SV=1 - [ACOT2_RAT]	49.7	7.332E-5	4.250E-4	4.593E-4	5.243E-4	67.30	549.23	711.91	696.73	2.43	25.17	37.97	26.27	1	9	12	9	2	19	25	24
O70196	Prolyl endopeptidase OS=Rattus norvegicus GN=Prep PE=1 SV=1 - [PPCE_RAT]	80.7	0.000E0	8.736E-5	1.328E-4	1.184E-4		336.55	459.83	419.04		14.37	25.63	22.82		8	14	13		14	21	27
O70199	UDP-glucose 6-dehydrogenase OS=Rattus norvegicus GN=Ugdh PE=1 SV=1 - [UGDH_RAT]	54.9	0.000E0	7.735E-5	1.821E-4	2.395E-4		272.20	830.72	733.74		17.24	33.87	41.18		6	13	15		10	27	30
O70351	3-hydroxyacyl-CoA dehydrogenase type-2 OS=Rattus norvegicus GN=Hsd17b10 PE=1 SV=3 - [HCD2_RAT]	27.2	0.000E0	0.000E0	7.486E-5	4.800E-5			193.56	92.27			32.95	18.01			6	3			10	4
O70352	CD82 antigen OS=Rattus norvegicus OX=10116 GN=Cd82 PE=1 SV=1 - [CD82_RAT]	29.5	8.136E-5	0.000E0	0.000E0	0.000E0	111.99				12.03				3				5			
O70377	Synaptosomal-associated protein 23 OS=Rattus norvegicus OX=10116 GN=Snap23 PE=1 SV=1 - [SNP23_RAT]	23.2	3.350E-5	0.000E0	0.000E0	0.000E0	34.04				6.67				1				1			
O70511	Ankyrin-3 OS=Rattus norvegicus GN=Ank3 PE=1 SV=3 - [ANK3_RAT]	284.3	3.022E-5	0.000E0	2.017E-5	0.000E0	30.94		52.94		0.50		1.30		1		2		2		3	
O70513	Galectin-3-binding protein OS=Rattus norvegicus GN=Lgals3bp PE=1 SV=2 - [LG3BP_RAT]	63.7	1.097E-4	0.000E0	3.795E-5	0.000E0	370.68		101.10		20.21		9.23		8		3		11		5	
O70531	Sulfate transporter OS=Rattus norvegicus OX=10116 GN=Slc26a2 PE=2 SV=1 - [S26A2_RAT]	82.0	8.014E-6	0.000E0	0.000E0	0.000E0	37.20				1.22				1				1			
O70593	Small glutamine-rich tetratricopeptide repeat-containing protein alpha OS=Rattus norvegicus GN=Sgta PE=1 SV=1 - [SGT	34.1	4.909E-5	9.823E-5	9.089E-5	5.552E-5	46.03	308.37	264.37	101.10	12.42	18.47	18.47	12.42	3	5	5	3	5	10	8	6
O88202	60 kDa lysophospholipase OS=Rattus norvegicus OX=10116 GN=Aspg PE=1 SV=1 - [LPP60_RAT]	60.8	0.000E0	2.843E-5	3.564E-5	0.000E0		24.79	38.25			4.08	4.08			2	2			2	3	
O88267	Acyl-coenzyme A thioesterase 1 OS=Rattus norvegicus GN=Acot1 PE=1 SV=1 - [ACOT1_RAT]	46.0	7.332E-5	1.940E-4	2.705E-4	2.076E-4	67.30	351.64	441.52	436.85	2.63	14.56	28.64	17.66	1	5	8	6	2	10	16	15
O88339	Epsin-1 OS=Rattus norvegicus GN=Epn1 PE=1 SV=1 - [EPN1_RAT]	60.1	5.693E-5	3.133E-5	0.000E0	0.000E0	128.09	79.62			4.17	1.22			3	1			7	2		
O88350	Putative hydrolase RBBP9 OS=Rattus norvegicus GN=Rbbp9 PE=1 SV=2 - [RBBP9_RAT]	21.0	0.000E0	6.455E-5	6.785E-5	0.000E0		34.97	50.99			6.99	29.57			1	3			1	3	
O88370	Phosphatidylinositol 5-phosphate 4-kinase type-2 gamma OS=Rattus norvegicus GN=Pip4k2c PE=1 SV=1 - [PI42C_RAT]	47.0	4.040E-5	3.225E-5	0.000E0	0.000E0	28.19	40.66			3.33	3.33			1	1			1	1		
O88506	STE20/SPS1-related proline-alanine-rich protein kinase OS=Rattus norvegicus GN=Stk39 PE=2 SV=2 - [STK39_RAT]	60.0	1.350E-4	1.103E-4	6.445E-5	5.050E-5	49.48	49.42	29.80	26.20	1.27	1.27	1.27	1.27	1	1	1	1	2	2	1	1
O88600	Heat shock 70 kDa protein 4 OS=Rattus norvegicus GN=Hspa4 PE=1 SV=1 - [HSP74_RAT]	94.0	4.247E-4	4.736E-4	4.736E-4	3.863E-4	481.08	1883.22	2525.03	1394.59	17.14	41.79	50.36	41.67	14	29	35	30	30	68	85	56
O88656	Actin-related protein 2/3 complex subunit 1B OS=Rattus norvegicus GN=Arpc1b PE=2 SV=3 - [ARC1B_RAT]	41.0	4.661E-5	1.761E-4	2.310E-4	1.625E-4	67.72	322.41	508.41	434.56	9.14	30.38	36.29	30.65	3	8	11	9	4	12	20	15
O88761	26S proteasome non-ATPase regulatory subunit 1 OS=Rattus norvegicus GN=Psmd1 PE=2 SV=1 - [PSMD1_RAT]	105.7	0.000E0	5.812E-5	1.513E-4	1.100E-4		130.27	473.35	229.13		14.27	24.55	10.28		9	14	7		11	24	11
O88767	Protein DJ-1 OS=Rattus norvegicus GN=Park7 PE=1 SV=1 - [PARK7_RAT]	20.0	0.000E0	2.158E-4	5.003E-4	3.840E-4		447.79	899.65	515.87		68.78	69.31	69.31		8	9	9		15	24	21
O88778	Protein bassoon OS=Rattus norvegicus GN=Bsn PE=1 SV=3 - [BSN_RAT]	418.2	0.000E0	0.000E0	0.000E0	5.130E-4				22.09				0.36				2				3
O88797	Disabled homolog 2 OS=Rattus norvegicus OX=10116 GN=Dab2 PE=1 SV=1 - [DAB2_RAT]	82.3	1.973E-4	1.889E-4	3.269E-4	3.460E-4	416.05	258.12	319.65	305.73	6.51	5.99	9.38	9.38	5	4	6	6	10	6	10	10
O88813	Long-chain-fatty-acid--CoA ligase 5 OS=Rattus norvegicus OX=10116 GN=Acsl5 PE=1 SV=1 - [ACSL5_RAT]	76.4	8.471E-5	0.000E0	5.242E-5	0.000E0	278.54		20.15		10.10		1.76		6		1		10		2	
O88902	Tyrosine-protein phosphatase non-receptor type 23 (Fragment) OS=Rattus norvegicus GN=Ptpn23 PE=1 SV=2 - [PTN23_	163.4	0.000E0	3.665E-5	0.000E0	0.000E0		25.73				0.87				1				1		
O88989	Malate dehydrogenase, cytoplasmic OS=Rattus norvegicus GN=Mdh1 PE=1 SV=3 - [MDHC_RAT]	36.5	2.921E-3	2.598E-3	3.132E-3	2.856E-3	1650.97	1456.39	1668.81	1274.16	51.20	43.41	53.59	53.59	14	12	17	15	44	40	45	42
O89046	Coronin-1B OS=Rattus norvegicus GN=Coro1b PE=1 SV=1 - [COR1B_RAT]	53.8	7.029E-5	2.052E-4	3.599E-4	2.889E-4	33.46	271.19	438.75	332.15	2.27	8.47	27.89	15.29	1	4	8	6	2	7	13	12
O89049	Thioredoxin reductase 1, cytoplasmic OS=Rattus norvegicus GN=Txnrd1 PE=1 SV=5 - [TRXR1_RAT]	54.6	8.475E-4	1.898E-4	5.014E-4	3.132E-4	1448.28	498.94	851.58	450.04	56.51	36.87	46.89	40.88	18	11	14	12	54	21	28	23
P00406	Cytochrome c oxidase subunit 2 OS=Rattus norvegicus OX=10116 GN=Mtco2 PE=1 SV=3 - [COX2_RAT]	25.9	3.140E-5	7.612E-5	2.960E-4	3.246E-5	46.82	55.87	65.80	69.73	4.41	7.49	7.49	4.41	1	2	2	1	2	3	3	2
P00481	Ornithine carbamoyltransferase, mitochondrial OS=Rattus norvegicus OX=10116 GN=Otc PE=1 SV=1 - [OTC_RAT]	39.9	0.000E0	4.952E-4	6.123E-4	3.997E-4		647.61	789.38	473.15		57.34	76.55	52.82		17	19	14		31	42	23
P00502	Glutathione S-transferase alpha-1 OS=Rattus norvegicus GN=Gsta1 PE=1 SV=3 - [GSTA1_RAT]	25.6	4.394E-3	5.099E-4	1.089E-3	9.131E-4	833.71	147.67	466.49	298.28	38.74	21.17	27.03	27.03	10	6	7	7	47	10	18	15
P00507	Aspartate aminotransferase, mitochondrial OS=Rattus norvegicus GN=Got2 PE=1 SV=2 - [AATM_RAT]	47.3	1.182E-3	6.355E-4	8.141E-4	5.541E-4	1721.49	1346.86	1401.38	976.32	47.21	39.77	58.60	49.53	18	14	19	16	59	42	47	34
P00564	Creatine kinase M-type OS=Rattus norvegicus GN=Ckm PE=1 SV=2 - [KCRM_RAT]	43.0	2.470E-4	1.906E-4	1.944E-4	2.137E-4	38.17	116.82	140.68	64.50	2.10	20.21	11.81	9.45	1	5	4	3	2	8	7	5
P00689	Pancreatic alpha-amylase OS=Rattus norvegicus GN=Amy2 PE=2 SV=2 - [AMYP_RAT]	57.1	1.026E-3	2.137E-3	2.562E-3	1.017E-3	1121.56	2272.57	2493.74	1429.91	49.21	59.25	67.32	58.27	18	22	23	21	42	72	72	52
P00731	Carboxypeptidase A1 OS=Rattus norvegicus OX=10116 GN=Cpa1 PE=2 SV=2 - [CBPA1_RAT]	47.2	4.859E-3	5.446E-4	7.390E-4	6.961E-4	2504.71	702.39	936.04	720.74	52.98	34.37	45.11	45.11	13	9	11	11	70	27	36	28
P00758	Kallikrein-1 OS=Rattus norvegicus OX=10116 GN=Ngfg PE=2 SV=2 - [KLK1_RAT]	28.8	1.293E-4	1.144E-4	9.244E-5	1.165E-4	107.83	82.36	44.21	59.76	18.77	8.81	3.83	8.81	3	2	1	2	5	4	2	4
P00762	Anionic trypsin-1 OS=Rattus norvegicus GN=Prss1 PE=1 SV=1 - [TRY1_RAT]	25.9	6.143E-3	7.520E-3	1.237E-2	1.202E-2	424.70	344.57	346.34	418.00	21.14	36.59	14.23	36.59	3	4	2	4	16	12	10	15
P00763	Anionic trypsin-2 OS=Rattus norvegicus OX=10116 GN=Prss2 PE=1 SV=2 - [TRY2_RAT]	26.2	3.288E-4	1.461E-4	6.295E-5	3.846E-5	385.16	74.93	62.42	67.98	31.71	4.07	19.51	19.51	4	1	2	2	12	2	3	4
P00770	Mast cell protease 2 OS=Rattus norvegicus GN=Mcpt2 PE=1 SV=1 - [MCPT2_RAT]	27.1	6.628E-4	1.926E-4	1.445E-4	1.690E-4	248.01	119.25	105.59	145.19	53.85	21.86	13.77	8.91	7	4	3	2	14	6	6	4
P00773	Chymotrypsin-like elastase family member 1 OS=Rattus norvegicus OX=10116 GN=Cela1 PE=1 SV=1 - [CELA1_RAT]	29.0	6.469E-5	4.694E-5	5.356E-5	1.508E-4	66.52	132.10	135.71	58.03	7.14	15.41	15.41	2.63	2	3	3	1	4	6	4	2
P00774	Chymotrypsin-like elastase family member 2A OS=Rattus norvegicus OX=10116 GN=Cela2a PE=2 SV=1 - [CEL2A_RAT]	28.9	3.735E-4	2.673E-4	2.661E-4	4.070E-4	360.68	416.97	435.32	543.43	44.28	47.60	44.28	63.10	8	8	8	10	15	12	14	21
P00786	Pro-cathepsin H OS=Rattus norvegicus GN=Ctsh PE=1 SV=1 - [CATH_RAT]	37.1	4.246E-3	2.176E-4	2.806E-4	1.921E-4	2400.39	305.54	563.20	364.02	47.15	30.93	34.83	36.04	11	6	5	6	99	14	14	13
P00787	Cathepsin B OS=Rattus norvegicus GN=Ctsb PE=1 SV=2 - [CATB_RAT]	37.4	2.740E-4	7.727E-4	1.169E-3	9.726E-4	308.09	1215.99	2106.06	1572.01	22.71	41.59	41.59	41.59	5	12	12	12	11	35	49	43
P00884	Fructose-bisphosphate aldolase B OS=Rattus norvegicus GN=Aldob PE=1 SV=2 - [ALDOB_RAT]	39.6	3.394E-4	1.538E-3	1.870E-3	1.189E-3	729.87	1675.82	1881.80	1404.81	31.32	56.32	58.52	56.04	9	16	17	15	21	48	56	37

P01015	Angiotensinogen OS=Rattus norvegicus GN=Agt PE=1 SV=1 - [ANGT_RAT]	51.9	1.157E-4	6.475E-5	9.085E-5	7.000E-5	224.82	138.31	179.87	146.12	12.37	10.48	4.82	10.48	4	3	2	3	10	5	4	5
P01026	Complement C3 OS=Rattus norvegicus GN=C3 PE=1 SV=3 - [CO3_RAT]	186.3	8.251E-4	2.028E-4	4.040E-4	1.871E-4	2114.78	885.83	1893.03	734.34	28.56	20.02	34.64	16.60	37	27	44	22	80	45	78	39
P01041	Cystatin-B OS=Rattus norvegicus GN=Cstb PE=1 SV=1 - [CYTB_RAT]	11.2	8.626E-4	5.802E-4	6.227E-4	3.918E-4	120.31	137.90	137.08	97.57	42.86	34.69	34.69	35.71	5	3	3	4	12	9	9	6
P01048	T-kininogen 1 OS=Rattus norvegicus GN=Map1 PE=1 SV=1 - [KNT1_RAT]	47.7	1.411E-3	1.842E-3	2.246E-3	1.710E-3	1277.38	1379.58	1974.27	1648.90	49.07	48.37	48.37	48.37	18	17	17	17	50	57	78	74
P01174	Whey acidic protein OS=Rattus norvegicus OX=10116 GN=Wap PE=1 SV=2 - [WAP_RAT]	14.8	1.627E-3	0.000E0	0.000E0	0.000E0	152.04				10.95				1				5			
P01835	Ig kappa chain C region, B allele OS=Rattus norvegicus PE=1 SV=1 - [KACB_RAT]	11.6	4.489E-3	4.281E-4	7.665E-4	3.712E-4	1487.10	495.28	811.34	340.06	48.11	81.13	81.13	41.51	5	6	7	4	33	17	26	13
P01836	Ig kappa chain C region, A allele OS=Rattus norvegicus PE=1 SV=1 - [KACA_RAT]	11.7	7.921E-3	2.075E-4	5.826E-4	2.482E-4	2153.57	340.24	610.98	250.94	83.96	33.02	43.40	35.85	7	3	5	3	56	9	18	9
P01946	Hemoglobin subunit alpha-1/2 OS=Rattus norvegicus GN=Hba1 PE=1 SV=3 - [HBA_RAT]	15.3	4.876E-2	4.962E-2	4.147E-2	3.060E-2	2911.98	3039.99	3153.64	2652.75	91.55	95.07	95.07	95.07	11	12	13	12	97	104	104	89
P02091	Hemoglobin subunit beta-1 OS=Rattus norvegicus GN=Hbb PE=1 SV=3 - [HBB1_RAT]	16.0	6.454E-2	8.019E-2	8.087E-2	6.510E-2	6388.18	11787.05	12254.21	7808.08	91.16	91.16	91.16	91.16	15	14	18	14	299	506	510	367
P02262	Histone H2A type 1 OS=Rattus norvegicus PE=1 SV=2 - [H2A1_RAT]	14.1	1.147E-3	2.429E-2	9.155E-3	2.847E-2	254.24	768.77	517.53	816.35	21.54	40.77	26.92	35.38	2	5	3	5	10	24	15	28
P02401	60S acidic ribosomal protein P2 OS=Rattus norvegicus GN=Rplp2 PE=1 SV=2 - [RLA2_RAT]	11.7	0.000E0	1.180E-4	1.132E-4	6.700E-5		74.16	95.05	63.97		10.43	10.43	10.43		1	1	1		2	2	2
P02454	Collagen alpha-1(I) chain OS=Rattus norvegicus GN=Col1a1 PE=1 SV=5 - [CO1A1_RAT]	137.9	0.000E0	6.846E-5	6.473E-5	5.943E-5		63.95	58.67	57.84		4.75	0.69	3.03		4	1	3		5	2	5
P02466	Collagen alpha-2(I) chain OS=Rattus norvegicus OX=10116 GN=Col1a2 PE=1 SV=3 - [CO1A2_RAT]	129.5	0.000E0	2.286E-5	0.000E0	0.000E0	24.00	43.09			0.87	0.87			1	1			1	1		
P02634	Protein S100-G OS=Rattus norvegicus OX=10116 GN=S100g PE=1 SV=3 - [S100G_RAT]	9.0	6.798E-5	1.153E-4	9.105E-5	2.322E-4	56.59	68.28	78.56	64.90	15.19	15.19	26.58	15.19	1	1	2	1	2	2	4	3
P02650	Apolipoprotein E OS=Rattus norvegicus GN=Apoe PE=1 SV=2 - [APOE_RAT]	35.7	1.289E-4	2.152E-4	1.789E-4	1.392E-4	198.83	646.90	556.10	394.52	16.35	27.24	25.96	23.08	5	8	8	7	8	17	16	15
P02651	Apolipoprotein A-IV OS=Rattus norvegicus GN=Apoa4 PE=1 SV=2 - [APOA4_RAT]	44.4	3.413E-4	4.853E-4	1.026E-3	6.446E-4	483.00	764.76	1693.71	1351.16	30.95	48.34	67.52	68.03	10	16	23	21	18	30	54	45
P02680	Fibrinogen gamma chain OS=Rattus norvegicus GN=Fgg PE=1 SV=3 - [FIBG_RAT]	50.6	7.622E-4	6.388E-4	7.080E-4	4.473E-4	768.63	808.24	742.98	657.96	46.74	48.54	43.37	48.09	15	16	14	16	32	35	28	28
P02692	Fatty acid-binding protein, liver OS=Rattus norvegicus OX=10116 GN=Fabp1 PE=1 SV=1 - [FABPL_RAT]	14.3	1.463E-2	5.849E-4	4.338E-4	8.249E-5	4225.24	648.79	612.22	172.50	69.29	68.50	67.72	50.39	8	7	6	5	146	20	18	7
P02693	Fatty acid-binding protein, intestinal OS=Rattus norvegicus OX=10116 GN=Fabp2 PE=1 SV=4 - [FABPI_RAT]	15.1	7.838E-3	1.466E-3	1.816E-3	7.956E-4	1216.99	495.36	580.95	439.40	46.21	36.36	36.36	28.03	7	4	4	3	32	13	14	11
P02696	Retinol-binding protein 1 OS=Rattus norvegicus GN=Rbp1 PE=1 SV=2 - [RET1_RAT]	15.8	7.739E-5	9.260E-5	9.139E-5	3.935E-5	135.15	149.79	135.68	51.51	37.04	17.78	17.78	8.89	4	2	2	1	5	4	3	1
P02761	Major urinary protein OS=Rattus norvegicus OX=10116 PE=1 SV=1 - [MUP_RAT]	20.7	1.220E-4	0.000E0	0.000E0	0.000E0	76.53				13.81				3				5			
P02767	Transthyretin OS=Rattus norvegicus GN=Ttr PE=1 SV=1 - [TTHY_RAT]	15.7	2.049E-2	1.056E-3	3.669E-3	1.448E-3	1587.05	708.94	1151.55	693.14	63.27	62.59	63.27	62.59	8	7	8	7	58	21	34	22
P02770	Serum albumin OS=Rattus norvegicus GN=Alb PE=1 SV=2 - [ALBU_RAT]	68.7	3.724E-2	3.291E-2	3.830E-2	4.105E-2	11669.76	12889.44	13769.59	12926.89	77.30	77.30	80.59	77.80	48	48	53	49	374	383	420	411
P02773	Alpha-fetoprotein OS=Rattus norvegicus GN=Afp PE=2 SV=1 - [FETA_RAT]	68.3	3.553E-3	3.278E-3	4.018E-3	3.848E-3	5060.85	5722.35	6506.01	5872.08	75.61	74.47	74.47	75.61	38	37	37	38	168	179	193	180
P02793	Ferritin light chain 1 OS=Rattus norvegicus GN=Ftl1 PE=1 SV=3 - [FRIL1_RAT]	20.7	1.933E-2	6.585E-3	1.753E-2	1.381E-2	2919.07	2213.64	3455.98	2844.74	83.06	75.41	75.41	75.41	14	13	13	13	83	61	90	73
P02803	Metallothionein-1 OS=Rattus norvegicus OX=10116 GN=Mt1 PE=1 SV=1 - [MT1_RAT]	6.0	1.265E-4	4.146E-5	5.702E-5	1.088E-4	109.21	126.85	101.08	109.78	19.67	19.67	19.67	19.67	1	1	1	1	2	2	2	2
P04041	Glutathione peroxidase 1 OS=Rattus norvegicus GN=Gpx1 PE=1 SV=4 - [GPX1_RAT]	22.3	9.691E-4	2.437E-4	4.448E-4	2.028E-4	808.00	244.34	497.84	172.98	58.21	52.24	62.19	39.30	10	8	10	6	31	15	22	9
P04182	Ornithine aminotransferase, mitochondrial OS=Rattus norvegicus GN=Oat PE=1 SV=1 - [OAT_RAT]	48.3	5.342E-4	7.570E-3	7.576E-3	4.453E-3	963.90	4204.50	4217.76	3200.91	46.24	77.68	77.68	75.17	17	24	24	22	43	132	135	120
P04256	Heterogeneous nuclear ribonucleoprotein A1 OS=Rattus norvegicus GN=Hnrnpa1 PE=1 SV=3 - [ROA1_RAT]	34.2	1.867E-3	3.195E-3	2.559E-3	2.697E-3	1677.22	2606.59	2180.00	2174.18	48.13	48.13	48.13	53.13	19	20	19	19	63	87	78	78
P04276	Vitamin D-binding protein OS=Rattus norvegicus GN=Gc PE=1 SV=1 - [VTDB_RAT]	53.5	9.655E-5	6.546E-5	5.609E-5	2.552E-5	247.27	147.52	141.74	54.79	14.71	12.39	6.30	3.57	5	4	2	2	8	6	4	4
P04355	Metallothionein-2 OS=Rattus norvegicus OX=10116 GN=Mt2 PE=1 SV=1 - [MT2_RAT]	6.1	8.218E-5	1.950E-5	5.018E-5	5.435E-5	115.32	88.13	96.13	97.71	32.79	19.67	19.67	32.79	2	1	1	2	3	2	2	3
P04550	Parathymosin OS=Rattus norvegicus GN=Ptms PE=1 SV=2 - [PTMS_RAT]	11.6	0.000E0	0.000E0	2.587E-5	1.982E-5		69.58	86.83	44.05		20.59	20.59	11.76		2	2	1		3	2	1
P04636	Malate dehydrogenase, mitochondrial OS=Rattus norvegicus GN=Mdh2 PE=1 SV=2 - [MDHM_RAT]	35.7	5.873E-3	1.962E-3	2.391E-3	1.377E-3	3905.68	2844.80	3230.78	2435.16	65.68	62.13	62.13	56.51	18	17	17	15	104	73	78	62
P04639	Apolipoprotein A-I OS=Rattus norvegicus GN=Apoa1 PE=1 SV=2 - [APOA1_RAT]	30.0	7.929E-4	3.872E-4	5.160E-4	2.624E-4	1186.97	762.31	967.91	421.84	62.93	55.21	66.41	46.33	16	14	17	11	46	35	40	21
P04642	L-lactate dehydrogenase A chain OS=Rattus norvegicus GN=Ldha PE=1 SV=1 - [LDHA_RAT]	36.4	4.582E-4	2.382E-3	3.283E-3	2.526E-3	519.00	1928.76	2534.48	1971.66	32.23	78.61	78.61	72.59	9	23	23	22	20	73	97	75
P04644	40S ribosomal protein S17 OS=Rattus norvegicus GN=Rps17 PE=1 SV=3 - [RS17_RAT]	15.5	0.000E0	7.267E-5	1.306E-4	1.402E-4		29.39	100.38	126.96		8.15	24.44	24.44		1	2	2		2	3	4
P04646	60S ribosomal protein L35a OS=Rattus norvegicus OX=10116 GN=Rpl35a PE=3 SV=1 - [RL35A_RAT]	12.5	0.000E0	1.482E-4	1.520E-4	1.164E-4		156.05	162.05	157.81		29.09	38.18	29.09		4	5	4		10	10	9
P04692	Tropomyosin alpha-1 chain OS=Rattus norvegicus GN=Tpm1 PE=1 SV=3 - [TPM1_RAT]	32.7	1.673E-3	2.285E-3	3.108E-3	2.298E-3	1238.03	1522.35	1847.70	1501.59	51.06	54.58	57.04	52.46	18	24	25	22	45	63	70	57
P04762	Catalase OS=Rattus norvegicus GN=Cat PE=1 SV=3 - [CATA_RAT]	59.7	5.530E-5	2.392E-4	2.932E-4	2.151E-4	124.18	718.49	705.33	607.88	7.40	33.40	37.38	33.02	3	14	15	13	5	30	32	27
P04764	Alpha-enolase OS=Rattus norvegicus GN=Eno1 PE=1 SV=4 - [ENOA_RAT]	47.1	3.730E-3	4.275E-3	4.940E-3	3.378E-3	4312.31	5115.06	6198.40	4301.18	75.35	75.35	76.73	74.65	27	27	29	27	119	145	175	133
P04785	Protein disulfide-isomerase OS=Rattus norvegicus GN=P4hb PE=1 SV=2 - [PDIA1_RAT]	56.9	4.736E-3	3.499E-3	3.634E-3	1.880E-3	3390.60	3670.40	3908.00	2841.57	61.89	62.08	60.51	62.28	29	30	29	31	118	122	125	97
P04797	Glyceraldehyde-3-phosphate dehydrogenase OS=Rattus norvegicus GN=Gapdh PE=1 SV=3 - [G3P_RAT]	35.8	2.856E-4	3.849E-4	1.398E-3	1.067E-3	294.98	555.68	1325.70	1241.89	38.14	42.04	53.15	48.95	8	9	13	11	15	24	46	49
P04897	Guanine nucleotide-binding protein G(i) subunit alpha-2 OS=Rattus norvegicus GN=Gnai2 PE=1 SV=3 - [GNAI2_RAT]	40.5	6.631E-5	6.943E-5	1.296E-4	9.351E-5	77.99	146.55	149.38	65.67	9.01	14.37	12.68	9.01	3	4	4	3	5	6	6	5
P04903	Glutathione S-transferase alpha-2 OS=Rattus norvegicus GN=Gsta2 PE=2 SV=2 - [GSTA2_RAT]	25.5	3.869E-3	4.970E-4	9.950E-4	8.558E-4	545.20	174.59	323.05	229.77	38.74	21.17	21.17	21.17	10	6	6	6	40	11	14	12
P04904	Glutathione S-transferase alpha-3 OS=Rattus norvegicus GN=Gsta3 PE=1 SV=3 - [GSTA3_RAT]	25.3	3.250E-3	4.773E-4	9.130E-4	8.122E-4	269.89	180.46	270.15	237.10	19.91	16.74	16.74	16.74	6	5	5	5	13	9	11	12
P04905	Glutathione S-transferase Mu 1 OS=Rattus norvegicus GN=Gstm1 PE=1 SV=2 - [GSTM1_RAT]	25.9	4.337E-4	4.947E-4	4.861E-4	4.811E-4	501.87	544.66	543.72	393.04	43.12	45.87	50.00	36.24	10	10	10	8	19	23	22	18
P04906	Glutathione S-transferase P OS=Rattus norvegicus GN=Gstp1 PE=1 SV=2 - [GSTP1_RAT]	23.4	3.176E-3	7.370E-3	7.108E-3	6.498E-3	1706.75	3490.91	3402.42	2815.32	64.76	64.76	64.76	64.76	12	12	12	12	58	105	95	93
P04916	Retinol-binding protein 4 OS=Rattus norvegicus GN=Rbp4 PE=1 SV=1 - [RET4_RAT]	23.2	3.613E-5	0.000E0	3.394E-5	0.000E0	74.32		62.80		14.93		8.96		3		2		4		2	
P04937	Fibronectin OS=Rattus norvegicus GN=Fn1 PE=1 SV=2 - [FINC_RAT]	272.3	3.715E-4	6.936E-4	9.118E-4	8.972E-4	829.52	1109.57	740.35	863.51	17.32	20.87	17.00	20.10	31	32	28	32	49	59	44	56
P04961	Proliferating cell nuclear antigen OS=Rattus norvegicus GN=Pcna PE=1 SV=1 - [PCNA_RAT]	28.7	0.000E0	4.700E-5	1.669E-4	1.305E-4		84.22	97.85	117.57		8.43	8.43	18.77		2	2	4		3	5	7
P05065	Fructose-bisphosphate aldolase A OS=Rattus norvegicus GN=Aldoa PE=1 SV=2 - [ALDOA_RAT]	39.3	2.157E-3	2.411E-3	2.958E-3	2.209E-3	2151.01	2633.26	2619.28	2135.57	76.92	76.65	83.24	74.45	24	24	27	24	74	86	92	76
P05197	Elongation factor 2 OS=Rattus norvegicus GN=Eef2 PE=1 SV=4 - [EF2_RAT]	95.2	5.914E-4	1.453E-3	1.808E-3	1.117E-3	1519.58	4050.46	4963.99	3331.46	37.53	63.40	60.49	65.85	28	43	42	43	65	162	175	140
P05369	Farnesyl pyrophosphate synthase OS=Rattus norvegicus GN=Fdps PE=2 SV=2 - [FPPS_RAT]	40.8	1.539E-5	3.057E-4	5.008E-4	4.772E-4	25.42	1149.07	1332.94	1154.84	2.55	41.64	49.58	41.64	1	15	15	14	1	39	41	40
P05370	Glucose-6-phosphate 1-dehydrogenase OS=Rattus norvegicus GN=G6pdx PE=1 SV=2 - [G6PD_RAT]	59.3	0.000E0	1.325E-4	1.481E-4	1.341E-4		144.61	278.29	175.91		12.82	18.45	13.20		6	8	6		9	12	11
P05426	60S ribosomal protein L7 OS=Rattus norvegicus GN=Rpl7 PE=1 SV=2 - [RL7_RAT]	30.3	7.480E-5	9.469E-4	1.041E-3	1.025E-3	156.74	684.77	945.63	806.77	7.31	45.77	46.54	48.85	2	11	15	13	3	33	42	37
P05544	Serine protease inhibitor A3L OS=Rattus norvegicus GN=Serpina3l PE=1 SV=3 - [SPA3L_RAT]	46.2	3.488E-3	6.315E-4	9.312E-4	4.755E-4	2402.88	971.73	1567.57	935.12	46.97	37.53	56.17	34.87	20	15	21	12	78	36	59	34
P05545	Serine protease inhibitor A3K OS=Rattus norvegicus GN=Serpina3k PE=1 SV=3 - [SPA3K_RAT]	46.5	3.488E-3	6.315E-4	9.312E-4	4.755E-4	2772.54	938.31	1797.94	741.98	46.15	42.79	46.15	42.79	18	15	18	14	85	41	61	37
P05708	Hexokinase-1 OS=Rattus norvegicus GN=Hk1 PE=1 SV=4 - [HXK1_RAT]	102.3	0.000E0	4.542E-5	4.051E-5	3.268E-5		95.95	51.40	91.62		8.93	2.51	4.36		7	2	4		11	3	8
P05712	Ras-related protein Rab-2A OS=Rattus norvegicus GN=Rab2a PE=1 SV=1 - [RAB2A_RAT]	23.5	4.617E-5	6.908E-5	1.560E-4	9.721E-5	20.25	277.75	264.65	215.61	6.60	26.89	38.21	30.66	1	4	6	5	1	7	9	8
P05943	Protein S100-A10 OS=Rattus norvegicus GN=S100a10 PE=1 SV=2 - [S10AA_RAT]	11.1	8.988E-4	7.168E-4	6.282E-4	1.462E-3	283.97	64.94	97.78	27.68	35.79	35.79	35.79	17.89	3	3	3	1	12	7	8	4
P05964	Protein S100-A6 OS=Rattus norvegicus GN=S100a6 PE=1 SV=3 - [S10A6_RAT]	10.0	6.200E-4	1.203E-3	7.427E-4	1.404E-3	200.51	244.25	211.37	198.67	26.97	26.97	57.30	26.97	3	3	4	3	8	10	9	8
P06214	Delta-aminolevulinic acid dehydratase OS=Rattus norvegicus GN=Alad PE=1 SV=1 - [HEM2_RAT]	36.0	3.749E-4	1.682E-4	2.122E-4	1.470E-4	680.85	386.43	571.82	270.84	49.70	40.30	53.64	34.85	13	9	12	9	32	16	22	13
P06238	Alpha-2-macroglobulin OS=Rattus norvegicus GN=A2m PE=2 SV=2 - [A2MG_RAT]	163.7	4.755E-4	3.228E-4	8.296E-4	2.387E-4	1486.37	1205.20	2122.73	957.74	30.98	28.74	39.88	23.10	35	29	42	25	71	53	91	44
P06302	Prothymosin alpha OS=Rattus norvegicus OX=10116 GN=Ptma PE=1 SV=2 - [PTMA_RAT]	12.4	0.000E0	3.864E-5	0.000E0	5.069E-5		48.81		57.70		8.93		8.93		1		1		1		2
P06399	Fibrinogen alpha chain OS=Rattus norvegicus GN=Fga PE=1 SV=3 - [FIBA_RAT]	86.6	3.913E-4	3.750E-4	4.766E-4	3.911E-4	474.29	503.64	645.66	584.88	14.83	11.76	17.52	16.37	12	10	14	13	23	24	28	30
P06685	Sodium/potassium-transporting ATPase subunit alpha-1 OS=Rattus norvegicus GN=Atp1a1 PE=1 SV=1 - [AT1A1_RAT]	113.0	1.579E-4	3.825E-4	3.607E-4	1.923E-4	413.93	1231.95	1062.48	266.46	12.51	25.22	27.47	10.65	9	21	20	9	17	46	38	15
P06686	Sodium/potassium-transporting ATPase subunit alpha-2 OS=Rattus norvegicus GN=Atp1a2 PE=1 SV=1 - [AT1A2_RAT]	112.1	1.113E-4	2.023E-4	1.705E-4	7.490E-5	123.76	720.07	563.54	104.27	9.22	13.14	14.51	7.55	6	10	10	5	10	21	20	8
P06760	Beta-glucuronidase OS=Rattus norvegicus GN=Gusb PE=2 SV=1 - [BGLR_RAT]	74.7	4.508E-4	7.986E-5	1.094E-4	1.263E-4	571.76	140.30	212.14	201.48	33.33	13.27	10.80	10.49	15	7	6	7	32	12	12	12
P06761	78 kDa glucose-regulated protein OS=Rattus norvegicus GN=Hspa5 PE=1 SV=1 - [GRP78_RAT]	72.3	7.155E-3	4.397E-3	3.977E-3	3.101E-3	6530.42	5631.30	5487.43	4523.79	51.68	51.38	51.99	48.93	39	32	32	32	194	162	161	140
P06762	Heme oxygenase 1 OS=Rattus norvegicus OX=10116 GN=Hmox1 PE=1 SV=1 - [HMOX1_RAT]	33.0	6.528E-4	0.000E0	1.781E-4	0.000E0	307.71		146.43		41.52		34.60		7		5		15		8	
P06766	DNA polymerase beta OS=Rattus norvegicus OX=10116 GN=Polb PE=1 SV=4 - [DPOLB_RAT]	38.3	0.000E0	0.000E0	0.000E0	3.295E-5				25.87				4.48				1				1
P06768	Retinol-binding protein 2 OS=Rattus norvegicus GN=Rbp2 PE=1 SV=3 - [RET2_RAT]	15.6	3.798E-3	1.178E-3	1.363E-3	3.486E-4	956.33	590.27	614.60	302.52	67.16	52.99	52.99	40.30	8	6	7	5	32	16	22	9
P06866	Haptoglobin OS=Rattus norvegicus GN=Hp PE=1 SV=3 - [HPT_RAT]	38.5	2.007E-4	8.593E-5	2.386E-4	6.522E-5	229.45	153.25	365.22	76.82	21.33	8.93	21.90	6.63	7	3	7	2	10	6	14	3
P07150	Annexin A1 OS=Rattus norvegicus GN=Anxa1 PE=1 SV=2 - [ANXA1_RAT]	38.8	3.541E-5	8.057E-5	1.041E-4	4.737E-5	49.94	209.87	264.55	95.61	5.49	21.10	25.14	12.43	2	6	7	3	2	8	10	4
P07153	Dolichyl-diphosphooligosaccharide--protein glycosyltransferase subunit 1 OS=Rattus norvegicus GN=Rpn1 PE=2 SV=1 - [R	68.3	0.000E0	4.291E-5	3.620E-5	2.812E-5		155.07	122.58	54.68		9.09	6.12	6.12		5	3	3		8	6	5
P07154	Cathepsin L1 OS=Rattus norvegicus GN=Ctsl PE=1 SV=2 - [CATL1_RAT]	37.6	1.061E-3	1.711E-4	5.103E-4	3.545E-4	870.33	287.13	685.20	362.07	52.69	29.64	47.60	44.01	12	6	9	8	34	12	24	21
P07314	Glutathione hydrolase 1 proenzyme OS=Rattus norvegicus OX=10116 GN=Ggt1 PE=1 SV=4 - [GGT1_RAT]	61.6	4.456E-5	4.190E-5	6.327E-5	8.096E-5	58.82	27.60	46.02	73.15	6.34	1.58	3.35	8.45	3	1	2	4	3	1	2	5
P07323	Gamma-enolase OS=Rattus norvegicus GN=Eno2 PE=1 SV=2 - [ENOG_RAT]	47.1	1.186E-3	1.281E-3	1.873E-3	1.128E-3	1069.67	1364.57	1697.19	1040.55	14.06	32.49	36.18	19.82	4	8	9	6	21	36	40	32
P07335	Creatine kinase B-type OS=Rattus norvegicus GN=Ckb PE=1 SV=2 - [KCRB_RAT]	42.7	2.686E-3	4.488E-3	5.279E-3	4.819E-3	3507.62	6106.84	6826.21	5347.50	71.39	71.92	82.15	71.92	21	22	22	22	126	211	239	196
P07338	Chymotrypsinogen B OS=Rattus norvegicus OX=10116 GN=Ctrb1 PE=1 SV=1 - [CTRB1_RAT]	27.8	2.874E-3	2.236E-3	1.164E-3	2.038E-4	1799.35	1776.46	1185.12	336.85	67.30	64.64	67.30	37.26	10	9	10	6	59	56	35	11
P07340	Sodium/potassium-transporting ATPase subunit beta-1 OS=Rattus norvegicus OX=10116 GN=Atp1b1 PE=1 SV=1 - [AT1B	35.2	1.110E-4	1.945E-4	2.824E-4	1.534E-4	59.76	95.56	161.23	103.06	7.24	10.53	8.88	12.50	2	3	2	3	4	9	6	5
P07379	Phosphoenolpyruvate carboxykinase, cytosolic [GTP] OS=Rattus norvegicus OX=10116 GN=Pck1 PE=1 SV=1 - [PCKGC_R	69.4	0.000E0	4.773E-5	4.541E-5	0.000E0		133.31	127.98			5.47	7.40			2	3			4	4	
P07483	Fatty acid-binding protein, heart OS=Rattus norvegicus GN=Fabp3 PE=1 SV=2 - [FABPH_RAT]	14.8	3.334E-4	1.957E-4	1.825E-4	8.988E-5	278.40	254.80	127.76	62.29	46.62	53.38	30.08	19.55	6	7	4	3	13	12	8	4
P07632	Superoxide dismutase [Cu-Zn] OS=Rattus norvegicus GN=Sod1 PE=1 SV=2 - [SODC_RAT]	15.9	3.541E-3	1.898E-3	2.449E-3	1.737E-3	810.65	598.82	653.11	542.01	59.09	50.00	50.00	50.00	8	7	7	7	25	22	20	19
P07633	Propionyl-CoA carboxylase beta chain, mitochondrial OS=Rattus norvegicus OX=10116 GN=Pccb PE=2 SV=1 - [PCCB_RAT	58.6	0.000E0	1.445E-5	2.866E-5	3.084E-5		20.49	65.74	33.15		2.77	9.24	5.55		1	3	2		1	4	3
P07756	Carbamoyl-phosphate synthase [ammonia], mitochondrial OS=Rattus norvegicus GN=Cps1 PE=1 SV=1 - [CPSM_RAT]	164.5	2.476E-4	4.709E-4	8.066E-4	3.249E-4	707.98	2172.05	3783.71	1449.66	16.73	46.47	48.60	31.93	20	48	53	36	39	105	131	71
P07824	Arginase-1 OS=Rattus norvegicus GN=Arg1 PE=1 SV=2 - [ARGI1_RAT]	35.0	2.207E-5	0.000E0	0.000E0	0.000E0	30.16				2.17				1				1			
P07861	Neprilysin OS=Rattus norvegicus OX=10116 GN=Mme PE=1 SV=2 - [NEP_RAT]	85.7	0.000E0	2.292E-4	2.023E-4	2.146E-4		725.40	473.26	609.25		28.93	28.40	31.07		17	15	20		25	21	36
P07871	3-ketoacyl-CoA thiolase B, peroxisomal OS=Rattus norvegicus GN=Acaa1b PE=1 SV=2 - [THIKB_RAT]	43.8	0.000E0	8.764E-5	1.603E-4	9.302E-5		72.00	171.28	53.96		14.62	19.81	14.39		4	6	4		6	13	8
P07872	Peroxisomal acyl-coenzyme A oxidase 1 OS=Rattus norvegicus GN=Acox1 PE=1 SV=1 - [ACOX1_RAT]	74.6	0.000E0	1.998E-4	2.773E-4	1.813E-4		731.25	1012.73	669.79		33.89	42.36	37.37		13	17	14		26	32	28
P07895	Superoxide dismutase [Mn], mitochondrial OS=Rattus norvegicus GN=Sod2 PE=1 SV=2 - [SODM_RAT]	24.7	4.772E-4	2.971E-4	3.076E-4	2.657E-4	615.20	480.49	429.16	360.97	31.98	34.68	34.68	31.53	6	6	6	5	20	16	15	12
P07943	Aldose reductase OS=Rattus norvegicus GN=Akr1b1 PE=1 SV=3 - [ALDR_RAT]	35.8	9.436E-5	3.879E-4	5.017E-4	3.099E-4	164.08	567.45	578.72	382.95	12.66	54.75	57.91	39.87	4	15	15	12	8	34	36	25
P08009	Glutathione S-transferase Yb-3 OS=Rattus norvegicus GN=Gstm3 PE=1 SV=2 - [GSTM4_RAT]	25.7	7.667E-4	7.535E-4	7.734E-4	8.205E-4	951.98	798.70	774.59	631.39	68.35	38.53	38.53	45.41	16	9	9	9	36	23	21	20
P08010	Glutathione S-transferase Mu 2 OS=Rattus norvegicus GN=Gstm2 PE=1 SV=2 - [GSTM2_RAT]	25.7	7.667E-4	7.535E-4	7.734E-4	8.241E-4	1105.48	1480.35	1369.46	1289.51	76.15	77.06	73.85	75.69	18	19	17	16	39	43	39	43
P08011	Microsomal glutathione S-transferase 1 OS=Rattus norvegicus GN=Mgst1 PE=1 SV=3 - [MGST1_RAT]	17.5	0.000E0	5.285E-5	6.600E-5	5.145E-5		87.44	79.74	47.49		8.39	8.39	8.39		1	1	1		2	2	2
P08426	Cationic trypsin-3 OS=Rattus norvegicus OX=10116 GN=Try3 PE=2 SV=1 - [TRY3_RAT]	26.3	5.151E-4	3.373E-4	1.494E-4	5.223E-5	407.47	346.07	266.24	83.86	29.15	44.13	31.98	12.15	6	7	6	2	14	13	10	4
P08460	Nidogen-1 (Fragment) OS=Rattus norvegicus GN=Nid1 PE=1 SV=2 - [NID1_RAT]	35.6	1.875E-4	1.236E-4	2.679E-4	5.370E-5	157.86	152.35	269.26	96.85	24.38	21.30	30.56	10.49	6	5	7	3	11	9	13	5
P08461	Dihydrolipoyllysine-residue acetyltransferase component of pyruvate dehydrogenase complex, mitochondrial OS=Rattus nc	67.1	5.374E-5	0.000E0	0.000E0	0.000E0	54.09				4.75				2				3			
P08503	Medium-chain specific acyl-CoA dehydrogenase, mitochondrial OS=Rattus norvegicus GN=Acadm PE=1 SV=1 - [ACADM_R	46.5	0.000E0	2.479E-4	3.772E-4	1.807E-4		391.39	611.29	326.66		33.02	43.47	23.04		10	14	8		16	31	17
P08592	Amyloid beta A4 protein OS=Rattus norvegicus GN=App PE=1 SV=2 - [A4_RAT]	86.6	3.150E-5	0.000E0	0.000E0	0.000E0	61.00				1.56				1				2			
P08649	Complement C4 OS=Rattus norvegicus GN=C4 PE=1 SV=3 - [CO4_RAT]	192.0	1.335E-4	0.000E0	2.103E-5	0.000E0	336.48		58.61		8.23		4.72		10		4		19		4	
P08699	Galectin-3 OS=Rattus norvegicus GN=Lgals3 PE=1 SV=4 - [LEG3_RAT]	27.2	9.177E-4	2.101E-3	1.615E-3	2.571E-3	463.27	844.60	553.51	836.06	27.86	40.46	40.46	40.46	8	9	9	9	24	41	29	39
P08753	Guanine nucleotide-binding protein G(k) subunit alpha OS=Rattus norvegicus GN=Gnai3 PE=1 SV=3 - [GNAI3_RAT]	40.5	6.631E-5	9.296E-5	1.296E-4	9.351E-5	77.99	109.96	130.65	72.24	9.04	6.50	11.86	13.56	3	2	4	4	5	4	6	6
P08932	T-kininogen 2 OS=Rattus norvegicus PE=1 SV=1 - [KNT2_RAT]	47.7	1.357E-3	1.697E-3	2.113E-3	1.616E-3	1276.95	1444.99	2120.73	1667.82	53.26	56.98	66.05	66.28	19	20	23	24	52	65	92	84
P09006	Serine protease inhibitor A3N OS=Rattus norvegicus GN=Serpina3n PE=1 SV=3 - [SPA3N_RAT]	46.6	4.748E-3	4.243E-4	1.348E-3	7.014E-4	4027.05	1118.98	2576.00	1373.83	58.37	42.11	60.53	52.87	24	13	21	18	116	35	75	46
P09034	Argininosuccinate synthase OS=Rattus norvegicus GN=Ass1 PE=1 SV=1 - [ASSY_RAT]	46.5	2.168E-4	1.300E-4	7.366E-4	5.626E-4	371.40	277.16	1796.66	1124.25	19.90	32.77	55.34	41.75	8	11	22	17	15	20	69	44
P09117	Fructose-bisphosphate aldolase C OS=Rattus norvegicus GN=Aldoc PE=1 SV=3 - [ALDOC_RAT]	39.3	7.843E-4	8.656E-4	1.000E-3	1.157E-3	74.36	124.92	159.75	101.38	20.66	22.87	20.39	7.71	5	6	5	2	7	12	9	7
P09414	Nuclear factor 1 A-type OS=Rattus norvegicus OX=10116 GN=Nfia PE=1 SV=2 - [NFIA_RAT]	55.9	0.000E0	0.000E0	0.000E0	4.651E-5				46.36				2.16				1				2
P09456	cAMP-dependent protein kinase type I-alpha regulatory subunit OS=Rattus norvegicus GN=Prkar1a PE=2 SV=2 - [KAP0_R	43.1	0.000E0	1.338E-4	1.749E-4	1.267E-4		325.22	456.68	383.57		21.78	26.51	21.78		7	8	7		11	15	13
P09495	Tropomyosin alpha-4 chain OS=Rattus norvegicus GN=Tpm4 PE=1 SV=3 - [TPM4_RAT]	28.5	1.688E-3	2.084E-3	3.057E-3	2.140E-3	1302.84	1174.04	1450.89	1130.89	43.95	48.39	52.02	47.98	17	17	20	16	42	40	50	43
P09527	Ras-related protein Rab-7a OS=Rattus norvegicus GN=Rab7a PE=1 SV=2 - [RAB7A_RAT]	23.5	9.050E-4	4.250E-4	5.470E-4	2.863E-4	655.85	875.67	809.05	601.02	64.73	57.00	57.00	59.42	11	10	10	10	26	25	28	23
P09811	Glycogen phosphorylase, liver form OS=Rattus norvegicus GN=Pygl PE=1 SV=5 - [PYGL_RAT]	97.4	5.033E-5	8.335E-5	1.673E-4	1.707E-4	20.60	175.75	405.79	381.29	1.41	6.82	13.53	15.53	1	5	9	10	2	8	15	17
P09895	60S ribosomal protein L5 OS=Rattus norvegicus GN=Rpl5 PE=1 SV=3 - [RL5_RAT]	34.4	0.000E0	1.096E-3	1.023E-3	9.411E-4		1165.82	1228.40	890.29		43.77	43.77	43.77		15	14	14		56	46	38
P0C0A1	Vacuolar protein-sorting-associated protein 25 OS=Rattus norvegicus GN=Vps25 PE=1 SV=1 - [VPS25_RAT]	20.7	4.997E-5	4.520E-5	7.541E-5	0.000E0	94.40	45.68	52.52		14.20	14.20	8.52		2	2	1		3	3	2	
P0C0S7	Histone H2A.Z OS=Rattus norvegicus GN=H2afz PE=1 SV=2 - [H2AZ_RAT]	13.5	1.372E-3	1.734E-2	6.549E-3	2.035E-2	81.07	354.78	218.69	420.17	7.03	31.25	20.31	31.25	1	4	3	4	2	14	8	17
P0C219	Sarcolemmal membrane-associated protein OS=Rattus norvegicus GN=Slmap PE=3 SV=1 - [SLMAP_RAT]	98.2	9.821E-5	0.000E0	0.000E0	0.000E0	20.93				1.28				1				1			
P0C5E3	Palladin (Fragment) OS=Rattus norvegicus OX=10116 GN=Palld PE=1 SV=1 - [PALLD_RAT]	66.7	3.137E-5	1.196E-4	1.042E-4	5.538E-5	90.74	133.11	186.63	150.38	3.32	16.75	10.45	7.13	2	7	5	3	4	9	8	5
P0C5H9	Mesencephalic astrocyte-derived neurotrophic factor OS=Rattus norvegicus OX=10116 GN=Manf PE=1 SV=1 - [MANF_RA	20.4	1.890E-4	1.587E-4	0.000E0	1.266E-4	127.22	170.27		107.74	40.78	40.78		25.14	8	7		5	13	12		11
P10111	Peptidyl-prolyl cis-trans isomerase A OS=Rattus norvegicus GN=Ppia PE=1 SV=2 - [PPIA_RAT]	17.9	1.819E-2	1.395E-2	1.139E-2	9.744E-3	4592.79	4752.55	4676.13	4231.78	70.73	70.73	74.39	70.73	11	11	12	11	154	162	143	147
P10536	Ras-related protein Rab-1B OS=Rattus norvegicus GN=Rab1b PE=1 SV=1 - [RAB1B_RAT]	22.1	5.565E-4	4.619E-4	7.121E-4	4.882E-4	238.24	302.96	451.79	137.90	31.84	36.82	44.28	32.34	5	5	6	5	14	15	18	9
P10610	Cytochrome P450 2G1 OS=Rattus norvegicus GN=Cyp2g1 PE=2 SV=1 - [CP2G1_RAT]	56.7	3.197E-4	0.000E0	0.000E0	0.000E0	20.98				1.42				1				1			
P10719	ATP synthase subunit beta, mitochondrial OS=Rattus norvegicus GN=Atp5b PE=1 SV=2 - [ATPB_RAT]	56.3	0.000E0	9.636E-5	3.623E-4	6.642E-4		271.94	1119.26	1635.19		15.31	42.16	54.06		6	15	19		9	38	59
P10758	Lithostathine OS=Rattus norvegicus OX=10116 GN=Reg1 PE=1 SV=1 - [LITH_RAT]	18.7	1.250E-4	3.458E-4	1.186E-4	2.769E-4	233.91	259.63	175.96	280.28	33.33	33.33	25.45	33.33	4	4	3	4	8	10	6	10
P10760	Adenosylhomocysteinase OS=Rattus norvegicus GN=Ahcy PE=1 SV=3 - [SAHH_RAT]	47.5	3.203E-5	5.306E-4	7.450E-4	5.891E-4	108.47	963.02	1188.65	693.10	3.94	58.33	66.90	52.08	2	18	20	16	4	39	48	41
P10860	Glutamate dehydrogenase 1, mitochondrial OS=Rattus norvegicus GN=Glud1 PE=1 SV=2 - [DHE3_RAT]	61.4	0.000E0	6.931E-4	1.120E-3	6.859E-4		1192.06	1624.72	1072.66		45.16	51.97	44.98		19	22	21		43	60	45
P10867	L-gulonolactone oxidase OS=Rattus norvegicus GN=Gulo PE=1 SV=3 - [GGLO_RAT]	50.6	1.708E-3	2.252E-3	2.527E-3	9.632E-4	79.08	54.00	62.62	51.12	1.59	1.59	1.59	3.41	1	1	1	2	4	4	2	4
P10960	Sulfated glycoprotein 1 OS=Rattus norvegicus GN=Psap PE=1 SV=1 - [SAP_RAT]	61.1	2.243E-4	6.000E-5	8.796E-5	1.066E-4	211.56	38.75	57.75	93.09	10.83	5.96	4.51	3.79	6	3	2	2	9	3	2	3
P11232	Thioredoxin OS=Rattus norvegicus GN=Txn PE=1 SV=2 - [THIO_RAT]	11.7	8.060E-4	1.157E-3	7.978E-4	6.429E-4	574.34	675.35	771.24	471.63	77.14	77.14	77.14	43.81	6	7	7	4	18	21	23	14
P11348	Dihydropteridine reductase OS=Rattus norvegicus GN=Qdpr PE=1 SV=2 - [DHPR_RAT]	25.5	6.889E-4	4.072E-4	5.105E-4	3.786E-4	963.26	810.25	933.48	525.42	54.77	60.17	60.17	46.89	9	10	10	8	31	26	29	20
P11442	Clathrin heavy chain 1 OS=Rattus norvegicus GN=Cltc PE=1 SV=3 - [CLH1_RAT]	191.5	6.040E-4	1.060E-3	1.362E-3	7.260E-4	1951.00	5014.57	6902.68	3227.52	32.66	54.57	63.34	47.46	41	75	85	58	82	196	249	122
P11517	Hemoglobin subunit beta-2 OS=Rattus norvegicus PE=1 SV=2 - [HBB2_RAT]	16.0	5.561E-2	6.997E-2	7.146E-2	5.766E-2	5312.22	8795.27	9156.81	6087.41	89.80	89.80	89.80	89.80	15	14	17	14	260	448	459	327
P11598	Protein disulfide-isomerase A3 OS=Rattus norvegicus GN=Pdia3 PE=1 SV=2 - [PDIA3_RAT]	56.6	5.189E-4	1.999E-3	2.168E-3	1.469E-3	1302.26	3031.86	3055.32	2344.63	53.86	62.57	62.97	59.60	24	34	34	29	58	106	105	93
P11762	Galectin-1 OS=Rattus norvegicus GN=Lgals1 PE=1 SV=2 - [LEG1_RAT]	14.8	0.000E0	3.839E-4	5.779E-4	3.415E-4		337.98	761.56	397.23		58.52	65.93	52.59		8	8	7		16	22	16
P11884	Aldehyde dehydrogenase, mitochondrial OS=Rattus norvegicus GN=Aldh2 PE=1 SV=1 - [ALDH2_RAT]	56.5	2.625E-4	3.325E-4	3.160E-4	1.967E-4	55.59	796.46	705.03	316.97	1.54	32.76	41.62	31.41	1	13	15	12	2	27	27	20
P11915	Non-specific lipid-transfer protein OS=Rattus norvegicus GN=Scp2 PE=1 SV=3 - [NLTP_RAT]	58.8	4.579E-4	4.701E-4	4.828E-4	4.209E-4	183.44	480.45	412.55	395.44	12.25	16.27	14.44	16.27	6	9	7	9	12	19	15	18
P11980	Pyruvate kinase PKM OS=Rattus norvegicus GN=Pkm PE=1 SV=3 - [KPYM_RAT]	57.8	4.515E-5	5.400E-4	8.429E-4	5.617E-4	134.66	1310.17	2244.80	1369.74	6.03	50.28	60.26	57.82	3	21	27	24	5	46	74	54
P12001	60S ribosomal protein L18 OS=Rattus norvegicus OX=10116 GN=Rpl18 PE=1 SV=2 - [RL18_RAT]	21.6	0.000E0	1.186E-3	1.225E-3	1.024E-3		618.14	781.44	811.46		41.49	41.49	36.17		8	8	7		18	20	19
P12007	Isovaleryl-CoA dehydrogenase, mitochondrial OS=Rattus norvegicus GN=Ivd PE=1 SV=2 - [IVD_RAT]	46.4	1.589E-5	9.633E-5	1.358E-4	5.157E-5	26.41	206.16	325.33	130.86	5.19	13.21	19.81	10.61	2	5	7	4	2	8	11	6
P12336	Solute carrier family 2, facilitated glucose transporter member 2 OS=Rattus norvegicus OX=10116 GN=Slc2a2 PE=2 SV=1	57.0	3.740E-5	0.000E0	0.000E0	0.000E0	29.33				2.30				1				1			
P12346	Serotransferrin OS=Rattus norvegicus GN=Tf PE=1 SV=3 - [TRFE_RAT]	76.3	5.900E-3	3.033E-3	4.098E-3	3.947E-3	5771.87	4614.16	5516.12	5032.83	66.48	62.46	70.49	62.46	42	38	40	38	167	140	159	157
P12368	cAMP-dependent protein kinase type II-alpha regulatory subunit OS=Rattus norvegicus OX=10116 GN=Prkar2a PE=1 SV=	45.5	0.000E0	1.982E-4	1.980E-4	1.236E-4		357.84	419.21	265.60		23.94	33.42	16.21		8	10	5		14	18	10
P12711	Alcohol dehydrogenase class-3 OS=Rattus norvegicus GN=Adh5 PE=1 SV=3 - [ADHX_RAT]	39.6	4.746E-4	3.632E-4	4.500E-4	3.808E-4	721.48	846.16	1069.20	964.70	48.66	45.19	65.51	46.79	15	13	16	15	36	31	37	34
P12749	60S ribosomal protein L26 OS=Rattus norvegicus GN=Rpl26 PE=1 SV=1 - [RL26_RAT]	17.3	0.000E0	1.092E-4	1.781E-4	2.743E-4		25.55	68.59	110.61		4.83	10.34	22.76		1	2	4		2	4	6
P12785	Fatty acid synthase OS=Rattus norvegicus GN=Fasn PE=1 SV=3 - [FAS_RAT]	272.5	0.000E0	1.429E-4	3.995E-4	3.138E-4		427.65	970.44	368.47		8.30	21.72	9.54		16	38	18		24	59	27
P12788	Trypsin-4 OS=Rattus norvegicus OX=10116 GN=Try4 PE=2 SV=1 - [TRY4_RAT]	26.6	4.867E-5	0.000E0	0.000E0	0.000E0	28.82				11.34				2				2			
P12839	Neurofilament medium polypeptide OS=Rattus norvegicus OX=10116 GN=Nefm PE=1 SV=4 - [NFM_RAT]	95.7	7.241E-4	5.130E-4	4.700E-4	6.507E-4	57.67	55.88	34.02	56.45	1.06	2.25	2.01	1.06	1	2	2	1	2	3	2	2
P12928	Pyruvate kinase PKLR OS=Rattus norvegicus GN=Pklr PE=2 SV=1 - [KPYR_RAT]	62.2	4.005E-4	4.297E-4	5.543E-4	3.180E-4	285.35	530.05	725.42	438.24	9.93	42.86	44.25	47.56	4	17	19	19	10	29	36	32
P13053	Vitamin D3 receptor OS=Rattus norvegicus OX=10116 GN=Vdr PE=1 SV=1 - [VDR_RAT]	47.8	0.000E0	9.953E-6	0.000E0	0.000E0		0.00				1.65				1				1		
P13084	Nucleophosmin OS=Rattus norvegicus GN=Npm1 PE=1 SV=1 - [NPM_RAT]	32.5	7.829E-4	1.637E-3	1.288E-3	4.559E-4	874.84	2027.78	1892.50	1095.84	23.97	36.99	44.86	34.25	5	9	9	9	29	61	53	38
P13086	Succinyl-CoA ligase [ADP/GDP-forming] subunit alpha, mitochondrial OS=Rattus norvegicus GN=Suclg1 PE=2 SV=2 - [SUC	36.1	0.000E0	1.451E-4	1.978E-4	1.429E-4		166.70	189.31	138.27		17.05	17.05	17.05		4	4	4		9	8	8
P13221	Aspartate aminotransferase, cytoplasmic OS=Rattus norvegicus GN=Got1 PE=1 SV=3 - [AATC_RAT]	46.4	2.303E-5	1.987E-4	2.354E-4	2.235E-4	48.52	591.28	603.07	381.83	8.47	35.11	36.56	37.29	2	9	9	10	3	17	19	17

P13264	Glutaminase kidney isoform, mitochondrial OS=Rattus norvegicus GN=Gls PE=1 SV=2 - [GLSK_RAT]	74.0	0.000E0	0.000E0	3.640E-5	0.000E0		25.27	125.71			2.52	12.61			1	5			1	7	
P13383	Nucleolin OS=Rattus norvegicus GN=Ncl PE=1 SV=3 - [NUCL_RAT]	77.1	1.166E-3	3.287E-3	2.610E-3	3.550E-3	1925.38	3390.09	3093.44	3391.99	31.56	36.04	35.62	38.43	21	26	26	29	61	95	86	103
P13437	3-ketoacyl-CoA thiolase, mitochondrial OS=Rattus norvegicus OX=10116 GN=Acaa2 PE=2 SV=1 - [THIM_RAT]	41.8	4.620E-4	7.713E-4	1.218E-3	7.726E-4	783.54	1413.81	1922.76	1237.28	57.93	65.74	65.49	63.48	16	20	19	16	29	53	66	48
P13471	40S ribosomal protein S14 OS=Rattus norvegicus GN=Rps14 PE=2 SV=3 - [RS14_RAT]	16.2	0.000E0	7.483E-5	2.446E-4	2.745E-4		53.22	224.36	242.57		8.61	29.80	31.13		1	3	4		2	5	8
P13596	Neural cell adhesion molecule 1 OS=Rattus norvegicus GN=Ncam1 PE=1 SV=1 - [NCAM1_RAT]	94.6	1.483E-4	7.793E-5	5.727E-5	5.492E-5	161.92	91.74	58.89	39.83	6.76	5.36	4.43	3.38	4	4	3	2	7	4	3	3
P13635	Ceruloplasmin OS=Rattus norvegicus GN=Cp PE=1 SV=3 - [CERU_RAT]	120.8	1.695E-4	1.007E-4	1.135E-4	1.056E-4	318.96	198.52	275.18	171.18	10.39	8.12	15.86	9.82	8	7	11	8	18	10	16	12
P13668	Stathmin OS=Rattus norvegicus GN=Stmn1 PE=1 SV=2 - [STMN1_RAT]	17.3	5.784E-5	5.716E-5	4.953E-5	0.000E0	41.32	25.41	26.75		6.04	6.04	5.37		1	1	1		2	2	1	
P13676	Acylamino-acid-releasing enzyme OS=Rattus norvegicus GN=Apeh PE=1 SV=1 - [ACPH_RAT]	81.3	0.000E0	1.630E-4	2.096E-4	1.308E-4		366.84	511.19	330.51		21.31	44.54	18.31		10	17	8		15	24	15
P13697	NADP-dependent malic enzyme OS=Rattus norvegicus GN=Me1 PE=1 SV=2 - [MAOX_RAT]	64.0	0.000E0	1.154E-4	1.570E-4	1.050E-4		365.93	573.93	143.19		33.04	37.24	24.13		13	14	8		21	25	11
P13721	Beta-galactoside alpha-2,6-sialyltransferase 1 OS=Rattus norvegicus OX=10116 GN=St6gal1 PE=1 SV=1 - [SIAT1_RAT]	46.7	1.515E-5	4.420E-5	4.121E-5	0.000E0	23.07	101.24	95.96	33.24	2.73	9.43	5.46	1.74	1	3	2	1	2	5	3	1
P13803	Electron transfer flavoprotein subunit alpha, mitochondrial OS=Rattus norvegicus GN=Etfa PE=1 SV=4 - [ETFA_RAT]	34.9	0.000E0	5.832E-4	7.734E-4	5.323E-4		729.80	1019.41	519.76		53.45	64.26	48.05		12	15	11		28	39	26
P13832	Myosin regulatory light chain RLC-A OS=Rattus norvegicus GN=Rlc-a PE=2 SV=2 - [MRLCA_RAT]	19.9	0.000E0	3.635E-4	3.366E-4	3.903E-4		501.22	436.80	594.28		41.28	41.28	62.21		6	6	8		16	13	20
P13941	Collagen alpha-1(III) chain OS=Rattus norvegicus GN=Col3a1 PE=2 SV=3 - [CO3A1_RAT]	138.9	5.756E-5	3.969E-5	4.923E-5	0.000E0	117.34	121.97	51.93		2.32	2.39	2.67		3	3	3		5	4	3	
P14046	Alpha-1-inhibitor 3 OS=Rattus norvegicus GN=A1i3 PE=1 SV=1 - [A1I3_RAT]	163.7	9.833E-3	1.911E-4	2.796E-4	1.368E-4	2989.92	212.37	415.91	175.44	20.24	6.57	14.29	6.50	26	8	15	8	151	15	25	13
P14173	Aromatic-L-amino-acid decarboxylase OS=Rattus norvegicus OX=10116 GN=Ddc PE=1 SV=1 - [DDC_RAT]	54.0	0.000E0	0.000E0	1.999E-5	0.000E0		20.83	55.74			1.46	5.42			1	2			1	3	
P14408	Fumarate hydratase, mitochondrial OS=Rattus norvegicus GN=Fh PE=1 SV=1 - [FUMH_RAT]	54.4	0.000E0	3.157E-4	3.640E-4	2.367E-4		979.45	879.60	657.40		43.00	46.55	32.94		15	16	11		34	30	24
P14480	Fibrinogen beta chain OS=Rattus norvegicus GN=Fgb PE=1 SV=4 - [FIBB_RAT]	54.2	6.205E-4	5.096E-4	6.014E-4	3.895E-4	1006.90	1150.88	1279.20	815.60	45.93	47.18	53.03	50.10	15	17	20	16	46	49	54	35
P14562	Lysosome-associated membrane glycoprotein 1 OS=Rattus norvegicus GN=Lamp1 PE=1 SV=1 - [LAMP1_RAT]	43.9	5.456E-4	3.332E-4	4.336E-4	3.457E-4	559.27	591.33	724.09	640.37	14.74	17.44	14.74	17.44	6	7	6	7	15	16	16	16
P14604	Enoyl-CoA hydratase, mitochondrial OS=Rattus norvegicus GN=Echs1 PE=1 SV=1 - [ECHM_RAT]	31.5	0.000E0	1.436E-4	2.323E-4	1.097E-4		243.32	469.62	85.86		27.93	31.03	14.83		6	7	3		13	22	4
P14659	Heat shock-related 70 kDa protein 2 OS=Rattus norvegicus GN=Hspa2 PE=1 SV=2 - [HSP72_RAT]	69.6	8.603E-3	5.014E-3	5.076E-3	4.302E-3	2028.88	1808.49	1913.55	1648.34	27.33	27.80	28.12	26.38	16	19	17	16	56	51	58	47
P14668	Annexin A5 OS=Rattus norvegicus GN=Anxa5 PE=1 SV=3 - [ANXA5_RAT]	35.7	9.236E-4	4.316E-4	4.148E-4	3.293E-4	1273.62	652.60	726.21	398.72	61.13	43.26	43.26	33.86	18	12	12	9	43	23	24	16
P14669	Annexin A3 OS=Rattus norvegicus GN=Anxa3 PE=1 SV=4 - [ANXA3_RAT]	36.3	5.768E-5	7.134E-5	1.138E-4	6.347E-5	65.80	172.04	350.00	137.41	2.78	24.07	29.01	10.80	1	7	10	4	2	12	18	9
P14740	Dipeptidyl peptidase 4 OS=Rattus norvegicus GN=Dpp4 PE=1 SV=2 - [DPP4_RAT]	88.0	2.497E-4	5.205E-4	6.453E-4	5.631E-4	530.02	1096.35	1253.51	1134.38	20.08	34.16	45.37	32.72	15	23	30	24	27	46	63	53
P14841	Cystatin-C OS=Rattus norvegicus GN=Cst3 PE=1 SV=2 - [CYTC_RAT]	15.4	4.054E-5	0.000E0	0.000E0	0.000E0	105.95				20.00				2				4			
P14942	Glutathione S-transferase alpha-4 OS=Rattus norvegicus GN=Gsta4 PE=1 SV=2 - [GSTA4_RAT]	25.5	3.596E-3	1.324E-3	1.385E-3	1.626E-3	1679.00	669.85	1042.90	807.88	58.11	54.95	54.95	58.11	12	11	11	12	72	40	49	43
P15083	Polymeric immunoglobulin receptor OS=Rattus norvegicus GN=Pigr PE=1 SV=1 - [PIGR_RAT]	84.7	6.074E-4	0.000E0	0.000E0	0.000E0	1133.67				20.03				14				39			
P15178	Aspartate--tRNA ligase, cytoplasmic OS=Rattus norvegicus GN=Dars PE=2 SV=1 - [SYDC_RAT]	57.1	1.902E-5	8.105E-5	1.315E-4	1.110E-4	26.44	337.23	454.27	367.27	3.99	20.56	30.54	34.73	2	9	13	14	2	15	20	22
P15205	Microtubule-associated protein 1B OS=Rattus norvegicus OX=10116 GN=Map1b PE=1 SV=3 - [MAP1B_RAT]	269.5	3.009E-6	0.000E0	0.000E0	1.508E-4	0.00			22.10	0.33			0.33	1			1	1			1
P15650	Long-chain specific acyl-CoA dehydrogenase, mitochondrial OS=Rattus norvegicus GN=Acadl PE=1 SV=1 - [ACADL_RAT]	47.8	1.348E-3	7.056E-4	9.558E-4	5.814E-4	1887.79	1109.54	1894.24	955.64	50.70	46.98	50.70	48.14	19	17	19	18	64	45	57	39
P15651	Short-chain specific acyl-CoA dehydrogenase, mitochondrial OS=Rattus norvegicus GN=Acads PE=1 SV=2 - [ACADS_RAT]	44.7	1.027E-4	1.934E-4	2.259E-4	1.505E-4	416.39	484.48	665.22	472.71	39.08	34.71	44.17	47.82	10	9	11	12	17	20	21	20
P15684	Aminopeptidase N OS=Rattus norvegicus OX=10116 GN=Anpep PE=1 SV=2 - [AMPN_RAT]	109.4	2.472E-3	1.047E-3	1.351E-3	1.630E-3	5938.96	3854.24	4921.07	4617.46	49.22	39.38	47.05	47.05	45	31	38	38	158	106	125	137
P15693	Intestinal-type alkaline phosphatase 1 OS=Rattus norvegicus OX=10116 GN=Alpi PE=1 SV=1 - [PPBI1_RAT]	58.4	6.086E-3	1.123E-3	2.162E-3	2.004E-3	3950.00	1768.30	3083.42	2153.85	55.19	46.48	55.19	53.70	21	16	19	19	131	65	106	92
P15791	Calcium/calmodulin-dependent protein kinase type II subunit delta OS=Rattus norvegicus GN=Camk2d PE=1 SV=1 - [KCC	60.0	0.000E0	9.654E-5	8.153E-5	8.941E-5		61.74	97.67	38.14		8.63	7.13	5.82		4	3	2		7	5	3
P15800	Laminin subunit beta-2 OS=Rattus norvegicus GN=Lamb2 PE=2 SV=1 - [LAMB2_RAT]	196.3	5.947E-5	0.000E0	0.000E0	0.000E0	25.28				0.39				1				1			
P15865	Histone H1.4 OS=Rattus norvegicus GN=Hist1h1e PE=1 SV=3 - [H14_RAT]	22.0	9.084E-5	1.964E-3	8.129E-4	3.287E-3	74.66	631.65	330.85	628.07	5.02	26.94	19.63	19.63	1	7	6	6	2	20	11	18
P15999	ATP synthase subunit alpha, mitochondrial OS=Rattus norvegicus GN=Atp5a1 PE=1 SV=2 - [ATPA_RAT]	59.7	0.000E0	1.376E-4	2.751E-4	5.662E-4		307.25	494.47	844.78		14.47	23.15	37.25		8	11	16		14	23	34
P16036	Phosphate carrier protein, mitochondrial OS=Rattus norvegicus OX=10116 GN=Slc25a3 PE=1 SV=1 - [MPCP_RAT]	39.4	0.000E0	4.152E-4	0.000E0	4.323E-4		21.74		42.43		6.18		6.18		2		2		2		3
P16086	Spectrin alpha chain, non-erythrocytic 1 OS=Rattus norvegicus GN=Sptan1 PE=1 SV=2 - [SPTN1_RAT]	284.5	1.229E-3	8.350E-4	7.804E-4	4.655E-4	9416.24	7832.75	7081.30	4193.39	61.57	58.21	58.45	45.71	144	123	118	89	345	276	246	176
P16391	RT1 class I histocompatibility antigen, AA alpha chain OS=Rattus norvegicus PE=1 SV=2 - [HA12_RAT]	41.8	4.935E-5	6.015E-5	7.044E-5	0.000E0	125.79	150.23	137.02		9.97	12.40	7.28		3	4	2		4	6	3	
P16446	Phosphatidylinositol transfer protein alpha isoform OS=Rattus norvegicus GN=Pitpna PE=1 SV=2 - [PIPNA_RAT]	31.9	0.000E0	1.830E-4	2.128E-4	2.323E-4		322.70	267.71	270.02		39.48	38.75	39.48		11	10	11		21	15	17
P16573	Carcinoembryonic antigen-related cell adhesion molecule 1 OS=Rattus norvegicus OX=10116 GN=Ceacam1 PE=1 SV=4 -	57.4	1.334E-3	1.917E-4	2.799E-4	2.200E-4	1053.64	376.98	567.62	342.39	23.70	15.22	20.62	21.00	10	6	8	8	33	11	14	12
P16617	Phosphoglycerate kinase 1 OS=Rattus norvegicus GN=Pgk1 PE=1 SV=2 - [PGK1_RAT]	44.5	9.368E-5	2.006E-3	2.567E-3	2.227E-3	70.08	2263.88	2626.18	1655.97	14.39	70.26	69.30	71.46	4	25	24	24	7	82	84	72
P16638	ATP-citrate synthase OS=Rattus norvegicus GN=Acly PE=1 SV=1 - [ACLY_RAT]	120.6	0.000E0	1.315E-4	6.470E-4	7.051E-4		466.21	1017.11	582.96		13.91	19.36	17.18		13	18	15		25	41	31
P16975	SPARC OS=Rattus norvegicus GN=Sparc PE=1 SV=4 - [SPRC_RAT]	34.3	1.199E-4	5.171E-5	4.313E-5	2.365E-5	259.71	108.43	156.62	29.45	20.27	15.61	20.60	8.64	6	4	4	2	13	6	7	2
P17046	Lysosome-associated membrane glycoprotein 2 OS=Rattus norvegicus OX=10116 GN=Lamp2 PE=1 SV=3 - [LAMP2_RAT]	45.1	5.743E-4	1.382E-4	3.290E-4	2.829E-4	295.74	155.56	256.97	108.93	13.38	9.49	9.49	9.49	5	3	3	3	15	8	12	7
P17074	40S ribosomal protein S19 OS=Rattus norvegicus GN=Rps19 PE=2 SV=3 - [RS19_RAT]	16.1	0.000E0	4.371E-5	2.073E-4	2.485E-4		242.94	428.58	464.22		28.97	37.93	44.83		4	7	8		8	13	15
P17077	60S ribosomal protein L9 OS=Rattus norvegicus GN=Rpl9 PE=1 SV=1 - [RL9_RAT]	21.9	0.000E0	2.720E-4	4.656E-4	3.921E-4		452.81	744.19	502.63		60.42	60.94	60.42		8	10	8		18	26	22
P17078	60S ribosomal protein L35 OS=Rattus norvegicus OX=10116 GN=Rpl35 PE=1 SV=3 - [RL35_RAT]	14.5	0.000E0	0.000E0	1.341E-4	2.704E-4			133.49	172.23			18.70	18.70			3	3			5	6
P17136	Small nuclear ribonucleoprotein-associated protein B OS=Rattus norvegicus OX=10116 GN=Snrpb PE=2 SV=2 - [RSMB_RA	23.6	1.650E-5	2.246E-4	2.200E-4	2.048E-4	35.14	190.66	221.13	193.01	3.46	19.05	12.55	12.55	1	4	3	3	2	8	9	7
P17164	Tissue alpha-L-fucosidase OS=Rattus norvegicus GN=Fuca1 PE=1 SV=1 - [FUCO_RAT]	53.5	1.057E-4	1.726E-4	2.748E-4	3.234E-4	91.63	243.11	244.54	314.11	6.06	9.74	18.61	20.78	2	4	6	7	4	8	10	14
P17220	Proteasome subunit alpha type-2 OS=Rattus norvegicus GN=Psma2 PE=1 SV=3 - [PSA2_RAT]	25.9	0.000E0	2.334E-4	4.224E-4	2.126E-4		372.16	744.09	408.11		44.87	50.00	49.57		8	11	10		17	26	21
P17425	Hydroxymethylglutaryl-CoA synthase, cytoplasmic OS=Rattus norvegicus GN=Hmgcs1 PE=1 SV=1 - [HMCS1_RAT]	57.4	0.000E0	1.870E-4	1.924E-4	5.190E-5		272.03	222.31	49.44		20.96	12.50	6.35		8	6	3		13	10	5
P17475	Alpha-1-antiproteinase OS=Rattus norvegicus GN=Serpina1 PE=1 SV=2 - [A1AT_RAT]	46.1	4.159E-2	2.381E-3	6.434E-3	3.981E-3	4777.51	1879.00	2658.86	2339.47	43.07	34.55	41.61	41.12	19	14	18	16	134	52	75	63
P17702	60S ribosomal protein L28 OS=Rattus norvegicus GN=Rpl28 PE=1 SV=4 - [RL28_RAT]	15.8	0.000E0	4.924E-5	4.725E-5	9.601E-5		39.18	164.63	138.91		5.84	25.55	25.55		1	4	4		2	7	7
P17764	Acetyl-CoA acetyltransferase, mitochondrial OS=Rattus norvegicus GN=Acat1 PE=1 SV=1 - [THIL_RAT]	44.7	0.000E0	3.188E-4	5.440E-4	3.958E-4		807.90	944.28	545.85		50.94	41.27	47.64		13	12	13		36	41	30
P18163	Long-chain-fatty-acid--CoA ligase 1 OS=Rattus norvegicus OX=10116 GN=Acsl1 PE=1 SV=1 - [ACSL1_RAT]	78.1	5.138E-5	0.000E0	0.000E0	0.000E0	114.67				7.15				4				5			
P18292	Prothrombin OS=Rattus norvegicus GN=F2 PE=1 SV=1 - [THRB_RAT]	70.4	0.000E0	3.784E-5	4.406E-5	0.000E0		120.63	76.86			5.83	6.16			2	2			3	4	
P18297	Sepiapterin reductase OS=Rattus norvegicus GN=Spr PE=1 SV=1 - [SPRE_RAT]	28.1	0.000E0	6.742E-5	1.117E-4	9.457E-5		243.02	317.19	215.41		18.70	24.43	21.37		3	4	4		6	7	8
P18298	S-adenosylmethionine synthase isoform type-2 OS=Rattus norvegicus GN=Mat2a PE=1 SV=1 - [METK2_RAT]	43.7	1.552E-4	3.220E-4	3.077E-4	2.833E-4	193.44	450.89	383.40	329.17	13.67	33.42	25.57	22.53	5	11	8	7	8	18	18	13
P18395	Cold shock domain-containing protein E1 OS=Rattus norvegicus OX=10116 GN=Csde1 PE=2 SV=1 - [CSDE1_RAT]	88.8	6.730E-6	0.000E0	0.000E0	1.238E-5	23.15			23.84	0.88			1.00	1			1	1			1
P18418	Calreticulin OS=Rattus norvegicus GN=Calr PE=1 SV=1 - [CALR_RAT]	48.0	8.535E-3	5.395E-3	5.104E-3	3.729E-3	4014.16	3546.96	2917.65	2255.78	65.14	59.62	65.14	63.46	27	26	25	22	173	152	131	98
P18420	Proteasome subunit alpha type-1 OS=Rattus norvegicus GN=Psma1 PE=1 SV=2 - [PSA1_RAT]	29.5	0.000E0	4.873E-4	6.945E-4	4.179E-4		583.62	851.41	352.78		57.79	66.92	55.89		12	15	12		23	34	20
P18421	Proteasome subunit beta type-1 OS=Rattus norvegicus GN=Psmb1 PE=1 SV=3 - [PSB1_RAT]	26.5	0.000E0	1.676E-4	3.630E-4	1.595E-4		210.57	473.60	200.40		27.92	39.17	27.92		5	7	5		8	20	8
P18422	Proteasome subunit alpha type-3 OS=Rattus norvegicus GN=Psma3 PE=1 SV=3 - [PSA3_RAT]	28.4	2.245E-5	2.705E-4	3.766E-4	2.531E-4	68.11	448.32	659.17	424.55	14.12	38.43	41.57	38.82	3	10	11	9	3	18	25	18
P18445	60S ribosomal protein L27a OS=Rattus norvegicus OX=10116 GN=Rpl27a PE=1 SV=3 - [RL27A_RAT]	16.6	0.000E0	1.129E-4	2.238E-4	4.466E-4		115.28	130.42	186.15		15.54	20.95	27.70		2	3	4		3	4	8
P18484	AP-2 complex subunit alpha-2 OS=Rattus norvegicus GN=Ap2a2 PE=1 SV=3 - [AP2A2_RAT]	104.0	0.000E0	8.858E-5	1.095E-4	6.779E-5		246.63	317.88	103.29		13.11	14.82	8.96		9	11	5		15	19	6
P18614	Integrin alpha-1 OS=Rattus norvegicus GN=Itga1 PE=1 SV=1 - [ITA1_RAT]	130.7	4.258E-5	3.273E-5	2.091E-5	1.540E-5	196.19	49.85	45.44	24.14	3.39	1.86	0.76	0.76	4	2	1	1	7	3	2	2
P18645	UDP-glucose 4-epimerase OS=Rattus norvegicus GN=Gale PE=2 SV=1 - [GALE_RAT]	38.2	0.000E0	0.000E0	0.000E0	3.016E-5				41.58				3.46				1				2
P18757	Cystathionine gamma-lyase OS=Rattus norvegicus GN=Cth PE=1 SV=2 - [CGL_RAT]	43.6	0.000E0	6.594E-5	7.796E-5	5.751E-5		154.10	200.54	139.48		13.57	17.09	19.85		4	5	6		8	8	9
P18886	Carnitine O-palmitoyltransferase 2, mitochondrial OS=Rattus norvegicus OX=10116 GN=Cpt2 PE=1 SV=1 - [CPT2_RAT]	74.1	0.000E0	0.000E0	6.168E-5	3.726E-5			82.20	24.42			13.68	3.65			5	2			7	2
P19132	Ferritin heavy chain OS=Rattus norvegicus GN=Fth1 PE=1 SV=3 - [FRIH_RAT]	21.1	4.195E-3	2.660E-3	4.090E-3	3.574E-3	1089.55	1137.47	1334.92	985.21	57.69	57.69	57.69	57.69	13	13	12	13	45	41	54	49
P19139	Casein kinase II subunit alpha OS=Rattus norvegicus GN=Csnk2a1 PE=1 SV=2 - [CSK21_RAT]	45.0	0.000E0	5.665E-4	2.497E-4	1.865E-4		141.61	230.01	118.48		35.04	31.97	24.04		8	7	6		15	13	9
P19218	Pancreatic secretory granule membrane major glycoprotein GP2 OS=Rattus norvegicus OX=10116 GN=Gp2 PE=1 SV=1 -	58.7	0.000E0	2.902E-5	5.015E-5	0.000E0		25.62	81.92			6.60	10.38			3	4			3	6	
P19222	Carboxypeptidase A2 OS=Rattus norvegicus OX=10116 GN=Cpa2 PE=1 SV=1 - [CBPA2_RAT]	46.9	6.011E-3	7.526E-4	6.284E-4	1.021E-3	1684.81	666.41	662.58	646.52	51.56	37.41	45.80	45.08	14	10	11	11	66	24	24	30
P19223	Carboxypeptidase B OS=Rattus norvegicus GN=Cpb1 PE=1 SV=1 - [CBPB1_RAT]	47.5	1.987E-3	1.960E-4	1.462E-4	1.857E-4	1499.17	509.91	371.89	460.14	47.95	25.30	23.61	26.75	15	9	8	9	53	18	16	19
P19234	NADH dehydrogenase [ubiquinone] flavoprotein 2, mitochondrial OS=Rattus norvegicus OX=10116 GN=Ndufv2 PE=1 SV=	27.4	3.613E-5	3.338E-5	4.249E-5	7.843E-5	55.73	113.42	167.11	161.75	4.03	13.71	13.71	13.71	1	3	3	3	1	6	5	6
P19356	Porphobilinogen deaminase OS=Rattus norvegicus GN=Hmbs PE=1 SV=2 - [HEM3_RAT]	39.3	5.586E-5	3.887E-5	5.149E-5	4.489E-5	198.07	95.21	117.45	38.20	19.11	13.85	16.90	16.90	5	4	5	3	10	7	8	4
P19468	Glutamate--cysteine ligase catalytic subunit OS=Rattus norvegicus GN=Gclc PE=1 SV=2 - [GSH1_RAT]	72.6	1.815E-5	8.418E-5	8.924E-5	6.758E-5	32.64	256.73	233.45	143.50	1.41	18.68	19.94	8.95	1	9	10	5	1	15	15	7
P19643	Amine oxidase [flavin-containing] B OS=Rattus norvegicus OX=10116 GN=Maob PE=1 SV=3 - [AOFB_RAT]	58.4	0.000E0	0.000E0	0.000E0	0.000E0				20.20				2.88				1				1
P19804	Nucleoside diphosphate kinase B OS=Rattus norvegicus GN=Nme2 PE=1 SV=1 - [NDKB_RAT]	17.3	7.943E-4	2.143E-3	2.484E-3	1.865E-3	471.17	1075.67	1126.53	654.47	74.34	79.61	78.95	78.95	9	11	11	10	26	44	52	34
P19944	60S acidic ribosomal protein P1 OS=Rattus norvegicus GN=Rplp1 PE=3 SV=1 - [RLA1_RAT]	11.5	9.421E-5	4.410E-4	4.047E-4	0.000E0	32.96	57.99	60.17		14.04	14.04	14.04		1	1	1		1	4	3	
P19945	60S acidic ribosomal protein P0 OS=Rattus norvegicus GN=Rplp0 PE=1 SV=2 - [RLA0_RAT]	34.2	9.554E-5	1.051E-3	1.075E-3	9.630E-4	291.73	888.15	1025.86	848.11	33.44	43.85	43.85	43.85	8	10	10	10	14	29	33	30
P20059	Hemopexin OS=Rattus norvegicus GN=Hpx PE=1 SV=3 - [HEMO_RAT]	51.3	2.917E-4	1.699E-4	4.179E-5	2.459E-5	524.25	296.87	106.07	27.15	27.39	24.13	11.09	4.35	11	8	4	2	23	12	6	3
P20069	Mitochondrial-processing peptidase subunit alpha OS=Rattus norvegicus OX=10116 GN=Pmpca PE=1 SV=1 - [MPPA_RAT	58.6	0.000E0	8.779E-5	1.045E-4	5.760E-5		204.60	330.90	123.33		9.92	14.31	9.92		3	5	3		8	10	5
P20070	NADH-cytochrome b5 reductase 3 OS=Rattus norvegicus OX=10116 GN=Cyb5r3 PE=1 SV=2 - [NB5R3_RAT]	34.2	3.768E-5	0.000E0	0.000E0	0.000E0	28.21				8.31				2				4			
P20280	60S ribosomal protein L21 OS=Rattus norvegicus GN=Rpl21 PE=1 SV=3 - [RL21_RAT]	18.5	0.000E0	0.000E0	3.087E-4	2.376E-4			55.18	90.54			9.38	16.25			1	2			2	3
P20650	Protein phosphatase 1A OS=Rattus norvegicus GN=Ppm1a PE=1 SV=1 - [PPM1A_RAT]	42.4	0.000E0	0.000E0	0.000E0	2.237E-5				32.72				3.14				1				2
P20651	Serine/threonine-protein phosphatase 2B catalytic subunit beta isoform OS=Rattus norvegicus GN=Ppp3cb PE=1 SV=1 - [	59.1	1.039E-4	8.176E-5	7.787E-5	6.151E-5	103.19	94.90	92.66	94.79	2.29	4.19	5.90	5.71	1	2	3	3	2	4	5	5
P20673	Argininosuccinate lyase OS=Rattus norvegicus GN=Asl PE=2 SV=1 - [ARLY_RAT]	51.5	7.914E-5	5.676E-5	1.983E-4	1.807E-4	144.82	49.34	449.00	386.52	11.93	1.95	26.03	27.77	4	1	9	11	7	2	18	19
P20717	Protein-arginine deiminase type-2 OS=Rattus norvegicus GN=Padi2 PE=1 SV=1 - [PADI2_RAT]	75.3	1.550E-4	0.000E0	9.149E-5	0.000E0	56.27		27.22		1.20		1.20		1		1		2		1	
P20759	Ig gamma-1 chain C region OS=Rattus norvegicus PE=1 SV=1 - [IGHG1_RAT]	35.9	6.514E-4	1.750E-4	1.609E-4	1.332E-4	456.93	192.17	189.41	93.14	20.55	6.75	6.75	3.07	5	2	2	1	16	4	6	2
P20760	Ig gamma-2A chain C region OS=Rattus norvegicus GN=Igg-2a PE=1 SV=1 - [IGG2A_RAT]	35.2	1.768E-3	5.601E-4	4.950E-4	3.482E-4	1234.16	673.55	770.51	334.12	59.94	40.06	43.48	27.95	15	10	11	7	48	25	30	15
P20761	Ig gamma-2B chain C region OS=Rattus norvegicus GN=Igh-1a PE=1 SV=1 - [IGG2B_RAT]	36.5	5.861E-4	2.430E-4	5.213E-4	3.512E-4	169.72	99.26	207.07	190.38	24.92	7.81	21.92	16.22	5	2	5	4	12	4	12	9
P20767	Ig lambda-2 chain C region OS=Rattus norvegicus PE=4 SV=1 - [LAC2_RAT]	11.3	4.707E-4	5.246E-5	5.723E-5	0.000E0	163.12	59.46	77.48		76.92	31.73	31.73		5	2	2		12	3	3	
P20788	Cytochrome b-c1 complex subunit Rieske, mitochondrial OS=Rattus norvegicus OX=10116 GN=Uqcrfs1 PE=1 SV=2 - [UCF	29.4	5.204E-5	0.000E0	0.000E0	0.000E0	165.83				10.58				3				5			
P21139	Alpha-mannosidase 2C1 OS=Rattus norvegicus GN=Man2c1 PE=1 SV=1 - [MA2C1_RAT]	115.9	0.000E0	7.721E-6	4.339E-5	0.000E0		25.93	37.30			1.44	5.96			1	4			1	4	
P21396	Amine oxidase [flavin-containing] A OS=Rattus norvegicus GN=Maoa PE=1 SV=1 - [AOFA_RAT]	59.5	0.000E0	1.524E-5	3.311E-5	6.746E-5		51.59	51.39	171.11		5.13	7.79	14.45		2	3	6		2	4	10
P21531	60S ribosomal protein L3 OS=Rattus norvegicus GN=Rpl3 PE=1 SV=3 - [RL3_RAT]	46.1	0.000E0	1.587E-4	4.135E-4	4.297E-4		284.97	828.32	722.08		13.65	44.91	30.02		6	16	12		15	37	29
P21533	60S ribosomal protein L6 OS=Rattus norvegicus GN=Rpl6 PE=1 SV=5 - [RL6_RAT]	33.5	8.336E-5	8.651E-4	9.400E-4	8.729E-4	178.65	1122.13	1319.17	1196.49	10.74	29.53	32.55	35.23	2	10	11	12	7	31	35	36
P21588	5'-nucleotidase OS=Rattus norvegicus GN=Nt5e PE=1 SV=1 - [5NTD_RAT]	63.9	2.571E-5	0.000E0	0.000E0	0.000E0	27.36				2.60				1				2			
P21670	Proteasome subunit alpha type-4 OS=Rattus norvegicus GN=Psma4 PE=1 SV=1 - [PSA4_RAT]	29.5	5.962E-5	3.680E-4	5.054E-4	2.963E-4	35.68	572.66	873.96	495.61	3.07	49.43	65.90	49.43	1	9	11	9	1	18	28	15
P21708	Mitogen-activated protein kinase 3 OS=Rattus norvegicus GN=Mapk3 PE=1 SV=5 - [MK03_RAT]	43.1	0.000E0	1.447E-4	1.710E-4	1.713E-4		295.40	410.58	288.72		31.84	46.05	29.74		10	13	9		21	30	21
P21807	Peripherin OS=Rattus norvegicus GN=Prph PE=1 SV=1 - [PERI_RAT]	53.5	1.587E-4	2.163E-4	2.586E-4	1.910E-4	130.27	264.24	257.55	209.18	10.47	6.41	6.41	7.91	5	4	4	5	7	8	7	9
P21913	Succinate dehydrogenase [ubiquinone] iron-sulfur subunit, mitochondrial OS=Rattus norvegicus OX=10116 GN=Sdhb PE=	31.8	5.046E-5	1.168E-4	1.173E-4	8.515E-5	24.40	318.06	311.18	195.37	12.06	26.95	33.69	27.66	4	8	10	8	4	13	15	12
P22062	Protein-L-isoaspartate(D-aspartate) O-methyltransferase OS=Rattus norvegicus GN=Pcmt1 PE=1 SV=2 - [PIMT_RAT]	24.6	0.000E0	4.817E-5	7.987E-5	2.266E-5		144.42	105.10	41.96		15.86	30.84	7.49		2	3	1		6	6	2
P22509	rRNA 2'-O-methyltransferase fibrillarin OS=Rattus norvegicus OX=10116 GN=Fbl PE=1 SV=2 - [FBRL_RAT]	34.2	0.000E0	7.419E-5	7.907E-5	1.320E-4		52.50	114.60	212.10		5.81	23.24	25.99		1	4	5		2	6	8
P22734	Catechol O-methyltransferase OS=Rattus norvegicus GN=Comt PE=1 SV=2 - [COMT_RAT]	29.6	3.166E-5	3.847E-4	3.391E-4	3.399E-4	43.04	347.90	357.93	388.02	3.79	47.73	45.08	57.95	1	9	9	9	2	18	19	16
P22791	Hydroxymethylglutaryl-CoA synthase, mitochondrial OS=Rattus norvegicus OX=10116 GN=Hmgcs2 PE=1 SV=1 - [HMCS2_	56.9	0.000E0	2.418E-4	3.620E-4	2.006E-4		377.09	623.76	325.39		27.36	30.31	20.87		13	16	10		20	28	17
P22985	Xanthine dehydrogenase/oxidase OS=Rattus norvegicus GN=Xdh PE=1 SV=3 - [XDH_RAT]	146.1	8.815E-4	1.099E-4	1.874E-4	7.903E-5	2401.47	436.82	969.85	202.89	45.76	20.21	25.54	8.26	38	16	20	9	104	25	40	16
P23358	60S ribosomal protein L12 OS=Rattus norvegicus GN=Rpl12 PE=2 SV=1 - [RL12_RAT]	17.8	8.840E-5	1.422E-3	1.385E-3	1.385E-3	167.18	857.51	858.62	816.76	54.55	63.64	63.64	63.64	6	8	8	8	9	28	27	28
P23457	3-alpha-hydroxysteroid dehydrogenase OS=Rattus norvegicus GN=Akr1c9 PE=1 SV=1 - [DIDH_RAT]	37.0	5.854E-4	1.522E-3	2.249E-3	1.749E-3	833.61	1709.88	2358.18	1493.08	49.07	60.56	73.91	62.73	13	17	20	18	32	64	74	61
P23514	Coatomer subunit beta OS=Rattus norvegicus GN=Copb1 PE=1 SV=1 - [COPB_RAT]	106.9	9.401E-5	0.000E0	7.158E-5	1.890E-5	85.77		225.33	37.94	6.51		14.06	1.15	6		9	1	8		11	2
P23739	Sucrase-isomaltase, intestinal OS=Rattus norvegicus GN=Si PE=1 SV=5 - [SUIS_RAT]	210.2	7.802E-5	1.867E-4	1.590E-4	1.591E-4	117.44	825.41	570.10	505.49	1.30	12.33	7.93	9.94	2	19	12	15	6	41	24	28
P23764	Glutathione peroxidase 3 OS=Rattus norvegicus GN=Gpx3 PE=2 SV=2 - [GPX3_RAT]	25.4	0.000E0	0.000E0	0.000E0	4.773E-5				22.37				13.72				2				3
P23785	Granulins OS=Rattus norvegicus GN=Grn PE=1 SV=3 - [GRN_RAT]	63.3	5.403E-5	0.000E0	2.465E-5	0.000E0	111.32		36.39		7.48		2.55		3		1		5		1	
P23897	Heat-stable enterotoxin receptor OS=Rattus norvegicus OX=10116 GN=Gucy2c PE=2 SV=2 - [GUC2C_RAT]	123.4	1.112E-4	9.515E-5	7.494E-5	1.255E-4	160.09	51.76	64.24	71.29	4.66	1.03	1.68	2.61	4	1	2	2	6	2	3	2
P23965	Enoyl-CoA delta isomerase 1, mitochondrial OS=Rattus norvegicus GN=Eci1 PE=1 SV=1 - [ECI1_RAT]	32.2	1.231E-4	1.121E-4	1.259E-4	9.568E-5	55.20	57.30	108.46	70.49	13.84	10.73	15.22	10.73	3	2	3	2	4	3	5	4
P24049	60S ribosomal protein L17 OS=Rattus norvegicus OX=10116 GN=Rpl17 PE=2 SV=3 - [RL17_RAT]	21.4	0.000E0	0.000E0	9.444E-5	2.350E-4			99.22	178.49			18.48	30.98			3	5			4	8
P24050	40S ribosomal protein S5 OS=Rattus norvegicus GN=Rps5 PE=1 SV=3 - [RS5_RAT]	22.9	3.022E-5	1.346E-4	3.142E-4	3.129E-4	30.45	167.69	361.84	391.97	6.37	28.92	47.55	33.33	1	5	9	7	1	7	16	15
P24051	40S ribosomal protein S27-like OS=Rattus norvegicus GN=Rps27l PE=1 SV=3 - [RS27L_RAT]	9.5	0.000E0	0.000E0	1.181E-4	1.180E-4			120.47	62.19			28.57	28.57			2	2			6	4
P24090	Alpha-2-HS-glycoprotein OS=Rattus norvegicus GN=Ahsg PE=1 SV=2 - [FETUA_RAT]	38.0	7.982E-4	4.699E-4	6.257E-4	4.066E-4	524.01	476.65	723.22	403.31	32.10	32.10	35.23	33.24	7	7	8	7	21	19	25	20
P24155	Thimet oligopeptidase OS=Rattus norvegicus GN=Thop1 PE=1 SV=4 - [THOP1_RAT]	78.3	1.134E-4	1.980E-4	2.147E-4	1.843E-4	318.79	625.75	729.11	511.37	18.63	24.75	26.93	24.02	11	13	15	13	15	24	27	22
P24268	Cathepsin D OS=Rattus norvegicus GN=Ctsd PE=1 SV=1 - [CATD_RAT]	44.7	7.809E-4	3.945E-4	5.428E-4	4.522E-4	633.00	520.06	757.69	472.02	31.45	21.38	31.45	26.54	10	6	9	8	31	23	30	26
P24329	Thiosulfate sulfurtransferase OS=Rattus norvegicus GN=Tst PE=1 SV=3 - [THTR_RAT]	33.4	0.000E0	1.929E-4	4.905E-4	4.092E-4		74.30	154.81	231.77		4.04	23.57	23.91		1	6	6		2	8	11
P24368	Peptidyl-prolyl cis-trans isomerase B OS=Rattus norvegicus GN=Ppib PE=1 SV=3 - [PPIB_RAT]	23.8	1.604E-3	1.136E-3	9.527E-4	1.229E-3	1256.78	1066.26	960.75	1124.27	53.70	52.31	53.70	53.70	12	11	11	12	49	36	37	42
P25030	Keratin, type I cytoskeletal 20 OS=Rattus norvegicus GN=Krt20 PE=2 SV=2 - [K1C20_RAT]	49.4	2.940E-3	7.859E-4	9.888E-4	9.439E-4	998.10	960.03	953.82	644.80	38.93	42.42	34.73	27.74	21	22	18	14	49	41	36	25
P25093	Fumarylacetoacetase OS=Rattus norvegicus GN=Fah PE=1 SV=1 - [FAAA_RAT]	45.9	2.419E-4	2.462E-5	4.319E-5	0.000E0	561.76	78.23	194.55		35.80	13.13	19.09		10	4	5		24	5	8	
P25113	Phosphoglycerate mutase 1 OS=Rattus norvegicus GN=Pgam1 PE=1 SV=4 - [PGAM1_RAT]	28.8	4.523E-3	2.275E-3	2.421E-3	2.091E-3	1810.92	1464.46	1879.04	1366.56	78.35	77.56	77.17	77.17	19	17	15	15	80	64	72	56
P25235	Dolichyl-diphosphooligosaccharide--protein glycosyltransferase subunit 2 OS=Rattus norvegicus OX=10116 GN=Rpn2 PE=	69.0	9.435E-5	4.490E-5	4.442E-5	0.000E0	127.07	125.71	184.70		12.84	9.35	7.92		5	4	3		6	7	5	
P25286	V-type proton ATPase 116 kDa subunit a isoform 1 OS=Rattus norvegicus OX=10116 GN=Atp6v0a1 PE=2 SV=1 - [VPP1_F	96.3	4.287E-5	3.062E-5	3.579E-5	0.000E0	22.50	24.54	28.95		2.27	2.51	3.46		1	2	2		1	3	2	
P25809	Creatine kinase U-type, mitochondrial OS=Rattus norvegicus GN=Ckmt1 PE=1 SV=1 - [KCRU_RAT]	47.0	8.116E-4	6.602E-4	6.057E-4	6.629E-4	678.01	515.50	660.30	574.60	48.09	36.12	47.61	38.52	14	13	13	12	36	26	30	29
P26051	CD44 antigen OS=Rattus norvegicus GN=Cd44 PE=1 SV=2 - [CD44_RAT]	55.9	1.493E-4	1.732E-4	1.692E-4	1.074E-4	52.81	72.19	70.03	37.91	2.39	2.39	2.39	2.39	1	1	1	1	2	2	2	1
P26284	Pyruvate dehydrogenase E1 component subunit alpha, somatic form, mitochondrial OS=Rattus norvegicus GN=Pdha1 PE=	43.2	0.000E0	1.220E-4	1.406E-4	2.340E-4		108.58	170.29	163.30		3.33	11.03	3.33		1	4	1		2	5	3
P26453	Basigin OS=Rattus norvegicus GN=Bsg PE=1 SV=2 - [BASI_RAT]	42.4	9.876E-5	6.310E-5	6.034E-5	5.350E-5	219.96	103.17	148.65	64.84	11.86	2.58	6.44	6.44	4	1	2	2	7	2	4	3
P26772	10 kDa heat shock protein, mitochondrial OS=Rattus norvegicus GN=Hspe1 PE=1 SV=3 - [CH10_RAT]	10.9	4.983E-4	4.207E-4	2.876E-4	2.567E-4	328.44	180.20	138.67	178.92	59.80	36.27	50.00	42.16	6	4	5	4	16	9	9	7
P27008	Poly [ADP-ribose] polymerase 1 OS=Rattus norvegicus GN=Parp1 PE=1 SV=4 - [PARP1_RAT]	112.6	0.000E0	3.840E-5	0.000E0	7.778E-5		74.50		85.18		3.25		6.61		3		5		5		8
P27139	Carbonic anhydrase 2 OS=Rattus norvegicus GN=Ca2 PE=1 SV=2 - [CAH2_RAT]	29.1	8.177E-4	3.958E-4	3.595E-4	4.639E-4	467.92	587.28	513.50	550.76	67.69	67.69	71.15	59.23	11	12	13	10	27	28	23	21
P27213	6-pyruvoyl tetrahydrobiopterin synthase OS=Rattus norvegicus OX=10116 GN=Pts PE=1 SV=1 - [PTPS_RAT]	16.2	1.455E-4	8.207E-5	7.901E-5	9.605E-5	45.50	27.81	34.87	44.48	6.25	6.25	6.25	6.25	1	1	1	1	2	1	2	2
P27605	Hypoxanthine-guanine phosphoribosyltransferase OS=Rattus norvegicus GN=Hprt1 PE=1 SV=1 - [HPRT_RAT]	24.5	1.392E-4	2.224E-4	2.649E-4	2.199E-4	303.31	602.00	617.05	421.64	43.58	49.54	49.54	49.54	8	9	9	9	14	19	19	16
P27615	Lysosome membrane protein 2 OS=Rattus norvegicus OX=10116 GN=Scarb2 PE=1 SV=2 - [SCRB2_RAT]	54.1	5.086E-5	4.834E-5	0.000E0	0.000E0	26.43	33.92			2.51	2.51			1	1			2	2		
P27657	Pancreatic triacylglycerol lipase OS=Rattus norvegicus OX=10116 GN=Pnlip PE=1 SV=1 - [LIPP_RAT]	51.4	1.375E-4	0.000E0	0.000E0	0.000E0	40.04				10.97				4				6			
P27791	cAMP-dependent protein kinase catalytic subunit alpha OS=Rattus norvegicus GN=Prkaca PE=1 SV=2 - [KAPCA_RAT]	40.6	0.000E0	3.680E-5	5.621E-5	8.242E-5		27.03	64.02	39.83		9.40	11.97	2.56		2	3	1		2	4	2
P27867	Sorbitol dehydrogenase OS=Rattus norvegicus GN=Sord PE=1 SV=4 - [DHSO_RAT]	38.2	0.000E0	2.528E-5	6.745E-5	4.325E-5		37.29	93.12	36.89		14.01	16.25	13.17		4	5	4		4	10	5
P27952	40S ribosomal protein S2 OS=Rattus norvegicus GN=Rps2 PE=1 SV=1 - [RS2_RAT]	31.2	4.324E-5	3.744E-4	6.658E-4	7.102E-4	45.25	337.39	685.01	560.17	3.75	32.42	42.32	41.98	1	8	12	10	1	15	25	25
P28023	Dynactin subunit 1 OS=Rattus norvegicus GN=Dctn1 PE=1 SV=2 - [DCTN1_RAT]	141.8	0.000E0	3.276E-4	0.000E0	0.000E0		49.18				1.48				2				2		
P28037	Cytosolic 10-formyltetrahydrofolate dehydrogenase OS=Rattus norvegicus GN=Aldh1l1 PE=1 SV=3 - [AL1L1_RAT]	98.8	0.000E0	4.579E-5	5.465E-5	3.073E-5		38.52	79.87	22.35		5.99	7.10	3.10		5	6	3		6	7	3
P28042	Single-stranded DNA-binding protein, mitochondrial OS=Rattus norvegicus GN=Ssbp1 PE=1 SV=2 - [SSBP_RAT]	17.4	0.000E0	0.000E0	0.000E0	4.884E-5				25.14				5.30				1				2
P28073	Proteasome subunit beta type-6 OS=Rattus norvegicus GN=Psmb6 PE=1 SV=3 - [PSB6_RAT]	25.3	0.000E0	1.910E-4	3.157E-4	1.046E-4		255.22	249.08	207.78		25.21	37.39	12.18		6	6	3		10	11	6
P28075	Proteasome subunit beta type-5 OS=Rattus norvegicus GN=Psmb5 PE=1 SV=3 - [PSB5_RAT]	28.6	0.000E0	7.620E-5	1.914E-4	1.121E-4		121.18	516.17	171.48		13.31	39.16	28.14		3	9	6		6	17	9
P28077	Proteasome subunit beta type-9 OS=Rattus norvegicus GN=Psmb9 PE=1 SV=2 - [PSB9_RAT]	23.3	0.000E0	2.960E-5	8.403E-5	0.000E0		47.95	70.73			17.35	9.59			3	1			4	2	
P28480	T-complex protein 1 subunit alpha OS=Rattus norvegicus GN=Tcp1 PE=1 SV=1 - [TCPA_RAT]	60.3	0.000E0	1.448E-4	2.862E-4	1.998E-4		278.22	709.76	394.61		21.40	47.84	29.14		9	17	12		13	30	22

P28494	Alpha-mannosidase 2 OS=Rattus norvegicus GN=Man2a1 PE=1 SV=2 - [MA2A1_RAT]	131.2	4.024E-5	7.955E-5	5.571E-5	5.072E-5	94.96	25.72	29.79	28.73	3.92	1.92	0.96	0.96	4	2	1	1	6	3	1	2
P28648	CD63 antigen OS=Rattus norvegicus GN=Cd63 PE=1 SV=2 - [CD63_RAT]	25.7	1.899E-4	0.000E0	1.701E-4	1.577E-4	83.53		38.03	51.85	7.56		7.56	7.56	1		1	1	2		2	2
P28826	Meprin A subunit beta OS=Rattus norvegicus OX=10116 GN=Mep1b PE=1 SV=3 - [MEP1B_RAT]	79.2	5.877E-4	4.924E-4	5.041E-4	4.065E-4	1030.39	927.31	946.96	761.67	30.54	30.82	29.69	27.56	14	15	14	13	37	31	32	28
P29117	Peptidyl-prolyl cis-trans isomerase F, mitochondrial OS=Rattus norvegicus OX=10116 GN=Ppif PE=1 SV=2 - [PPIF_RAT]	21.8	3.177E-4	1.241E-4	1.179E-4	9.416E-5	226.90	142.26	216.97	61.01	51.94	38.83	22.82	18.93	7	6	4	3	14	8	7	5
P29147	D-beta-hydroxybutyrate dehydrogenase, mitochondrial OS=Rattus norvegicus OX=10116 GN=Bdh1 PE=1 SV=2 - [BDH_RA	38.2	0.000E0	1.879E-5	2.434E-5	3.868E-5		44.33	43.47	53.63		5.25	5.25	5.25		2	2	2		3	2	3
P29266	3-hydroxyisobutyrate dehydrogenase, mitochondrial OS=Rattus norvegicus GN=Hibadh PE=1 SV=3 - [3HIDH_RAT]	35.3	0.000E0	9.056E-5	1.070E-4	5.027E-5		220.55	177.24	75.18		22.39	13.73	12.84		5	3	3		9	5	4
P29314	40S ribosomal protein S9 OS=Rattus norvegicus GN=Rps9 PE=1 SV=4 - [RS9_RAT]	22.6	2.798E-5	3.264E-4	7.895E-4	8.967E-4	29.46	205.91	429.47	496.40	4.12	30.41	36.60	45.36	1	7	10	14	1	11	22	31
P29315	Ribonuclease inhibitor OS=Rattus norvegicus GN=Rnh1 PE=1 SV=2 - [RINI_RAT]	49.9	0.000E0	1.266E-4	2.744E-4	4.397E-4		569.23	833.00	466.80		42.11	56.36	49.12		11	16	14		18	29	23
P29410	Adenylate kinase 2, mitochondrial OS=Rattus norvegicus GN=Ak2 PE=2 SV=2 - [KAD2_RAT]	26.4	3.047E-4	7.841E-4	1.096E-3	9.196E-4	425.30	1030.07	1288.20	1075.89	54.39	70.29	70.29	65.27	11	15	15	13	22	36	48	39
P29411	GTP:AMP phosphotransferase AK3, mitochondrial OS=Rattus norvegicus GN=Ak3 PE=2 SV=2 - [KAD3_RAT]	25.4	4.477E-5	7.787E-5	1.042E-4	9.026E-5	73.77	300.10	242.36	196.64	14.54	24.23	14.54	14.54	3	5	3	3	4	10	8	7
P29457	Serpin H1 OS=Rattus norvegicus GN=Serpinh1 PE=1 SV=1 - [SERPH_RAT]	46.5	9.513E-5	7.405E-5	1.525E-4	2.130E-4	63.12	52.47	106.32	515.29	4.08	4.08	18.23	26.14	1	1	6	8	2	2	7	19
P29476	Nitric oxide synthase, brain OS=Rattus norvegicus OX=10116 GN=Nos1 PE=1 SV=1 - [NOS1_RAT]	160.5	3.117E-5	0.000E0	0.000E0	0.000E0	21.45				0.63				1				1			
P30349	Leukotriene A-4 hydrolase OS=Rattus norvegicus GN=Lta4h PE=2 SV=2 - [LKHA4_RAT]	69.1	7.433E-5	6.906E-4	8.683E-4	5.473E-4	49.25	1592.86	2046.65	1697.19	1.97	43.28	46.07	46.07	1	21	22	22	2	56	71	68
P30427	Plectin OS=Rattus norvegicus GN=Plec PE=1 SV=2 - [PLEC_RAT]	533.2	2.729E-4	4.506E-4	3.702E-4	3.364E-4	1966.42	4080.73	4406.28	2164.58	21.95	25.07	29.25	19.29	84	95	109	70	129	170	189	115
P30713	Glutathione S-transferase theta-2 OS=Rattus norvegicus GN=Gstt2 PE=1 SV=3 - [GSTT2_RAT]	27.4	1.354E-4	1.075E-4	1.254E-4	9.830E-5	60.07	138.41	141.63	88.15	30.74	35.66	35.25	20.90	6	6	6	4	8	7	9	4
P30835	ATP-dependent 6-phosphofructokinase, liver type OS=Rattus norvegicus GN=Pfkl PE=2 SV=3 - [PFKAL_RAT]	85.3	0.000E0	6.044E-5	1.324E-4	9.808E-5		80.64	240.74	78.53		6.67	10.90	10.26		3	6	5		5	11	7
P30904	Macrophage migration inhibitory factor OS=Rattus norvegicus GN=Mif PE=1 SV=4 - [MIF_RAT]	12.5	0.000E0	9.838E-4	8.603E-4	5.184E-4		382.58	314.82	297.10		23.48	23.48	23.48		3	3	3		13	12	11
P30919	N(4)-(Beta-N-acetylglucosaminyl)-L-asparaginase OS=Rattus norvegicus OX=10116 GN=Aga PE=1 SV=2 - [ASPG_RAT]	37.1	4.588E-4	7.002E-5	6.786E-5	8.292E-5	410.12	144.85	120.91	143.51	26.67	13.91	20.29	17.39	7	2	5	3	20	4	6	5
P31000	Vimentin OS=Rattus norvegicus GN=Vim PE=1 SV=2 - [VIME_RAT]	53.7	9.119E-4	8.358E-4	8.509E-4	7.114E-4	1315.70	1197.77	1438.46	1205.15	45.06	41.42	43.13	40.34	23	18	22	21	45	36	43	41
P31044	Phosphatidylethanolamine-binding protein 1 OS=Rattus norvegicus GN=Pebp1 PE=1 SV=3 - [PEBP1_RAT]	20.8	9.760E-4	1.200E-3	1.129E-3	7.538E-4	710.06	1048.57	865.31	799.88	56.15	61.50	60.43	60.96	6	9	7	8	19	27	22	20
P31053	Leukocyte antigen CD37 OS=Rattus norvegicus OX=10116 GN=Cd37 PE=2 SV=1 - [CD37_RAT]	31.7	2.014E-5	0.000E0	0.000E0	0.000E0	20.98				2.85				1				1			
P31232	Transgelin OS=Rattus norvegicus GN=Tagln PE=1 SV=2 - [TAGL_RAT]	22.6	3.642E-3	6.916E-3	4.874E-3	3.556E-3	2063.63	4286.98	2897.47	2549.09	65.17	70.65	65.17	70.65	14	15	14	15	77	182	110	90
P31430	Dipeptidase 1 OS=Rattus norvegicus OX=10116 GN=Dpep1 PE=2 SV=2 - [DPEP1_RAT]	45.5	8.949E-4	4.207E-5	6.710E-5	3.112E-5	601.22	38.79	27.57	45.61	52.20	2.93	8.54	7.07	14	2	2	2	29	2	2	3
P31977	Ezrin OS=Rattus norvegicus GN=Ezr PE=1 SV=3 - [EZRI_RAT]	69.3	6.998E-4	1.287E-3	1.339E-3	1.428E-3	567.83	1473.25	1579.46	1386.23	31.40	53.92	51.71	53.58	20	33	34	32	40	79	80	76
P32362	Uroporphyrinogen decarboxylase (Fragment) OS=Rattus norvegicus GN=Urod PE=1 SV=1 - [DCUP_RAT]	40.4	2.117E-5	1.368E-4	1.962E-4	1.766E-4	32.07	277.64	364.43	273.46	1.92	21.98	21.98	21.98	1	5	5	5	1	11	16	10
P32551	Cytochrome b-c1 complex subunit 2, mitochondrial OS=Rattus norvegicus OX=10116 GN=Uqcrc2 PE=1 SV=2 - [QCR2_RA	48.4	4.174E-5	3.327E-5	2.020E-5	5.594E-5	27.48	24.80	29.92	72.26	5.97	6.42	3.54	14.38	2	2	1	5	3	3	1	7
P34058	Heat shock protein HSP 90-beta OS=Rattus norvegicus GN=Hsp90ab1 PE=1 SV=4 - [HS90B_RAT]	83.2	1.340E-3	3.726E-3	4.139E-3	3.126E-3	1387.00	4221.70	4846.45	4024.14	23.34	50.00	61.19	56.63	17	38	44	43	45	154	164	152
P34064	Proteasome subunit alpha type-5 OS=Rattus norvegicus GN=Psma5 PE=2 SV=1 - [PSA5_RAT]	26.4	1.632E-4	4.905E-4	6.219E-4	2.685E-4	118.02	482.49	565.85	294.40	13.28	46.89	46.89	25.31	2	8	8	4	3	13	20	9
P34067	Proteasome subunit beta type-4 OS=Rattus norvegicus GN=Psmb4 PE=1 SV=2 - [PSB4_RAT]	29.2	0.000E0	1.930E-4	3.018E-4	1.393E-4		368.43	604.35	208.43		28.14	37.26	31.18		5	6	6		17	20	11
P35171	Cytochrome c oxidase subunit 7A2, mitochondrial OS=Rattus norvegicus OX=10116 GN=Cox7a2 PE=1 SV=1 - [CX7A2_RA	9.3	3.723E-5	0.000E0	0.000E0	4.962E-5	38.43	51.41		67.02	12.05	12.05		12.05	1	1		1	1	1		1
P35213	14-3-3 protein beta/alpha OS=Rattus norvegicus GN=Ywhab PE=1 SV=3 - [1433B_RAT]	28.0	1.572E-3	5.663E-3	5.136E-3	4.574E-3	974.65	2656.79	2656.65	1912.81	54.88	62.60	62.60	59.76	12	16	16	15	40	93	93	77
P35280	Ras-related protein Rab-8A OS=Rattus norvegicus GN=Rab8a PE=1 SV=2 - [RAB8A_RAT]	23.7	4.624E-4	6.580E-4	1.013E-3	6.907E-4	169.48	186.67	159.32	81.58	14.01	10.63	10.63	10.63	3	2	2	2	8	7	5	4
P35427	60S ribosomal protein L13a OS=Rattus norvegicus GN=Rpl13a PE=1 SV=2 - [RL13A_RAT]	23.5	0.000E0	4.695E-4	5.714E-4	5.705E-4		329.27	266.61	334.02		24.14	26.11	30.54		5	5	7		13	8	15
P35433	Amidophosphoribosyltransferase OS=Rattus norvegicus OX=10116 GN=Ppat PE=1 SV=1 - [PUR1_RAT]	57.4	0.000E0	2.714E-5	2.964E-5	1.982E-5		103.96	71.07	29.33		5.03	7.93	1.74		2	3	1		4	5	1
P35434	ATP synthase subunit delta, mitochondrial OS=Rattus norvegicus OX=10116 GN=Atp5f1d PE=1 SV=2 - [ATPD_RAT]	17.6	1.281E-4	2.105E-4	2.112E-4	2.464E-4	92.18	146.06	139.29	102.72	8.33	13.69	13.69	13.69	1	2	2	2	2	3	4	2
P35435	ATP synthase subunit gamma, mitochondrial OS=Rattus norvegicus GN=Atp5c1 PE=1 SV=2 - [ATPG_RAT]	30.2	0.000E0	0.000E0	3.418E-5	5.650E-5			32.44	37.99			4.03	7.69			1	2			1	2
P35467	Protein S100-A1 OS=Rattus norvegicus OX=10116 GN=S100a1 PE=1 SV=3 - [S10A1_RAT]	10.6	1.251E-4	0.000E0	4.213E-5	0.000E0	109.14		76.32		26.60		19.15		3		2		6		5	
P35559	Insulin-degrading enzyme OS=Rattus norvegicus GN=Ide PE=1 SV=1 - [IDE_RAT]	117.6	0.000E0	1.619E-4	1.691E-4	1.298E-4		427.60	379.73	187.54		14.62	13.15	10.79		13	12	9		20	21	13
P35565	Calnexin OS=Rattus norvegicus GN=Canx PE=1 SV=1 - [CALX_RAT]	67.2	7.083E-4	5.224E-4	5.697E-4	3.898E-4	1080.62	987.32	1159.80	782.51	37.23	38.07	40.10	37.23	22	21	20	18	48	46	45	37
P35704	Peroxiredoxin-2 OS=Rattus norvegicus GN=Prdx2 PE=1 SV=3 - [PRDX2_RAT]	21.8	1.702E-3	1.973E-3	1.701E-3	1.273E-3	878.14	1049.48	986.61	614.46	40.40	40.40	40.40	40.40	8	8	8	8	34	35	35	28
P36633	Amiloride-sensitive amine oxidase [copper-containing] OS=Rattus norvegicus OX=10116 GN=Aoc1 PE=2 SV=1 - [AOC1_R	85.0	5.650E-5	1.033E-4	9.892E-5	8.591E-5	37.10	92.95	185.63	94.00	3.62	14.88	12.33	5.23	3	8	7	4	4	10	11	7
P36860	Ras-related protein Ral-B OS=Rattus norvegicus GN=Ralb PE=2 SV=1 - [RALB_RAT]	23.3	4.644E-5	0.000E0	3.606E-5	2.475E-5	53.66		40.62	23.47	19.42		8.74	3.40	4		2	1	6		3	1
P36876	Serine/threonine-protein phosphatase 2A 55 kDa regulatory subunit B alpha isoform OS=Rattus norvegicus GN=Ppp2r2a P	51.6	0.000E0	3.014E-5	6.041E-5	0.000E0		38.68	91.38			8.05	8.05			3	3			5	5	
P36972	Adenine phosphoribosyltransferase OS=Rattus norvegicus GN=Aprt PE=1 SV=1 - [APT_RAT]	19.5	6.605E-5	4.392E-4	4.958E-4	4.162E-4	51.23	414.18	671.30	331.61	18.33	71.11	69.44	55.00	2	8	9	7	4	19	26	18
P37397	Calponin-3 OS=Rattus norvegicus GN=Cnn3 PE=1 SV=1 - [CNN3_RAT]	36.4	5.242E-5	0.000E0	1.544E-4	5.658E-5	116.94		46.26	42.06	3.03		3.33	3.03	1		1	1	2		2	1
P37805	Transgelin-3 OS=Rattus norvegicus GN=Tagln3 PE=1 SV=2 - [TAGL3_RAT]	22.5	0.000E0	1.016E-4	1.809E-4	1.389E-4		58.34	55.88	48.99		11.06	4.02	4.02		2	1	1		3	2	2
P37996	ADP-ribosylation factor-like protein 3 OS=Rattus norvegicus GN=Arl3 PE=1 SV=2 - [ARL3_RAT]	20.4	0.000E0	1.066E-4	1.090E-4	8.872E-5		141.53	140.78	103.67		32.97	28.57	34.07		4	3	4		7	5	6
P38552	Galectin-4 OS=Rattus norvegicus GN=Lgals4 PE=1 SV=1 - [LEG4_RAT]	36.3	6.678E-3	4.107E-3	3.162E-3	2.252E-3	3233.94	2982.53	2766.44	2071.68	57.72	57.72	57.41	57.41	17	17	16	16	131	108	94	78
P38650	Cytoplasmic dynein 1 heavy chain 1 OS=Rattus norvegicus GN=Dync1h1 PE=1 SV=1 - [DYHC1_RAT]	531.9	0.000E0	9.532E-5	1.339E-4	8.577E-5		225.31	1117.67	392.64		3.47	12.19	6.01		11	42	22		16	66	33
P38652	Phosphoglucomutase-1 OS=Rattus norvegicus GN=Pgm1 PE=1 SV=2 - [PGM1_RAT]	61.4	0.000E0	6.635E-5	1.006E-4	1.000E-4		567.70	690.58	647.04		27.40	37.90	30.25		11	14	12		20	24	21
P38656	Lupus La protein homolog OS=Rattus norvegicus GN=Ssb PE=1 SV=1 - [LA_RAT]	47.7	4.685E-4	7.083E-4	6.613E-4	7.235E-4	978.65	1755.67	1535.07	1559.55	37.83	46.02	42.89	45.06	18	23	19	20	42	65	51	56
P38659	Protein disulfide-isomerase A4 OS=Rattus norvegicus GN=Pdia4 PE=1 SV=2 - [PDIA4_RAT]	72.7	1.941E-4	6.092E-4	6.019E-4	4.369E-4	338.95	1806.53	1751.00	1247.32	16.95	49.61	47.74	34.68	9	27	28	20	20	68	67	48
P38918	Aflatoxin B1 aldehyde reductase member 3 OS=Rattus norvegicus OX=10116 GN=Akr7a3 PE=1 SV=2 - [ARK73_RAT]	36.7	1.038E-3	7.484E-4	6.303E-4	4.453E-4	750.19	441.17	680.59	459.89	59.02	46.18	59.94	46.79	14	10	12	10	35	19	26	23
P38983	40S ribosomal protein SA OS=Rattus norvegicus GN=Rpsa PE=1 SV=3 - [RSSA_RAT]	32.8	5.724E-4	9.328E-4	1.338E-3	1.503E-3	543.54	615.32	1422.74	1334.82	30.85	31.86	39.66	51.86	6	7	8	11	16	15	31	37
P39032	60S ribosomal protein L36 OS=Rattus norvegicus OX=10116 GN=Rpl36 PE=1 SV=2 - [RL36_RAT]	12.3	0.000E0	0.000E0	1.310E-4	1.033E-4			96.28	129.95			12.38	20.95			2	3			4	4
P39052	Dynamin-2 OS=Rattus norvegicus GN=Dnm2 PE=1 SV=1 - [DYN2_RAT]	98.2	0.000E0	0.000E0	3.669E-5	3.346E-5			41.77	23.24			5.52	4.37			4	3			6	4
P40112	Proteasome subunit beta type-3 OS=Rattus norvegicus GN=Psmb3 PE=1 SV=1 - [PSB3_RAT]	22.9	4.998E-5	2.100E-4	3.366E-4	1.814E-4	34.13	246.89	426.63	199.57	8.78	27.32	38.54	20.00	1	4	7	3	2	13	17	10
P40241	CD9 antigen OS=Rattus norvegicus GN=Cd9 PE=1 SV=2 - [CD9_RAT]	25.2	3.010E-5	1.591E-5	2.264E-5	0.000E0	42.36	37.23	34.63	32.70	4.42	4.42	4.42	4.42	1	1	1	1	2	1	1	1
P40307	Proteasome subunit beta type-2 OS=Rattus norvegicus GN=Psmb2 PE=1 SV=1 - [PSB2_RAT]	22.9	3.688E-5	1.738E-4	3.088E-4	1.861E-4	41.33	247.46	286.51	217.16	5.47	20.90	36.32	24.38	1	3	4	4	2	10	13	10
P40329	Arginine--tRNA ligase, cytoplasmic OS=Rattus norvegicus GN=Rars PE=1 SV=2 - [SYRC_RAT]	75.8	0.000E0	1.977E-4	2.641E-4	1.857E-4		516.95	855.52	562.98		35.76	36.06	28.79		19	21	14		26	39	25
P40615	H/ACA ribonucleoprotein complex subunit 4 OS=Rattus norvegicus GN=Dkc1 PE=1 SV=4 - [DKC1_RAT]	56.6	0.000E0	7.115E-5	5.944E-5	7.421E-5		78.15	94.88	88.73		11.79	9.43	6.29		4	3	2		5	4	3
P41123	60S ribosomal protein L13 OS=Rattus norvegicus GN=Rpl13 PE=1 SV=2 - [RL13_RAT]	24.3	0.000E0	3.010E-5	8.819E-5	7.680E-5		64.65	71.78	143.41		5.21	5.21	14.69		1	1	3		2	1	6
P41350	Caveolin-1 OS=Rattus norvegicus OX=10116 GN=Cav1 PE=1 SV=3 - [CAV1_RAT]	20.5	0.000E0	0.000E0	4.333E-5	2.120E-5			37.27	40.57			4.49	4.49			1	1			1	2
P41498	Low molecular weight phosphotyrosine protein phosphatase OS=Rattus norvegicus GN=Acp1 PE=1 SV=3 - [PPAC_RAT]	18.1	0.000E0	1.240E-4	1.353E-4	1.073E-4		185.56	336.12	124.22		24.68	47.47	13.29		3	5	2		7	9	4
P41499	Tyrosine-protein phosphatase non-receptor type 11 OS=Rattus norvegicus GN=Ptpn11 PE=1 SV=4 - [PTN11_RAT]	68.4	3.708E-5	3.482E-5	2.211E-5	1.746E-5	51.05	225.00	130.06	40.57	4.52	12.06	7.04	7.04	2	5	3	3	2	8	5	4
P41516	DNA topoisomerase 2-alpha OS=Rattus norvegicus GN=Top2a PE=2 SV=1 - [TOP2A_RAT]	173.1	5.504E-5	3.991E-5	0.000E0	0.000E0	30.51	25.91			0.52	0.52			1	1			2	2		
P41542	General vesicular transport factor p115 OS=Rattus norvegicus GN=Uso1 PE=1 SV=1 - [USO1_RAT]	107.1	2.708E-5	5.154E-5	4.103E-5	2.005E-5	44.83	85.99	90.75	24.38	1.25	4.80	5.74	1.88	1	4	4	2	2	5	6	2
P41562	Isocitrate dehydrogenase [NADP] cytoplasmic OS=Rattus norvegicus GN=Idh1 PE=1 SV=1 - [IDHC_RAT]	46.7	1.870E-5	9.699E-4	1.500E-3	1.187E-3	22.53	1472.10	2159.55	1606.42	3.14	53.38	64.73	62.56	1	21	23	22	1	57	75	68
P42123	L-lactate dehydrogenase B chain OS=Rattus norvegicus GN=Ldhb PE=1 SV=2 - [LDHB_RAT]	36.6	1.565E-4	7.860E-4	1.113E-3	8.292E-4	219.68	442.20	546.35	378.11	6.29	25.15	29.04	17.96	2	8	10	7	3	15	22	13
P42676	Neurolysin, mitochondrial OS=Rattus norvegicus GN=Nln PE=1 SV=1 - [NEUL_RAT]	80.2	0.000E0	6.545E-5	6.861E-5	5.183E-5		212.04	243.24	142.39		11.93	14.91	8.81		6	7	4		9	11	5
P42930	Heat shock protein beta-1 OS=Rattus norvegicus GN=Hspb1 PE=1 SV=1 - [HSPB1_RAT]	22.9	0.000E0	0.000E0	0.000E0	3.170E-5				23.16				7.77				1				1
P43138	DNA-(apurinic or apyrimidinic site) lyase OS=Rattus norvegicus GN=Apex1 PE=1 SV=2 - [APEX1_RAT]	35.5	0.000E0	1.325E-4	7.841E-5	3.174E-4		361.29	157.74	522.80		40.06	25.24	47.95		9	5	10		15	8	19
P43244	Matrin-3 OS=Rattus norvegicus GN=Matr3 PE=1 SV=2 - [MATR3_RAT]	94.4	0.000E0	1.859E-4	2.741E-4	3.402E-4		141.36	201.21	248.28		6.39	6.63	9.82		3	4	6		5	10	12
P43245	Multidrug resistance protein 1 OS=Rattus norvegicus OX=10116 GN=Abcb1 PE=2 SV=1 - [MDR1_RAT]	141.3	4.427E-5	6.276E-5	4.870E-5	3.720E-5	67.39	118.27	88.38	75.66	1.02	1.02	2.98	1.02	1	1	2	1	2	2	3	2
P43278	Histone H1.0 OS=Rattus norvegicus OX=10116 GN=H1f0 PE=2 SV=2 - [H10_RAT]	20.9	0.000E0	0.000E0	0.000E0	2.704E-5				68.13				11.86				2				3
P43424	Galactose-1-phosphate uridylyltransferase OS=Rattus norvegicus OX=10116 GN=Galt PE=2 SV=1 - [GALT_RAT]	43.3	0.000E0	0.000E0	6.579E-5	3.650E-5			41.47	37.42			7.12	6.33			2	2			3	4
P43527	Caspase-1 OS=Rattus norvegicus GN=Casp1 PE=2 SV=1 - [CASP1_RAT]	45.5	0.000E0	1.268E-4	8.802E-5	7.618E-5		191.14	143.59	41.94		13.93	8.71	5.72		5	3	2		8	5	2
P45352	Thymidylate synthase OS=Rattus norvegicus GN=Tyms PE=1 SV=1 - [TYSY_RAT]	35.0	1.001E-3	1.990E-5	6.045E-4	0.000E0	24.27	24.80	23.47		2.61	2.93	2.61		1	1	1		2	1	2	
P45479	Palmitoyl-protein thioesterase 1 OS=Rattus norvegicus GN=Ppt1 PE=1 SV=1 - [PPT1_RAT]	34.4	2.271E-5	6.721E-5	8.057E-5	7.811E-5	32.71	84.62	224.87	76.67	4.90	10.78	17.65	10.78	1	2	4	2	1	2	6	3
P45592	Cofilin-1 OS=Rattus norvegicus GN=Cfl1 PE=1 SV=3 - [COF1_RAT]	18.5	4.619E-4	5.448E-4	1.486E-3	1.278E-3	436.66	694.38	1370.86	996.63	59.04	51.20	68.07	65.06	8	7	12	12	20	26	42	39
P45953	Very long-chain specific acyl-CoA dehydrogenase, mitochondrial OS=Rattus norvegicus OX=10116 GN=Acadvl PE=1 SV=1	70.7	6.782E-5	0.000E0	0.000E0	0.000E0	127.92				12.82				6				9			
P46101	Dipeptidyl aminopeptidase-like protein 6 OS=Rattus norvegicus GN=Dpp6 PE=1 SV=1 - [DPP6_RAT]	97.2	2.053E-5	0.000E0	0.000E0	0.000E0	28.65				2.33				2				2			
P46413	Glutathione synthetase OS=Rattus norvegicus GN=Gss PE=1 SV=1 - [GSHB_RAT]	52.3	5.581E-4	6.303E-3	2.859E-4	2.320E-4	1335.93	935.97	852.16	651.46	42.19	39.24	37.13	34.39	18	16	15	15	50	38	35	27
P46418	Glutathione S-transferase alpha-5 OS=Rattus norvegicus GN=Gsta5 PE=1 SV=2 - [GSTA5_RAT]	25.3	2.918E-3	4.773E-4	8.873E-4	8.065E-4	158.81	151.84	173.29	150.64	11.76	16.74	16.74	16.74	4	5	5	5	8	8	9	9
P46462	Transitional endoplasmic reticulum ATPase OS=Rattus norvegicus GN=Vcp PE=1 SV=3 - [TERA_RAT]	89.3	0.000E0	8.617E-4	1.394E-3	1.156E-3		2658.22	4137.70	3085.88		45.53	59.80	67.74		28	38	40		83	125	110
P46844	Biliverdin reductase A OS=Rattus norvegicus GN=Blvra PE=1 SV=1 - [BIEA_RAT]	33.5	2.671E-5	1.062E-4	1.460E-4	1.191E-4	54.10	331.29	381.95	279.53	14.58	45.08	50.85	41.36	3	10	10	9	3	18	20	15
P47198	60S ribosomal protein L22 OS=Rattus norvegicus GN=Rpl22 PE=2 SV=2 - [RL22_RAT]	14.8	2.147E-4	5.046E-4	4.911E-4	6.864E-4	117.07	422.67	251.22	290.36	10.16	36.72	36.72	36.72	1	3	3	3	2	12	7	10
P47245	Nardilysin OS=Rattus norvegicus GN=Nrd1 PE=1 SV=1 - [NRDC_RAT]	132.9	0.000E0	2.464E-5	4.008E-5	3.051E-5		93.31	92.39	46.21		2.15	2.24	2.33		3	3	3		4	4	4
P47727	Carbonyl reductase [NADPH] 1 OS=Rattus norvegicus GN=Cbr1 PE=1 SV=2 - [CBR1_RAT]	30.6	4.794E-4	1.224E-3	9.325E-4	1.083E-3	670.85	1347.31	1177.15	1272.36	39.71	51.99	54.51	65.34	12	14	15	16	25	48	45	45
P47728	Calretinin OS=Rattus norvegicus GN=Calb2 PE=1 SV=1 - [CALB2_RAT]	31.4	5.821E-5	6.937E-5	4.096E-5	3.180E-5	32.10	84.65	57.69	22.14	7.75	7.75	7.75	4.06	2	2	2	1	2	4	3	1
P47819	Glial fibrillary acidic protein OS=Rattus norvegicus GN=Gfap PE=1 SV=2 - [GFAP_RAT]	49.9	1.858E-3	7.133E-4	1.078E-3	1.223E-3	256.50	169.48	221.79	162.31	8.37	6.51	8.37	6.51	4	3	4	3	11	8	10	8
P47820	Angiotensin-converting enzyme OS=Rattus norvegicus GN=Ace PE=1 SV=1 - [ACE_RAT]	150.8	2.582E-4	2.911E-4	3.992E-4	4.780E-4	642.81	858.26	1171.36	1302.31	17.36	23.61	26.88	28.48	16	24	25	29	34	48	55	62
P47875	Cysteine and glycine-rich protein 1 OS=Rattus norvegicus GN=Csrp1 PE=2 SV=2 - [CSRP1_RAT]	20.6	1.658E-4	5.748E-4	5.762E-4	4.727E-4	106.84	205.92	222.71	305.50	22.28	27.98	36.27	36.79	3	5	5	6	4	8	9	10
P47942	Dihydropyrimidinase-related protein 2 OS=Rattus norvegicus GN=Dpysl2 PE=1 SV=1 - [DPYL2_RAT]	62.2	0.000E0	7.153E-4	9.247E-4	5.089E-4		1485.17	1996.86	1129.71		59.09	56.29	48.78		22	21	19		54	59	41
P48004	Proteasome subunit alpha type-7 OS=Rattus norvegicus GN=Psma7 PE=1 SV=1 - [PSA7_RAT]	28.3	0.000E0	4.667E-4	7.348E-4	4.622E-4		397.93	567.66	393.52		44.49	44.49	44.49		9	9	9		14	19	18
P48037	Annexin A6 OS=Rattus norvegicus GN=Anxa6 PE=1 SV=2 - [ANXA6_RAT]	75.7	1.409E-4	1.760E-4	1.886E-4	1.379E-4	512.05	777.26	752.25	536.78	24.22	31.95	28.38	30.61	15	18	17	18	26	32	32	28
P48199	C-reactive protein OS=Rattus norvegicus GN=Crp PE=1 SV=1 - [CRP_RAT]	25.5	7.744E-3	5.358E-5	4.031E-5	2.339E-5	1186.04	38.89	92.43	23.67	40.43	4.35	13.91	4.35	6	1	3	1	42	1	4	2
P48284	Carbonic anhydrase 4 OS=Rattus norvegicus OX=10116 GN=Ca4 PE=1 SV=1 - [CAH4_RAT]	35.1	9.313E-5	0.000E0	0.000E0	0.000E0	45.32				7.77				2				2			
P48500	Triosephosphate isomerase OS=Rattus norvegicus GN=Tpi1 PE=1 SV=2 - [TPIS_RAT]	26.8	8.736E-3	3.704E-3	4.454E-3	3.786E-3	3968.00	3093.57	3299.49	2793.77	85.94	85.94	86.35	85.94	17	17	18	17	110	86	93	78
P48508	Glutamate--cysteine ligase regulatory subunit OS=Rattus norvegicus GN=Gclm PE=1 SV=1 - [GSH0_RAT]	30.5	0.000E0	0.000E0	3.343E-5	2.708E-5			33.15	53.31			5.47	11.31			1	2			1	3
P48675	Desmin OS=Rattus norvegicus OX=10116 GN=Des PE=1 SV=2 - [DESM_RAT]	53.4	4.319E-4	7.049E-4	6.499E-4	4.779E-4	867.77	1556.93	1794.38	1245.70	33.05	39.02	53.73	46.27	15	19	23	21	29	43	49	39
P48679	Prelamin-A/C OS=Rattus norvegicus GN=Lmna PE=1 SV=1 - [LMNA_RAT]	74.3	1.006E-3	7.076E-4	3.421E-4	3.660E-4	2165.91	1730.50	934.08	905.24	44.21	40.45	28.87	34.74	32	28	19	23	78	62	35	41
P48721	Stress-70 protein, mitochondrial OS=Rattus norvegicus GN=Hspa9 PE=1 SV=3 - [GRP75_RAT]	73.8	9.207E-4	1.406E-3	1.209E-3	6.598E-4	1964.28	3834.42	3560.27	2083.13	35.79	58.76	66.13	49.34	22	36	37	29	61	120	121	74
P49134	Integrin beta-1 OS=Rattus norvegicus GN=Itgb1 PE=2 SV=1 - [ITB1_RAT]	88.4	1.754E-4	2.007E-4	2.263E-4	1.727E-4	231.35	648.95	564.33	318.48	13.52	16.52	20.78	19.52	8	10	12	11	17	23	22	18
P49242	40S ribosomal protein S3a OS=Rattus norvegicus GN=Rps3a PE=1 SV=2 - [RS3A_RAT]	29.9	4.001E-5	4.924E-4	7.996E-4	1.016E-3	33.38	322.37	503.29	448.32	3.03	33.71	39.02	35.61	1	8	10	9	1	17	24	25
P49432	Pyruvate dehydrogenase E1 component subunit beta, mitochondrial OS=Rattus norvegicus GN=Pdhb PE=1 SV=2 - [ODPB	39.0	0.000E0	3.219E-5	6.253E-5	5.940E-5		51.00	161.32	73.04		11.14	16.16	11.70		3	5	4		4	8	6
P49804	Regulator of G-protein signaling 8 OS=Rattus norvegicus OX=10116 GN=Rgs8 PE=1 SV=2 - [RGS8_RAT]	20.9	0.000E0	0.000E0	0.000E0	7.134E-5				32.79				9.44				2				2
P49911	Acidic leucine-rich nuclear phosphoprotein 32 family member A OS=Rattus norvegicus GN=Anp32a PE=2 SV=1 - [AN32A_	28.5	1.969E-4	3.037E-4	3.429E-4	2.470E-4	248.14	452.28	375.93	530.58	21.86	24.29	44.94	25.10	5	7	11	6	8	14	17	16
P50116	Protein S100-A9 OS=Rattus norvegicus OX=10116 GN=S100a9 PE=1 SV=3 - [S10A9_RAT]	13.1	0.000E0	2.210E-5	9.814E-5	9.912E-5		25.17	32.75	25.99		6.19	6.19	6.19		1	1	1		2	2	2
P50123	Glutamyl aminopeptidase OS=Rattus norvegicus OX=10116 GN=Enpep PE=1 SV=2 - [AMPE_RAT]	107.9	3.135E-3	9.429E-4	1.428E-3	1.079E-3	2620.36	1717.32	2460.51	1819.64	37.67	35.24	40.63	35.03	30	28	31	28	105	77	105	86
P50137	Transketolase OS=Rattus norvegicus GN=Tkt PE=1 SV=1 - [TKT_RAT]	67.6	4.997E-3	2.770E-3	3.077E-3	2.510E-3	4200.61	3517.28	4209.06	3160.88	73.68	65.01	75.76	71.11	37	33	38	36	157	127	151	121
P50398	Rab GDP dissociation inhibitor alpha OS=Rattus norvegicus GN=Gdi1 PE=1 SV=1 - [GDIA_RAT]	50.5	1.019E-4	7.530E-4	8.630E-4	7.172E-4	23.91	1121.02	1360.00	927.98	4.25	51.45	51.90	45.86	1	18	19	15	2	47	50	43
P50399	Rab GDP dissociation inhibitor beta OS=Rattus norvegicus GN=Gdi2 PE=1 SV=2 - [GDIB_RAT]	50.5	8.449E-5	1.046E-3	1.141E-3	9.106E-4	72.13	2336.54	2895.34	2011.86	8.31	76.40	81.57	81.57	3	35	35	33	6	99	110	91
P50430	Arylsulfatase B OS=Rattus norvegicus GN=Arsb PE=2 SV=2 - [ARSB_RAT]	58.9	4.699E-4	9.215E-5	1.219E-4	1.181E-4	358.82	68.52	113.67	53.90	12.88	8.90	10.61	10.61	7	4	5	5	15	6	8	8
P50475	Alanine--tRNA ligase, cytoplasmic OS=Rattus norvegicus GN=Aars PE=1 SV=3 - [SYAC_RAT]	106.7	0.000E0	1.085E-4	1.176E-4	6.529E-5		456.15	572.50	219.03		14.67	20.56	7.02		11	15	6		20	23	9
P50503	Hsc70-interacting protein OS=Rattus norvegicus GN=St13 PE=1 SV=1 - [F10A1_RAT]	41.3	1.042E-3	1.184E-3	8.900E-4	9.971E-4	807.11	1162.86	874.34	680.58	24.46	24.46	19.29	24.46	9	9	7	9	31	31	25	25
P50554	4-aminobutyrate aminotransferase, mitochondrial OS=Rattus norvegicus GN=Abat PE=1 SV=3 - [GABT_RAT]	56.4	0.000E0	1.343E-5	0.000E0	0.000E0		34.35				9.20				3				4		
P50878	60S ribosomal protein L4 OS=Rattus norvegicus GN=Rpl4 PE=1 SV=3 - [RL4_RAT]	47.2	0.000E0	3.144E-4	6.290E-4	6.595E-4		525.30	968.71	735.96		22.33	34.92	34.92		9	13	12		20	40	31
P51146	Ras-related protein Rab-4B OS=Rattus norvegicus GN=Rab4b PE=2 SV=1 - [RAB4B_RAT]	23.6	7.511E-4	6.631E-4	1.040E-3	7.196E-4	83.62	107.96	73.32	56.21	8.92	5.16	5.16	5.16	2	1	1	1	4	5	3	2
P51574	Solute carrier family 15 member 1 OS=Rattus norvegicus OX=10116 GN=Slc15a1 PE=2 SV=1 - [S15A1_RAT]	78.9	6.967E-5	0.000E0	0.000E0	0.000E0	86.96				3.94				2				4			
P51607	N-acylglucosamine 2-epimerase OS=Rattus norvegicus GN=Renbp PE=2 SV=2 - [RENBP_RAT]	49.6	3.202E-5	6.768E-5	1.767E-4	1.175E-4	34.03	109.07	387.18	209.58	4.88	13.49	30.70	24.88	2	4	14	11	3	6	21	18
P51635	Alcohol dehydrogenase [NADP(+)] OS=Rattus norvegicus GN=Akr1a1 PE=1 SV=2 - [AK1A1_RAT]	36.5	2.550E-5	1.204E-3	1.206E-3	1.011E-3	41.09	1353.32	1451.60	1178.25	7.69	60.92	58.77	69.85	2	18	17	18	2	53	52	54
P51647	Retinal dehydrogenase 1 OS=Rattus norvegicus OX=10116 GN=Aldh1a1 PE=1 SV=3 - [AL1A1_RAT]	54.4	1.082E-3	6.526E-4	9.986E-4	7.116E-4	1330.82	1273.76	1681.24	917.56	43.51	51.10	44.91	42.51	16	19	21	18	46	43	62	38
P51650	Succinate-semialdehyde dehydrogenase, mitochondrial OS=Rattus norvegicus GN=Aldh5a1 PE=1 SV=2 - [SSDH_RAT]	56.1	0.000E0	2.762E-5	5.377E-5	9.026E-5		52.13	70.01	62.01		3.82	7.27	3.82		2	3	2		2	4	4
P51886	Lumican OS=Rattus norvegicus GN=Lum PE=1 SV=1 - [LUM_RAT]	38.3	1.040E-4	3.567E-4	3.651E-4	2.589E-4	106.86	683.75	807.24	449.58	13.02	39.05	34.91	28.11	4	11	10	8	6	29	31	20
P52020	Squalene monooxygenase OS=Rattus norvegicus OX=10116 GN=Sqle PE=2 SV=1 - [ERG1_RAT]	64.0	3.936E-5	0.000E0	0.000E0	0.000E0	20.43				1.22				1				1			
P52296	Importin subunit beta-1 OS=Rattus norvegicus GN=Kpnb1 PE=1 SV=1 - [IMB1_RAT]	97.1	2.939E-5	1.920E-4	2.781E-4	1.263E-4	90.10	494.51	1034.80	486.73	4.57	19.20	29.83	17.49	3	11	18	11	4	20	42	21
P52303	AP-1 complex subunit beta-1 OS=Rattus norvegicus GN=Ap1b1 PE=1 SV=1 - [AP1B1_RAT]	104.5	4.108E-4	1.387E-4	1.742E-4	9.654E-5	759.55	413.87	730.69	367.72	19.18	10.12	22.55	10.75	13	8	16	9	32	15	26	15
P52555	Endoplasmic reticulum resident protein 29 OS=Rattus norvegicus GN=Erp29 PE=1 SV=2 - [ERP29_RAT]	28.6	6.013E-4	8.040E-4	6.571E-4	6.022E-4	997.16	1079.22	854.80	783.33	46.15	46.15	46.15	44.23	10	10	10	9	28	32	27	31
P52631	Signal transducer and activator of transcription 3 OS=Rattus norvegicus GN=Stat3 PE=1 SV=1 - [STAT3_RAT]	88.0	0.000E0	5.424E-5	8.385E-5	0.000E0		56.30	234.10			4.94	12.73			3	7			3	13	
P52759	Ribonuclease UK114 OS=Rattus norvegicus GN=Hrsp12 PE=1 SV=3 - [UK114_RAT]	14.3	2.970E-4	1.963E-4	1.625E-4	1.716E-4	376.46	309.57	199.05	163.01	67.88	48.18	48.18	34.31	6	5	5	4	11	10	7	7
P52925	High mobility group protein B2 OS=Rattus norvegicus GN=Hmgb2 PE=2 SV=2 - [HMGB2_RAT]	24.1	0.000E0	2.577E-4	1.379E-4	1.296E-3		286.50	145.06	884.30		27.62	18.10	41.90		6	3	10		14	8	35
P52944	PDZ and LIM domain protein 1 OS=Rattus norvegicus GN=Pdlim1 PE=1 SV=4 - [PDLI1_RAT]	35.6	9.608E-5	8.442E-5	0.000E0	0.000E0	30.55	114.39			6.73	6.73			2	2			4	4		
P53042	Serine/threonine-protein phosphatase 5 OS=Rattus norvegicus GN=Ppp5c PE=1 SV=1 - [PPP5_RAT]	56.9	1.018E-4	9.889E-5	9.050E-5	8.000E-5	106.49	150.55	177.45	161.58	8.42	5.01	8.82	5.01	4	2	3	2	8	3	6	4
P53534	Glycogen phosphorylase, brain form (Fragment) OS=Rattus norvegicus GN=Pygb PE=1 SV=3 - [PYGB_RAT]	96.1	5.033E-5	1.415E-4	2.512E-4	2.126E-4	20.60	643.43	881.06	711.21	1.43	22.32	35.80	33.29	1	14	23	21	2	22	40	35
P53790	Sodium/glucose cotransporter 1 OS=Rattus norvegicus OX=10116 GN=Slc5a1 PE=2 SV=1 - [SC5A1_RAT]	73.0	1.182E-4	0.000E0	0.000E0	0.000E0	135.85				5.11				4				6			
P53812	Phosphatidylinositol transfer protein beta isoform OS=Rattus norvegicus GN=Pitpnb PE=1 SV=2 - [PIPNB_RAT]	31.4	0.000E0	1.422E-4	1.356E-4	1.509E-4		96.72	131.72	139.99		19.93	17.34	20.66		6	5	6		10	9	10
P53987	Monocarboxylate transporter 1 OS=Rattus norvegicus GN=Slc16a1 PE=1 SV=1 - [MOT1_RAT]	53.2	3.815E-5	2.372E-5	2.597E-5	0.000E0	124.77	97.03	84.47		2.63	2.63	2.63		1	1	1		2	2	2	
P54311	Guanine nucleotide-binding protein G(I)/G(S)/G(T) subunit beta-1 OS=Rattus norvegicus GN=Gnb1 PE=1 SV=4 - [GBB1_F	37.4	1.414E-4	1.474E-4	2.052E-4	1.526E-4	104.38	138.25	194.53	99.59	6.18	14.71	18.24	8.24	2	4	5	3	4	6	7	4
P54313	Guanine nucleotide-binding protein G(I)/G(S)/G(T) subunit beta-2 OS=Rattus norvegicus GN=Gnb2 PE=1 SV=4 - [GBB2_F	37.3	1.414E-4	1.474E-4	2.052E-4	1.706E-4	104.38	164.01	263.30	149.04	6.18	16.76	30.88	17.65	2	5	8	6	4	8	12	8
P54319	Phospholipase A-2-activating protein OS=Rattus norvegicus GN=Plaa PE=2 SV=3 - [PLAP_RAT]	87.0	1.001E-4	3.262E-5	3.537E-5	1.303E-5	41.30	179.81	259.13	21.20	1.01	11.19	12.70	1.01	1	6	7	1	2	7	10	1
P55053	Fatty acid-binding protein, epidermal OS=Rattus norvegicus GN=Fabp5 PE=1 SV=3 - [FABP5_RAT]	15.0	1.256E-3	1.293E-3	1.765E-3	1.216E-3	1175.14	1449.52	1801.39	1250.74	69.63	69.63	69.63	69.63	9	10	10	10	39	48	58	42
P55091	Chymotrypsin-C OS=Rattus norvegicus OX=10116 GN=Ctrc PE=1 SV=1 - [CTRC_RAT]	29.4	0.000E0	0.000E0	5.084E-5	6.804E-5			46.28	34.91			14.93	4.10			3	1			3	2
P55213	Caspase-3 OS=Rattus norvegicus GN=Casp3 PE=2 SV=2 - [CASP3_RAT]	31.5	0.000E0	7.887E-5	1.695E-4	9.675E-5	28.36	139.10	160.98	108.41	2.89	11.19	23.10	6.86	1	3	5	2	2	4	6	5
P55260	Annexin A4 OS=Rattus norvegicus GN=Anxa4 PE=1 SV=3 - [ANXA4_RAT]	35.8	1.719E-4	1.891E-4	2.082E-4	1.173E-4	488.94	624.95	928.70	398.87	38.24	36.68	42.63	30.09	11	11	14	8	25	20	31	15
P55281	Cadherin-17 OS=Rattus norvegicus OX=10116 GN=Cdh17 PE=2 SV=1 - [CAD17_RAT]	91.8	1.932E-3	1.010E-3	8.467E-4	3.098E-4	1287.09	1095.09	1156.13	330.56	26.60	24.55	32.29	18.50	19	14	18	9	51	36	41	18
P55770	NHP2-like protein 1 OS=Rattus norvegicus GN=Snu13 PE=2 SV=4 - [NH2L1_RAT]	14.2	0.000E0	1.647E-4	1.231E-4	1.088E-4		216.72	187.65	186.55		27.34	27.34	27.34		3	3	3		5	5	5
P56571	ES1 protein homolog, mitochondrial OS=Rattus norvegicus PE=1 SV=2 - [ES1_RAT]	28.2	0.000E0	1.033E-4	1.404E-4	2.087E-4		22.09	32.43	26.97		9.40	15.04	9.40		1	2	1		2	4	2
P56574	Isocitrate dehydrogenase [NADP], mitochondrial OS=Rattus norvegicus GN=Idh2 PE=1 SV=2 - [IDHP_RAT]	50.9	0.000E0	6.421E-4	1.006E-3	8.344E-4		1270.17	1467.68	972.27		47.35	54.20	46.46		20	22	21		51	59	49
P57113	Maleylacetoacetate isomerase OS=Rattus norvegicus GN=Gstz1 PE=1 SV=2 - [MAAI_RAT]	23.9	0.000E0	0.000E0	0.000E0	0.000E0			49.57	50.95			6.94	6.94			1	1			2	1
P58195	Phospholipid scramblase 1 OS=Rattus norvegicus OX=10116 GN=Plscr1 PE=1 SV=1 - [PLS1_RAT]	36.7	2.184E-4	5.109E-5	6.154E-5	5.805E-5	284.79	143.99	93.11	140.32	22.09	12.84	12.84	6.87	7	3	3	2	12	5	4	4

P58405	Striatin-3 OS=Rattus norvegicus OX=10116 GN=Strn3 PE=1 SV=2 - [STRN3_RAT]	87.1	0.000E0	2.044E-5	0.000E0	0.000E0		20.31				1.64				1				1		
P58775	Tropomyosin beta chain OS=Rattus norvegicus GN=Tpm2 PE=3 SV=1 - [TPM2_RAT]	32.8	1.688E-3	2.084E-3	3.057E-3	2.140E-3	1013.49	1442.78	1596.76	1355.73	34.86	42.25	44.72	41.90	14	20	22	17	33	48	56	49
P60123	RuvB-like 1 OS=Rattus norvegicus GN=Ruvbl1 PE=1 SV=1 - [RUVB1_RAT]	50.2	0.000E0	1.777E-5	9.969E-5	6.167E-5		37.19	225.87	123.96		5.92	21.49	15.79		2	7	5		4	11	7
P60522	Gamma-aminobutyric acid receptor-associated protein-like 2 OS=Rattus norvegicus GN=Gabarapl2 PE=3 SV=1 - [GBRL2_I	13.7	0.000E0	6.397E-5	5.629E-5	2.975E-5		158.64	47.78	54.88		17.09	9.40	9.40		3	1	1		4	1	2
P60711	Actin, cytoplasmic 1 OS=Rattus norvegicus GN=Actb PE=1 SV=1 - [ACTB_RAT]	41.7	2.102E-3	9.448E-3	1.678E-2	1.541E-2	2031.99	5706.83	8737.00	7377.28	67.73	72.53	71.73	73.07	20	22	24	24	76	240	356	326
P60868	40S ribosomal protein S20 OS=Rattus norvegicus GN=Rps20 PE=3 SV=1 - [RS20_RAT]	13.4	0.000E0	5.693E-5	2.682E-4	2.711E-4		72.98	191.89	194.08		15.13	28.57	25.21		2	4	3		2	8	6
P60892	Ribose-phosphate pyrophosphokinase 1 OS=Rattus norvegicus GN=Prps1 PE=1 SV=2 - [PRPS1_RAT]	34.8	0.000E0	3.982E-5	5.374E-5	3.931E-5		130.42	170.85	38.88		14.47	19.18	10.69		3	4	2		5	6	2
P60901	Proteasome subunit alpha type-6 OS=Rattus norvegicus GN=Psma6 PE=1 SV=1 - [PSA6_RAT]	27.4	1.059E-4	4.287E-4	6.409E-4	3.653E-4	63.93	617.28	844.59	581.03	4.88	48.78	48.78	47.97	1	10	11	10	2	19	26	18
P61023	Calcineurin B homologous protein 1 OS=Rattus norvegicus OX=10116 GN=Chp1 PE=1 SV=1 - [CHP1_RAT]	22.4	5.900E-5	0.000E0	0.000E0	0.000E0	46.61				6.15				1				1			
P61078	Ubiquitin-conjugating enzyme E2 D3 OS=Rattus norvegicus GN=Ube2d3 PE=2 SV=1 - [UB2D3_RAT]	16.7	1.599E-4	3.274E-4	2.553E-4	3.550E-4	32.58	175.90	170.15	188.71	7.48	28.57	21.09	40.82	1	3	2	4	1	6	6	7
P61107	Ras-related protein Rab-14 OS=Rattus norvegicus GN=Rab14 PE=1 SV=3 - [RAB14_RAT]	23.9	2.961E-4	3.190E-4	4.862E-4	3.276E-4	135.89	286.03	416.37	267.24	22.79	43.26	50.23	39.07	4	6	7	5	8	13	15	10
P61203	COP9 signalosome complex subunit 2 OS=Rattus norvegicus GN=Cops2 PE=2 SV=1 - [CSN2_RAT]	51.6	0.000E0	5.649E-5	9.601E-5	5.661E-5		70.29	169.45	86.33		7.00	19.64	9.93		3	8	4		6	11	6
P61206	ADP-ribosylation factor 3 OS=Rattus norvegicus GN=Arf3 PE=2 SV=2 - [ARF3_RAT]	20.6	4.276E-4	1.690E-4	2.522E-4	2.230E-4	553.11	496.30	560.83	543.11	53.59	50.28	54.70	54.70	7	7	8	8	16	14	16	18
P61212	ADP-ribosylation factor-like protein 1 OS=Rattus norvegicus GN=Arl1 PE=1 SV=1 - [ARL1_RAT]	20.4	7.532E-5	1.123E-4	1.253E-4	1.356E-4	68.53	59.16	35.56	33.49	11.05	6.08	6.08	6.08	2	1	1	1	2	2	1	2
P61314	60S ribosomal protein L15 OS=Rattus norvegicus GN=Rpl15 PE=1 SV=2 - [RL15_RAT]	24.1	0.000E0	4.319E-4	6.585E-4	6.507E-4		509.41	664.42	640.79		45.59	57.35	52.94		9	13	12		23	29	28
P61354	60S ribosomal protein L27 OS=Rattus norvegicus OX=10116 GN=Rpl27 PE=2 SV=1 - [RL27_RAT]	15.8	0.000E0	3.716E-4	4.547E-4	4.284E-4		289.23	300.32	245.49		40.44	47.06	42.65		5	6	6		13	14	15
P61459	Pterin-4-alpha-carbinolamine dehydratase OS=Rattus norvegicus OX=10116 GN=Pcbd1 PE=1 SV=2 - [PHS_RAT]	12.0	1.517E-4	9.120E-5	0.000E0	0.000E0	36.21	26.05			9.62	9.62			1	1			1	1		
P61480	Ribosome biogenesis protein WDR12 OS=Rattus norvegicus GN=Wdr12 PE=2 SV=1 - [WDR12_RAT]	47.5	0.000E0	3.185E-5	3.069E-5	0.000E0		49.76	29.65			7.57	1.65			2	1			3	1	
P61515	Putative 60S ribosomal protein L37a OS=Rattus norvegicus OX=10116 GN=Rpl37a-ps1 PE=5 SV=2 - [RL37P_RAT]	10.3	0.000E0	2.124E-5	6.334E-5	9.972E-5		51.68	64.66	155.73		8.70	28.26	41.30		1	2	3		2	3	5
P61589	Transforming protein RhoA OS=Rattus norvegicus GN=Rhoa PE=1 SV=1 - [RHOA_RAT]	21.8	5.825E-5	5.336E-4	6.030E-4	7.130E-4	115.59	465.70	637.50	487.95	19.17	51.30	55.44	57.51	3	9	9	8	4	17	17	23
P61751	ADP-ribosylation factor 4 OS=Rattus norvegicus GN=Arf4 PE=2 SV=2 - [ARF4_RAT]	20.4	3.311E-4	1.386E-4	2.151E-4	1.803E-4	202.42	234.31	335.60	318.82	33.33	34.44	34.44	34.44	4	5	5	5	9	10	10	11
P61959	Small ubiquitin-related modifier 2 OS=Rattus norvegicus GN=Sumo2 PE=1 SV=1 - [SUMO2_RAT]	10.9	0.000E0	1.313E-4	1.225E-4	1.129E-4		36.18	76.23	84.02		12.63	12.63	12.63		1	1	1		2	2	2
P61972	Nuclear transport factor 2 OS=Rattus norvegicus GN=Nutf2 PE=1 SV=1 - [NTF2_RAT]	14.5	2.068E-4	4.090E-4	2.828E-4	2.948E-4	128.33	236.98	223.62	174.09	56.69	56.69	50.39	56.69	4	4	3	4	6	12	6	7
P61980	Heterogeneous nuclear ribonucleoprotein K OS=Rattus norvegicus GN=Hnrnpk PE=1 SV=1 - [HNRPK_RAT]	50.9	7.141E-5	4.739E-4	4.316E-4	9.597E-4	42.94	827.79	898.52	943.26	2.59	26.78	29.16	30.89	1	10	11	11	1	26	29	31
P61983	14-3-3 protein gamma OS=Rattus norvegicus GN=Ywhag PE=1 SV=2 - [1433G_RAT]	28.3	1.572E-3	5.663E-3	5.136E-3	4.574E-3	728.43	2030.39	2147.92	1709.56	49.39	63.97	67.21	56.68	11	14	14	13	30	81	84	71
P62083	40S ribosomal protein S7 OS=Rattus norvegicus GN=Rps7 PE=1 SV=1 - [RS7_RAT]	22.1	4.201E-5	5.006E-5	5.466E-4	5.170E-4	66.17	66.24	435.68	425.98	10.31	21.65	54.12	54.12	2	4	10	9	3	6	24	22
P62138	Serine/threonine-protein phosphatase PP1-alpha catalytic subunit OS=Rattus norvegicus GN=Ppp1ca PE=1 SV=1 - [PP1A_	37.5	5.056E-4	6.048E-4	5.788E-4	4.365E-4	985.18	1162.09	1382.36	841.82	42.42	46.67	50.61	44.85	13	14	15	13	34	40	44	37
P62142	Serine/threonine-protein phosphatase PP1-beta catalytic subunit OS=Rattus norvegicus GN=Ppp1cb PE=1 SV=3 - [PP1B_F	37.2	5.056E-4	6.048E-4	5.788E-4	4.365E-4	965.54	1155.90	1338.62	870.99	40.06	49.24	51.38	55.05	12	14	15	14	32	41	42	39
P62161	Calmodulin OS=Rattus norvegicus GN=Calm1 PE=1 SV=2 - [CALM_RAT]	16.8	6.226E-4	4.527E-4	4.530E-4	6.541E-4	350.76	255.45	285.31	236.00	35.57	41.61	47.65	35.57	5	4	5	5	14	9	9	10
P62193	26S protease regulatory subunit 4 OS=Rattus norvegicus GN=Psmc1 PE=2 SV=1 - [PRS4_RAT]	49.2	0.000E0	0.000E0	3.569E-5	2.666E-5			102.59	20.73			11.14	4.77			3	1			5	1
P62198	26S protease regulatory subunit 8 OS=Rattus norvegicus GN=Psmc5 PE=1 SV=1 - [PRS8_RAT]	45.6	0.000E0	0.000E0	0.000E0	4.886E-5				40.75				4.68				1				2
P62243	40S ribosomal protein S8 OS=Rattus norvegicus GN=Rps8 PE=1 SV=2 - [RS8_RAT]	24.2	0.000E0	6.706E-5	5.042E-4	7.699E-4		222.66	684.68	568.82		41.35	41.35	50.96		7	7	9		9	16	21
P62246	40S ribosomal protein S15a OS=Rattus norvegicus GN=Rps15a PE=1 SV=2 - [RS15A_RAT]	14.8	2.703E-4	8.996E-4	1.040E-3	8.351E-4	320.72	655.48	589.09	653.05	51.54	51.54	51.54	51.54	6	6	6	6	10	19	19	18
P62250	40S ribosomal protein S16 OS=Rattus norvegicus GN=Rps16 PE=1 SV=2 - [RS16_RAT]	16.4	4.208E-5	2.395E-4	7.711E-4	6.957E-4	43.74	210.44	390.13	402.54	6.85	26.03	45.21	47.26	1	4	7	7	2	10	15	16
P62255	Ubiquitin-conjugating enzyme E2 G1 OS=Rattus norvegicus GN=Ube2g1 PE=2 SV=3 - [UB2G1_RAT]	19.5	6.132E-5	9.276E-5	7.560E-5	0.000E0	27.95	74.01	75.36		21.76	21.76	21.76		2	2	2		3	3	3	
P62260	14-3-3 protein epsilon OS=Rattus norvegicus GN=Ywhae PE=1 SV=1 - [1433E_RAT]	29.2	1.743E-3	6.835E-3	6.111E-3	5.293E-3	1413.66	4075.54	3880.91	3062.42	52.16	72.16	71.37	72.16	13	22	22	21	48	143	133	119
P62268	40S ribosomal protein S23 OS=Rattus norvegicus GN=Rps23 PE=1 SV=3 - [RS23_RAT]	15.8	0.000E0	0.000E0	8.420E-5	1.959E-4			78.67	187.24			18.88	26.57			1	2			2	4
P62271	40S ribosomal protein S18 OS=Rattus norvegicus GN=Rps18 PE=1 SV=3 - [RS18_RAT]	17.7	0.000E0	1.543E-4	6.160E-4	5.833E-4		297.41	549.44	494.17		25.00	40.13	36.18		4	7	6		8	17	16
P62278	40S ribosomal protein S13 OS=Rattus norvegicus GN=Rps13 PE=1 SV=2 - [RS13_RAT]	17.2	5.746E-5	1.725E-4	2.700E-4	4.318E-4	49.81	70.57	166.73	235.68	4.64	4.64	33.77	40.40	1	1	6	7	2	2	10	12
P62282	40S ribosomal protein S11 OS=Rattus norvegicus GN=Rps11 PE=1 SV=3 - [RS11_RAT]	18.4	0.000E0	0.000E0	9.641E-5	2.168E-4			83.63	223.29			20.25	36.71			3	6			5	12
P62329	Thymosin beta-4 OS=Rattus norvegicus OX=10116 GN=Tmsb4x PE=1 SV=2 - [TYB4_RAT]	5.0	4.611E-5	0.000E0	0.000E0	0.000E0	21.81				29.55				1				1			
P62425	60S ribosomal protein L7a OS=Rattus norvegicus GN=Rpl7a PE=1 SV=2 - [RL7A_RAT]	30.0	0.000E0	8.784E-4	8.666E-4	7.863E-4		897.07	977.84	767.46		42.48	46.99	53.01		14	15	17		33	38	35
P62489	DNA-directed RNA polymerase II subunit RPB7 OS=Rattus norvegicus GN=Polr2g PE=2 SV=1 - [RPB7_RAT]	19.3	0.000E0	4.814E-5	3.771E-5	0.000E0		47.58	62.37			7.56	7.56			1	1			2	2	
P62630	Elongation factor 1-alpha 1 OS=Rattus norvegicus GN=Eef1a1 PE=1 SV=1 - [EF1A1_RAT]	50.1	1.343E-3	3.420E-3	4.172E-3	5.406E-3	723.68	2236.04	2774.33	2448.77	32.03	54.55	54.55	63.42	11	17	17	18	25	80	102	105
P62634	Cellular nucleic acid-binding protein OS=Rattus norvegicus GN=Cnbp PE=2 SV=1 - [CNBP_RAT]	19.4	1.840E-4	1.193E-4	1.455E-4	2.157E-4	189.04	127.77	227.38	208.91	26.55	22.03	30.51	27.68	4	3	4	4	8	5	8	8
P62703	40S ribosomal protein S4, X isoform OS=Rattus norvegicus GN=Rps4x PE=2 SV=2 - [RS4X_RAT]	29.6	3.354E-5	2.323E-4	4.835E-4	4.498E-4	44.54	502.07	667.83	441.81	7.98	36.88	46.39	40.30	2	9	12	10	4	20	34	20
P62716	Serine/threonine-protein phosphatase 2A catalytic subunit beta isoform OS=Rattus norvegicus GN=Ppp2cb PE=1 SV=1 - [	35.6	1.026E-3	5.703E-4	5.245E-4	5.159E-4	1364.03	982.85	994.29	1055.84	68.28	67.31	60.84	58.58	16	15	13	13	46	34	33	38
P62718	60S ribosomal protein L18a OS=Rattus norvegicus OX=10116 GN=Rpl18a PE=2 SV=1 - [RL18A_RAT]	20.7	4.948E-5	4.604E-4	5.677E-4	5.149E-4	178.79	477.48	498.97	368.41	19.32	48.30	48.30	48.30	3	9	9	9	6	20	19	19
P62738	Actin, aortic smooth muscle OS=Rattus norvegicus GN=Acta2 PE=2 SV=1 - [ACTA_RAT]	42.0	1.721E-3	6.234E-3	1.155E-2	1.208E-2	1210.40	3554.48	4854.25	4726.31	53.85	70.56	71.09	70.03	16	21	23	21	52	159	207	213
P62744	AP-2 complex subunit sigma OS=Rattus norvegicus GN=Ap2s1 PE=1 SV=1 - [AP2S1_RAT]	17.0	0.000E0	9.312E-5	0.000E0	1.154E-4		32.46		33.98		4.93		4.93		1		1		2		2
P62752	60S ribosomal protein L23a OS=Rattus norvegicus GN=Rpl23a PE=2 SV=1 - [RL23A_RAT]	17.7	0.000E0	0.000E0	8.006E-5	1.592E-4			134.53	238.49			14.74	20.51			2	4			3	11
P62755	40S ribosomal protein S6 OS=Rattus norvegicus OX=10116 GN=Rps6 PE=1 SV=1 - [RS6_RAT]	28.7	0.000E0	0.000E0	1.258E-4	2.941E-4			98.31	240.85			10.84	20.88			2	4			2	9
P62775	Myotrophin OS=Rattus norvegicus GN=Mtpn PE=1 SV=2 - [MTPN_RAT]	12.9	0.000E0	7.861E-5	5.866E-5	7.858E-5		57.31	62.23	48.12		14.41	14.41	14.41		1	1	1		2	2	2
P62804	Histone H4 OS=Rattus norvegicus GN=Hist1h4b PE=1 SV=2 - [H4_RAT]	11.4	2.327E-3	3.680E-2	1.485E-2	4.366E-2	598.50	1697.15	1680.75	5035.58	50.49	53.40	53.40	53.40	5	8	8	8	19	55	52	128
P62815	V-type proton ATPase subunit B, brain isoform OS=Rattus norvegicus GN=Atp6v1b2 PE=1 SV=1 - [VATB2_RAT]	56.5	0.000E0	4.194E-5	1.455E-4	8.711E-5		22.51	434.48	127.12		8.22	38.55	10.76		2	12	4		2	20	7
P62828	GTP-binding nuclear protein Ran OS=Rattus norvegicus GN=Ran PE=1 SV=3 - [RAN_RAT]	24.4	0.000E0	1.786E-3	1.765E-3	1.930E-3		895.55	696.00	684.64		43.06	43.06	43.06		9	9	9		30	24	28
P62832	60S ribosomal protein L23 OS=Rattus norvegicus GN=Rpl23 PE=2 SV=1 - [RL23_RAT]	14.9	0.000E0	3.716E-5	1.026E-4	2.083E-4		38.08	174.46	159.32		20.00	30.71	25.71		2	3	3		3	6	7
P62836	Ras-related protein Rap-1A OS=Rattus norvegicus GN=Rap1a PE=1 SV=1 - [RAP1A_RAT]	21.0	0.000E0	2.231E-5	4.735E-5	4.340E-5		95.81	197.07	140.63		10.33	15.76	15.76		2	3	3		3	8	6
P62845	40S ribosomal protein S15 OS=Rattus norvegicus GN=Rps15 PE=1 SV=2 - [RS15_RAT]	17.0	0.000E0	1.548E-4	2.525E-4	1.505E-4		56.35	74.66	61.27		40.00	40.69	22.07		3	4	3		6	12	8
P62850	40S ribosomal protein S24 OS=Rattus norvegicus GN=Rps24 PE=2 SV=1 - [RS24_RAT]	15.4	0.000E0	4.582E-5	1.321E-4	2.042E-4		49.98	178.71	343.61		11.28	11.28	20.30		1	1	2		1	3	7
P62853	40S ribosomal protein S25 OS=Rattus norvegicus GN=Rps25 PE=2 SV=1 - [RS25_RAT]	13.7	5.373E-5	3.793E-4	8.610E-4	7.975E-4	76.42	298.36	460.44	452.45	15.20	29.60	29.60	29.60	2	4	5	5	3	10	15	14
P62856	40S ribosomal protein S26 OS=Rattus norvegicus GN=Rps26 PE=3 SV=3 - [RS26_RAT]	13.0	0.000E0	2.417E-5	1.054E-4	2.109E-4		43.63	135.65	52.29		7.83	20.87	20.87		1	2	2		2	4	4
P62864	40S ribosomal protein S30 OS=Rattus norvegicus OX=10116 GN=Fau PE=1 SV=1 - [RS30_RAT]	6.6	0.000E0	0.000E0	4.028E-5	3.381E-4			22.28	78.79			16.95	16.95			1	1			1	2
P62890	60S ribosomal protein L30 OS=Rattus norvegicus GN=Rpl30 PE=3 SV=2 - [RL30_RAT]	12.8	3.887E-5	8.165E-4	7.268E-4	6.753E-4	53.29	533.20	438.44	461.66	20.87	58.26	59.13	58.26	2	5	6	5	3	15	16	15
P62898	Cytochrome c, somatic OS=Rattus norvegicus GN=Cycs PE=1 SV=2 - [CYC_RAT]	11.6	8.976E-5	3.849E-5	2.671E-5	5.023E-5	93.50	70.78	74.04	80.49	18.10	18.10	18.10	18.10	2	2	2	2	4	3	3	3
P62902	60S ribosomal protein L31 OS=Rattus norvegicus OX=10116 GN=Rpl31 PE=1 SV=1 - [RL31_RAT]	14.5	0.000E0	0.000E0	6.369E-5	1.309E-4			79.52	77.22			17.60	17.60			2	2			5	4
P62907	60S ribosomal protein L10a OS=Rattus norvegicus GN=Rpl10a PE=1 SV=2 - [RL10A_RAT]	24.8	0.000E0	6.640E-4	6.785E-4	5.359E-4		241.62	295.20	250.55		33.18	42.40	35.48		7	9	7		18	18	14
P62909	40S ribosomal protein S3 OS=Rattus norvegicus GN=Rps3 PE=1 SV=1 - [RS3_RAT]	26.7	1.127E-4	4.316E-4	8.381E-4	7.340E-4	309.96	934.57	1126.18	1074.78	33.74	61.73	61.73	61.73	8	14	14	14	13	34	38	35
P62912	60S ribosomal protein L32 OS=Rattus norvegicus OX=10116 GN=Rpl32 PE=1 SV=2 - [RL32_RAT]	15.8	0.000E0	0.000E0	2.286E-4	2.529E-4			262.57	229.53			33.33	33.33			4	4			9	11
P62914	60S ribosomal protein L11 OS=Rattus norvegicus GN=Rpl11 PE=1 SV=2 - [RL11_RAT]	20.2	0.000E0	1.053E-4	2.693E-4	4.805E-4		52.36	210.69	317.86		16.85	21.35	21.35		3	4	4		3	8	11
P62919	60S ribosomal protein L8 OS=Rattus norvegicus GN=Rpl8 PE=2 SV=2 - [RL8_RAT]	28.0	0.000E0	3.324E-4	4.750E-4	4.951E-4		264.52	303.58	316.82		35.80	42.41	45.14		8	9	10		16	17	19
P62944	AP-2 complex subunit beta OS=Rattus norvegicus GN=Ap2b1 PE=1 SV=1 - [AP2B1_RAT]	104.5	2.648E-4	1.654E-4	2.260E-4	1.547E-4	380.21	457.34	891.84	360.88	12.27	13.23	22.73	13.55	10	11	18	11	21	21	35	18
P62959	Histidine triad nucleotide-binding protein 1 OS=Rattus norvegicus GN=Hint1 PE=1 SV=5 - [HINT1_RAT]	13.8	8.022E-5	4.726E-4	5.376E-4	3.695E-4	91.13	235.53	193.42	203.69	23.02	58.73	77.78	77.78	3	5	6	6	4	11	13	10
P62961	Nuclease-sensitive element-binding protein 1 OS=Rattus norvegicus OX=10116 GN=Ybx1 PE=1 SV=3 - [YBOX1_RAT]	35.7	0.000E0	0.000E0	2.969E-5	5.381E-5			51.14	59.77			5.90	5.90			1	1			1	2
P62963	Profilin-1 OS=Rattus norvegicus GN=Pfn1 PE=1 SV=2 - [PROF1_RAT]	14.9	1.094E-3	5.231E-3	6.817E-3	5.657E-3	1062.24	2358.91	2715.85	2051.98	63.57	65.00	73.57	73.57	8	10	11	11	38	81	90	66
P62982	Ubiquitin-40S ribosomal protein S27a OS=Rattus norvegicus GN=Rps27a PE=1 SV=2 - [RS27A_RAT]	17.9	1.509E-3	7.890E-4	6.653E-4	1.074E-3	399.63	200.66	346.93	400.82	24.36	21.79	42.31	42.31	4	3	5	5	21	8	11	15
P62994	Growth factor receptor-bound protein 2 OS=Rattus norvegicus GN=Grb2 PE=1 SV=1 - [GRB2_RAT]	25.2	1.895E-4	1.209E-4	7.855E-5	3.783E-5	273.70	207.93	158.59	89.39	28.57	22.12	18.43	8.76	6	5	4	2	11	9	8	4
P62997	Transformer-2 protein homolog beta OS=Rattus norvegicus OX=10116 GN=Tra2b PE=1 SV=1 - [TRA2B_RAT]	33.6	2.078E-5	1.484E-4	2.412E-4	2.789E-4	69.98	284.66	410.94	438.65	8.68	15.97	15.97	26.74	2	4	4	6	3	9	11	13
P63004	Platelet-activating factor acetylhydrolase IB subunit alpha OS=Rattus norvegicus GN=Pafah1b1 PE=1 SV=2 - [LIS1_RAT]	46.6	4.082E-4	1.961E-4	2.177E-4	1.703E-4	820.32	625.25	632.51	358.99	58.54	28.78	39.76	23.90	17	10	12	8	33	24	24	19
P63018	Heat shock cognate 71 kDa protein OS=Rattus norvegicus GN=Hspa8 PE=1 SV=1 - [HSP7C_RAT]	70.8	9.925E-3	5.529E-3	5.401E-3	4.467E-3	7425.35	6764.18	7186.82	5488.44	65.17	58.05	65.94	56.19	40	35	36	32	244	220	226	181
P63029	Translationally-controlled tumor protein OS=Rattus norvegicus GN=Tpt1 PE=1 SV=1 - [TCTP_RAT]	19.4	6.890E-4	7.299E-4	6.746E-4	5.036E-4	505.98	738.75	581.26	528.38	34.88	34.88	34.88	33.72	8	8	8	7	22	28	22	20
P63036	DnaJ homolog subfamily A member 1 OS=Rattus norvegicus GN=Dnaja1 PE=1 SV=1 - [DNJA1_RAT]	44.8	0.000E0	7.120E-4	1.082E-3	8.313E-4		35.43	39.60	21.78		10.08	13.35	6.80		3	4	2		7	8	5
P63039	60 kDa heat shock protein, mitochondrial OS=Rattus norvegicus GN=Hspd1 PE=1 SV=1 - [CH60_RAT]	60.9	0.000E0	0.000E0	1.133E-4	8.091E-5			446.27	187.10			24.08	14.14			10	6			18	12
P63074	Eukaryotic translation initiation factor 4E OS=Rattus norvegicus GN=Eif4e PE=1 SV=1 - [IF4E_RAT]	25.0	0.000E0	2.574E-4	1.462E-4	1.839E-4		234.06	184.63	144.93		18.43	18.43	15.21		4	4	3		7	6	5
P63086	Mitogen-activated protein kinase 1 OS=Rattus norvegicus GN=Mapk1 PE=1 SV=3 - [MK01_RAT]	41.2	0.000E0	1.246E-4	1.486E-4	1.494E-4		188.29	243.24	301.32		16.20	21.79	26.54		5	6	9		13	15	18
P63088	Serine/threonine-protein phosphatase PP1-gamma catalytic subunit OS=Rattus norvegicus GN=Ppp1cc PE=1 SV=1 - [PP1(	37.0	5.056E-4	6.048E-4	5.788E-4	4.365E-4	899.87	1064.97	1331.00	796.14	40.56	44.89	52.01	40.56	12	13	15	12	32	37	43	35
P63095	Guanine nucleotide-binding protein G(s) subunit alpha isoforms short OS=Rattus norvegicus GN=Gnas PE=1 SV=1 - [GNA	45.6	7.820E-5	8.470E-5	2.076E-4	1.356E-4	21.11	77.94	41.93	49.29	2.79	6.09	2.79	2.79	1	2	1	1	1	3	2	2
P63102	14-3-3 protein zeta/delta OS=Rattus norvegicus GN=Ywhaz PE=1 SV=1 - [1433Z_RAT]	27.8	2.560E-3	7.873E-3	7.535E-3	6.812E-3	2224.71	3487.19	3524.17	3135.02	59.59	62.86	62.86	62.86	17	17	20	18	67	105	107	102
P63155	Crooked neck-like protein 1 OS=Rattus norvegicus GN=Crnkl1 PE=2 SV=1 - [CRNL1_RAT]	83.4	0.000E0	2.175E-5	1.188E-4	0.000E0		57.73	22.04			6.96	3.62			4	2			4	3	
P63159	High mobility group protein B1 OS=Rattus norvegicus GN=Hmgb1 PE=1 SV=2 - [HMGB1_RAT]	24.9	0.000E0	3.881E-4	3.277E-4	2.576E-3		323.91	329.63	1180.77		26.05	26.05	43.72		5	5	10		11	12	42
P63170	Dynein light chain 1, cytoplasmic OS=Rattus norvegicus GN=Dynll1 PE=1 SV=1 - [DYL1_RAT]	10.4	5.048E-4	3.697E-4	2.528E-4	3.461E-4	148.01	202.11	136.81	105.12	24.72	49.44	49.44	24.72	2	3	3	2	7	7	7	6
P63245	Guanine nucleotide-binding protein subunit beta-2-like 1 OS=Rattus norvegicus GN=Gnb2l1 PE=1 SV=3 - [GBLP_RAT]	35.1	9.353E-5	1.554E-3	1.735E-3	1.353E-3	214.90	1956.49	2019.93	1667.49	25.87	78.23	83.28	83.91	6	16	18	18	11	56	61	53
P63255	Cysteine-rich protein 1 OS=Rattus norvegicus OX=10116 GN=Crip1 PE=1 SV=2 - [CRIP1_RAT]	8.5	0.000E0	1.290E-4	1.398E-4	2.491E-4		51.31	103.36	93.33		12.99	49.35	12.99		1	2	1		4	5	4
P63281	SUMO-conjugating enzyme UBC9 OS=Rattus norvegicus GN=Ube2i PE=1 SV=1 - [UBC9_RAT]	18.0	0.000E0	9.231E-5	0.000E0	3.062E-5		72.19		34.10		41.77		14.56		5		2		6		2
P63312	Thymosin beta-10 OS=Rattus norvegicus OX=10116 GN=Tmsb10 PE=1 SV=2 - [TYB10_RAT]	5.0	3.142E-5	0.000E0	0.000E0	2.576E-5	65.74			52.05	18.18			18.18	1			1	2			1
P63324	40S ribosomal protein S12 OS=Rattus norvegicus GN=Rps12 PE=1 SV=2 - [RS12_RAT]	14.5	0.000E0	1.824E-5	2.667E-4	9.167E-5		26.49	262.14	130.08		7.58	42.42	35.61		1	5	4		1	11	9
P63326	40S ribosomal protein S10 OS=Rattus norvegicus GN=Rps10 PE=2 SV=1 - [RS10_RAT]	18.9	0.000E0	6.417E-5	1.478E-4	2.085E-4		50.08	216.17	266.28		5.45	22.42	22.42		1	4	4		1	10	14
P63329	Serine/threonine-protein phosphatase 2B catalytic subunit alpha isoform OS=Rattus norvegicus GN=Ppp3ca PE=1 SV=1 -	58.6	8.047E-5	1.097E-4	1.042E-4	5.456E-5	135.65	145.96	148.23	84.83	3.84	12.67	9.40	5.76	2	5	5	3	6	10	10	4
P63331	Serine/threonine-protein phosphatase 2A catalytic subunit alpha isoform OS=Rattus norvegicus GN=Ppp2ca PE=1 SV=1 -	35.6	1.026E-3	5.703E-4	5.245E-4	5.159E-4	1485.54	1009.79	1079.70	1159.89	70.87	67.96	63.43	61.17	17	15	14	14	50	36	37	42
P67779	Prohibitin OS=Rattus norvegicus OX=10116 GN=Phb PE=1 SV=1 - [PHB_RAT]	29.8	0.000E0	0.000E0	1.918E-5	4.628E-5			43.23	52.30			4.04	7.35			1	2			1	3
P67874	Casein kinase II subunit beta OS=Rattus norvegicus GN=Csnk2b PE=1 SV=1 - [CSK2B_RAT]	24.9	2.592E-5	1.379E-4	5.398E-5	5.846E-5	30.79	206.69	61.41	72.67	5.12	19.07	12.56	5.12	1	3	2	1	1	6	2	2
P68101	Eukaryotic translation initiation factor 2 subunit 1 OS=Rattus norvegicus GN=Eif2s1 PE=1 SV=2 - [IF2A_RAT]	36.1	0.000E0	1.540E-4	9.212E-5	7.731E-5		539.58	406.80	209.64		39.37	30.79	21.90		10	8	6		19	14	10
P68182	cAMP-dependent protein kinase catalytic subunit beta OS=Rattus norvegicus GN=Prkacb PE=1 SV=2 - [KAPCB_RAT]	40.7	0.000E0	3.128E-5	4.247E-5	5.649E-5		42.66	106.67	97.35		5.98	8.55	5.98		2	3	2		2	4	4
P68255	14-3-3 protein theta OS=Rattus norvegicus GN=Ywhaq PE=1 SV=1 - [1433T_RAT]	27.8	1.572E-3	5.663E-3	5.136E-3	4.574E-3	1334.45	2740.32	2721.80	2648.63	60.00	62.86	62.86	60.00	17	19	20	19	48	99	92	90
P68370	Tubulin alpha-1A chain OS=Rattus norvegicus GN=Tuba1a PE=1 SV=1 - [TBA1A_RAT]	50.1	5.754E-4	6.661E-4	1.238E-3	1.839E-3	668.72	746.83	1752.01	1892.31	28.82	33.04	48.12	54.10	11	11	17	18	28	30	63	67
P68511	14-3-3 protein eta OS=Rattus norvegicus GN=Ywhah PE=1 SV=2 - [1433F_RAT]	28.2	1.572E-3	5.663E-3	5.136E-3	4.574E-3	864.83	2413.14	2261.50	1908.75	46.34	60.98	56.10	59.76	11	19	17	17	32	95	85	76
P69060	N-acylneuraminate cytidylyltransferase OS=Rattus norvegicus GN=Cmas PE=2 SV=1 - [NEUA_RAT]	48.1	0.000E0	0.000E0	3.192E-5	4.526E-5			44.29	72.90			5.56	5.56			2	2			3	4
P69897	Tubulin beta-5 chain OS=Rattus norvegicus GN=Tubb5 PE=1 SV=1 - [TBB5_RAT]	49.6	3.773E-4	3.920E-4	1.065E-3	1.251E-3	687.38	800.01	2080.37	2753.12	34.01	29.73	59.01	66.44	12	12	20	22	33	37	80	103
P70470	Acyl-protein thioesterase 1 OS=Rattus norvegicus GN=Lypla1 PE=1 SV=1 - [LYPA1_RAT]	24.7	0.000E0	7.307E-5	5.968E-5	4.630E-5		132.10	66.92	48.74		15.22	9.13	9.13		3	2	2		5	2	3
P70473	Alpha-methylacyl-CoA racemase OS=Rattus norvegicus GN=Amacr PE=1 SV=3 - [AMACR_RAT]	41.8	6.003E-5	0.000E0	0.000E0	0.000E0	54.65				2.36				1				2			
P70490	Lactadherin OS=Rattus norvegicus GN=Mfge8 PE=2 SV=1 - [MFGM_RAT]	47.4	1.313E-4	0.000E0	0.000E0	0.000E0	282.49				17.56				6				11			
P70552	GTP cyclohydrolase 1 feedback regulatory protein OS=Rattus norvegicus GN=Gchfr PE=1 SV=3 - [GFRP_RAT]	9.7	1.136E-4	0.000E0	0.000E0	0.000E0	224.46				52.38				3				6			
P70564	Serpin B5 OS=Rattus norvegicus OX=10116 GN=Serpinb5 PE=2 SV=1 - [SPB5_RAT]	42.0	0.000E0	6.559E-5	1.147E-4	1.192E-4		82.48	160.18	153.62		14.93	23.47	26.13		4	7	8		7	10	11
P70566	Tropomodulin-2 OS=Rattus norvegicus GN=Tmod2 PE=1 SV=1 - [TMOD2_RAT]	39.5	3.230E-4	2.738E-4	2.586E-4	1.798E-4	50.28	31.36	30.89	30.04	2.28	4.56	2.28	2.28	1	2	1	1	4	4	2	2
P70583	Deoxyuridine 5'-triphosphate nucleotidohydrolase OS=Rattus norvegicus GN=Dut PE=2 SV=3 - [DUT_RAT]	22.0	0.000E0	0.000E0	0.000E0	0.000E0		21.78	20.88			8.29	8.29			1	1			1	1	
P70600	Protein-tyrosine kinase 2-beta OS=Rattus norvegicus GN=Ptk2b PE=1 SV=1 - [FAK2_RAT]	115.7	2.413E-5	5.908E-4	3.606E-4	4.834E-4	21.71	24.88	23.58	22.30	0.99	0.99	1.78	1.78	1	1	2	2	1	2	3	3
P70615	Lamin-B1 OS=Rattus norvegicus GN=Lmnb1 PE=1 SV=3 - [LMNB1_RAT]	66.6	3.519E-4	2.166E-4	1.889E-4	1.968E-4	980.40	598.79	489.53	497.13	34.24	23.17	20.10	16.52	17	12	9	9	37	20	14	15
P70618	Mitogen-activated protein kinase 14 OS=Rattus norvegicus GN=Mapk14 PE=2 SV=3 - [MK14_RAT]	41.3	3.434E-5	1.686E-4	2.370E-4	1.842E-4	25.67	177.66	178.52	133.21	2.22	6.39	6.39	10.56	1	2	2	3	1	6	7	8
P70619	Glutathione reductase (Fragment) OS=Rattus norvegicus GN=Gsr PE=2 SV=2 - [GSHR_RAT]	46.3	4.312E-4	2.671E-4	5.744E-4	4.470E-4	344.35	269.49	373.07	273.42	34.91	34.91	36.56	31.13	10	7	9	8	19	12	20	14
P70623	Fatty acid-binding protein, adipocyte OS=Rattus norvegicus GN=Fabp4 PE=1 SV=3 - [FABP4_RAT]	14.7	7.919E-4	6.983E-4	8.347E-4	6.187E-4	880.32	956.48	1124.79	725.99	68.18	59.09	59.09	65.15	9	8	7	8	26	23	22	24
P70645	Bleomycin hydrolase OS=Rattus norvegicus GN=Blmh PE=1 SV=1 - [BLMH_RAT]	52.3	0.000E0	2.231E-4	3.310E-4	2.437E-4		402.35	464.84	237.27		21.81	38.99	27.53		7	11	8		13	20	14
P80020	Gastrotropin OS=Rattus norvegicus OX=10116 GN=Fabp6 PE=1 SV=3 - [FABP6_RAT]	14.5	1.396E-4	4.221E-4	1.894E-4	3.846E-4	92.34	368.54	217.67	356.80	32.81	51.56	40.63	51.56	3	7	4	7	4	12	6	12
P80067	Dipeptidyl peptidase 1 OS=Rattus norvegicus OX=10116 GN=Ctsc PE=1 SV=3 - [CATC_RAT]	52.2	3.187E-4	4.428E-4	6.609E-4	6.065E-4	62.34	407.56	603.69	291.26	7.14	21.65	28.79	24.24	3	7	10	8	7	22	25	17
P80254	D-dopachrome decarboxylase OS=Rattus norvegicus GN=Ddt PE=1 SV=3 - [DOPD_RAT]	13.1	1.635E-3	9.434E-4	8.206E-4	5.489E-4	860.92	700.71	681.99	357.31	95.76	95.76	95.76	65.25	9	9	9	6	29	23	24	14
P80299	Bifunctional epoxide hydrolase 2 OS=Rattus norvegicus OX=10116 GN=Ephx2 PE=1 SV=1 - [HYES_RAT]	62.3	0.000E0	8.014E-5	9.531E-5	6.725E-5		103.09	156.24	64.10		12.27	22.02	6.14		5	9	3		8	12	5
P81155	Voltage-dependent anion-selective channel protein 2 OS=Rattus norvegicus OX=10116 GN=Vdac2 PE=1 SV=2 - [VDAC2_I	31.7	6.932E-5	0.000E0	6.305E-5	7.114E-5	89.70		57.01	56.48	3.39		3.39	6.10	1		1	2	2		1	2
P81718	Tyrosine-protein phosphatase non-receptor type 6 OS=Rattus norvegicus OX=10116 GN=Ptpn6 PE=1 SV=1 - [PTN6_RAT]	69.5	0.000E0	0.000E0	4.131E-5	2.494E-5			149.45	25.37			13.54	6.04			6	3			9	3
P81795	Eukaryotic translation initiation factor 2 subunit 3 OS=Rattus norvegicus GN=Eif2s3 PE=1 SV=2 - [IF2G_RAT]	51.0	0.000E0	1.621E-4	1.243E-4	1.667E-4		345.69	281.36	329.22		34.53	20.76	32.84		11	7	10		18	13	18
P81799	N-acetyl-D-glucosamine kinase OS=Rattus norvegicus GN=Nagk PE=1 SV=4 - [NAGK_RAT]	37.2	5.567E-5	1.009E-4	1.600E-4	8.351E-5	213.17	224.46	391.20	193.69	15.74	28.86	32.94	13.70	5	6	8	3	10	8	14	6
P82450	Sialate O-acetylesterase OS=Rattus norvegicus OX=10116 GN=Siae PE=1 SV=2 - [SIAE_RAT]	60.4	4.480E-5	0.000E0	0.000E0	0.000E0	90.09				12.73				4				7			
P82808	Glutamine--fructose-6-phosphate aminotransferase [isomerizing] 1 OS=Rattus norvegicus GN=Gfpt1 PE=1 SV=3 - [GFPT1	76.8	2.213E-5	2.744E-5	3.735E-5	6.769E-5	26.59	42.13	99.58	158.68	1.62	1.62	2.79	7.93	1	1	2	4	2	1	3	6
P82995	Heat shock protein HSP 90-alpha OS=Rattus norvegicus GN=Hsp90aa1 PE=1 SV=3 - [HS90A_RAT]	84.8	1.082E-3	3.514E-3	3.858E-3	3.009E-3	1092.72	3236.01	4326.37	3598.40	21.69	49.80	59.62	58.25	16	38	45	42	40	128	147	135
P83645	Glutathione peroxidase 2 OS=Rattus norvegicus OX=10116 GN=Gpx2 PE=1 SV=3 - [GPX2_RAT]	22.0	2.355E-5	1.680E-4	2.471E-4	1.819E-4	35.83	423.93	423.79	453.85	10.00	52.63	71.58	47.37	2	9	12	10	2	15	22	19
P83868	Prostaglandin E synthase 3 OS=Rattus norvegicus GN=Ptges3 PE=1 SV=1 - [TEBP_RAT]	18.7	0.000E0	2.102E-4	2.490E-4	1.822E-4		49.32	151.79	128.16		18.75	18.75	18.75		2	2	2		3	6	4
P83883	60S ribosomal protein L36a OS=Rattus norvegicus OX=10116 GN=Rpl36a PE=1 SV=2 - [RL36A_RAT]	12.4	0.000E0	0.000E0	5.593E-5	1.515E-4			75.96	76.85			16.04	16.04			2	2			3	3
P83941	Transcription elongation factor B polypeptide 1 OS=Rattus norvegicus GN=Tceb1 PE=1 SV=1 - [ELOC_RAT]	12.5	0.000E0	4.327E-5	3.710E-5	0.000E0		86.99	100.59			39.29	21.43			3	2			5	4	
P84039	Ectonucleotide pyrophosphatase/phosphodiesterase family member 5 OS=Rattus norvegicus OX=10116 GN=Enpp5 PE=1	54.3	6.766E-4	0.000E0	5.612E-5	0.000E0	218.00		59.42		20.96		8.60		7		2		15		4	
P84092	AP-2 complex subunit mu OS=Rattus norvegicus GN=Ap2m1 PE=1 SV=1 - [AP2M1_RAT]	49.6	0.000E0	7.130E-5	9.292E-5	6.174E-5		117.34	216.80	83.95		11.26	15.63	11.26		4	6	4		7	10	5
P84100	60S ribosomal protein L19 OS=Rattus norvegicus OX=10116 GN=Rpl19 PE=1 SV=1 - [RL19_RAT]	23.5	0.000E0	4.077E-5	2.215E-4	2.311E-4		102.29	370.29	306.14		13.27	13.27	13.78		2	2	3		2	6	7
P84245	Histone H3.3 OS=Rattus norvegicus GN=H3f3b PE=1 SV=2 - [H33_RAT]	15.3	9.327E-4	1.109E-2	4.155E-3	1.199E-2	139.13	471.91	352.52	486.01	10.29	48.53	45.59	45.59	2	6	5	5	7	20	18	22
P84817	Mitochondrial fission 1 protein OS=Rattus norvegicus GN=Fis1 PE=1 SV=1 - [FIS1_RAT]	17.0	4.153E-5	0.000E0	0.000E0	0.000E0	79.41				23.03				3				5			
P84855	Major urinary protein (Fragments) OS=Rattus rattus OX=10117 PE=1 SV=2 - [MUP_RATRT]	5.3	2.952E-5	0.000E0	0.000E0	0.000E0	59.65				36.96				2				4			
P85108	Tubulin beta-2A chain OS=Rattus norvegicus GN=Tubb2a PE=1 SV=1 - [TBB2A_RAT]	49.9	3.773E-4	3.920E-4	1.065E-3	1.251E-3	615.58	693.99	1872.05	2252.26	33.93	29.66	49.66	53.71	12	12	17	19	31	35	72	88
P85125	Polymerase I and transcript release factor OS=Rattus norvegicus GN=Ptrf PE=1 SV=1 - [PTRF_RAT]	43.9	0.000E0	2.109E-4	2.487E-4	1.721E-4		82.80	239.67	157.02		8.93	8.93	8.93		2	2	2		3	6	4
P85515	Alpha-centractin OS=Rattus norvegicus GN=Actr1a PE=1 SV=1 - [ACTZ_RAT]	42.6	0.000E0	2.126E-5	1.181E-4	7.690E-5		33.37	167.41	67.36		4.26	22.07	19.95		1	5	4		2	10	9
P85834	Elongation factor Tu, mitochondrial OS=Rattus norvegicus GN=Tufm PE=1 SV=1 - [EFTU_RAT]	49.5	2.142E-4	1.412E-4	1.434E-4	1.845E-4	173.71	176.24	148.59	334.58	14.16	16.37	16.59	21.46	5	6	5	7	10	9	7	11
P85845	Fascin OS=Rattus norvegicus GN=Fscn1 PE=1 SV=2 - [FSCN1_RAT]	54.5	0.000E0	2.105E-4	3.520E-4	2.632E-4		419.51	505.86	364.89		19.68	29.61	17.24		7	10	6		13	19	11
P85968	6-phosphogluconate dehydrogenase, decarboxylating OS=Rattus norvegicus GN=Pgd PE=1 SV=1 - [6PGD_RAT]	53.2	2.020E-5	4.313E-4	4.885E-4	3.834E-4	30.78	947.32	918.12	681.69	5.18	37.27	44.51	28.57	2	13	16	11	2	30	35	28
P85970	Actin-related protein 2/3 complex subunit 2 OS=Rattus norvegicus GN=Arpc2 PE=1 SV=1 - [ARPC2_RAT]	34.4	0.000E0	2.773E-4	4.068E-4	3.030E-4		282.94	405.72	195.19		28.67	42.67	28.33		9	11	8		17	20	14
P85971	6-phosphogluconolactonase OS=Rattus norvegicus GN=Pgls PE=1 SV=1 - [6PGL_RAT]	27.2	0.000E0	2.529E-4	2.946E-4	2.045E-4		506.14	567.04	295.27		61.09	51.36	42.80		8	6	7		21	16	13
P85972	Vinculin OS=Rattus norvegicus GN=Vcl PE=1 SV=1 - [VINC_RAT]	116.5	1.850E-3	1.691E-3	1.508E-3	1.264E-3	5299.06	5412.37	5544.27	3982.61	57.32	55.72	57.32	55.82	58	55	56	54	196	189	183	151

P85973	Purine nucleoside phosphorylase OS=Rattus norvegicus GN=Pnp PE=1 SV=1 - [PNPH_RAT]	32.3	6.182E-5	8.454E-4	9.542E-4	7.971E-4	99.10	1742.57	2187.82	1075.32	18.69	72.32	72.32	68.51	5	14	14	13	8	71	76	54
P86252	Transcriptional activator protein Pur-alpha (Fragments) OS=Rattus norvegicus GN=Pura PE=1 SV=1 - [PURA_RAT]	15.3	0.000E0	9.531E-5	3.364E-5	6.034E-5		41.63	45.07	52.35		35.51	25.36	23.19		2	2	2		2	3	4
P97519	Hydroxymethylglutaryl-CoA lyase, mitochondrial OS=Rattus norvegicus OX=10116 GN=Hmgcl PE=2 SV=1 - [HMGCL_RAT]	34.2	1.973E-5	1.117E-4	1.528E-4	7.184E-5	30.44	348.01	389.77	208.66	4.31	23.08	25.85	19.08	1	5	6	4	1	11	12	9
P97532	3-mercaptopyruvate sulfurtransferase OS=Rattus norvegicus OX=10116 GN=Mpst PE=1 SV=3 - [THTM_RAT]	32.9	0.000E0	5.719E-5	8.845E-5	1.020E-4		82.96	208.32	223.97		8.42	18.86	12.46		2	5	3		2	9	6
P97536	Cullin-associated NEDD8-dissociated protein 1 OS=Rattus norvegicus GN=Cand1 PE=1 SV=1 - [CAND1_RAT]	136.3	7.335E-5	3.877E-5	1.634E-4	1.461E-4	81.89	102.86	761.44	372.52	1.63	3.66	20.89	15.69	2	4	20	14	4	6	33	21
P97546	Neuroplastin OS=Rattus norvegicus OX=10116 GN=Nptn PE=1 SV=2 - [NPTN_RAT]	43.9	1.593E-4	1.244E-4	1.106E-4	9.280E-5	95.92	94.44	95.24	109.36	2.54	2.54	2.54	7.12	1	1	1	2	2	2	2	3
P97576	GrpE protein homolog 1, mitochondrial OS=Rattus norvegicus GN=Grpel1 PE=1 SV=2 - [GRPE1_RAT]	24.3	2.226E-5	7.938E-5	6.971E-5	5.699E-5	27.00	104.08	109.15	59.28	5.07	17.51	13.82	5.07	1	3	3	1	1	5	5	2
P97584	Prostaglandin reductase 1 OS=Rattus norvegicus GN=Ptgr1 PE=2 SV=3 - [PTGR1_RAT]	35.7	1.030E-3	2.987E-4	3.557E-4	2.790E-4	1222.46	616.03	700.79	516.24	62.92	43.16	40.12	45.59	16	9	8	10	53	21	25	23
P97607	Protein jagged-2 (Fragment) OS=Rattus norvegicus OX=10116 GN=Jag2 PE=2 SV=1 - [JAG2_RAT]	129.6	0.000E0	5.407E-5	2.519E-5	3.552E-5		29.67	34.24	38.40		0.75	2.25	0.75		1	2	1		2	3	2
P97633	Casein kinase I isoform alpha OS=Rattus norvegicus GN=Csnk1a1 PE=2 SV=1 - [KC1A_RAT]	37.5	0.000E0	3.536E-5	4.154E-5	4.320E-5		91.97	43.66	26.65		3.08	3.08	3.08		1	1	1		2	2	2
P97675	Ectonucleotide pyrophosphatase/phosphodiesterase family member 3 OS=Rattus norvegicus GN=Enpp3 PE=1 SV=2 - [EN	99.0	3.000E-3	5.056E-4	6.171E-4	6.616E-4	3110.17	812.45	1166.91	830.70	45.71	24.23	38.06	31.89	30	15	22	19	124	39	58	39
P97690	Structural maintenance of chromosomes protein 3 OS=Rattus norvegicus GN=Smc3 PE=1 SV=1 - [SMC3_RAT]	138.4	0.000E0	2.100E-5	0.000E0	2.293E-5		0.00		30.70		0.59		1.43		1		2		2		3
P97694	Cytohesin-1 OS=Rattus norvegicus GN=Cyth1 PE=1 SV=1 - [CYH1_RAT]	46.2	0.000E0	0.000E0	2.593E-5	2.655E-5			41.95	30.63			2.76	2.76			1	1			2	2
P97697	Inositol monophosphatase 1 OS=Rattus norvegicus GN=Impa1 PE=1 SV=2 - [IMPA1_RAT]	30.5	5.453E-4	3.019E-4	3.426E-4	2.633E-4	584.92	362.26	403.97	291.48	36.82	33.21	33.21	25.99	9	8	8	6	17	14	14	10
P97779	Hyaluronan-mediated motility receptor OS=Rattus norvegicus OX=10116 GN=Hmmr PE=2 SV=1 - [HMMR_RAT]	57.8	0.000E0	1.143E-4	0.000E0	1.067E-4		22.65		27.69		1.41		1.41		1		1		2		2
P97829	Leukocyte surface antigen CD47 OS=Rattus norvegicus GN=Cd47 PE=1 SV=1 - [CD47_RAT]	33.0	8.338E-5	6.038E-5	7.042E-5	3.285E-5	57.60	69.52	57.85	38.14	2.64	2.64	2.64	2.64	1	1	1	1	2	2	2	2
P97834	COP9 signalosome complex subunit 1 OS=Rattus norvegicus GN=Gps1 PE=1 SV=1 - [CSN1_RAT]	53.4	0.000E0	2.664E-5	5.742E-5	4.303E-5		84.45	131.44	35.64		4.46	9.77	1.49		2	5	1		3	7	2
P97840	Galectin-9 OS=Rattus norvegicus OX=10116 GN=Lgals9 PE=2 SV=2 - [LEG9_RAT]	39.9	1.568E-3	6.876E-4	6.060E-4	4.232E-4	1248.13	736.16	854.28	513.79	44.07	42.37	42.09	40.40	12	11	11	10	40	25	28	20
P97852	Peroxisomal multifunctional enzyme type 2 OS=Rattus norvegicus GN=Hsd17b4 PE=1 SV=3 - [DHB4_RAT]	79.4	1.149E-4	1.039E-4	1.328E-4	9.921E-5	308.22	694.62	641.93	450.77	17.96	14.15	15.78	10.61	10	8	9	6	16	17	17	13
P97874	Cyclin-G-associated kinase OS=Rattus norvegicus OX=10116 GN=Gak PE=1 SV=1 - [GAK_RAT]	143.6	0.000E0	1.443E-5	1.277E-5	0.000E0		29.10	28.91			3.52	0.61			3	1			4	2	
P97881	Mucin-13 OS=Rattus norvegicus OX=10116 GN=Muc13 PE=2 SV=1 - [MUC13_RAT]	57.6	5.295E-4	3.589E-4	4.417E-4	3.208E-4	587.14	403.41	408.86	364.23	22.85	24.31	20.66	22.12	11	9	9	8	26	21	20	17
P98089	Intestinal mucin-like protein (Fragment) OS=Rattus norvegicus OX=10116 PE=2 SV=1 - [MUC2L_RAT]	91.4	7.089E-4	6.737E-4	4.529E-4	4.625E-4	692.81	961.84	768.03	753.47	27.48	28.79	23.66	26.88	16	18	15	17	38	39	35	37
Q00438	Polypyrimidine tract-binding protein 1 OS=Rattus norvegicus GN=Ptbp1 PE=1 SV=1 - [PTBP1_RAT]	59.3	4.598E-4	8.444E-4	8.670E-4	9.736E-4	458.87	1167.11	1424.01	1172.56	25.41	42.16	47.21	38.38	10	15	16	13	20	49	62	47
Q00657	Chondroitin sulfate proteoglycan 4 OS=Rattus norvegicus OX=10116 GN=Cspg4 PE=1 SV=2 - [CSPG4_RAT]	251.8	6.112E-5	1.307E-5	1.974E-5	0.000E0	298.32	31.53	79.14		6.36	0.43	1.98		10	1	3		16	1	4	
Q00715	Histone H2B type 1 OS=Rattus norvegicus PE=1 SV=2 - [H2B1_RAT]	14.0	1.209E-3	2.114E-2	9.125E-3	2.794E-2	656.10	7838.95	4548.46	7837.03	40.80	47.20	47.20	47.20	5	7	7	7	25	256	153	259
Q00729	Histone H2B type 1-A OS=Rattus norvegicus GN=Hist1h2ba PE=1 SV=2 - [H2B1A_RAT]	14.2	1.079E-3	2.066E-2	8.393E-3	2.508E-2	218.00	386.81	318.25	437.66	21.26	38.58	38.58	26.77	3	5	5	4	8	18	15	19
Q00981	Ubiquitin carboxyl-terminal hydrolase isozyme L1 OS=Rattus norvegicus GN=Uchl1 PE=1 SV=2 - [UCHL1_RAT]	24.8	2.107E-4	6.288E-4	6.308E-4	3.832E-4	47.49	318.61	318.77	260.75	11.66	39.01	48.43	34.08	2	5	6	5	3	12	13	9
Q01129	Decorin OS=Rattus norvegicus GN=Dcn PE=1 SV=1 - [PGS2_RAT]	39.8	3.276E-5	0.000E0	2.806E-5	3.293E-5	49.68		44.27	51.58	6.21		6.21	6.21	2		2	2	3		3	3
Q01177	Plasminogen OS=Rattus norvegicus GN=Plg PE=2 SV=2 - [PLMN_RAT]	90.5	4.019E-5	2.465E-5	4.194E-5	5.689E-5	50.17	47.82	95.43	80.10	5.67	5.54	7.14	7.39	4	4	5	6	5	4	9	10
Q01205	Dihydrolipoyllysine-residue succinyltransferase component of 2-oxoglutarate dehydrogenase complex, mitochondrial OS=R	48.9	8.352E-4	2.766E-4	3.062E-4	2.056E-4	936.38	599.60	554.95	385.33	20.70	14.32	14.32	14.32	7	6	6	6	22	19	17	15
Q01986	Dual specificity mitogen-activated protein kinase kinase 1 OS=Rattus norvegicus GN=Map2k1 PE=1 SV=2 - [MP2K1_RAT]	43.4	0.000E0	0.000E0	6.535E-5	5.164E-5			71.47	45.82			7.63	5.60			2	1			2	2
Q02253	Methylmalonate-semialdehyde dehydrogenase [acylating], mitochondrial OS=Rattus norvegicus GN=Aldh6a1 PE=1 SV=1 -	57.8	0.000E0	0.000E0	7.596E-5	4.044E-5			249.10	65.55			9.16	7.66			4	3			6	4
Q02401	Lactase-phlorizin hydrolase OS=Rattus norvegicus GN=Lct PE=2 SV=2 - [LPH_RAT]	217.1	2.399E-3	4.942E-4	4.003E-4	5.784E-4	1770.49	1013.57	986.14	922.25	15.56	13.43	13.28	13.38	29	22	23	23	73	47	44	47
Q02589	[Protein ADP-ribosylarginine] hydrolase OS=Rattus norvegicus GN=Adprh PE=1 SV=2 - [ADPRH_RAT]	39.9	0.000E0	0.000E0	0.000E0	3.363E-5				42.50				3.04				1				1
Q02765	Cathepsin S OS=Rattus norvegicus OX=10116 GN=Ctss PE=2 SV=1 - [CATS_RAT]	36.8	1.413E-4	1.772E-4	1.835E-4	1.249E-4	156.99	328.35	271.26	273.63	10.91	19.39	19.39	13.64	2	5	4	3	4	8	7	7
Q02874	Core histone macro-H2A.1 OS=Rattus norvegicus GN=H2afy PE=1 SV=4 - [H2AY_RAT]	39.5	1.861E-5	3.156E-4	1.918E-4	3.749E-4	35.42	347.21	184.06	496.83	2.43	27.76	17.25	32.35	1	8	5	9	1	16	9	18
Q03344	ATPase inhibitor, mitochondrial OS=Rattus norvegicus OX=10116 GN=Atp5if1 PE=3 SV=2 - [ATIF1_RAT]	12.2	0.000E0	0.000E0	0.000E0	5.903E-6				31.31				10.28				1				2
Q03346	Mitochondrial-processing peptidase subunit beta OS=Rattus norvegicus OX=10116 GN=Pmpcb PE=1 SV=3 - [MPPB_RAT]	54.2	0.000E0	8.081E-5	6.600E-5	3.282E-5		122.89	184.53	37.14		13.50	11.04	8.18		5	4	3		6	7	3
Q03555	Gephyrin OS=Rattus norvegicus GN=Gphn PE=1 SV=3 - [GEPH_RAT]	83.2	1.179E-4	4.142E-5	2.968E-5	2.958E-5	232.87	53.16	32.56	59.13	6.25	2.47	8.33	2.86	4	2	3	2	7	2	3	2
Q03626	Murinoglobulin-1 OS=Rattus norvegicus GN=Mug1 PE=2 SV=1 - [MUG1_RAT]	165.2	1.190E-2	1.911E-4	3.415E-4	1.368E-4	3102.24	300.29	527.84	184.05	19.64	10.56	20.51	8.14	26	12	21	9	156	21	33	14
Q04462	Valine--tRNA ligase OS=Rattus norvegicus GN=Vars PE=2 SV=2 - [SYVC_RAT]	140.3	0.000E0	1.484E-4	1.281E-4	9.350E-5		350.00	385.74	274.98		11.79	14.48	9.89		12	13	10		20	19	15
Q04631	Protein farnesyltransferase/geranylgeranyltransferase type-1 subunit alpha OS=Rattus norvegicus GN=Fnta PE=1 SV=1 - [	44.0	0.000E0	3.429E-5	4.862E-5	0.000E0		47.36	89.11			9.02	10.61			3	3			4	6	
Q04931	FACT complex subunit SSRP1 OS=Rattus norvegicus GN=Ssrp1 PE=1 SV=2 - [SSRP1_RAT]	80.9	2.372E-5	4.184E-5	5.070E-5	8.079E-5	45.12	105.44	55.65	45.11	3.24	8.74	4.51	4.09	2	4	2	2	3	6	2	2
Q05962	ADP/ATP translocase 1 OS=Rattus norvegicus GN=Slc25a4 PE=1 SV=3 - [ADT1_RAT]	33.0	6.660E-5	8.603E-5	7.142E-5	1.667E-4	83.92	187.27	71.81	164.06	6.71	18.12	9.40	15.10	2	6	3	5	4	10	4	9
Q05982	Nucleoside diphosphate kinase A OS=Rattus norvegicus GN=Nme1 PE=1 SV=1 - [NDKA_RAT]	17.2	7.943E-4	2.072E-3	2.434E-3	1.827E-3	409.00	862.66	925.27	554.26	55.92	73.68	79.61	73.03	7	10	11	9	20	34	42	25
Q06647	ATP synthase subunit O, mitochondrial OS=Rattus norvegicus GN=Atp5o PE=1 SV=1 - [ATPO_RAT]	23.4	0.000E0	0.000E0	0.000E0	3.661E-5				35.38				14.08				2				3
Q07009	Calpain-2 catalytic subunit OS=Rattus norvegicus GN=Capn2 PE=1 SV=3 - [CAN2_RAT]	79.9	5.836E-5	7.755E-5	2.455E-4	1.476E-4	57.80	92.62	686.64	345.25	7.00	12.57	39.57	20.14	3	7	18	11	4	11	29	14
Q07205	Eukaryotic translation initiation factor 5 OS=Rattus norvegicus GN=Eif5 PE=1 SV=1 - [IF5_RAT]	48.9	0.000E0	7.235E-5	9.877E-5	8.220E-5		142.07	103.11	137.46		15.15	5.59	11.66		5	2	4		7	6	6
Q07439	Heat shock 70 kDa protein 1A/1B OS=Rattus norvegicus GN=Hspa1a PE=2 SV=2 - [HSP71_RAT]	70.1	6.751E-3	3.934E-3	4.190E-3	3.049E-3	822.44	914.66	1001.02	735.57	20.75	23.09	22.00	20.75	11	13	11	10	30	31	33	26
Q07803	Elongation factor G, mitochondrial OS=Rattus norvegicus OX=10116 GN=Gfm1 PE=1 SV=2 - [EFGM_RAT]	83.4	0.000E0	6.127E-5	5.755E-5	0.000E0		104.06	75.27			7.72	5.73			4	3			5	3	
Q07936	Annexin A2 OS=Rattus norvegicus GN=Anxa2 PE=1 SV=2 - [ANXA2_RAT]	38.7	4.062E-4	5.153E-4	7.714E-4	7.192E-4	1125.89	1491.47	1874.62	1343.48	56.05	64.90	64.90	62.83	18	18	18	17	41	51	58	46
Q07969	Platelet glycoprotein 4 OS=Rattus norvegicus OX=10116 GN=Cd36 PE=1 SV=3 - [CD36_RAT]	52.7	1.514E-4	0.000E0	0.000E0	0.000E0	98.06				14.19				5				7			
Q08163	Adenylyl cyclase-associated protein 1 OS=Rattus norvegicus GN=Cap1 PE=1 SV=3 - [CAP1_RAT]	51.6	4.166E-4	8.748E-4	9.445E-4	5.117E-4	1048.56	1597.00	2054.48	1251.66	46.84	52.32	57.17	60.13	17	22	23	22	47	60	76	54
Q08290	Calponin-1 OS=Rattus norvegicus OX=10116 GN=Cnn1 PE=1 SV=1 - [CNN1_RAT]	33.3	1.148E-3	1.966E-3	1.513E-3	1.366E-3	533.78	1109.87	811.56	649.35	32.66	32.66	32.32	34.68	9	9	8	10	24	37	29	29
Q08415	Kynurenine--oxoglutarate transaminase 1, mitochondrial OS=Rattus norvegicus GN=Ccbl1 PE=1 SV=1 - [KAT1_RAT]	51.6	1.166E-3	4.636E-5	5.480E-5	2.748E-5	315.91	84.90	147.43	67.42	19.26	12.25	10.28	7.00	6	3	3	2	11	4	5	3
Q08849	Syntaxin-3 OS=Rattus norvegicus OX=10116 GN=Stx3 PE=1 SV=1 - [STX3_RAT]	33.2	1.237E-4	3.045E-5	0.000E0	2.073E-5	217.70	54.84		42.56	12.46	7.27		7.61	4	2		2	7	3		2
Q09073	ADP/ATP translocase 2 OS=Rattus norvegicus GN=Slc25a5 PE=1 SV=3 - [ADT2_RAT]	32.9	2.843E-4	1.039E-4	4.436E-4	1.667E-4	145.25	206.86	71.81	226.76	16.44	18.12	15.44	15.10	5	6	5	5	8	10	7	9
Q09167	Serine/arginine-rich splicing factor 5 OS=Rattus norvegicus OX=10116 GN=Srsf5 PE=1 SV=1 - [SRSF5_RAT]	30.9	0.000E0	0.000E0	3.463E-4	5.220E-4			180.89	246.99			14.13	21.19			3	5			5	9
Q10728	Protein phosphatase 1 regulatory subunit 12A OS=Rattus norvegicus GN=Ppp1r12a PE=1 SV=2 - [MYPT1_RAT]	115.2	0.000E0	2.105E-4	3.704E-4	3.351E-4		73.17	133.94	37.31		3.88	6.69	3.39		4	6	4		6	11	7
Q10758	Keratin, type II cytoskeletal 8 OS=Rattus norvegicus GN=Krt8 PE=1 SV=3 - [K2C8_RAT]	54.0	4.655E-3	2.003E-3	2.373E-3	2.339E-3	2981.69	2954.91	3112.46	2469.07	47.62	50.52	56.31	49.28	30	34	32	31	114	107	105	97
Q1JU68	Eukaryotic translation initiation factor 3 subunit A OS=Rattus norvegicus GN=Eif3a PE=2 SV=2 - [EIF3A_RAT]	163.1	1.937E-5	6.905E-5	1.366E-4	7.559E-5	29.15	151.02	392.67	138.41	1.11	6.57	8.71	4.58	2	7	9	5	3	10	18	8
Q1LZ53	DNA (cytosine-5)-methyltransferase 3A OS=Rattus norvegicus OX=10116 GN=Dnmt3a PE=1 SV=1 - [DNM3A_RAT]	101.6	0.000E0	0.000E0	0.000E0	1.228E-5				26.09				1.87				2				2
Q27W01	RNA-binding protein 8A OS=Rattus norvegicus OX=10116 GN=Rbm8a PE=1 SV=1 - [RBM8A_RAT]	19.9	1.193E-4	1.461E-4	2.036E-4	1.817E-4	109.21	194.00	173.24	171.83	21.84	17.24	17.24	22.41	3	2	2	3	6	4	4	5
Q27W02	Protein mago nashi homolog OS=Rattus norvegicus GN=Magoh PE=2 SV=1 - [MGN_RAT]	17.2	4.793E-4	5.585E-4	4.657E-4	5.920E-4	241.30	247.73	220.24	314.48	46.58	42.47	45.21	54.79	6	5	5	6	12	12	12	14
Q2A865	Sodium-dependent neutral amino acid transporter B(0)AT1 OS=Rattus norvegicus OX=10116 GN=Slc6a19 PE=1 SV=2 - [S	71.2	9.168E-5	0.000E0	0.000E0	0.000E0	38.77				3.15				2				2			
Q2LAP6	Testin OS=Rattus norvegicus GN=Tes PE=1 SV=1 - [TES_RAT]	47.6	0.000E0	6.730E-5	1.437E-4	1.462E-4		156.00	290.13	216.36		10.26	21.24	12.65		3	6	4		7	11	9
Q2PQA9	Kinesin-1 heavy chain OS=Rattus norvegicus GN=Kif5b PE=1 SV=1 - [KINH_RAT]	109.5	1.048E-4	6.146E-5	6.631E-5	5.972E-5	327.68	176.67	312.14	100.65	13.60	4.78	9.45	6.13	10	3	7	4	15	6	10	7
Q2TL32	E3 ubiquitin-protein ligase UBR4 OS=Rattus norvegicus GN=Ubr4 PE=1 SV=2 - [UBR4_RAT]	573.4	1.219E-4	1.132E-4	0.000E0	1.038E-4	22.97	37.44		21.74	0.19	0.35		0.35	1	2		2	1	3		2
Q32KJ6	N-acetylgalactosamine-6-sulfatase OS=Rattus norvegicus GN=Galns PE=1 SV=1 - [GALNS_RAT]	58.3	2.223E-3	3.050E-4	5.303E-4	5.902E-4	1896.47	579.75	1073.64	920.05	50.38	33.21	48.85	48.85	21	13	20	19	85	24	42	39
Q32PX2	Aminoacyl tRNA synthase complex-interacting multifunctional protein 2 OS=Rattus norvegicus GN=Aimp2 PE=2 SV=1 - [A	35.4	0.000E0	0.000E0	4.759E-5	4.339E-5			137.82	27.54			14.37	2.81			3	1			5	1
Q32PX7	Far upstream element-binding protein 1 OS=Rattus norvegicus GN=Fubp1 PE=1 SV=1 - [FUBP1_RAT]	67.2	3.582E-5	1.088E-4	8.897E-5	2.159E-4	51.60	217.40	186.25	493.89	2.03	9.08	10.64	20.66	1	4	5	11	2	8	8	22
Q3B7D0	Oxygen-dependent coproporphyrinogen-III oxidase, mitochondrial OS=Rattus norvegicus GN=Cpox PE=1 SV=1 - [HEM6_I	49.2	2.598E-4	7.720E-5	1.006E-4	5.680E-5	631.07	245.87	289.13	102.64	41.53	19.19	18.96	8.80	15	7	6	3	30	10	11	5
Q3B7D1	Ubiquitin-conjugating enzyme E2 Z OS=Rattus norvegicus GN=Ube2z PE=2 SV=2 - [UBE2Z_RAT]	38.3	0.000E0	0.000E0	3.740E-5	1.615E-5			114.53	45.65			7.02	5.06			3	2			4	3
Q3B8Q1	Nucleolar RNA helicase 2 OS=Rattus norvegicus OX=10116 GN=Ddx21 PE=2 SV=1 - [DDX21_RAT]	85.9	0.000E0	4.701E-5	3.903E-5	7.141E-5		79.38	51.42	197.32		5.88	4.35	8.70		4	3	6		5	5	9
Q3B8Q2	Eukaryotic initiation factor 4A-III OS=Rattus norvegicus GN=Eif4a3 PE=1 SV=1 - [IF4A3_RAT]	46.8	0.000E0	5.939E-4	6.381E-4	5.941E-4		370.89	551.42	476.02		29.44	30.90	35.28		11	11	13		19	24	24
Q3KR86	MICOS complex subunit Mic60 (Fragment) OS=Rattus norvegicus GN=Immt PE=1 SV=1 - [MIC60_RAT]	67.1	2.276E-4	1.027E-4	6.821E-5	5.701E-5	567.14	396.05	382.58	236.96	11.17	11.33	7.06	7.06	5	5	3	3	12	8	6	4
Q3KRC5	tRNA-dihydrouridine(47) synthase [NAD(P)(+)]-like OS=Rattus norvegicus OX=10116 GN=Dus3l PE=2 SV=1 - [DUS3L_RA	71.5	0.000E0	2.573E-5	0.000E0	0.000E0		34.53				5.78				4				4		
Q3KRC6	Volume-regulated anion channel subunit LRRC8E OS=Rattus norvegicus OX=10116 GN=Lrrc8e PE=1 SV=1 - [LRC8E_RAT]	90.4	3.686E-4	0.000E0	0.000E0	0.000E0	23.84				3.52				1				1			
Q3KRD8	Eukaryotic translation initiation factor 6 OS=Rattus norvegicus GN=Eif6 PE=1 SV=1 - [IF6_RAT]	26.6	0.000E0	7.082E-4	8.368E-4	4.605E-4		667.52	606.80	344.54		51.02	51.02	36.33		7	7	5		18	17	11
Q3MIE4	Synaptic vesicle membrane protein VAT-1 homolog OS=Rattus norvegicus GN=Vat1 PE=1 SV=1 - [VAT1_RAT]	43.1	2.270E-4	2.067E-4	3.920E-4	2.147E-4	311.68	574.52	968.28	439.11	23.02	30.20	37.87	31.68	6	9	12	9	12	18	30	18
Q3SWT4	Protein IWS1 homolog OS=Rattus norvegicus GN=Iws1 PE=1 SV=1 - [IWS1_RAT]	85.4	0.000E0	0.000E0	0.000E0	1.860E-5				23.48				1.18				1				1
Q3SWU3	Heterogeneous nuclear ribonucleoprotein D-like OS=Rattus norvegicus GN=Hnrnpdl PE=2 SV=1 - [HNRDL_RAT]	35.3	1.016E-3	1.640E-3	1.422E-3	2.259E-3	141.01	239.23	303.75	256.92	5.59	21.74	21.74	21.74	2	6	6	6	6	13	14	14
Q3T1I4	Protein PRRC1 OS=Rattus norvegicus GN=Prrc1 PE=2 SV=1 - [PRRC1_RAT]	46.3	1.231E-4	3.272E-5	5.084E-5	0.000E0	182.59	63.66	108.60		8.80	3.16	12.42		3	1	3		5	2	5	
Q3T1J1	Eukaryotic translation initiation factor 5A-1 OS=Rattus norvegicus GN=Eif5a PE=1 SV=3 - [IF5A1_RAT]	16.8	1.305E-4	3.933E-4	4.144E-4	3.457E-4	280.49	590.12	638.88	352.31	36.36	66.88	61.69	46.10	5	9	8	6	12	25	21	16
Q3T1J9	MOB kinase activator 1A OS=Rattus norvegicus GN=Mob1a PE=2 SV=3 - [MOB1A_RAT]	25.1	1.483E-4	1.044E-4	4.273E-5	1.233E-4	98.79	51.43	59.09	67.08	10.65	5.09	20.83	5.09	2	1	3	1	3	2	3	2
Q3T1K5	F-actin-capping protein subunit alpha-2 OS=Rattus norvegicus GN=Capza2 PE=1 SV=1 - [CAZA2_RAT]	32.9	4.317E-5	3.872E-4	5.366E-4	3.458E-4	33.83	557.18	865.91	728.63	9.79	58.74	65.38	54.20	2	10	11	9	2	16	24	21
Q3T1L0	Aldehyde dehydrogenase family 16 member A1 OS=Rattus norvegicus GN=Aldh16a1 PE=2 SV=1 - [A16A1_RAT]	85.4	0.000E0	4.993E-5	1.078E-4	6.074E-5		374.45	560.54	351.18		6.23	16.83	10.72		4	8	6		7	15	10
Q496Z9	TRMT1-like protein OS=Rattus norvegicus GN=Trmt1l PE=2 SV=1 - [TRM1L_RAT]	80.1	0.000E0	1.692E-4	1.047E-4	7.360E-5		42.27	58.45	33.19		1.24	1.24	1.24		1	1	1		2	2	2
Q497B0	Omega-amidase NIT2 OS=Rattus norvegicus GN=Nit2 PE=1 SV=1 - [NIT2_RAT]	30.7	0.000E0	9.171E-5	8.450E-5	8.484E-5		209.08	201.12	158.87		33.33	35.51	15.22		7	6	4		9	9	6
Q497C3	UPF0585 protein C16orf13 homolog OS=Rattus norvegicus PE=2 SV=2 - [CP013_RAT]	22.6	0.000E0	2.203E-5	1.856E-5	0.000E0		27.61	31.79			13.73	8.82			2	1			2	1	
Q498D9	PDZ domain-containing protein GIPC2 OS=Rattus norvegicus GN=Gipc2 PE=2 SV=1 - [GIPC2_RAT]	34.2	7.760E-5	0.000E0	9.040E-5	1.356E-4	21.82		63.93	57.24	9.24		12.10	11.78	2		2	2	2		4	2
Q498E0	Thioredoxin domain-containing protein 12 OS=Rattus norvegicus GN=Txndc12 PE=2 SV=2 - [TXD12_RAT]	19.0	1.410E-4	2.178E-4	2.381E-4	1.992E-4	181.72	209.17	209.82	45.40	26.47	22.35	31.18	8.82	4	3	4	1	7	6	6	2
Q498M4	WD repeat-containing protein 5 OS=Rattus norvegicus GN=Wdr5 PE=1 SV=1 - [WDR5_RAT]	36.6	0.000E0	1.103E-4	1.640E-4	4.104E-5		89.20	80.00	27.27		9.88	5.69	7.78		3	2	2		4	3	2
Q498R7	UPF0587 protein C1orf123 homolog OS=Rattus norvegicus PE=2 SV=1 - [CA123_RAT]	18.1	1.016E-4	2.536E-4	2.103E-4	2.081E-4	140.59	190.28	204.06	196.07	21.88	41.88	36.88	36.88	3	6	5	5	6	10	9	10
Q499T2	Gamma-interferon-inducible lysosomal thiol reductase OS=Rattus norvegicus GN=Ifi30 PE=2 SV=1 - [GILT_RAT]	27.6	3.311E-4	1.880E-4	1.704E-4	1.944E-4	133.11	107.52	85.27	78.72	15.32	5.65	15.32	4.44	5	3	4	2	8	7	8	5
Q4AE70	Histone-arginine methyltransferase CARM1 OS=Rattus norvegicus GN=Carm1 PE=1 SV=1 - [CARM1_RAT]	70.3	0.000E0	6.536E-5	4.959E-5	4.795E-5		67.48	75.68	69.59		5.68	7.68	9.22		3	3	4		4	4	7
Q4AEF8	Coatomer subunit gamma-1 OS=Rattus norvegicus GN=Copg1 PE=2 SV=1 - [COPG1_RAT]	97.6	3.383E-5	1.204E-4	1.135E-4	6.384E-5	86.47	161.11	362.46	122.70	2.75	12.59	23.68	6.29	2	8	15	5	3	12	22	8
Q4FZT8	SPRY domain-containing protein 4 OS=Rattus norvegicus OX=10116 GN=Spryd4 PE=2 SV=1 - [SPRY4_RAT]	23.2	4.616E-5	4.538E-5	0.000E0	0.000E0	24.12	43.73			4.83	4.83			1	1			1	2		
Q4FZT9	26S proteasome non-ATPase regulatory subunit 2 OS=Rattus norvegicus GN=Psmd2 PE=2 SV=1 - [PSMD2_RAT]	100.1	0.000E0	1.062E-4	2.163E-4	1.623E-4		352.38	755.56	468.55		15.97	26.10	18.94		11	17	13		19	36	24
Q4FZU2	Keratin, type II cytoskeletal 6A OS=Rattus norvegicus GN=Krt6a PE=1 SV=1 - [K2C6A_RAT]	59.2	1.075E-2	2.663E-3	3.789E-3	5.335E-3	1101.92	646.49	595.63	674.36	19.38	13.95	11.59	11.59	12	9	7	7	43	27	21	25
Q4FZU6	Annexin A8 OS=Rattus norvegicus GN=Anxa8 PE=1 SV=1 - [ANXA8_RAT]	36.7	2.649E-5	7.556E-5	7.394E-5	4.452E-5	24.50	206.37	231.48	141.53	6.12	12.23	22.63	9.48	2	2	5	2	2	4	8	4
Q4FZV0	Beta-mannosidase OS=Rattus norvegicus GN=Manba PE=2 SV=1 - [MANBA_RAT]	101.2	6.116E-5	0.000E0	0.000E0	0.000E0	145.46				6.13				6				11			
Q4FZY0	EF-hand domain-containing protein D2 OS=Rattus norvegicus GN=Efhd2 PE=1 SV=1 - [EFHD2_RAT]	26.7	3.859E-4	2.904E-4	2.236E-4	2.315E-4	439.52	510.22	381.55	347.98	37.24	35.15	29.29	28.45	9	9	7	7	20	20	15	13
Q4G009	Malignant T-cell-amplified sequence 1 OS=Rattus norvegicus GN=Mcts1 PE=2 SV=1 - [MCTS1_RAT]	20.5	0.000E0	5.437E-5	5.033E-5	5.350E-5		30.93	49.36	35.49		9.34	9.34	9.34		1	1	1		2	3	1
Q4G061	Eukaryotic translation initiation factor 3 subunit B OS=Rattus norvegicus GN=Eif3b PE=1 SV=1 - [EIF3B_RAT]	90.9	1.176E-4	1.641E-4	1.365E-4	1.259E-4	245.44	478.59	458.49	409.35	12.92	18.32	18.82	14.30	9	12	12	10	11	20	21	16
Q4G063	Cysteine-rich with EGF-like domain protein 2 OS=Rattus norvegicus GN=Creld2 PE=1 SV=1 - [CREL2_RAT]	38.2	4.340E-5	2.974E-5	3.051E-5	2.320E-5	38.06	66.56	48.07	59.05	6.02	8.31	8.31	8.31	2	2	2	2	3	3	3	3
Q4G075	Leukocyte elastase inhibitor A OS=Rattus norvegicus GN=Serpinb1a PE=1 SV=1 - [ILEUA_RAT]	42.7	1.876E-3	1.496E-3	1.332E-3	1.398E-3	2292.70	2389.84	2604.39	2287.55	55.94	55.67	55.67	55.67	24	23	22	22	83	81	76	78
Q4KLF8	Actin-related protein 2/3 complex subunit 5 OS=Rattus norvegicus GN=Arpc5 PE=1 SV=3 - [ARPC5_RAT]	16.3	0.000E0	6.862E-5	0.000E0	0.000E0		30.45				8.61				1				1		
Q4KLH4	Paraspeckle component 1 OS=Rattus norvegicus OX=10116 GN=Pspc1 PE=1 SV=1 - [PSPC1_RAT]	58.7	0.000E0	4.191E-5	5.260E-5	8.325E-5		21.98	75.20	79.82		4.41	8.05	7.47		2	4	4		3	4	6
Q4KLJ8	Phosducin-like protein 3 OS=Rattus norvegicus GN=Pdcl3 PE=1 SV=1 - [PDCL3_RAT]	27.7	0.000E0	9.727E-5	2.058E-4	7.569E-5		64.56	69.22	50.20		9.58	12.92	11.25		2	3	3		2	5	5
Q4KLL0	Transcription elongation factor A protein 1 OS=Rattus norvegicus GN=Tcea1 PE=1 SV=1 - [TCEA1_RAT]	33.9	6.993E-5	1.312E-4	8.822E-5	9.544E-5	170.28	290.36	275.86	228.91	11.63	14.95	15.28	15.28	3	4	5	5	5	8	8	8
Q4KLN6	Ribonucleoside-diphosphate reductase subunit M2 OS=Rattus norvegicus GN=Rrm2 PE=2 SV=1 - [RIR2_RAT]	45.0	6.238E-5	0.000E0	0.000E0	0.000E0	72.35				12.05				4				5			
Q4KLN7	ADP-ribosylation factor GTPase-activating protein 3 OS=Rattus norvegicus GN=Arfgap3 PE=1 SV=1 - [ARFG3_RAT]	57.6	4.588E-5	2.957E-5	3.175E-5	1.838E-5	40.53	41.44	33.02	32.30	4.57	3.05	1.52	1.52	3	2	1	1	5	3	2	1
Q4KLZ6	Triokinase/FMN cyclase OS=Rattus norvegicus GN=Tkfc PE=1 SV=1 - [TKFC_RAT]	59.4	5.579E-5	1.848E-4	2.355E-4	6.180E-5	147.64	362.91	365.18	92.63	18.86	35.29	25.95	3.46	7	12	9	2	8	21	19	3
Q4KM35	Proteasome subunit beta type-10 OS=Rattus norvegicus GN=Psmb10 PE=2 SV=1 - [PSB10_RAT]	29.0	1.382E-5	5.988E-5	8.071E-5	0.000E0	37.24	173.57	172.75		12.45	20.15	20.15		2	4	4		2	6	7	
Q4KM49	Tyrosine--tRNA ligase, cytoplasmic OS=Rattus norvegicus GN=Yars PE=2 SV=3 - [SYYC_RAT]	59.1	0.000E0	1.335E-4	1.200E-4	1.020E-4		325.69	235.15	236.70		28.41	25.95	23.86		12	12	11		18	16	15
Q4KM62	Palmdelphin OS=Rattus norvegicus GN=Palmd PE=1 SV=1 - [PALMD_RAT]	62.4	4.909E-5	0.000E0	0.000E0	0.000E0	33.74				1.45				1				2			
Q4KM65	Cleavage and polyadenylation specificity factor subunit 5 OS=Rattus norvegicus GN=Nudt21 PE=2 SV=1 - [CPSF5_RAT]	26.2	6.979E-5	1.041E-4	1.335E-4	1.793E-4	83.54	100.12	160.85	147.30	23.35	23.35	30.40	43.17	3	3	5	7	5	7	10	10
Q4KM73	UMP-CMP kinase OS=Rattus norvegicus GN=Cmpk1 PE=1 SV=2 - [KCY_RAT]	22.2	3.544E-4	9.593E-4	8.417E-4	6.150E-4	394.03	836.05	684.54	520.75	43.88	64.29	58.67	58.67	7	11	10	9	16	34	28	27
Q4KM74	Vesicle-trafficking protein SEC22b OS=Rattus norvegicus GN=Sec22b PE=1 SV=3 - [SC22B_RAT]	24.7	0.000E0	3.427E-5	4.122E-5	0.000E0		68.61	70.95			8.84	21.40			2	4			3	6	
Q4KM92	tRNA pseudouridine synthase A OS=Rattus norvegicus OX=10116 GN=Pus1 PE=1 SV=2 - [TRUA_RAT]	47.5	0.000E0	2.276E-5	0.000E0	0.000E0		28.43				2.60				1				1		
Q4KMA2	UV excision repair protein RAD23 homolog B OS=Rattus norvegicus GN=Rad23b PE=1 SV=1 - [RD23B_RAT]	43.5	1.223E-4	0.000E0	0.000E0	0.000E0	115.70				9.16				4				9			
Q4QQR9	Protein MEMO1 OS=Rattus norvegicus GN=Memo1 PE=1 SV=1 - [MEMO1_RAT]	33.7	0.000E0	4.160E-5	4.495E-5	3.546E-5		108.48	94.47	84.10		14.14	21.21	14.14		4	5	4		7	7	6
Q4QQT3	CUGBP Elav-like family member 1 OS=Rattus norvegicus GN=Celf1 PE=2 SV=1 - [CELF1_RAT]	52.2	0.000E0	9.875E-5	6.827E-5	5.462E-5		187.53	113.35	82.75		15.61	8.62	6.98		6	3	3		12	6	5
Q4QQT4	Serine/threonine-protein phosphatase 2A 65 kDa regulatory subunit A beta isoform OS=Rattus norvegicus GN=Ppp2r1b PE	66.0	5.272E-5	6.516E-5	1.495E-4	9.198E-5	53.32	204.73	376.69	259.83	5.82	6.99	6.99	6.99	2	3	3	3	6	9	12	8
Q4QQW4	Histone deacetylase 1 OS=Rattus norvegicus GN=Hdac1 PE=1 SV=1 - [HDAC1_RAT]	55.1	0.000E0	9.543E-5	5.433E-5	1.048E-4		127.16	110.28	87.17		10.79	8.30	10.37		4	3	3		5	4	5
Q4QQW8	Putative phospholipase B-like 2 OS=Rattus norvegicus GN=Plbd2 PE=1 SV=2 - [PLBL2_RAT]	65.4	6.340E-5	5.744E-5	6.049E-5	6.790E-5	116.18	213.19	153.41	217.12	8.03	10.26	11.79	11.28	3	4	5	4	5	7	7	6
Q4QR75	Exosome complex component RRP45 OS=Rattus norvegicus GN=Exosc9 PE=1 SV=1 - [EXOS9_RAT]	48.9	0.000E0	7.028E-5	0.000E0	0.000E0		102.99				5.26				2				3		
Q4QR85	Methylosome protein 50 OS=Rattus norvegicus GN=Wdr77 PE=1 SV=1 - [MEP50_RAT]	37.1	0.000E0	9.251E-5	1.065E-4	5.839E-5		78.06	76.34	64.31		15.79	11.40	6.43		4	3	2		5	5	3
Q4QRB4	Tubulin beta-3 chain OS=Rattus norvegicus GN=Tubb3 PE=1 SV=1 - [TBB3_RAT]	50.4	3.773E-4	3.920E-4	9.023E-4	1.066E-3	441.15	470.34	958.59	1519.28	17.11	15.78	28.22	34.89	7	7	11	14	17	21	42	58
Q4V7A0	WD repeat-containing protein 61 OS=Rattus norvegicus GN=Wdr61 PE=1 SV=1 - [WDR61_RAT]	33.7	0.000E0	4.379E-5	0.000E0	0.000E0		22.34				13.44				3				4		
Q4V7C6	GMP synthase [glutamine-hydrolyzing] OS=Rattus norvegicus GN=Gmps PE=1 SV=1 - [GUAA_RAT]	76.7	0.000E0	1.000E-4	9.735E-5	0.000E0		45.59	62.41			8.08	6.49			4	3			5	7	
Q4V7C7	Actin-related protein 3 OS=Rattus norvegicus GN=Actr3 PE=1 SV=1 - [ARP3_RAT]	47.3	0.000E0	1.877E-4	3.631E-4	3.088E-4		232.40	645.12	536.00		31.10	56.46	38.04		9	15	11		16	30	21
Q4V7E8	Leucine-rich repeat flightless-interacting protein 2 OS=Rattus norvegicus OX=10116 GN=Lrrfip2 PE=1 SV=1 - [LRRF2_RAT	49.7	1.036E-3	1.972E-4	1.993E-4	4.921E-4	40.63	23.53	20.96	21.91	1.60	3.20	3.20	1.60	1	2	2	1	4	3	3	2
Q4V8C3	Echinoderm microtubule-associated protein-like 1 OS=Rattus norvegicus OX=10116 GN=Eml1 PE=1 SV=2 - [EMAL1_RAT]	89.7	0.000E0	2.205E-5	2.481E-5	2.181E-5		71.84	67.72	59.39		2.83	2.95	1.60		2	2	1		3	3	1
Q4V8F9	Hydroxysteroid dehydrogenase-like protein 2 OS=Rattus norvegicus OX=10116 GN=Hsdl2 PE=2 SV=1 - [HSDL2_RAT]	58.3	0.000E0	1.352E-4	2.290E-5	1.467E-4	24.61	36.49	25.30	35.29	1.72	1.72	1.34	1.72	1	1	1	1	1	2	1	2
Q4V8H8	EH domain-containing protein 2 OS=Rattus norvegicus GN=Ehd2 PE=1 SV=1 - [EHD2_RAT]	61.2	0.000E0	0.000E0	1.043E-4	5.658E-4			53.63	49.58			3.31	3.31			2	2			3	4
Q4V8K2	Beta-catenin-like protein 1 OS=Rattus norvegicus GN=Ctnnbl1 PE=1 SV=1 - [CTBL1_RAT]	64.9	0.000E0	4.942E-5	7.857E-5	5.231E-5		65.83	80.41	74.53		2.66	2.66	2.66		2	2	2		4	4	3
Q4V8K5	BRO1 domain-containing protein BROX OS=Rattus norvegicus GN=Brox PE=2 SV=1 - [BROX_RAT]	46.2	0.000E0	5.424E-5	4.132E-5	9.504E-5		32.60	71.39	25.12		7.30	10.71	2.68		2	2	1		2	3	1
Q4VSI4	Ubiquitin carboxyl-terminal hydrolase 7 OS=Rattus norvegicus GN=Usp7 PE=1 SV=1 - [UBP7_RAT]	128.3	0.000E0	1.256E-4	1.092E-4	9.605E-5		226.77	234.10	106.92		13.33	15.50	7.16		10	11	5		13	16	7
Q505J8	Phenylalanine--tRNA ligase alpha subunit OS=Rattus norvegicus GN=Farsa PE=1 SV=1 - [SYFA_RAT]	57.7	3.192E-5	1.406E-4	7.832E-5	5.492E-5	69.89	377.79	273.51	265.78	7.09	19.29	11.22	9.65	3	6	4	4	7	12	8	9
Q561R9	Beta-lactamase-like protein 2 OS=Rattus norvegicus GN=Lactb2 PE=2 SV=1 - [LACB2_RAT]	32.5	4.110E-5	8.134E-5	7.649E-5	7.754E-5	32.52	194.51	164.68	199.59	12.50	41.67	29.51	30.56	2	8	6	7	3	13	10	10
Q561S0	NADH dehydrogenase [ubiquinone] 1 alpha subcomplex subunit 10, mitochondrial OS=Rattus norvegicus GN=Ndufa10 PE	40.5	1.645E-5	2.235E-5	0.000E0	2.537E-5	26.70	38.00		30.60	1.69	1.69		7.89	1	1		2	1	2		2
Q562A2	Zinc finger RNA-binding protein OS=Rattus norvegicus GN=Zfr PE=1 SV=2 - [ZFR_RAT]	116.7	0.000E0	2.680E-5	2.114E-5	0.000E0		55.81	30.79			1.49	2.33			1	2			2	2	
Q562B5	Serine/threonine-protein phosphatase PGAM5, mitochondrial OS=Rattus norvegicus OX=10116 GN=Pgam5 PE=2 SV=1 - [	32.0	3.620E-5	0.000E0	0.000E0	0.000E0	45.54			26.46	3.13			3.47	1			1	2			1
Q562C4	Methyltransferase-like protein 7B OS=Rattus norvegicus OX=10116 GN=Mettl7b PE=1 SV=1 - [MET7B_RAT]	27.9	0.000E0	3.972E-5	5.751E-5	3.877E-5		39.79	85.61	24.39		15.98	26.23	7.79		2	5	2		3	6	3
Q562C9	1,2-dihydroxy-3-keto-5-methylthiopentene dioxygenase OS=Rattus norvegicus OX=10116 GN=Adi1 PE=2 SV=1 - [MTND_	21.4	1.826E-4	0.000E0	0.000E0	0.000E0	89.64				41.34				6				10			
Q566C7	Diphosphoinositol polyphosphate phosphohydrolase 1 OS=Rattus norvegicus GN=Nudt3 PE=1 SV=1 - [NUDT3_RAT]	19.1	0.000E0	3.143E-5	3.491E-5	2.164E-5		144.39	119.29	125.41		13.69	8.33	13.69		2	1	2		3	2	3
Q566E5	KDEL motif-containing protein 2 OS=Rattus norvegicus GN=Kdelc2 PE=2 SV=1 - [KDEL2_RAT]	58.7	0.000E0	5.475E-5	6.531E-5	7.552E-5		62.44	65.52	61.36		6.89	6.89	3.35		2	2	1		3	4	2
Q566Q8	UPF0696 protein C11orf68 homolog OS=Rattus norvegicus OX=10116 GN=Bles03 PE=1 SV=2 - [CK068_RAT]	31.4	0.000E0	0.000E0	4.811E-5	5.074E-5			117.93	130.69			10.27	14.73			2	3			3	4
Q568Z6	IST1 homolog OS=Rattus norvegicus GN=Ist1 PE=2 SV=1 - [IST1_RAT]	39.9	0.000E0	2.181E-5	4.108E-5	5.987E-5		56.22	161.29	204.63		9.56	12.30	12.30		3	4	4		5	7	7

Q569B7	RWD domain-containing protein 4 OS=Rattus norvegicus OX=10116 GN=Rwdd4 PE=2 SV=1 - [RWDD4_RAT]	21.1	0.000E0	1.454E-5	0.000E0	0.000E0		44.89		#01_sham		4.79				1				2		
Q56A27	Nuclear cap-binding protein subunit 1 OS=Rattus norvegicus GN=Ncbp1 PE=1 SV=1 - [NCBP1_RAT]	91.9	0.000E0	3.189E-5	2.213E-5	1.162E-5		94.31	100.40	39.29		8.86	7.85	2.41		5	5	2		8	8	4
Q5BJ92	Serine/threonine-protein phosphatase 4 catalytic subunit OS=Rattus norvegicus GN=Ppp4c PE=2 SV=1 - [PP4C_RAT]	35.0	2.206E-4	1.041E-4	1.352E-4	1.498E-4	134.14	243.36	261.55	314.37	4.89	20.20	15.31	18.89	2	4	4	5	5	10	8	12
Q5BJP3	Ubiquitin-fold modifier 1 OS=Rattus norvegicus GN=Ufm1 PE=3 SV=1 - [UFM1_RAT]	9.1	1.648E-4	1.173E-4	1.217E-4	1.048E-4	95.40	147.95	134.93	46.93	68.24	58.82	58.82	58.82	5	4	4	3	9	9	8	4
Q5BJQ6	Cleavage stimulation factor subunit 1 OS=Rattus norvegicus GN=Cstf1 PE=2 SV=1 - [CSTF1_RAT]	48.4	0.000E0	5.255E-5	6.718E-5	3.071E-5		90.82	118.70	85.17		6.96	11.83	11.83		2	3	3		3	5	4
Q5BJY6	Putative N-acetylglucosamine-6-phosphate deacetylase OS=Rattus norvegicus GN=Amdhd2 PE=3 SV=2 - [NAGA_RAT]	43.5	7.659E-4	1.155E-4	1.768E-4	1.291E-4	1134.27	321.00	582.38	299.16	62.59	43.28	43.28	31.05	16	9	9	7	49	14	18	14
Q5BJY9	Keratin, type I cytoskeletal 18 OS=Rattus norvegicus GN=Krt18 PE=1 SV=3 - [K1C18_RAT]	47.7	6.713E-3	2.153E-3	2.643E-3	2.527E-3	1534.01	1328.57	1460.39	1126.44	57.21	47.99	50.83	46.57	24	19	21	19	58	44	48	41
Q5BK06	Cyclin-D1-binding protein 1 OS=Rattus norvegicus OX=10116 GN=Ccndbp1 PE=2 SV=1 - [CCDB1_RAT]	39.0	8.450E-6	0.000E0	0.000E0	0.000E0	36.98				1.97				1				1			
Q5BK81	Prostaglandin reductase 2 OS=Rattus norvegicus GN=Ptgr2 PE=2 SV=2 - [PTGR2_RAT]	38.1	0.000E0	3.440E-5	3.338E-5	0.000E0		55.73	42.62			7.12	8.83			2	2			3	3	
Q5EGY4	Synaptobrevin homolog YKT6 OS=Rattus norvegicus GN=Ykt6 PE=1 SV=1 - [YKT6_RAT]	22.4	2.815E-5	0.000E0	0.000E0	0.000E0	27.98				4.04				1				1			
Q5EGZ1	Angiotensin-converting enzyme 2 OS=Rattus norvegicus OX=10116 GN=Ace2 PE=1 SV=1 - [ACE2_RAT]	92.4	0.000E0	3.088E-4	3.552E-4	4.552E-4		1015.08	1120.06	1186.38		29.07	33.04	34.53		16	17	20		38	40	44
Q5EZ72	Ectonucleotide pyrophosphatase/phosphodiesterase family member 7 OS=Rattus norvegicus OX=10116 GN=Enpp7 PE=1	49.8	3.363E-5	0.000E0	0.000E0	0.000E0	31.83				2.28				1				1			
Q5FVF9	Biotinidase OS=Rattus norvegicus GN=Btd PE=2 SV=1 - [BTD_RAT]	58.0	0.000E0	1.587E-5	0.000E0	0.000E0		26.71				2.30				1				1		
Q5FVH2	Phospholipase D3 OS=Rattus norvegicus GN=Pld3 PE=2 SV=1 - [PLD3_RAT]	54.4	1.171E-4	0.000E0	4.223E-5	4.028E-5	174.26		140.16	149.11	11.27		8.81	9.02	4		3	3	7		4	4
Q5FVI6	V-type proton ATPase subunit C 1 OS=Rattus norvegicus GN=Atp6v1c1 PE=2 SV=1 - [VATC1_RAT]	43.9	0.000E0	5.674E-5	3.978E-5	3.255E-5		44.10	101.93	49.01		2.62	9.42	11.52		1	3	3		2	8	4
Q5FVM4	Non-POU domain-containing octamer-binding protein OS=Rattus norvegicus GN=Nono PE=1 SV=3 - [NONO_RAT]	54.9	0.000E0	3.381E-4	3.694E-4	4.836E-4		274.92	364.73	432.38		11.76	18.91	20.59		6	8	9		13	16	21
Q5FVQ4	Malectin OS=Rattus norvegicus OX=10116 GN=Mlec PE=2 SV=1 - [MLEC_RAT]	32.4	1.346E-4	3.839E-5	2.990E-5	2.487E-5	151.74	84.98	83.87	41.41	26.80	16.84	14.43	2.41	6	4	3	1	10	4	5	2
Q5FWT7	Ubiquitin-like domain-containing CTD phosphatase 1 OS=Rattus norvegicus GN=Ublcp1 PE=2 SV=1 - [UBCP1_RAT]	36.8	0.000E0	4.779E-5	5.183E-5	0.000E0		36.71	58.22			3.14	11.95			1	3			2	5	
Q5HZE4	Methylthioribose-1-phosphate isomerase OS=Rattus norvegicus GN=Mri1 PE=2 SV=1 - [MTNA_RAT]	39.6	0.000E0	1.928E-5	0.000E0	0.000E0		31.68				3.52				1				1		
Q5HZV9	Protein phosphatase 1 regulatory subunit 7 OS=Rattus norvegicus GN=Ppp1r7 PE=1 SV=1 - [PP1R7_RAT]	41.3	0.000E0	8.986E-5	9.484E-5	6.555E-5		487.39	354.68	274.78		26.94	24.17	21.11		8	8	7		15	12	11
Q5HZW7	Placenta-expressed transcript 1 protein OS=Rattus norvegicus OX=10116 GN=Plet1 PE=2 SV=1 - [PLET1_RAT]	26.8	3.100E-4	0.000E0	0.000E0	0.000E0	80.41				6.67				2				4			
Q5HZY2	GTP-binding protein SAR1b OS=Rattus norvegicus GN=Sar1b PE=2 SV=1 - [SAR1B_RAT]	22.4	1.572E-4	0.000E0	0.000E0	0.000E0	90.24				25.76				3				4			
Q5I0D1	Glyoxalase domain-containing protein 4 OS=Rattus norvegicus GN=Glod4 PE=1 SV=1 - [GLOD4_RAT]	33.2	1.267E-4	3.383E-4	3.128E-4	2.805E-4	244.24	1021.30	859.46	665.63	30.20	62.42	60.40	58.39	7	16	17	14	12	34	32	26
Q5I0D7	Xaa-Pro dipeptidase OS=Rattus norvegicus GN=Pepd PE=2 SV=1 - [PEPD_RAT]	54.7	3.783E-3	9.667E-4	1.153E-3	8.841E-4	2791.42	1318.19	1834.92	1357.01	55.08	42.07	50.00	41.26	22	15	18	16	86	36	44	40
Q5I0E7	Transmembrane emp24 domain-containing protein 9 OS=Rattus norvegicus OX=10116 GN=Tmed9 PE=1 SV=1 - [TMED9_	27.0	7.309E-5	7.442E-5	6.613E-5	1.344E-4	73.84	84.01	112.66	77.25	4.68	4.68	11.91	10.21	1	1	2	2	2	2	3	3
Q5I0G4	Glycine--tRNA ligase (Fragment) OS=Rattus norvegicus GN=Gars PE=1 SV=1 - [SYG_RAT]	72.0	0.000E0	8.592E-5	1.578E-4	9.694E-5		171.80	405.35	260.26		14.91	21.51	11.46		7	12	6		11	21	11
Q5I0P2	Glycine cleavage system H protein, mitochondrial OS=Rattus norvegicus OX=10116 GN=Gcsh PE=2 SV=1 - [GCSH_RAT]	18.5	0.000E0	3.117E-5	0.000E0	3.520E-5		23.53		48.07		17.65		17.65		1		1		1		2
Q5M7U6	Actin-related protein 2 OS=Rattus norvegicus GN=Actr2 PE=1 SV=1 - [ARP2_RAT]	44.7	4.247E-5	1.420E-4	1.650E-4	1.306E-4	40.52	173.51	689.88	387.97	4.57	14.97	26.65	21.57	2	5	8	8	3	11	21	19
Q5M7V8	Thyroid hormone receptor-associated protein 3 OS=Rattus norvegicus OX=10116 GN=Thrap3 PE=1 SV=1 - [TR150_RAT]	108.2	1.399E-4	2.103E-4	2.440E-4	2.240E-4	25.00	25.18	26.72	23.37	0.63	1.58	1.58	4.21	1	2	2	4	1	3	3	4
Q5M819	Phosphoserine phosphatase OS=Rattus norvegicus GN=Psph PE=2 SV=1 - [SERB_RAT]	25.0	0.000E0	3.645E-5	3.802E-5	0.000E0		44.53	35.25			3.56	3.56			1	1			1	1	
Q5M823	NudC domain-containing protein 2 OS=Rattus norvegicus OX=10116 GN=Nudcd2 PE=2 SV=1 - [NUDC2_RAT]	17.7	0.000E0	3.979E-5	0.000E0	0.000E0		24.98				12.74				2				3		
Q5M827	Pirin OS=Rattus norvegicus GN=Pir PE=1 SV=1 - [PIR_RAT]	32.2	0.000E0	9.008E-5	1.734E-4	1.071E-4		62.44	233.36	85.59		16.84	16.84	12.71		4	4	3		7	11	7
Q5M939	Histone acetyltransferase type B catalytic subunit OS=Rattus norvegicus GN=Hat1 PE=2 SV=1 - [HAT1_RAT]	49.2	0.000E0	2.791E-5	3.382E-5	0.000E0		24.19	24.59			2.39	2.39			1	1			2	1	
Q5M9G3	Caprin-1 OS=Rattus norvegicus GN=Caprin1 PE=1 SV=2 - [CAPR1_RAT]	78.1	4.214E-4	3.591E-4	3.236E-4	2.683E-4	620.88	576.52	583.94	515.24	16.97	16.97	16.97	15.98	10	10	10	9	22	20	23	19
Q5M9G9	FAST kinase domain-containing protein 4 OS=Rattus norvegicus OX=10116 GN=Tbrg4 PE=2 SV=1 - [FAKD4_RAT]	71.1	0.000E0	3.542E-5	1.627E-5	2.456E-5		60.39	30.96	40.07		2.38	1.27	1.27		2	1	1		3	1	2
Q5PPH0	Enolase-phosphatase E1 OS=Rattus norvegicus GN=Enoph1 PE=2 SV=1 - [ENOPH_RAT]	28.9	2.085E-5	7.970E-5	7.495E-5	6.242E-5	28.41	120.21	159.47	152.67	3.07	23.75	16.09	16.09	1	4	3	3	1	6	7	7
Q5PPJ4	Deoxyhypusine hydroxylase OS=Rattus norvegicus OX=10116 GN=Dohh PE=2 SV=1 - [DOHH_RAT]	33.1	0.000E0	1.906E-5	2.620E-5	0.000E0		20.19	44.74			6.62	7.95			2	2			2	2	
Q5PQP1	RNA-binding motif, single-stranded-interacting protein 1 OS=Rattus norvegicus OX=10116 GN=Rbms1 PE=2 SV=1 - [RBM	44.0	0.000E0	6.445E-5	5.406E-5	4.553E-5		37.74	47.61	39.65		3.72	3.72	7.44		1	1	2		1	1	3
Q5RJP0	Aldose reductase-related protein 1 OS=Rattus norvegicus GN=Akr1b7 PE=1 SV=1 - [ALD1_RAT]	36.1	1.440E-4	1.277E-4	2.021E-4	1.456E-4	62.42	98.75	205.21	139.06	2.53	7.91	16.77	14.24	1	3	5	4	2	8	11	9
Q5RJR2	Twinfilin-1 OS=Rattus norvegicus GN=Twf1 PE=2 SV=1 - [TWF1_RAT]	40.1	3.738E-5	2.233E-4	2.366E-4	1.677E-4	26.29	312.01	395.03	222.65	4.57	34.00	43.14	29.71	2	8	10	8	4	15	17	14
Q5RJR8	Leucine-rich repeat-containing protein 59 OS=Rattus norvegicus GN=Lrrc59 PE=1 SV=1 - [LRC59_RAT]	34.8	0.000E0	4.903E-5	6.718E-5	3.053E-5		61.88	134.34	61.95		16.61	19.87	13.36		4	5	4		4	8	5
Q5RK00	39S ribosomal protein L46, mitochondrial OS=Rattus norvegicus OX=10116 GN=Mrpl46 PE=2 SV=1 - [RM46_RAT]	31.7	0.000E0	8.733E-5	7.325E-5	4.115E-5		47.69	58.58	33.55		11.91	11.91	7.22		3	3	2		5	6	3
Q5RK09	Eukaryotic translation initiation factor 3 subunit G OS=Rattus norvegicus GN=Eif3g PE=1 SV=1 - [EIF3G_RAT]	35.6	1.104E-4	7.803E-5	5.493E-5	5.615E-5	88.61	68.75	48.13	35.66	2.81	2.81	2.81	2.81	1	1	1	1	2	2	1	1
Q5RK30	Ribosome maturation protein SBDS OS=Rattus norvegicus GN=Sbds PE=2 SV=1 - [SBDS_RAT]	28.7	0.000E0	7.322E-5	5.722E-5	6.719E-5		127.09	101.28	104.66		14.40	7.20	7.20		3	2	2		6	4	4
Q5RKH0	Putative oxidoreductase GLYR1 OS=Rattus norvegicus OX=10116 GN=Glyr1 PE=1 SV=1 - [GLYR1_RAT]	60.4	0.000E0	2.243E-5	7.005E-5	4.479E-5		35.85	83.40	30.89		4.35	3.08	4.71		2	1	2		2	2	3
Q5RKH1	Serine/threonine-protein kinase PRP4 homolog OS=Rattus norvegicus OX=10116 GN=Prpf4b PE=1 SV=1 - [PRP4B_RAT]	116.9	0.000E0	0.000E0	0.000E0	1.495E-5				26.44				1.09				1				2
Q5RKH7	Solute carrier family 35 member F6 OS=Rattus norvegicus OX=10116 GN=Slc35f6 PE=2 SV=1 - [S35F6_RAT]	41.1	2.535E-5	0.000E0	0.000E0	0.000E0	55.10				6.45				2				2			
Q5RKI0	WD repeat-containing protein 1 OS=Rattus norvegicus GN=Wdr1 PE=1 SV=3 - [WDR1_RAT]	66.1	1.175E-3	9.032E-4	1.098E-3	7.352E-4	1374.33	2021.28	2681.09	1378.56	34.32	61.06	72.61	52.64	18	25	30	21	46	65	76	46
Q5RKI1	Eukaryotic initiation factor 4A-II OS=Rattus norvegicus GN=Eif4a2 PE=1 SV=1 - [IF4A2_RAT]	46.4	5.166E-5	9.090E-4	1.016E-3	9.578E-4	57.90	750.89	1064.66	788.95	5.90	35.87	40.29	34.64	2	14	15	14	3	32	40	35
Q5SGE0	Leucine-rich PPR motif-containing protein, mitochondrial OS=Rattus norvegicus GN=Lrpprc PE=1 SV=1 - [LPPRC_RAT]	156.6	3.421E-5	1.491E-4	1.661E-4	1.262E-4	67.16	998.46	1221.42	786.88	2.59	18.46	26.44	15.52	3	21	28	17	3	36	46	33
Q5U1X1	Oligoribonuclease, mitochondrial OS=Rattus norvegicus GN=Rexo2 PE=2 SV=1 - [ORN_RAT]	26.7	0.000E0	5.114E-5	5.824E-5	4.991E-5		81.48	103.19	48.26		16.88	16.88	9.28		3	3	2		5	6	3
Q5U1Z2	Trafficking protein particle complex subunit 3 OS=Rattus norvegicus GN=Trappc3 PE=2 SV=1 - [TPPC3_RAT]	20.3	6.718E-5	1.064E-4	1.089E-4	9.833E-5	32.53	53.83	42.55	55.77	4.44	4.44	4.44	4.44	1	1	1	1	2	2	2	2
Q5U216	ATP-dependent RNA helicase DDX39A OS=Rattus norvegicus GN=Ddx39a PE=2 SV=1 - [DX39A_RAT]	49.1	2.513E-4	4.776E-4	7.792E-4	9.993E-4	237.85	491.21	824.04	815.71	25.29	30.44	37.47	41.92	9	11	14	15	14	28	37	40
Q5U2M6	DDB1- and CUL4-associated factor 8 OS=Rattus norvegicus GN=Dcaf8 PE=1 SV=1 - [DCAF8_RAT]	66.1	0.000E0	1.411E-4	1.523E-4	0.000E0		35.01	49.65			3.89	3.89			3	3			3	6	
Q5U2Q3	Ester hydrolase C11orf54 homolog OS=Rattus norvegicus PE=1 SV=1 - [CK054_RAT]	35.0	0.000E0	7.559E-5	5.149E-5	4.683E-5		128.37	78.80	59.59		6.67	11.75	8.89		2	3	2		3	5	4
Q5U2Q7	Eukaryotic peptide chain release factor subunit 1 OS=Rattus norvegicus GN=Etf1 PE=2 SV=3 - [ERF1_RAT]	49.0	1.358E-4	1.651E-4	1.402E-4	1.029E-4	115.93	370.08	347.17	236.06	16.02	25.17	25.17	24.71	6	9	9	8	7	19	16	14
Q5U2R0	Methionine adenosyltransferase 2 subunit beta OS=Rattus norvegicus GN=Mat2b PE=2 SV=1 - [MAT2B_RAT]	37.4	6.333E-5	1.648E-4	1.844E-4	9.200E-5	139.93	237.97	311.04	89.19	16.17	21.26	23.35	16.17	4	5	5	4	6	9	9	6
Q5U2R7	LRP chaperone MESD OS=Rattus norvegicus OX=10116 GN=Mesd PE=2 SV=1 - [MESD_RAT]	25.2	6.863E-5	9.296E-5	9.909E-5	1.326E-4	68.16	50.34	41.80	44.99	7.59	7.59	7.59	7.59	2	1	1	1	4	1	1	2
Q5U2U2	Crk-like protein OS=Rattus norvegicus GN=Crkl PE=1 SV=1 - [CRKL_RAT]	33.8	8.407E-5	8.981E-5	0.000E0	9.806E-5	27.58	50.97		33.66	8.58	13.20		4.95	2	2		1	2	3		2
Q5U2U7	mRNA cap guanine-N7 methyltransferase OS=Rattus norvegicus GN=Rnmt PE=1 SV=1 - [MCES_RAT]	52.8	0.000E0	6.054E-5	3.545E-5	5.473E-5		76.35	51.40	59.34		13.02	10.20	11.93		5	3	4		6	3	6
Q5U2V4	Phospholipase B-like 1 OS=Rattus norvegicus OX=10116 GN=Plbd1 PE=2 SV=1 - [PLBL1_RAT]	63.0	1.656E-4	7.092E-5	4.538E-5	5.485E-5	349.99	271.82	183.44	165.03	14.55	11.27	9.27	10.36	7	5	4	4	15	10	5	7
Q5U2Y1	General transcription factor II-I OS=Rattus norvegicus GN=Gtf2i PE=1 SV=2 - [GTF2I_RAT]	110.1	0.000E0	1.266E-4	0.000E0	5.722E-5		33.92		45.67		2.04		5.41		2		4		2		6
Q5U2Z3	Nucleosome assembly protein 1-like 4 OS=Rattus norvegicus GN=Nap1l4 PE=1 SV=1 - [NP1L4_RAT]	43.9	0.000E0	0.000E0	4.596E-5	5.185E-5			149.25	69.19			17.36	7.77			4	3			6	5
Q5U300	Ubiquitin-like modifier-activating enzyme 1 OS=Rattus norvegicus GN=Uba1 PE=1 SV=1 - [UBA1_RAT]	117.7	1.012E-4	4.332E-4	4.672E-4	3.393E-4	158.42	2198.87	2554.56	1758.87	5.48	43.86	49.62	48.02	4	31	34	33	7	71	82	70
Q5XFW8	Protein SEC13 homolog OS=Rattus norvegicus GN=Sec13 PE=1 SV=1 - [SEC13_RAT]	35.5	1.588E-4	2.196E-4	2.594E-4	1.039E-4	222.13	347.13	409.59	198.67	17.70	26.40	45.96	15.22	4	6	8	3	6	12	17	5
Q5XFX0	Transgelin-2 OS=Rattus norvegicus GN=Tagln2 PE=2 SV=1 - [TAGL2_RAT]	22.4	7.727E-4	1.348E-3	9.558E-4	5.797E-4	1159.57	2152.66	1743.79	1205.43	67.34	71.36	76.88	76.38	13	13	14	13	37	60	51	40
Q5XHY5	Threonine--tRNA ligase, cytoplasmic OS=Rattus norvegicus GN=Tars PE=1 SV=1 - [SYTC_RAT]	80.5	2.263E-4	2.587E-4	2.922E-4	2.432E-4	564.49	996.13	1194.51	857.60	22.16	33.96	47.19	34.10	13	20	27	19	29	39	49	34
Q5XHZ0	Heat shock protein 75 kDa, mitochondrial OS=Rattus norvegicus GN=Trap1 PE=1 SV=1 - [TRAP1_RAT]	80.4	3.296E-4	1.622E-3	1.857E-3	1.470E-3	131.92	378.00	490.31	397.79	1.98	6.80	12.04	6.66	1	4	6	4	2	9	12	10
Q5XI01	La-related protein 7 OS=Rattus norvegicus OX=10116 GN=Larp7 PE=1 SV=2 - [LARP7_RAT]	64.9	0.000E0	4.266E-5	3.181E-5	4.509E-5		64.34	32.74	58.62		3.68	3.68	5.95		2	2	3		4	3	4
Q5XI07	Lipoma-preferred partner homolog OS=Rattus norvegicus OX=10116 GN=Lpp PE=1 SV=1 - [LPP_RAT]	68.2	0.000E0	1.033E-4	0.000E0	5.283E-5		30.25		25.53		1.42		7.28		1		3		2		4
Q5XI22	Acetyl-CoA acetyltransferase, cytosolic OS=Rattus norvegicus GN=Acat2 PE=1 SV=1 - [THIC_RAT]	41.1	1.529E-3	5.243E-4	6.605E-4	4.220E-4	726.34	460.45	663.86	311.73	76.07	39.55	46.35	36.52	15	9	10	8	41	22	27	14
Q5XI29	Cleavage and polyadenylation specificity factor subunit 7 OS=Rattus norvegicus OX=10116 GN=Cpsf7 PE=1 SV=1 - [CPSF	51.0	0.000E0	2.497E-5	3.601E-5	0.000E0		43.51	20.99			4.11	1.73			2	1			3	1	
Q5XI32	F-actin-capping protein subunit beta OS=Rattus norvegicus GN=Capzb PE=1 SV=1 - [CAPZB_RAT]	30.6	1.326E-4	6.611E-4	6.184E-4	5.249E-4	142.60	835.49	998.26	643.61	22.79	59.93	55.15	56.99	4	14	12	13	8	32	33	30
Q5XI60	Receptor expression-enhancing protein 6 OS=Rattus norvegicus GN=Reep6 PE=2 SV=1 - [REEP6_RAT]	23.3	2.137E-4	2.767E-5	8.556E-5	4.575E-5	139.04	43.32	70.89	41.73	6.16	6.16	6.16	6.16	1	1	1	1	4	2	3	2
Q5XI73	Rho GDP-dissociation inhibitor 1 OS=Rattus norvegicus GN=Arhgdia PE=1 SV=1 - [GDIR1_RAT]	23.4	3.298E-3	2.409E-3	2.278E-3	1.718E-3	1236.75	1407.49	1478.51	1115.48	47.06	55.39	55.39	55.39	8	10	10	10	44	49	50	44
Q5XI78	2-oxoglutarate dehydrogenase, mitochondrial OS=Rattus norvegicus GN=Ogdh PE=1 SV=1 - [ODO1_RAT]	116.2	1.835E-4	2.196E-4	2.713E-4	1.770E-4	358.68	810.32	1051.86	478.73	22.09	35.48	31.77	21.90	15	29	27	17	29	51	54	31
Q5XI81	Fragile X mental retardation syndrome-related protein 1 OS=Rattus norvegicus OX=10116 GN=Fxr1 PE=1 SV=1 - [FXR1_I	63.9	2.090E-3	1.256E-3	0.000E0	1.493E-3	98.85	73.69		37.80	5.99	7.75		5.28	3	4		3	5	9		5
Q5XI96	Ribonuclease H2 subunit B OS=Rattus norvegicus GN=Rnaseh2b PE=2 SV=1 - [RNH2B_RAT]	34.6	0.000E0	0.000E0	1.042E-5	1.825E-5			0.00	14.59			3.58	3.58			1	1			2	1
Q5XIB2	Peptidyl-prolyl cis-trans isomerase CWC27 homolog OS=Rattus norvegicus GN=Cwc27 PE=1 SV=1 - [CWC27_RAT]	53.3	2.354E-5	7.723E-5	5.211E-5	0.000E0	30.08	118.54	40.18		2.78	7.69	2.78		1	3	1		2	4	1	
Q5XID1	Anamorsin OS=Rattus norvegicus GN=Ciapin1 PE=2 SV=1 - [CPIN1_RAT]	33.0	0.000E0	1.220E-5	0.000E0	0.000E0		22.72				3.88				1				1		
Q5XIE0	Acidic leucine-rich nuclear phosphoprotein 32 family member E OS=Rattus norvegicus OX=10116 GN=Anp32e PE=2 SV=1	29.4	0.000E0	2.990E-4	3.343E-4	2.442E-4		502.87	484.05	412.53		30.62	30.62	24.03		7	7	5		16	15	11
Q5XIE3	39S ribosomal protein L11, mitochondrial OS=Rattus norvegicus OX=10116 GN=Mrpl11 PE=2 SV=1 - [RM11_RAT]	22.4	0.000E0	0.000E0	2.010E-5	2.810E-5			28.08	41.06			4.43	4.43			1	1			1	2
Q5XIE6	3-hydroxyisobutyryl-CoA hydrolase, mitochondrial OS=Rattus norvegicus GN=Hibch PE=1 SV=2 - [HIBCH_RAT]	43.0	0.000E0	8.947E-5	8.992E-5	7.837E-5		251.10	340.41	147.20		20.26	29.09	10.13		6	8	3		10	11	6
Q5XIF6	Tubulin alpha-4A chain OS=Rattus norvegicus GN=Tuba4a PE=2 SV=1 - [TBA4A_RAT]	49.9	3.636E-4	5.114E-4	1.142E-3	1.839E-3	662.82	785.49	1753.53	1746.64	27.01	29.02	42.86	44.64	10	9	14	15	29	29	59	64
Q5XIG0	ADP-ribose pyrophosphatase, mitochondrial OS=Rattus norvegicus OX=10116 GN=Nudt9 PE=2 SV=1 - [NUDT9_RAT]	38.5	0.000E0	7.186E-5	4.489E-5	0.000E0		86.11	33.56			12.00	2.57			4	1			6	1	
Q5XIG6	N-acetylgalactosamine kinase OS=Rattus norvegicus OX=10116 GN=Galk2 PE=2 SV=1 - [GALK2_RAT]	50.2	0.000E0	0.000E0	3.900E-5	4.541E-5			69.52	40.21			5.46	5.46			2	2			3	4
Q5XIG8	Serine-threonine kinase receptor-associated protein OS=Rattus norvegicus GN=Strap PE=1 SV=1 - [STRAP_RAT]	38.4	1.293E-4	1.095E-4	1.408E-4	6.058E-5	232.71	382.25	490.31	145.72	35.14	30.00	30.00	21.43	9	7	7	5	13	13	13	6
Q5XIH7	Prohibitin-2 OS=Rattus norvegicus GN=Phb2 PE=1 SV=1 - [PHB2_RAT]	33.3	0.000E0	0.000E0	0.000E0	1.164E-5				55.50				10.03				2				3
Q5XIM7	Lysine--tRNA ligase OS=Rattus norvegicus OX=10116 GN=Kars PE=1 SV=1 - [SYK_RAT]	71.6	0.000E0	3.651E-4	4.658E-4	2.123E-4		337.80	491.09	400.19		14.70	28.12	26.20		8	13	12		15	23	26
Q5XIM9	T-complex protein 1 subunit beta OS=Rattus norvegicus GN=Cct2 PE=1 SV=3 - [TCPB_RAT]	57.4	9.734E-5	1.539E-4	3.535E-4	1.940E-4	161.18	355.52	1196.02	712.80	9.53	28.97	43.55	33.27	4	10	19	14	6	16	43	30
Q5XIP6	Flap endonuclease 1 OS=Rattus norvegicus GN=Fen1 PE=2 SV=1 - [FEN1_RAT]	42.6	0.000E0	5.390E-5	3.980E-5	5.101E-5		42.78	25.76	32.21		2.89	7.37	7.37		1	2	2		2	5	3
Q60587	Trifunctional enzyme subunit beta, mitochondrial OS=Rattus norvegicus OX=10116 GN=Hadhb PE=1 SV=1 - [ECHB_RAT]	51.4	3.417E-5	4.977E-5	8.512E-5	6.736E-5	40.05	107.97	181.31	126.05	1.89	8.42	13.47	13.26	1	3	5	5	2	4	9	9
Q62625	Microtubule-associated proteins 1A/1B light chain 3B OS=Rattus norvegicus GN=Map1lc3b PE=1 SV=3 - [MLP3B_RAT]	16.4	1.607E-4	1.233E-4	4.620E-5	9.265E-5	95.18	60.73	24.94	26.71	19.72	19.72	4.93	9.86	3	3	1	2	5	6	2	3
Q62635	Mucin-2 (Fragment) OS=Rattus norvegicus OX=10116 GN=Muc2 PE=1 SV=1 - [MUC2_RAT]	165.9	1.241E-3	7.678E-4	5.347E-4	6.008E-4	1971.72	2168.40	1448.13	1577.92	31.26	32.91	30.27	32.45	37	41	33	37	91	98	75	87
Q62638	Golgi apparatus protein 1 OS=Rattus norvegicus GN=Glg1 PE=1 SV=1 - [GSLG1_RAT]	133.5	1.365E-4	7.686E-5	7.261E-5	5.298E-5	566.37	192.88	229.56	40.55	12.30	6.92	9.99	1.71	12	7	9	2	21	9	12	4
Q62651	Delta(3,5)-Delta(2,4)-dienoyl-CoA isomerase, mitochondrial OS=Rattus norvegicus GN=Ech1 PE=1 SV=2 - [ECH1_RAT]	36.1	2.959E-4	8.364E-5	1.166E-4	7.145E-5	534.43	137.80	184.77	51.44	41.90	15.90	18.35	13.76	11	5	6	4	24	9	10	6
Q62658	Peptidyl-prolyl cis-trans isomerase FKBP1A OS=Rattus norvegicus GN=Fkbp1a PE=1 SV=3 - [FKB1A_RAT]	11.9	4.367E-4	5.464E-4	4.784E-4	3.660E-4	439.85	530.92	576.11	476.49	41.67	71.30	71.30	41.67	5	6	5	4	14	17	16	15
Q62667	Major vault protein OS=Rattus norvegicus GN=Mvp PE=1 SV=4 - [MVP_RAT]	95.7	2.131E-4	4.141E-4	3.616E-4	3.003E-4	143.79	760.94	1155.32	767.37	7.90	28.92	43.32	36.93	6	16	25	21	9	36	53	42
Q62696	Disks large homolog 1 OS=Rattus norvegicus GN=Dlg1 PE=1 SV=1 - [DLG1_RAT]	100.5	1.459E-4	1.226E-4	7.294E-5	3.086E-5	221.49	181.12	186.31	67.07	10.65	16.79	13.72	2.41	6	9	7	2	11	13	12	3
Q62698	Cytoplasmic dynein 1 light intermediate chain 2 OS=Rattus norvegicus GN=Dync1li2 PE=1 SV=1 - [DC1L2_RAT]	54.7	0.000E0	2.476E-5	4.650E-5	0.000E0		21.68	74.19			4.43	4.43			2	2			2	3	
Q62736	Non-muscle caldesmon OS=Rattus norvegicus OX=10116 GN=Cald1 PE=1 SV=1 - [CALD1_RAT]	60.5	2.073E-5	1.693E-4	4.310E-5	3.158E-5	33.61	231.36	90.67	51.53	3.20	10.73	8.10	6.40	2	7	5	3	2	13	8	5
Q62745	CD81 antigen OS=Rattus norvegicus GN=Cd81 PE=1 SV=1 - [CD81_RAT]	25.9	0.000E0	0.000E0	9.703E-5	9.304E-5			20.30	39.52			3.39	3.39			1	1			1	2
Q62773	Sodium/nucleoside cotransporter 2 OS=Rattus norvegicus OX=10116 GN=Slc28a2 PE=1 SV=1 - [S28A2_RAT]	72.6	9.172E-5	0.000E0	1.080E-5	0.000E0	107.06		30.40		5.61		1.37		4		1		7		1	
Q62774	Unconventional myosin-Ia (Fragment) OS=Rattus norvegicus OX=10116 GN=Myo1a PE=2 SV=1 - [MYO1A_RAT]	97.1	0.000E0	6.780E-5	3.111E-5	3.112E-5		109.19	67.29	40.11		8.55	5.58	2.02		6	4	2		7	5	2
Q62780	Probable ATP-dependent RNA helicase DDX46 OS=Rattus norvegicus OX=10116 GN=Ddx46 PE=1 SV=1 - [DDX46_RAT]	117.3	0.000E0	0.000E0	7.311E-6	1.630E-5			20.99	35.46			0.97	3.20			1	3			1	3
Q62786	Prostaglandin F2 receptor negative regulator OS=Rattus norvegicus GN=Ptgfrn PE=1 SV=1 - [FPRP_RAT]	98.7	4.168E-5	0.000E0	0.000E0	0.000E0	84.98				2.73				2				3			
Q62812	Myosin-9 OS=Rattus norvegicus GN=Myh9 PE=1 SV=3 - [MYH9_RAT]	226.2	9.315E-5	5.252E-4	4.301E-4	7.014E-4	227.65	2861.77	2570.49	3280.29	3.93	27.69	30.09	37.02	7	47	50	63	11	96	91	133
Q62826	Heterogeneous nuclear ribonucleoprotein M OS=Rattus norvegicus GN=Hnrnpm PE=1 SV=4 - [HNRPM_RAT]	73.7	1.745E-4	5.862E-4	3.959E-4	5.499E-4	312.39	473.00	502.25	526.29	8.84	11.30	14.64	17.10	5	7	8	9	10	16	14	21
Q62839	Golgin subfamily A member 2 OS=Rattus norvegicus OX=10116 GN=Golga2 PE=1 SV=4 - [GOGA2_RAT]	112.8	8.098E-6	0.000E0	0.000E0	0.000E0	21.68				1.50				1				1			
Q62847	Gamma-adducin OS=Rattus norvegicus GN=Add3 PE=1 SV=2 - [ADDG_RAT]	78.8	0.000E0	2.916E-5	4.398E-5	4.361E-5		27.16	52.57	48.98		1.42	1.42	3.69		1	1	2		1	2	3
Q62848	ADP-ribosylation factor GTPase-activating protein 1 OS=Rattus norvegicus GN=Arfgap1 PE=1 SV=1 - [ARFG1_RAT]	45.4	1.422E-5	0.000E0	0.000E0	0.000E0	30.45				6.75				2				2			
Q62867	Gamma-glutamyl hydrolase OS=Rattus norvegicus GN=Ggh PE=1 SV=1 - [GGH_RAT]	35.8	3.600E-4	1.062E-4	1.013E-4	2.199E-4	201.36	74.32	34.58	137.28	21.14	12.62	7.26	15.14	7	4	3	5	9	5	4	7
Q62871	Cytoplasmic dynein 1 intermediate chain 2 OS=Rattus norvegicus GN=Dync1i2 PE=1 SV=1 - [DC1I2_RAT]	71.1	0.000E0	5.171E-5	6.079E-5	2.095E-5		102.24	166.09	23.41		3.76	11.60	7.99		1	4	2		2	7	3
Q62902	Protein ERGIC-53 OS=Rattus norvegicus OX=10116 GN=Lman1 PE=1 SV=1 - [LMAN1_RAT]	57.9	6.562E-5	9.200E-5	0.000E0	0.000E0	39.37	71.20			10.06	6.77			3	2			5	3		
Q62915	Peripheral plasma membrane protein CASK OS=Rattus norvegicus GN=Cask PE=1 SV=1 - [CSKP_RAT]	103.2	1.057E-5	0.000E0	0.000E0	0.000E0	28.02				0.77				1				2			
Q62920	PDZ and LIM domain protein 5 OS=Rattus norvegicus GN=Pdlim5 PE=1 SV=2 - [PDLI5_RAT]	63.2	1.313E-4	1.351E-4	1.669E-4	1.873E-4	261.17	270.15	210.51	199.88	8.97	14.55	10.83	10.49	4	7	6	6	7	13	9	11
Q62940	E3 ubiquitin-protein ligase NEDD4 OS=Rattus norvegicus GN=Nedd4 PE=1 SV=1 - [NEDD4_RAT]	102.3	7.482E-5	1.009E-4	1.291E-4	7.346E-5	172.63	256.73	266.74	175.28	6.43	12.74	11.50	8.23	5	9	8	7	7	13	16	10
Q62952	Dihydropyrimidinase-related protein 3 OS=Rattus norvegicus GN=Dpysl3 PE=1 SV=1 - [DPYL3_RAT]	61.9	0.000E0	2.206E-4	3.358E-4	2.144E-4		861.56	1033.22	643.70		28.77	42.11	36.67		12	16	14		28	35	26
Q62967	Diphosphomevalonate decarboxylase OS=Rattus norvegicus GN=Mvd PE=1 SV=1 - [MVD1_RAT]	43.9	0.000E0	8.014E-5	4.614E-5	8.740E-4		177.78	99.76	28.43		8.98	5.74	7.23		3	2	2		5	3	3
Q62975	Protein Z-dependent protease inhibitor OS=Rattus norvegicus GN=Serpina10 PE=2 SV=2 - [ZPI_RAT]	50.2	9.791E-5	0.000E0	0.000E0	0.000E0	304.08				11.01				4				8			
Q62991	Sec1 family domain-containing protein 1 OS=Rattus norvegicus GN=Scfd1 PE=1 SV=1 - [SCFD1_RAT]	72.2	3.085E-5	0.000E0	2.049E-5	0.000E0	29.71		59.50		2.98		2.98		2		2		2		3	
Q63009	Protein arginine N-methyltransferase 1 OS=Rattus norvegicus GN=Prmt1 PE=1 SV=1 - [ANM1_RAT]	40.5	0.000E0	2.289E-4	2.698E-4	2.817E-4		591.89	647.16	547.78		25.78	38.81	48.73		10	12	14		20	20	21
Q63014	A-kinase anchor protein 8 OS=Rattus norvegicus OX=10116 GN=Akap8 PE=1 SV=1 - [AKAP8_RAT]	76.1	0.000E0	5.178E-5	0.000E0	0.000E0		92.76		20.29		4.51		1.31		2		1		3		1
Q63041	Alpha-1-macroglobulin OS=Rattus norvegicus GN=A1m PE=1 SV=1 - [A1M_RAT]	167.0	2.365E-3	2.464E-4	5.947E-4	2.213E-4	1569.39	586.47	1138.93	344.80	27.73	17.00	27.67	13.60	26	16	27	12	73	28	57	19
Q63060	Glycerol kinase OS=Rattus norvegicus OX=10116 GN=Gk PE=2 SV=1 - [GLPK_RAT]	57.4	0.000E0	5.483E-5	8.173E-5	4.444E-5		103.49	145.94	48.32		14.69	17.37	7.44		6	7	3		9	10	4
Q63068	Phosphorylated adapter RNA export protein OS=Rattus norvegicus OX=10116 GN=Phax PE=1 SV=2 - [PHAX_RAT]	43.1	0.000E0	2.348E-5	0.000E0	0.000E0		32.34				2.86				1				2		
Q63081	Protein disulfide-isomerase A6 OS=Rattus norvegicus GN=Pdia6 PE=1 SV=2 - [PDIA6_RAT]	48.1	1.610E-3	1.214E-3	1.039E-3	7.540E-4	1493.95	1494.56	1734.52	1136.08	32.27	36.14	40.91	40.23	11	12	14	13	36	42	46	34
Q63083	Nucleobindin-1 OS=Rattus norvegicus OX=10116 GN=Nucb1 PE=1 SV=1 - [NUCB1_RAT]	53.5	0.000E0	0.000E0	4.023E-5	4.808E-5			143.34	123.15			8.71	7.63			3	3			7	6
Q63120	Canalicular multispecific organic anion transporter 1 OS=Rattus norvegicus OX=10116 GN=Abcc2 PE=1 SV=1 - [MRP2_RA	173.3	1.052E-4	0.000E0	8.061E-5	4.374E-5	269.66		385.43	98.08	8.63		10.58	6.62	12		9	6	18		14	8
Q63135	Complement component receptor 1-like protein OS=Rattus norvegicus GN=Cr1l PE=1 SV=1 - [CR1L_RAT]	61.6	0.000E0	4.552E-5	4.488E-5	0.000E0		29.14	23.13			10.73	6.08			3	2			4	2	
Q63191	Apical endosomal glycoprotein OS=Rattus norvegicus OX=10116 GN=Mamdc4 PE=1 SV=1 - [AEGP_RAT]	133.7	1.566E-3	2.217E-4	2.286E-4	2.117E-4	2391.20	865.98	957.89	645.24	26.15	18.59	16.69	15.30	23	16	15	14	88	37	31	25
Q63228	Glia maturation factor beta OS=Rattus norvegicus GN=Gmfb PE=1 SV=2 - [GMFB_RAT]	16.7	0.000E0	2.066E-4	1.503E-4	1.021E-4		140.12	93.39	143.94		14.08	14.08	14.08		2	2	2		3	3	4
Q63270	Cytoplasmic aconitate hydratase OS=Rattus norvegicus GN=Aco1 PE=1 SV=1 - [ACOC_RAT]	98.1	1.122E-4	4.268E-4	5.268E-4	3.991E-4	89.43	869.06	1083.05	496.99	10.91	34.08	39.15	23.40	8	22	25	15	11	51	59	33
Q63279	Keratin, type I cytoskeletal 19 OS=Rattus norvegicus GN=Krt19 PE=1 SV=2 - [K1C19_RAT]	44.6	6.860E-3	4.490E-3	4.773E-3	2.602E-3	2352.07	2687.61	2525.40	1995.91	60.79	69.23	65.76	67.49	28	30	30	27	87	91	90	78
Q63347	26S protease regulatory subunit 7 OS=Rattus norvegicus GN=Psmc2 PE=1 SV=3 - [PRS7_RAT]	48.5	0.000E0	0.000E0	2.917E-5	3.374E-5			68.48	95.11			9.93	8.31			3	3			4	5
Q63357	Unconventional myosin-Id OS=Rattus norvegicus OX=10116 GN=Myo1d PE=1 SV=3 - [MYO1D_RAT]	116.0	0.000E0	0.000E0	0.000E0	1.378E-5				22.57				0.89				1				1
Q63362	NADH dehydrogenase [ubiquinone] 1 alpha subcomplex subunit 5 OS=Rattus norvegicus OX=10116 GN=Ndufa5 PE=1 SV	13.4	2.251E-5	0.000E0	0.000E0	0.000E0	52.85				17.24				1				2			
Q63396	Activated RNA polymerase II transcriptional coactivator p15 OS=Rattus norvegicus GN=Sub1 PE=2 SV=3 - [TCP4_RAT]	14.4	0.000E0	3.512E-5	3.116E-5	4.579E-5		36.47	32.69	42.74		7.09	7.09	7.09		1	1	1		2	2	2
Q63413	Spliceosome RNA helicase Ddx39b OS=Rattus norvegicus GN=Ddx39b PE=1 SV=3 - [DX39B_RAT]	49.0	2.750E-4	4.963E-4	7.792E-4	9.993E-4	607.73	979.18	1236.15	1318.44	39.02	45.79	48.36	48.36	14	16	18	18	24	46	52	55
Q63416	Inter-alpha-trypsin inhibitor heavy chain H3 OS=Rattus norvegicus GN=Itih3 PE=2 SV=1 - [ITIH3_RAT]	99.0	1.452E-4	7.029E-5	1.470E-4	1.030E-4	131.19	160.96	251.22	119.42	10.37	7.67	14.09	13.42	8	6	8	7	12	10	14	9
Q63493	Antigen-presenting glycoprotein CD1d OS=Rattus norvegicus OX=10116 GN=Cd1d PE=2 SV=2 - [CD1D_RAT]	38.5	1.424E-4	1.473E-4	9.872E-5	1.193E-4	175.50	185.93	170.95	95.97	12.50	16.67	12.50	16.67	4	5	4	5	9	9	8	11
Q63507	60S ribosomal protein L14 OS=Rattus norvegicus GN=Rpl14 PE=1 SV=3 - [RL14_RAT]	23.3	1.145E-4	7.066E-4	8.128E-4	5.008E-4	128.64	554.87	406.26	579.23	5.61	20.09	24.77	24.77	1	4	5	5	2	17	15	17
Q63524	Transmembrane emp24 domain-containing protein 2 OS=Rattus norvegicus OX=10116 GN=Tmed2 PE=1 SV=1 - [TMED2_	22.7	0.000E0	3.517E-5	2.180E-5	0.000E0		23.55	32.95			10.45	18.41			2	2			2	4	
Q63525	Nuclear migration protein nudC OS=Rattus norvegicus GN=Nudc PE=1 SV=1 - [NUDC_RAT]	38.4	2.571E-4	1.735E-4	1.620E-4	1.154E-4	241.38	439.75	284.79	250.29	13.25	33.73	27.71	31.63	4	9	7	8	7	16	10	11
Q63530	Phosphotriesterase-related protein OS=Rattus norvegicus GN=Pter PE=2 SV=2 - [PTER_RAT]	39.1	5.288E-5	5.413E-5	4.762E-5	3.449E-5	122.97	210.47	210.48	98.12	10.32	11.75	13.75	13.75	3	3	4	4	5	5	8	5
Q63556	Serine protease inhibitor A3M (Fragment) OS=Rattus norvegicus GN=Serpina3m PE=2 SV=1 - [SPA3M_RAT]	46.1	2.941E-3	3.217E-4	5.800E-4	4.559E-4	1077.54	136.46	150.81	29.12	27.43	6.55	6.55	3.64	9	3	3	1	35	5	5	2
Q63569	26S protease regulatory subunit 6A OS=Rattus norvegicus GN=Psmc3 PE=2 SV=1 - [PRS6A_RAT]	49.1	0.000E0	7.377E-5	2.449E-4	5.715E-5		191.69	352.09	232.36		15.03	29.61	12.07		5	10	4		8	18	6
Q63570	26S protease regulatory subunit 6B OS=Rattus norvegicus GN=Psmc4 PE=1 SV=1 - [PRS6B_RAT]	47.4	0.000E0	0.000E0	6.357E-5	5.054E-5			78.74	56.14			9.09	10.53			3	4			5	4
Q63584	Transmembrane emp24 domain-containing protein 10 OS=Rattus norvegicus OX=10116 GN=Tmed10 PE=1 SV=2 - [TMEI	24.8	9.729E-5	6.215E-5	1.070E-4	1.519E-4	183.70	181.57	181.45	25.53	27.85	27.85	27.85	10.50	4	4	4	2	7	8	7	3
Q63598	Plastin-3 OS=Rattus norvegicus GN=Pls3 PE=2 SV=2 - [PLST_RAT]	70.6	1.032E-3	8.155E-4	1.030E-3	6.560E-4	416.90	569.21	729.25	585.27	5.56	13.33	17.30	19.21	3	7	9	10	12	17	23	21
Q63610	Tropomyosin alpha-3 chain OS=Rattus norvegicus GN=Tpm3 PE=1 SV=2 - [TPM3_RAT]	29.0	1.619E-3	2.126E-3	2.956E-3	2.053E-3	1259.25	1081.75	1334.07	1124.47	40.32	48.79	54.44	45.16	15	19	21	17	42	42	53	44

Q63617	Hypoxia up-regulated protein 1 OS=Rattus norvegicus GN=Hyou1 PE=1 SV=1 - [HYOU1_RAT]	111.2	4.180E-4	9.333E-4	9.882E-4	2.289E-4	377.23	312.47	290.86	200.15	9.01	10.31	13.01	12.21	7	8	10	9	18	15	19	12
Q63623	Protein SCAF8 OS=Rattus norvegicus OX=10116 GN=Scaf8 PE=1 SV=1 - [SCAF8_RAT]	139.4	0.000E0	0.000E0	0.000E0	3.977E-5				26.10				0.95				1				1
Q63644	Rho-associated protein kinase 1 OS=Rattus norvegicus GN=Rock1 PE=1 SV=1 - [ROCK1_RAT]	159.5	0.000E0	0.000E0	8.642E-6	0.000E0	23.73	26.40	16.32		0.73	0.73	0.73		1	1	1		1	1	1	
Q63661	Mucin-4 OS=Rattus norvegicus GN=Muc4 PE=1 SV=2 - [MUC4_RAT]	247.9	9.196E-5	0.000E0	0.000E0	0.000E0	144.43				1.71				4				7			
Q63690	Apoptosis regulator BAX OS=Rattus norvegicus GN=Bax PE=1 SV=2 - [BAX_RAT]	21.3	0.000E0	0.000E0	0.000E0	2.053E-5				35.41				6.77				1				1
Q63692	Hsp90 co-chaperone Cdc37 OS=Rattus norvegicus GN=Cdc37 PE=1 SV=2 - [CDC37_RAT]	44.5	4.699E-5	1.477E-4	1.722E-4	1.399E-4	74.64	120.66	217.55	141.38	4.75	13.46	16.36	13.46	2	4	5	4	4	8	11	8
Q63716	Peroxiredoxin-1 OS=Rattus norvegicus GN=Prdx1 PE=1 SV=1 - [PRDX1_RAT]	22.1	1.123E-2	6.912E-3	6.274E-3	4.778E-3	2208.84	1949.58	1920.47	1546.29	75.88	75.88	79.40	79.40	17	16	17	17	96	87	87	80
Q63768	Adapter molecule crk OS=Rattus norvegicus GN=Crk PE=1 SV=1 - [CRK_RAT]	33.8	1.408E-4	9.768E-5	1.255E-4	6.962E-5	76.59	129.83	110.47	90.53	7.24	20.72	10.53	13.16	2	3	2	3	4	5	5	5
Q63797	Proteasome activator complex subunit 1 OS=Rattus norvegicus GN=Psme1 PE=2 SV=1 - [PSME1_RAT]	28.6	3.837E-4	3.720E-4	4.422E-4	4.605E-4	298.60	417.74	612.11	562.71	40.56	35.34	49.00	49.00	9	9	11	11	23	21	25	23
Q63798	Proteasome activator complex subunit 2 OS=Rattus norvegicus GN=Psme2 PE=2 SV=3 - [PSME2_RAT]	26.8	6.242E-5	0.000E0	1.070E-4	1.304E-4	47.42	56.42	358.17	270.70	16.81	5.88	44.54	24.37	4	1	10	6	6	1	16	9
Q63862	Myosin-11 (Fragments) OS=Rattus norvegicus GN=Myh11 PE=1 SV=3 - [MYH11_RAT]	152.4	6.549E-4	2.438E-3	2.176E-3	1.892E-3	348.03	4496.49	4610.38	5251.68	9.95	47.70	46.80	51.24	12	63	59	69	18	150	136	187
Q63945	Protein SET OS=Rattus norvegicus GN=Set PE=2 SV=2 - [SET_RAT]	33.4	0.000E0	1.175E-3	1.112E-3	1.019E-3		533.70	543.70	600.36		29.41	40.48	43.94		9	9	10		19	21	21
Q64057	Alpha-aminoadipic semialdehyde dehydrogenase OS=Rattus norvegicus GN=Aldh7a1 PE=1 SV=2 - [AL7A1_RAT]	58.7	0.000E0	1.868E-4	3.152E-4	1.539E-4		419.27	823.55	490.14		33.77	45.27	39.52		11	16	14		22	30	25
Q64060	Probable ATP-dependent RNA helicase DDX4 OS=Rattus norvegicus OX=10116 GN=Ddx4 PE=1 SV=1 - [DDX4_RAT]	77.9	0.000E0	0.000E0	1.802E-5	2.159E-5			25.89	26.78			1.68	1.68			1	1			2	1
Q64119	Myosin light polypeptide 6 OS=Rattus norvegicus GN=Myl6 PE=1 SV=3 - [MYL6_RAT]	17.0	3.875E-5	1.663E-4	1.714E-4	2.015E-4	40.06	178.34	188.68	189.47	15.89	42.38	15.89	35.76	2	5	2	5	3	11	5	10
Q64122	Myosin regulatory light polypeptide 9 OS=Rattus norvegicus OX=10116 GN=Myl9 PE=1 SV=2 - [MYL9_RAT]	19.7	0.000E0	4.640E-4	4.477E-4	5.185E-4		707.15	714.55	619.07		52.05	52.05	52.05		7	7	7		21	18	22
Q64194	Lysosomal acid lipase/cholesteryl ester hydrolase OS=Rattus norvegicus GN=Lipa PE=2 SV=1 - [LICH_RAT]	45.2	5.858E-5	8.540E-5	7.632E-5	9.496E-5	32.52	134.62	130.98	132.21	3.27	5.79	10.83	5.79	1	2	3	2	2	7	8	4
Q641W2	UPF0160 protein MYG1, mitochondrial OS=Rattus norvegicus GN=Myg1 PE=1 SV=1 - [MYG1_RAT]	42.9	2.395E-5	1.183E-4	1.158E-4	7.941E-5	27.04	150.01	137.47	77.37	2.10	18.11	12.60	15.49	1	5	4	4	1	10	7	6
Q641X3	Beta-hexosaminidase subunit alpha OS=Rattus norvegicus GN=Hexa PE=2 SV=1 - [HEXA_RAT]	60.5	1.666E-4	1.275E-4	2.178E-4	3.181E-4	34.43	107.17	147.41	280.82	3.60	11.36	17.05	18.56	2	4	6	7	3	7	7	13
Q641X8	Eukaryotic translation initiation factor 3 subunit E OS=Rattus norvegicus GN=Eif3e PE=2 SV=1 - [EIF3E_RAT]	52.2	0.000E0	3.334E-5	1.060E-4	7.637E-5		55.27	330.13	91.93		8.54	19.78	15.06		4	7	6		7	12	9
Q641Y0	Dolichyl-diphosphooligosaccharide--protein glycosyltransferase 48 kDa subunit OS=Rattus norvegicus OX=10116 GN=Ddo	48.9	0.000E0	5.551E-5	0.000E0	0.000E0		40.05				4.54				2				2		
Q641Y8	ATP-dependent RNA helicase DDX1 OS=Rattus norvegicus GN=Ddx1 PE=2 SV=1 - [DDX1_RAT]	82.4	2.168E-5	2.334E-4	1.592E-4	1.623E-4	35.95	570.14	530.35	425.13	5.00	19.73	19.86	20.54	3	12	12	12	3	26	22	22
Q641Z4	Cyclin-dependent kinase 9 OS=Rattus norvegicus GN=Cdk9 PE=2 SV=1 - [CDK9_RAT]	42.7	3.682E-5	8.873E-5	6.450E-5	1.322E-4	24.96	50.38	40.24	51.61	4.30	4.30	4.30	4.30	2	2	2	2	3	4	3	5
Q641Z6	EH domain-containing protein 1 OS=Rattus norvegicus GN=Ehd1 PE=1 SV=1 - [EHD1_RAT]	60.6	0.000E0	0.000E0	6.254E-5	4.695E-5			59.62	44.65			4.49	7.68			2	3			3	5
Q64230	Meprin A subunit alpha OS=Rattus norvegicus OX=10116 GN=Mep1a PE=1 SV=2 - [MEP1A_RAT]	85.1	1.285E-3	7.804E-4	7.693E-4	6.928E-4	1467.33	1218.93	1189.27	944.03	35.96	29.68	31.28	25.53	21	17	19	15	57	48	51	43
Q64240	Protein AMBP OS=Rattus norvegicus GN=Ambp PE=1 SV=1 - [AMBP_RAT]	38.8	4.449E-5	4.606E-5	5.756E-5	0.000E0	34.90	47.35	84.20		6.30	6.59	6.30		2	2	2		3	3	5	
Q64268	Heparin cofactor 2 OS=Rattus norvegicus GN=Serpind1 PE=1 SV=1 - [HEP2_RAT]	54.5	4.321E-5	0.000E0	0.000E0	0.000E0	37.11				4.18				2				3			
Q642A7	Protein FAM151A OS=Rattus norvegicus OX=10116 GN=Fam151a PE=2 SV=1 - [F151A_RAT]	67.1	6.227E-5	0.000E0	2.505E-5	0.000E0	86.77		41.63		7.07		1.64		3		1		4		2	
Q642C0	DnaJ homolog subfamily C member 8 OS=Rattus norvegicus GN=Dnajc8 PE=2 SV=1 - [DNJC8_RAT]	29.8	2.304E-4	2.574E-4	1.773E-4	2.204E-4	106.63	172.15	88.02	74.91	19.76	27.27	16.60	19.76	4	6	3	4	7	12	6	6
Q64350	Translation initiation factor eIF-2B subunit epsilon OS=Rattus norvegicus GN=Eif2b5 PE=1 SV=2 - [EI2BE_RAT]	80.2	0.000E0	4.221E-5	5.261E-5	0.000E0		58.80	36.82			6.28	2.51			3	2			4	2	
Q64361	Latexin OS=Rattus norvegicus GN=Lxn PE=1 SV=1 - [LXN_RAT]	25.6	1.945E-4	1.937E-4	2.304E-4	1.515E-4	145.75	236.09	160.96	79.79	14.35	14.35	12.11	8.07	2	2	2	1	5	5	5	2
Q64428	Trifunctional enzyme subunit alpha, mitochondrial OS=Rattus norvegicus GN=Hadha PE=1 SV=2 - [ECHA_RAT]	82.6	0.000E0	2.507E-5	6.784E-5	3.725E-5		53.11	95.38	85.23		3.01	18.87	9.57		2	8	6		3	12	8
Q64537	Calpain small subunit 1 OS=Rattus norvegicus GN=Capns1 PE=1 SV=3 - [CPNS1_RAT]	28.6	3.043E-4	1.615E-4	2.060E-4	1.102E-4	319.76	224.90	212.78	188.90	20.74	13.70	22.22	13.70	5	4	5	4	9	7	9	7
Q64550	UDP-glucuronosyltransferase 1-1 OS=Rattus norvegicus OX=10116 GN=Ugt1a1 PE=1 SV=1 - [UD11_RAT]	59.6	7.210E-5	0.000E0	8.101E-5	4.471E-5	79.52		35.53	34.83	5.42		9.72	2.06	3		3	1	4		4	1
Q64559	Cytosolic acyl coenzyme A thioester hydrolase OS=Rattus norvegicus GN=Acot7 PE=1 SV=4 - [BACH_RAT]	42.7	0.000E0	0.000E0	0.000E0	1.955E-5				28.76				2.89				1				2
Q64560	Tripeptidyl-peptidase 2 OS=Rattus norvegicus GN=Tpp2 PE=2 SV=3 - [TPP2_RAT]	138.2	0.000E0	3.412E-5	5.861E-5	3.809E-5		56.70	162.44	73.58		2.56	8.33	7.05		2	7	6		3	9	8
Q64591	2,4-dienoyl-CoA reductase, mitochondrial OS=Rattus norvegicus GN=Decr1 PE=1 SV=2 - [DECR_RAT]	36.1	0.000E0	0.000E0	1.518E-4	8.060E-5			25.90	127.38			6.57	13.13			1	3			1	5
Q64620	Serine/threonine-protein phosphatase 6 catalytic subunit OS=Rattus norvegicus GN=Ppp6c PE=2 SV=2 - [PPP6_RAT]	35.1	0.000E0	5.797E-5	6.418E-5	5.954E-5		80.49	154.75	73.73		8.52	24.92	18.69		2	5	4		3	8	5
Q64632	Integrin beta-4 OS=Rattus norvegicus GN=Itgb4 PE=1 SV=1 - [ITB4_RAT]	200.5	3.161E-5	5.544E-5	4.482E-5	0.000E0	37.77	64.90	35.99		2.99	3.54	3.54		5	5	5		6	7	6	
Q64640	Adenosine kinase OS=Rattus norvegicus GN=Adk PE=1 SV=3 - [ADK_RAT]	40.1	0.000E0	2.884E-4	3.437E-4	3.537E-4		373.48	497.96	477.88		27.15	37.12	32.69		7	11	10		16	18	20
Q64716	Insulin receptor-related protein OS=Rattus norvegicus OX=10116 GN=Insrr PE=1 SV=3 - [INSRR_RAT]	144.8	2.733E-4	2.726E-4	0.000E0	0.000E0	21.59	23.35			0.62	0.54			2	1			4	2		
Q66H12	Alpha-N-acetylgalactosaminidase OS=Rattus norvegicus GN=Naga PE=2 SV=1 - [NAGAB_RAT]	46.8	3.264E-3	9.520E-4	1.041E-3	1.196E-3	2810.71	1625.78	1920.38	1617.61	60.72	50.36	57.83	62.65	25	19	21	22	127	67	72	79
Q66H59	N-acetylneuraminate lyase OS=Rattus norvegicus OX=10116 GN=Npl PE=2 SV=1 - [NPL_RAT]	35.1	1.218E-3	1.750E-4	6.641E-4	5.423E-4	1413.94	528.13	1185.76	762.06	79.06	35.63	57.81	51.88	21	8	15	13	55	17	44	32
Q66H61	Glutamine--tRNA ligase OS=Rattus norvegicus OX=10116 GN=Qars PE=1 SV=1 - [SYQ_RAT]	87.6	0.000E0	9.764E-5	1.467E-4	1.008E-4		506.13	462.87	358.55		27.61	23.61	24.52		16	12	12		25	21	17
Q66H68	RNA-binding protein 47 OS=Rattus norvegicus GN=Rbm47 PE=2 SV=1 - [RBM47_RAT]	64.1	0.000E0	1.368E-4	2.365E-4	1.022E-4		83.39	166.00	149.64		9.32	15.25	13.39		5	7	6		10	13	10
Q66H71	Serine/threonine-protein phosphatase CPPED1 OS=Rattus norvegicus GN=Cpped1 PE=1 SV=1 - [CPPED_RAT]	35.2	2.895E-4	1.665E-3	6.449E-4	1.673E-4	377.63	276.79	295.83	114.33	26.60	24.36	24.36	8.65	7	6	6	2	12	11	11	4
Q66H76	Paxillin OS=Rattus norvegicus OX=10116 GN=Pxn PE=1 SV=1 - [PAXI_RAT]	64.0	0.000E0	3.812E-5	0.000E0	0.000E0		22.86				6.14				2				3		
Q66H80	Coatomer subunit delta OS=Rattus norvegicus GN=Arcn1 PE=2 SV=1 - [COPD_RAT]	57.2	0.000E0	2.723E-4	2.260E-4	1.265E-4		524.99	455.27	303.41		17.42	18.98	16.63		8	10	8		17	16	13
Q66H94	Peptidyl-prolyl cis-trans isomerase FKBP9 OS=Rattus norvegicus GN=Fkbp9 PE=2 SV=1 - [FKBP9_RAT]	63.1	1.428E-4	6.272E-5	7.141E-5	8.334E-5	152.60	55.89	61.31	52.34	9.47	4.74	4.91	1.58	6	3	3	1	12	4	3	2
Q66HA6	ADP-ribosylation factor-like protein 8B OS=Rattus norvegicus GN=Arl8b PE=2 SV=1 - [ARL8B_RAT]	21.5	1.016E-4	2.962E-5	4.392E-5	0.000E0	139.97	25.13	115.83		27.42	22.04	27.42		3	2	3		7	2	6	
Q66HA8	Heat shock protein 105 kDa OS=Rattus norvegicus GN=Hsph1 PE=2 SV=1 - [HS105_RAT]	96.4	3.574E-5	3.268E-4	2.281E-4	7.509E-5	26.72	439.84	295.92	200.10	0.93	20.05	14.45	12.59	1	12	8	8	1	21	14	11
Q66HB6	Cancer-associated gene 1 protein homolog OS=Rattus norvegicus GN=Cage1 PE=2 SV=1 - [CAGE1_RAT]	97.4	7.515E-5	0.000E0	6.647E-5	0.000E0	0.00		0.00		1.89		0.83		2		1		3		1	
Q66HD0	Endoplasmin OS=Rattus norvegicus GN=Hsp90b1 PE=1 SV=2 - [ENPL_RAT]	92.7	1.199E-3	1.812E-3	2.484E-3	1.910E-3	511.63	1138.48	2109.64	1893.29	10.32	24.38	44.53	45.27	8	19	34	36	15	46	87	81
Q66HD3	Nuclear autoantigenic sperm protein OS=Rattus norvegicus GN=Nasp PE=1 SV=1 - [NASP_RAT]	84.1	0.000E0	0.000E0	9.209E-5	3.802E-5			87.52	39.03			5.80	4.51			3	2			5	4
Q66HF1	NADH-ubiquinone oxidoreductase 75 kDa subunit, mitochondrial OS=Rattus norvegicus GN=Ndufs1 PE=1 SV=1 - [NDUS1	79.4	0.000E0	4.070E-5	3.258E-5	2.823E-5		80.57	66.93	52.62		5.64	3.16	4.54		4	2	3		5	2	3
Q66HF8	Aldehyde dehydrogenase X, mitochondrial OS=Rattus norvegicus GN=Aldh1b1 PE=1 SV=1 - [AL1B1_RAT]	57.6	2.625E-4	3.042E-4	4.184E-4	2.299E-4	55.59	463.48	553.83	315.70	1.54	32.76	38.73	27.36	1	12	14	11	2	28	29	21
Q66HG3	Beta-Ala-His dipeptidase OS=Rattus norvegicus OX=10116 GN=Cndp1 PE=1 SV=1 - [CNDP1_RAT]	54.9	5.834E-5	1.116E-4	1.289E-4	7.450E-5	87.15	97.45	211.15	68.85	5.69	14.84	27.03	12.60	3	5	8	4	5	7	11	5
Q66HG4	Aldose 1-epimerase OS=Rattus norvegicus OX=10116 GN=Galm PE=1 SV=1 - [GALM_RAT]	37.9	8.493E-4	2.316E-4	3.040E-4	2.926E-4	634.51	306.42	452.04	350.73	37.13	26.02	28.07	28.07	8	6	6	6	29	12	17	15
Q66HG5	Transmembrane 9 superfamily member 2 OS=Rattus norvegicus OX=10116 GN=Tm9sf2 PE=2 SV=1 - [TM9S2_RAT]	75.5	4.819E-5	5.062E-5	7.222E-5	0.000E0	27.52	92.54	45.83		4.07	7.24	2.26		2	3	1		2	4	2	
Q66HR2	Microtubule-associated protein RP/EB family member 1 OS=Rattus norvegicus GN=Mapre1 PE=1 SV=3 - [MARE1_RAT]	30.0	1.083E-4	1.269E-4	1.114E-4	1.331E-4	54.04	88.55	96.17	42.86	6.72	26.87	17.54	6.72	2	5	4	2	4	7	6	3
Q66HS7	PDZ and LIM domain protein 3 OS=Rattus norvegicus OX=10116 GN=Pdlim3 PE=1 SV=2 - [PDLI3_RAT]	39.1	0.000E0	4.258E-5	0.000E0	0.000E0		49.35				4.14				1				1		
Q66X93	Staphylococcal nuclease domain-containing protein 1 OS=Rattus norvegicus GN=Snd1 PE=2 SV=1 - [SND1_RAT]	101.9	0.000E0	1.989E-4	2.651E-4	1.829E-4		529.81	607.26	432.96		41.36	37.51	33.22		23	23	20		38	40	35
Q675A5	Group XV phospholipase A2 OS=Rattus norvegicus OX=10116 GN=Pla2g15 PE=1 SV=1 - [PAG15_RAT]	47.4	3.874E-4	0.000E0	6.488E-5	8.131E-5	252.43		77.60	33.31	23.97		7.75	6.30	7		2	2	12		3	2
Q68A21	Transcriptional activator protein Pur-beta OS=Rattus norvegicus GN=Purb PE=1 SV=3 - [PURB_RAT]	33.4	7.698E-5	0.000E0	6.361E-5	3.390E-5	21.69		56.51	54.53	1.90		20.00	6.98	1		5	3	2		8	9
Q68FP1	Gelsolin OS=Rattus norvegicus GN=Gsn PE=1 SV=1 - [GELS_RAT]	86.0	5.597E-4	6.680E-4	5.452E-4	4.350E-4	1040.33	1321.12	1391.92	921.69	28.46	31.15	40.77	28.08	16	17	18	15	34	45	47	35
Q68FQ0	T-complex protein 1 subunit epsilon OS=Rattus norvegicus GN=Cct5 PE=1 SV=1 - [TCPE_RAT]	59.5	4.080E-5	1.093E-4	3.010E-4	1.843E-4	147.56	266.08	832.79	304.25	4.25	11.09	40.11	17.38	3	6	17	8	5	9	38	14
Q68FR6	Elongation factor 1-gamma OS=Rattus norvegicus GN=Eef1g PE=1 SV=3 - [EF1G_RAT]	50.0	1.516E-3	9.880E-4	7.516E-4	7.725E-4	744.23	893.24	872.76	938.87	36.84	28.15	41.19	36.38	13	11	16	14	35	36	43	46
Q68FR9	Elongation factor 1-delta OS=Rattus norvegicus GN=Eef1d PE=1 SV=2 - [EF1D_RAT]	31.3	0.000E0	1.296E-4	1.414E-4	1.030E-4		133.11	138.94	144.68		29.18	20.64	25.98		5	4	4		7	5	5
Q68FS2	COP9 signalosome complex subunit 4 OS=Rattus norvegicus GN=Cops4 PE=1 SV=1 - [CSN4_RAT]	46.3	0.000E0	1.581E-4	1.587E-4	6.016E-5		222.02	284.53	157.36		23.89	29.80	14.04		8	8	5		12	14	9
Q68FS3	F-box only protein 7 OS=Rattus norvegicus GN=Fbxo7 PE=2 SV=1 - [FBX7_RAT]	57.5	0.000E0	1.540E-5	0.000E0	0.000E0		29.68	45.53			3.45	3.45			1	1			1	1	
Q68FS4	Cytosol aminopeptidase OS=Rattus norvegicus GN=Lap3 PE=1 SV=1 - [AMPL_RAT]	56.1	1.236E-3	5.699E-4	6.619E-4	2.875E-4	801.32	1015.88	1247.93	851.25	52.22	52.41	54.72	49.13	20	20	23	18	35	41	48	34
Q68FT1	Ubiquinone biosynthesis protein COQ9, mitochondrial OS=Rattus norvegicus OX=10116 GN=Coq9 PE=1 SV=2 - [COQ9_R	35.1	9.588E-5	2.779E-5	0.000E0	0.000E0	85.72	32.34			6.41	9.62			2	3			3	3		
Q68FT3	Pyridine nucleotide-disulfide oxidoreductase domain-containing protein 2 OS=Rattus norvegicus OX=10116 GN=Pyroxd2 P	62.8	3.140E-5	0.000E0	0.000E0	0.000E0	36.94				2.24				1				1			
Q68FU3	Electron transfer flavoprotein subunit beta OS=Rattus norvegicus GN=Etfb PE=2 SV=3 - [ETFB_RAT]	27.7	0.000E0	5.417E-4	6.950E-4	4.819E-4		1037.28	1329.90	990.38		49.02	49.02	45.49		11	11	10		27	35	27
Q68FV6	Glycosylated lysosomal membrane protein OS=Rattus norvegicus OX=10116 GN=Glmp PE=2 SV=1 - [GLMP_RAT]	43.8	1.698E-4	4.976E-5	0.000E0	0.000E0	114.64	65.41			4.95	4.95			1	1			2	1		
Q68FW9	COP9 signalosome complex subunit 3 OS=Rattus norvegicus GN=Cops3 PE=2 SV=1 - [CSN3_RAT]	47.8	0.000E0	6.015E-5	6.976E-5	0.000E0		33.66	81.36			7.33	7.33			2	2			3	3	
Q68FX0	Isocitrate dehydrogenase [NAD] subunit beta, mitochondrial OS=Rattus norvegicus GN=Idh3B PE=1 SV=1 - [IDH3B_RAT]	42.3	0.000E0	0.000E0	4.511E-5	4.359E-5			49.20	60.02			4.16	4.16			1	1			2	2
Q68FY0	Cytochrome b-c1 complex subunit 1, mitochondrial OS=Rattus norvegicus OX=10116 GN=Uqcrc1 PE=1 SV=1 - [QCR1_RA	52.8	3.843E-5	7.138E-5	7.887E-5	1.230E-4	43.64	79.17	90.34	211.00	3.96	10.83	12.71	21.04	1	3	4	7	2	5	5	10
Q68G31	Phenazine biosynthesis-like domain-containing protein OS=Rattus norvegicus OX=10116 GN=Pbld PE=2 SV=1 - [PBLD_RA	31.7	0.000E0	4.731E-5	0.000E0	0.000E0		28.42				3.82				1				2		
Q6AXQ0	SUMO-activating enzyme subunit 1 OS=Rattus norvegicus GN=Sae1 PE=2 SV=1 - [SAE1_RAT]	38.5	0.000E0	0.000E0	0.000E0	2.715E-5				30.21				6.59				1				1
Q6AXR4	Beta-hexosaminidase subunit beta OS=Rattus norvegicus GN=Hexb PE=2 SV=1 - [HEXB_RAT]	61.5	3.040E-4	3.151E-4	5.185E-4	6.811E-4	230.70	367.59	829.62	624.49	14.90	25.33	32.96	36.13	9	12	15	15	16	24	27	28
Q6AXS3	Protein DEK OS=Rattus norvegicus OX=10116 GN=Dek PE=1 SV=1 - [DEK_RAT]	42.9	0.000E0	3.700E-5	0.000E0	1.282E-4		38.81		77.43		5.82		5.82		2		2		2		3
Q6AXT8	Splicing factor 3A subunit 2 OS=Rattus norvegicus GN=Sf3a2 PE=2 SV=1 - [SF3A2_RAT]	49.9	1.753E-4	1.502E-4	1.309E-4	0.000E0	22.55	30.44	22.79		3.82	3.82	3.82		1	1	1		2	2	2	
Q6AY09	Heterogeneous nuclear ribonucleoprotein H2 OS=Rattus norvegicus GN=Hnrnph2 PE=1 SV=1 - [HNRH2_RAT]	49.3	1.233E-3	1.907E-3	1.676E-3	1.632E-3	803.95	1093.60	1027.50	962.47	24.72	24.72	30.51	22.94	10	10	11	9	28	34	34	27
Q6AY19	Atypical kinase COQ8B, mitochondrial OS=Rattus norvegicus OX=10116 GN=Coq8b PE=1 SV=1 - [COQ8B_RAT]	58.9	0.000E0	1.973E-5	0.000E0	0.000E0		29.26				1.89				1				1		
Q6AY20	Cation-dependent mannose-6-phosphate receptor OS=Rattus norvegicus OX=10116 GN=M6pr PE=1 SV=1 - [MPRD_RAT]	31.1	1.756E-4	9.811E-5	9.894E-5	0.000E0	82.78	62.36	54.08		5.04	5.04	5.04		1	1	1		2	2	2	
Q6AY80	Ribosyldihydronicotinamide dehydrogenase [quinone] OS=Rattus norvegicus OX=10116 GN=Nqo2 PE=1 SV=3 - [NQO2_R	26.3	0.000E0	4.704E-5	0.000E0	2.209E-5		136.20		37.42		9.96		17.75		2		3		4		3
Q6AY86	Vacuolar protein sorting-associated protein 26A OS=Rattus norvegicus GN=Vps26a PE=1 SV=1 - [VP26A_RAT]	38.1	8.261E-5	8.171E-5	8.971E-5	6.141E-5	94.45	166.30	213.27	55.78	17.43	21.41	27.83	19.88	4	5	7	5	4	8	11	7
Q6AYA1	H/ACA ribonucleoprotein complex subunit 1 OS=Rattus norvegicus OX=10116 GN=Gar1 PE=2 SV=1 - [GAR1_RAT]	23.0	0.000E0	0.000E0	0.000E0	6.767E-5				59.49				20.80				3				5
Q6AYC4	Macrophage-capping protein OS=Rattus norvegicus GN=Capg PE=1 SV=1 - [CAPG_RAT]	38.8	2.933E-4	3.299E-4	3.209E-4	2.376E-4	389.23	485.94	470.25	311.88	47.28	48.14	37.54	45.27	9	9	7	8	17	18	16	12
Q6AYD3	Proliferation-associated protein 2G4 OS=Rattus norvegicus GN=Pa2g4 PE=1 SV=1 - [PA2G4_RAT]	43.6	9.203E-5	5.017E-4	6.752E-4	5.141E-4	160.08	1030.94	977.66	723.60	19.04	55.33	54.82	55.33	5	17	17	17	8	36	41	30
Q6AYD9	Nucleoside diphosphate-linked moiety X motif 19 OS=Rattus norvegicus OX=10116 GN=Nudt19 PE=2 SV=1 - [NUD19_RA	39.9	0.000E0	3.374E-5	0.000E0	0.000E0		24.93				3.92				1				1		
Q6AYH5	Dynactin subunit 2 OS=Rattus norvegicus GN=Dctn2 PE=1 SV=1 - [DCTN2_RAT]	44.1	2.357E-5	1.883E-5	4.354E-5	1.941E-5	21.38	39.35	63.73	42.97	3.98	7.71	13.68	7.71	1	2	4	2	1	2	5	2
Q6AYK1	RNA-binding protein with serine-rich domain 1 OS=Rattus norvegicus OX=10116 GN=Rnps1 PE=2 SV=1 - [RNPS1_RAT]	34.2	0.000E0	5.193E-5	7.808E-5	9.851E-5		79.77	189.51	128.64		4.92	11.48	11.48		1	3	3		2	6	6
Q6AYK3	Inositol-3-phosphate synthase 1 OS=Rattus norvegicus GN=Isyna1 PE=3 SV=2 - [INO1_RAT]	60.8	1.065E-4	5.859E-5	0.000E0	6.244E-5	343.51	129.87		184.69	13.82	9.52		8.62	7	5		5	14	7		8
Q6AYK6	Calcyclin-binding protein OS=Rattus norvegicus GN=Cacybp PE=1 SV=1 - [CYBP_RAT]	26.5	1.956E-4	1.173E-4	1.051E-4	8.827E-5	234.20	183.67	152.06	95.49	29.26	21.83	11.79	11.79	6	4	3	3	11	7	6	4
Q6AYK8	Eukaryotic translation initiation factor 3 subunit D OS=Rattus norvegicus GN=Eif3d PE=1 SV=1 - [EIF3D_RAT]	63.9	0.000E0	2.462E-4	2.334E-4	1.717E-4		395.25	302.17	201.72		31.20	27.37	21.35		9	9	7		16	14	10
Q6AYL5	Splicing factor 3B subunit 4 OS=Rattus norvegicus GN=Sf3b4 PE=2 SV=1 - [SF3B4_RAT]	44.3	6.546E-5	1.285E-4	9.387E-5	1.029E-4	68.54	216.52	151.46	79.95	9.67	12.97	17.45	8.73	2	3	4	2	3	8	5	2
Q6AYQ2	Aldo-keto reductase family 1 member C21 OS=Rattus norvegicus GN=Akr1c21 PE=2 SV=1 - [AK1CL_RAT]	36.1	8.732E-5	2.163E-4	3.417E-4	2.251E-4	147.04	138.12	97.77	141.98	8.18	8.18	7.86	8.18	4	3	3	3	7	8	6	8
Q6AYQ8	Acylpyruvase FAHD1, mitochondrial OS=Rattus norvegicus OX=10116 GN=Fahd1 PE=1 SV=1 - [FAHD1_RAT]	24.5	0.000E0	8.112E-5	7.313E-5	9.365E-5		172.26	115.89	200.36		19.91	19.46	30.32		3	4	6		8	8	11
Q6AYR5	Clarin-3 OS=Rattus norvegicus OX=10116 GN=Clrn3 PE=2 SV=1 - [CLRN3_RAT]	25.3	8.871E-5	0.000E0	0.000E0	0.000E0	69.23				5.31				1				2			
Q6AYR8	Secernin-2 OS=Rattus norvegicus GN=Scrn2 PE=2 SV=1 - [SCRN2_RAT]	46.5	0.000E0	4.560E-5	3.651E-5	0.000E0		30.39	77.59			8.51	12.77			2	3			3	4	
Q6AYS4	Plasma alpha-L-fucosidase OS=Rattus norvegicus OX=10116 GN=Fuca2 PE=2 SV=1 - [FUCO2_RAT]	53.2	3.034E-5	5.590E-5	6.982E-5	7.715E-5	36.16	75.97	41.84	44.32	6.97	15.47	15.69	10.24	3	6	6	4	5	8	9	6
Q6AYS7	Aminoacylase-1A OS=Rattus norvegicus GN=Acy1a PE=1 SV=1 - [ACY1A_RAT]	45.8	6.941E-4	2.403E-4	2.185E-4	1.447E-4	617.54	374.08	329.66	204.23	48.04	22.79	31.13	14.22	16	9	11	6	35	16	18	10
Q6AYT0	Quinone oxidoreductase OS=Rattus norvegicus GN=Cryz PE=2 SV=1 - [QOR_RAT]	35.0	7.623E-5	0.000E0	7.548E-5	0.000E0	71.40		34.81		24.92		8.21		4		1		4		1	
Q6AYT3	tRNA-splicing ligase RtcB homolog OS=Rattus norvegicus GN=Rtcb PE=2 SV=1 - [RTCB_RAT]	55.2	0.000E0	1.717E-4	2.332E-4	1.317E-4		398.52	396.51	309.02		28.71	30.30	22.97		11	11	9		17	17	15
Q6AYZ1	Tubulin alpha-1C chain OS=Rattus norvegicus GN=Tuba1c PE=1 SV=1 - [TBA1C_RAT]	49.9	5.754E-4	6.555E-4	1.238E-3	1.839E-3	714.79	848.05	1538.08	1721.08	28.95	33.18	42.98	54.34	11	11	16	18	25	30	55	63
Q6AZ50	Ubiquitin-like-conjugating enzyme ATG3 OS=Rattus norvegicus GN=Atg3 PE=2 SV=1 - [ATG3_RAT]	35.8	0.000E0	0.000E0	0.000E0	2.873E-5		31.40		22.14		3.18		3.18		1		1		1		1
Q6AZ61	Probable lysosomal cobalamin transporter OS=Rattus norvegicus OX=10116 GN=Lmbrd1 PE=1 SV=1 - [LMBD1_RAT]	61.0	2.404E-4	0.000E0	8.497E-5	0.000E0	111.53		58.66		3.35		3.35		1		1		2		2	
Q6BBI8	Ubiquitin-fold modifier-conjugating enzyme 1 OS=Rattus norvegicus GN=Ufc1 PE=2 SV=1 - [UFC1_RAT]	19.5	0.000E0	4.062E-5	8.322E-5	0.000E0		21.04	20.71			4.79	10.18			1	2			1	2	
Q6DGG0	Peptidyl-prolyl cis-trans isomerase D OS=Rattus norvegicus GN=Ppid PE=1 SV=3 - [PPID_RAT]	40.7	6.458E-5	4.434E-4	4.554E-4	3.631E-4	131.82	838.81	740.13	599.23	14.59	54.86	54.59	37.03	5	16	16	12	6	33	30	24
Q6DGG1	Alpha/beta hydrolase domain-containing protein 14B OS=Rattus norvegicus GN=Abhd14b PE=2 SV=1 - [ABHEB_RAT]	22.6	6.812E-4	2.337E-4	2.246E-4	2.531E-4	350.47	195.75	151.08	194.41	35.24	31.43	24.76	31.43	7	5	4	5	19	10	8	10
Q6EV70	GDP-fucose protein O-fucosyltransferase 1 OS=Rattus norvegicus GN=Pofut1 PE=2 SV=1 - [OFUT1_RAT]	45.0	1.905E-4	5.731E-5	8.926E-5	0.000E0	129.51	45.21	72.06		32.91	9.11	9.11		9	2	2		13	3	3	
Q6GQP4	Ras-related protein Rab-31 OS=Rattus norvegicus GN=Rab31 PE=1 SV=2 - [RAB31_RAT]	21.4	7.067E-5	0.000E0	0.000E0	0.000E0	26.37				5.67				1				1			
Q6I7R3	Isochorismatase domain-containing protein 1 OS=Rattus norvegicus GN=Isoc1 PE=2 SV=1 - [ISOC1_RAT]	32.0	0.000E0	1.135E-4	8.358E-5	1.284E-4		64.65	122.71	94.11		5.05	21.21	8.75		1	4	2		1	5	3
Q6IFU7	Keratin, type I cytoskeletal 42 OS=Rattus norvegicus GN=Krt42 PE=3 SV=1 - [K1C42_RAT]	50.2	4.712E-3	1.713E-3	1.762E-3	1.615E-3	985.44	551.23	494.49	444.54	25.66	16.37	16.59	12.83	14	9	10	7	42	18	21	16
Q6IFU8	Keratin, type I cytoskeletal 17 OS=Rattus norvegicus GN=Krt17 PE=2 SV=1 - [K1C17_RAT]	48.1	6.860E-3	2.087E-3	2.317E-3	2.388E-3	1160.48	816.09	692.26	601.78	29.10	23.09	21.48	19.40	17	14	13	10	46	29	28	24
Q6IFV1	Keratin, type I cytoskeletal 14 OS=Rattus norvegicus GN=Krt14 PE=2 SV=1 - [K1C14_RAT]	52.7	6.689E-3	1.783E-3	1.958E-3	1.946E-3	1032.45	555.40	633.13	614.38	31.75	13.20	11.55	13.61	17	9	7	8	47	17	18	21
Q6IFW6	Keratin, type I cytoskeletal 10 OS=Rattus norvegicus GN=Krt10 PE=3 SV=1 - [K1C10_RAT]	56.5	1.003E-2	1.207E-3	1.690E-3	1.965E-3	4060.87	1447.86	1680.84	1861.45	25.48	19.58	19.58	20.91	18	14	13	15	115	48	56	63
Q6IFX1	Keratin, type I cytoskeletal 24 OS=Rattus norvegicus GN=Krt24 PE=3 SV=1 - [K1C24_RAT]	52.3	2.779E-3	1.526E-3	1.817E-3	1.936E-3	226.44	69.71	67.67	83.29	6.31	1.43	1.43	2.85	4	1	1	2	11	2	3	4
Q6IG02	Keratin, type II cytoskeletal 2 epidermal OS=Rattus norvegicus GN=Krt2 PE=3 SV=1 - [K22E_RAT]	69.1	4.177E-3	2.811E-4	6.024E-4	6.329E-4	966.45	369.84	453.53	469.10	9.34	7.59	7.59	8.91	12	7	6	9	35	11	14	16
Q6IG03	Keratin, type II cytoskeletal 73 OS=Rattus norvegicus GN=Krt73 PE=1 SV=1 - [K2C73_RAT]	60.3	1.160E-2	2.719E-3	3.963E-3	5.167E-3	540.67	403.03	445.74	490.78	8.86	7.59	9.40	11.03	6	5	6	7	20	17	18	20
Q6IG04	Keratin, type II cytoskeletal 72 OS=Rattus norvegicus GN=Krt72 PE=3 SV=2 - [K2C72_RAT]	56.8	1.907E-3	7.402E-4	7.501E-4	7.538E-4	156.68	99.29	102.02	103.77	5.38	3.08	5.19	5.38	3	2	3	3	10	6	6	7
Q6IG05	Keratin, type II cytoskeletal 75 OS=Rattus norvegicus GN=Krt75 PE=3 SV=2 - [K2C75_RAT]	59.0	7.711E-3	1.974E-3	2.729E-3	3.624E-3	1146.21	692.47	607.57	788.51	15.68	14.02	11.81	13.47	13	10	7	10	45	29	23	31
Q6IG12	Keratin, type II cytoskeletal 7 OS=Rattus norvegicus GN=Krt7 PE=3 SV=1 - [K2C7_RAT]	50.7	3.649E-3	1.189E-3	1.764E-3	1.941E-3	252.22	329.92	362.30	319.12	7.22	7.44	9.63	9.63	4	5	6	6	14	15	17	16
Q6IMF3	Keratin, type II cytoskeletal 1 OS=Rattus norvegicus GN=Krt1 PE=2 SV=1 - [K2C1_RAT]	64.8	1.720E-2	3.234E-3	4.467E-3	6.203E-3	1425.62	1047.07	1110.24	1253.13	11.68	11.20	13.44	11.20	10	9	10	9	43	34	38	39
Q6IMY8	Heterogeneous nuclear ribonucleoprotein U OS=Rattus norvegicus OX=10116 GN=Hnrnpu PE=1 SV=1 - [HNRPU_RAT]	87.7	4.007E-5	6.666E-5	1.893E-4	8.127E-4	25.50	94.01	278.82	1087.25	1.63	4.64	12.66	28.32	2	2	8	19	4	3	13	48
Q6IRK9	Carboxypeptidase Q OS=Rattus norvegicus GN=Cpq PE=1 SV=1 - [CBPQ_RAT]	52.0	4.283E-4	0.000E0	1.985E-5	5.174E-5	821.30		22.77	92.19	32.20		4.87	2.33	10		1	1	19		1	2
Q6IUU3	Sulfhydryl oxidase 1 OS=Rattus norvegicus GN=Qsox1 PE=1 SV=1 - [QSOX1_RAT]	82.4	1.809E-5	0.000E0	0.000E0	0.000E0	22.12				1.47				1				2			
Q6JE36	Protein NDRG1 OS=Rattus norvegicus GN=Ndrg1 PE=1 SV=1 - [NDRG1_RAT]	42.9	0.000E0	0.000E0	1.465E-4	8.383E-5			24.38	55.44			4.31	16.50			1	4			2	7
Q6LED0	Histone H3.1 OS=Rattus norvegicus PE=1 SV=3 - [H31_RAT]	15.4	9.327E-4	1.109E-2	4.155E-3	1.199E-2	139.13	615.58	379.07	600.26	10.29	55.88	45.59	52.94	2	7	5	6	7	25	21	25
Q6MG08	ATP-binding cassette sub-family F member 1 OS=Rattus norvegicus OX=10116 GN=Abcf1 PE=1 SV=1 - [ABCF1_RAT]	95.2	0.000E0	0.000E0	8.840E-6	2.176E-5			43.52	37.06			1.19	1.19			1	1			1	2
Q6MG60	N(G),N(G)-dimethylarginine dimethylaminohydrolase 2 OS=Rattus norvegicus GN=Ddah2 PE=1 SV=1 - [DDAH2_RAT]	29.7	2.335E-4	1.709E-4	1.940E-4	1.077E-4	331.56	192.99	216.60	92.39	45.96	29.47	37.54	29.47	10	6	7	6	20	10	11	8
Q6MG61	Chloride intracellular channel protein 1 OS=Rattus norvegicus GN=Clic1 PE=1 SV=1 - [CLIC1_RAT]	27.0	0.000E0	8.048E-4	8.781E-4	7.561E-4		1275.89	1066.93	1000.23		77.59	61.41	59.75		14	11	11		42	34	35
Q6MG71	Choline transporter-like protein 4 OS=Rattus norvegicus OX=10116 GN=Slc44a4 PE=2 SV=1 - [CTL4_RAT]	78.7	1.437E-4	6.438E-5	5.891E-5	5.153E-5	190.92	104.45	125.25	73.30	6.08	4.24	6.08	4.24	3	2	3	2	8	4	5	4
Q6MGD0	Protein CutA OS=Rattus norvegicus GN=Cuta PE=1 SV=2 - [CUTA_RAT]	18.6	0.000E0	9.193E-5	8.718E-5	0.000E0		50.12	52.57			39.55	18.08			3	2			5	4	
Q6NX65	Programmed cell death protein 10 OS=Rattus norvegicus GN=Pdcd10 PE=2 SV=1 - [PDC10_RAT]	24.3	0.000E0	5.495E-5	6.032E-5	6.176E-5		40.50	46.15	37.42		6.19	10.95	10.95		1	2	2		2	3	3
Q6NYB7	Ras-related protein Rab-1A OS=Rattus norvegicus GN=Rab1A PE=1 SV=3 - [RAB1A_RAT]	22.7	5.565E-4	5.219E-4	8.048E-4	5.510E-4	481.64	618.41	960.50	489.60	55.61	60.00	60.49	56.10	9	9	10	10	21	23	26	19
Q6P0K8	Junction plakoglobin OS=Rattus norvegicus GN=Jup PE=1 SV=1 - [PLAK_RAT]	81.7	1.519E-4	1.582E-4	1.688E-4	1.412E-4	288.67	428.61	816.26	486.03	17.58	23.76	30.47	23.76	12	13	15	12	20	20	27	21
Q6P4Z9	COP9 signalosome complex subunit 8 OS=Rattus norvegicus GN=Cops8 PE=2 SV=1 - [CSN8_RAT]	23.2	0.000E0	5.859E-5	6.299E-5	3.612E-5		139.02	127.58	62.09		6.70	6.70	6.70		1	1	1		2	2	2
Q6P502	T-complex protein 1 subunit gamma OS=Rattus norvegicus GN=Cct3 PE=1 SV=1 - [TCPG_RAT]	60.6	0.000E0	7.289E-5	3.561E-4	2.116E-4		368.33	792.12	551.44		11.38	44.40	30.64		6	17	13		12	30	22
Q6P686	Osteoclast-stimulating factor 1 OS=Rattus norvegicus GN=Ostf1 PE=1 SV=1 - [OSTF1_RAT]	23.7	0.000E0	1.690E-4	1.888E-4	1.524E-4		284.93	276.10	155.85		42.99	42.52	27.57		7	6	4		12	11	6
Q6P6Q2	Keratin, type II cytoskeletal 5 OS=Rattus norvegicus GN=Krt5 PE=1 SV=1 - [K2C5_RAT]	61.8	8.563E-3	2.093E-3	2.954E-3	4.022E-3	2125.54	936.17	1150.37	1397.46	27.60	17.88	22.92	26.56	27	17	19	23	91	45	48	61
Q6P6R2	Dihydrolipoyl dehydrogenase, mitochondrial OS=Rattus norvegicus GN=Dld PE=1 SV=1 - [DLDH_RAT]	54.0	4.901E-5	3.245E-4	3.680E-4	2.510E-4	28.40	1279.88	1112.10	665.64	3.73	41.26	40.86	32.42	1	13	12	10	1	37	31	24
Q6P6S9	Ectonucleoside triphosphate diphosphohydrolase 5 OS=Rattus norvegicus OX=10116 GN=Entpd5 PE=2 SV=1 - [ENTP5_R	47.3	4.285E-5	0.000E0	3.735E-5	0.000E0	45.28		26.30		19.67		11.94		5		3		5		3	
Q6P6T4	Echinoderm microtubule-associated protein-like 2 OS=Rattus norvegicus GN=Eml2 PE=1 SV=1 - [EMAL2_RAT]	70.7	0.000E0	3.448E-5	7.213E-5	3.365E-5		92.71	257.28	123.04		7.24	18.03	12.02		4	8	6		5	12	10
Q6P6V0	Glucose-6-phosphate isomerase OS=Rattus norvegicus GN=Gpi PE=1 SV=1 - [G6PI_RAT]	62.8	2.561E-3	1.011E-3	1.339E-3	1.055E-3	2404.13	1416.46	1805.53	1433.80	53.58	50.36	58.60	57.35	26	23	25	24	109	78	84	77
Q6P734	Plasma protease C1 inhibitor OS=Rattus norvegicus GN=Serping1 PE=2 SV=1 - [IC1_RAT]	55.6	1.585E-4	3.639E-5	1.222E-4	0.000E0	288.68	23.03	90.25		16.27	2.18	5.56		6	1	2		10	2	3	
Q6P742	Proteolipid protein 2 OS=Rattus norvegicus GN=Plp2 PE=2 SV=1 - [PLP2_RAT]	16.5	5.354E-5	4.647E-5	3.310E-5	0.000E0	49.60	54.30	45.85		7.95	7.95	7.95		1	1	1		3	2	2	
Q6P747	Heterochromatin protein 1-binding protein 3 OS=Rattus norvegicus OX=10116 GN=Hp1bp3 PE=1 SV=1 - [HP1B3_RAT]	60.8	0.000E0	0.000E0	0.000E0	5.182E-5				30.03				1.45				1				2
Q6P767	Pituitary tumor-transforming gene 1 protein-interacting protein OS=Rattus norvegicus GN=Pttg1ip PE=2 SV=1 - [PTTG_R	19.9	1.180E-4	0.000E0	0.000E0	5.242E-5	54.15			35.69	4.60			4.60	1			1	2			1

Q6P799	Serine--tRNA ligase, cytoplasmic OS=Rattus norvegicus GN=Sars PE=1 SV=3 - [SYSC_RAT]	58.6	0.000E0	3.099E-4	3.878E-4	2.650E-4		620.13	748.57	508.23		36.13	37.89	28.52		13	14	12		24	28	24
Q6P7A9	Lysosomal alpha-glucosidase OS=Rattus norvegicus GN=Gaa PE=2 SV=1 - [LYAG_RAT]	106.1	5.072E-5	3.815E-5	9.811E-5	9.962E-5	101.58	98.05	420.86	310.34	9.02	6.30	9.86	15.84	7	4	7	10	9	6	15	17
Q6P7B0	Tryptophan--tRNA ligase, cytoplasmic OS=Rattus norvegicus GN=Wars PE=1 SV=2 - [SYWC_RAT]	54.1	0.000E0	0.000E0	6.236E-5	6.181E-5			25.58	23.95			7.07	3.33			2	1			3	1
Q6P7P5	Basic leucine zipper and W2 domain-containing protein 1 OS=Rattus norvegicus GN=Bzw1 PE=2 SV=1 - [BZW1_RAT]	48.0	5.701E-5	5.526E-5	1.719E-4	9.844E-5	71.13	126.07	177.98	218.90	3.58	6.68	20.53	10.50	2	3	8	5	3	5	12	9
Q6P7Q1	BRCA1-A complex subunit BRE OS=Rattus norvegicus GN=Bre PE=2 SV=1 - [BRE_RAT]	43.5	0.000E0	0.000E0	0.000E0	2.903E-5				22.58				2.61				1				1
Q6P7Q4	Lactoylglutathione lyase OS=Rattus norvegicus GN=Glo1 PE=1 SV=3 - [LGUL_RAT]	20.8	2.997E-4	2.034E-4	2.065E-4	1.523E-4	431.74	380.23	339.43	225.13	57.61	50.00	50.00	29.35	9	7	7	5	22	15	15	9
Q6P7S1	Acid ceramidase OS=Rattus norvegicus GN=Asah1 PE=2 SV=1 - [ASAH1_RAT]	44.4	1.651E-4	7.504E-5	1.121E-4	1.120E-4	176.62	79.54	162.23	126.53	19.04	11.42	17.01	13.20	6	3	5	4	10	4	7	5
Q6P9T8	Tubulin beta-4B chain OS=Rattus norvegicus GN=Tubb4b PE=1 SV=1 - [TBB4B_RAT]	49.8	3.773E-4	3.920E-4	9.023E-4	1.066E-3	614.56	759.22	1753.06	2355.24	24.94	29.66	41.12	60.67	10	12	16	21	29	36	69	93
Q6P9U8	Eukaryotic translation initiation factor 3 subunit H OS=Rattus norvegicus GN=Eif3h PE=1 SV=1 - [EIF3H_RAT]	39.9	0.000E0	3.099E-5	8.755E-5	3.230E-5		70.10	160.25	32.78		7.10	14.20	3.13		2	3	1		3	6	2
Q6PCU2	V-type proton ATPase subunit E 1 OS=Rattus norvegicus GN=Atp6v1e1 PE=1 SV=1 - [VATE1_RAT]	26.1	2.978E-5	3.540E-5	1.196E-4	8.115E-5	18.06	86.24	175.84	103.71	6.19	15.04	23.89	15.04	1	3	4	3	1	5	7	6
Q6PDU1	Serine/arginine-rich splicing factor 2 OS=Rattus norvegicus GN=Srsf2 PE=1 SV=3 - [SRSF2_RAT]	25.5	8.480E-5	1.500E-4	3.742E-4	5.741E-4	79.64	120.62	388.01	372.72	3.62	11.76	19.46	19.46	1	3	4	4	2	5	10	12
Q6PDV7	60S ribosomal protein L10 OS=Rattus norvegicus GN=Rpl10 PE=1 SV=3 - [RL10_RAT]	24.6	0.000E0	7.957E-5	1.659E-4	1.740E-4		113.56	286.30	143.58		26.17	38.32	28.50		4	6	5		7	12	12
Q6PEC0	Bis(5'-nucleosyl)-tetraphosphatase [asymmetrical] OS=Rattus norvegicus GN=Nudt2 PE=2 SV=3 - [AP4A_RAT]	16.9	0.000E0	1.842E-4	1.604E-4	1.059E-4		170.53	175.26	112.39		50.34	61.90	46.26		4	6	5		10	11	7
Q6PEC4	S-phase kinase-associated protein 1 OS=Rattus norvegicus GN=Skp1 PE=2 SV=3 - [SKP1_RAT]	18.7	2.692E-4	2.945E-4	2.202E-4	1.683E-4	235.05	237.10	281.59	177.73	23.93	57.06	65.64	31.29	4	7	8	5	8	14	15	9
Q6Q0N1	Cytosolic non-specific dipeptidase OS=Rattus norvegicus GN=Cndp2 PE=1 SV=1 - [CNDP2_RAT]	52.7	3.110E-4	1.564E-3	2.269E-3	1.535E-3	405.36	1822.14	3067.26	1935.17	21.05	62.11	81.26	62.11	7	25	29	25	13	85	113	87
Q6RJR6	Reticulon-3 OS=Rattus norvegicus GN=Rtn3 PE=1 SV=1 - [RTN3_RAT]	101.5	8.703E-5	7.885E-5	1.054E-4	8.089E-5	29.79	32.99	43.33	24.28	2.02	1.17	1.17	1.17	2	1	1	1	3	2	2	2
Q6RUV5	Ras-related C3 botulinum toxin substrate 1 OS=Rattus norvegicus GN=Rac1 PE=1 SV=1 - [RAC1_RAT]	21.4	0.000E0	2.733E-4	2.572E-4	3.803E-4		252.66	252.67	250.66		26.04	26.04	33.33		5	5	6		10	9	11
Q6RY07	Acidic mammalian chitinase OS=Rattus norvegicus OX=10116 GN=Chia PE=2 SV=1 - [CHIA_RAT]	51.9	5.142E-5	3.037E-4	7.591E-4	1.050E-3	34.58	312.00	790.20	872.73	1.90	27.27	38.90	35.73	1	9	11	10	2	17	32	32
Q6T3U3	NPC1-like intracellular cholesterol transporter 1 OS=Rattus norvegicus OX=10116 GN=Npc1l1 PE=1 SV=1 - [NPCL1_RAT]	146.3	4.027E-5	0.000E0	0.000E0	0.000E0	101.75				2.03				3				4			
Q6UPE1	Electron transfer flavoprotein-ubiquinone oxidoreductase, mitochondrial OS=Rattus norvegicus OX=10116 GN=Etfdh PE=1	68.2	0.000E0	0.000E0	0.000E0	0.000E0	37.73				1.79				1				1			
Q6URK4	Heterogeneous nuclear ribonucleoprotein A3 OS=Rattus norvegicus GN=Hnrnpa3 PE=1 SV=1 - [ROA3_RAT]	39.6	1.760E-3	5.456E-3	4.469E-3	4.552E-3	1406.80	2520.68	2094.35	2099.82	36.94	39.31	39.31	39.31	18	20	20	19	54	89	79	74
Q6VBQ5	Myeloid-associated differentiation marker OS=Rattus norvegicus GN=Myadm PE=1 SV=1 - [MYADM_RAT]	35.1	0.000E0	1.941E-5	1.550E-5	1.262E-5		67.64	72.97	39.79		5.35	5.35	5.35		1	1	1		2	2	2
Q6VV72	Eukaryotic translation initiation factor 1A OS=Rattus norvegicus OX=10116 GN=Eif1a PE=2 SV=3 - [IF1A_RAT]	16.5	0.000E0	4.756E-5	3.454E-5	5.320E-5		41.77	113.53	39.30		7.64	14.58	7.64		1	2	1		1	4	2
Q6Y1S1	Myeloid differentiation primary response protein MyD88 OS=Rattus norvegicus OX=10116 GN=Myd88 PE=2 SV=2 - [MYD8	33.8	4.135E-5	3.086E-5	0.000E0	0.000E0	42.21	32.97			3.04	3.04			1	1			2	1		
Q711G3	Isoamyl acetate-hydrolyzing esterase 1 homolog OS=Rattus norvegicus GN=Iah1 PE=2 SV=2 - [IAH1_RAT]	28.0	8.144E-4	2.451E-4	2.722E-4	1.890E-4	573.15	284.12	387.11	174.52	65.86	44.58	48.19	36.14	14	8	10	7	34	19	22	15
Q71UF4	Histone-binding protein RBBP7 OS=Rattus norvegicus GN=Rbbp7 PE=2 SV=1 - [RBBP7_RAT]	47.8	7.226E-4	6.098E-4	5.765E-4	3.407E-4	582.16	653.88	660.88	482.14	30.82	34.82	40.00	32.47	10	11	11	9	23	26	24	19
Q75WE7	von Willebrand factor A domain-containing protein 5A OS=Rattus norvegicus GN=Vwa5a PE=2 SV=1 - [VWA5A_RAT]	91.4	1.347E-4	7.316E-5	1.057E-4	6.580E-5	137.52	329.18	407.51	256.47	2.07	12.29	13.02	5.11	2	9	9	4	4	14	16	8
Q78P75	Dynein light chain 2, cytoplasmic OS=Rattus norvegicus GN=Dynll2 PE=1 SV=1 - [DYL2_RAT]	10.3	5.943E-4	3.722E-4	2.565E-4	2.915E-4	225.55	240.33	166.63	205.57	49.44	49.44	49.44	49.44	3	3	3	3	9	7	7	8
Q793F9	Vacuolar protein sorting-associated protein 4A OS=Rattus norvegicus GN=Vps4a PE=2 SV=1 - [VPS4A_RAT]	48.9	0.000E0	0.000E0	0.000E0	3.493E-4				36.53				5.03				2				3
Q794E4	Heterogeneous nuclear ribonucleoprotein F OS=Rattus norvegicus GN=Hnrnpf PE=1 SV=3 - [HNRPF_RAT]	45.7	6.253E-4	1.205E-3	9.983E-4	6.013E-4	787.23	1198.71	1256.15	891.12	31.57	28.43	40.24	42.89	8	8	11	11	27	33	34	34
Q794F9	4F2 cell-surface antigen heavy chain OS=Rattus norvegicus GN=Slc3a2 PE=1 SV=1 - [4F2_RAT]	58.0	2.598E-4	5.141E-5	6.724E-5	0.000E0	508.34	36.88	49.63		35.29	8.73	8.73		13	2	2		23	3	3	
Q7M0E3	Destrin OS=Rattus norvegicus GN=Dstn PE=1 SV=3 - [DEST_RAT]	18.5	6.870E-5	1.176E-3	1.809E-3	1.887E-3	169.04	1336.91	1502.40	1546.78	30.30	75.76	75.15	75.15	5	16	15	15	8	51	58	59
Q7M767	Ubiquitin-conjugating enzyme E2 variant 2 OS=Rattus norvegicus GN=Ube2v2 PE=1 SV=3 - [UB2V2_RAT]	16.3	6.451E-5	4.121E-4	4.054E-4	3.109E-4	35.82	194.86	155.51	158.49	11.72	24.83	20.00	24.83	2	4	3	4	2	9	7	7
Q7TMA5	Apolipoprotein B-100 OS=Rattus norvegicus GN=Apob PE=1 SV=1 - [APOB_RAT]	535.7	3.524E-4	0.000E0	4.393E-5	0.000E0	714.93	22.25	88.93		8.31	0.36	2.49		30	1	7		49	1	10	
Q7TP17	Splicing factor U2AF 26 kDa subunit OS=Rattus norvegicus OX=10116 GN=U2af1l4 PE=2 SV=1 - [U2AF4_RAT]	25.8	0.000E0	2.375E-5	4.443E-5	7.310E-5		32.81	61.71	87.41		4.09	4.09	7.73		1	1	2		1	2	4
Q7TP47	Heterogeneous nuclear ribonucleoprotein Q OS=Rattus norvegicus GN=Syncrip PE=2 SV=1 - [HNRPQ_RAT]	59.7	3.683E-4	9.351E-4	9.049E-4	9.423E-4	298.34	1105.58	1004.14	871.42	17.45	34.33	35.83	30.39	8	17	15	15	15	49	43	45
Q7TP52	Carboxymethylenebutenolidase homolog OS=Rattus norvegicus OX=10116 GN=Cmbl PE=2 SV=1 - [CMBL_RAT]	27.9	1.101E-4	1.166E-4	8.962E-5	8.152E-5	113.75	115.52	80.57	57.63	24.49	21.63	14.69	9.80	5	4	3	2	8	7	4	3
Q7TP98	Interleukin enhancer-binding factor 2 OS=Rattus norvegicus GN=Ilf2 PE=1 SV=1 - [ILF2_RAT]	51.3	5.568E-5	2.507E-4	2.684E-4	2.693E-4	91.12	322.83	278.96	286.58	4.75	18.79	20.52	10.37	2	6	7	4	3	10	10	8
Q7TPB1	T-complex protein 1 subunit delta OS=Rattus norvegicus GN=Cct4 PE=1 SV=3 - [TCPD_RAT]	58.1	0.000E0	9.987E-5	2.440E-4	1.545E-4		161.70	874.52	308.19		11.69	42.30	32.28		5	16	12		10	36	23
Q7TPJ0	Translocon-associated protein subunit alpha OS=Rattus norvegicus OX=10116 GN=Ssr1 PE=1 SV=1 - [SSRA_RAT]	35.6	0.000E0	3.896E-5	4.196E-5	0.000E0		24.85	47.79			2.51	7.21			1	2			1	2	
Q7TQ20	DnaJ homolog subfamily C member 2 OS=Rattus norvegicus GN=Dnajc2 PE=1 SV=1 - [DNJC2_RAT]	71.7	0.000E0	9.077E-5	0.000E0	0.000E0		25.16				4.19				2				2		
Q7TQ94	Deaminated glutathione amidase OS=Rattus norvegicus OX=10116 GN=Nit1 PE=2 SV=2 - [NIT1_RAT]	36.1	0.000E0	8.789E-5	1.122E-4	8.392E-5		202.37	278.74	163.25		28.75	41.59	24.77		7	9	6		13	15	9
Q7TQM5	Keratinocyte proline-rich protein OS=Rattus norvegicus OX=10116 GN=Kprp PE=2 SV=1 - [KPRP_RAT]	76.3	1.560E-4	0.000E0	0.000E0	0.000E0	63.94				1.43				1				2			
Q80U96	Exportin-1 OS=Rattus norvegicus GN=Xpo1 PE=1 SV=1 - [XPO1_RAT]	123.0	3.065E-5	6.091E-5	1.253E-4	1.189E-4	39.70	165.78	516.48	232.74	0.65	8.78	16.06	11.67	1	6	12	9	2	10	24	15
Q80W57	ATP-binding cassette sub-family G member 2 OS=Rattus norvegicus OX=10116 GN=Abcg2 PE=1 SV=1 - [ABCG2_RAT]	72.9	2.463E-5	3.364E-5	2.494E-5	2.084E-5	23.70	98.35	100.13	39.24	1.83	5.02	3.35	1.52	1	3	2	1	1	4	4	2
Q80ZA5	Sodium-driven chloride bicarbonate exchanger OS=Rattus norvegicus OX=10116 GN=Slc4a10 PE=1 SV=1 - [S4A10_RAT]	125.6	0.000E0	2.137E-5	0.000E0	0.000E0		20.00				0.72				1				2		
Q810F4	Protein FAM3C OS=Rattus norvegicus GN=Fam3c PE=2 SV=1 - [FAM3C_RAT]	24.7	9.318E-5	3.854E-5	2.948E-5	0.000E0	232.48	86.04	31.19		19.82	12.33	7.05		4	2	1		7	3	2	
Q811A3	Procollagen-lysine,2-oxoglutarate 5-dioxygenase 2 OS=Rattus norvegicus GN=Plod2 PE=2 SV=1 - [PLOD2_RAT]	84.5	0.000E0	3.808E-5	5.942E-5	3.367E-5		163.43	186.39	65.75		5.43	12.21	1.63		3	6	1		5	8	2
Q811U3	ELKS/Rab6-interacting/CAST family member 1 OS=Rattus norvegicus GN=Erc1 PE=1 SV=1 - [RB6I2_RAT]	108.8	2.933E-5	0.000E0	0.000E0	0.000E0	0.00				0.95				1				1			
Q811X6	Lambda-crystallin homolog OS=Rattus norvegicus OX=10116 GN=Cryl1 PE=1 SV=3 - [CRYL1_RAT]	35.3	0.000E0	7.730E-5	1.305E-4	1.065E-4		163.49	312.81	118.41		31.97	34.80	21.63		7	8	5		10	15	8
Q812D3	Peptidyl-prolyl cis-trans isomerase-like 3 OS=Rattus norvegicus GN=Ppil3 PE=2 SV=1 - [PPIL3_RAT]	18.1	0.000E0	1.093E-4	0.000E0	0.000E0		71.01				11.80				1				2		
Q8CFD1	RNA-binding protein 45 OS=Rattus norvegicus OX=10116 GN=Rbm45 PE=1 SV=1 - [RBM45_RAT]	53.4	0.000E0	2.229E-5	0.000E0	0.000E0		57.49				5.25				2				3		
Q8CFN2	Cell division control protein 42 homolog OS=Rattus norvegicus GN=Cdc42 PE=1 SV=2 - [CDC42_RAT]	21.2	0.000E0	2.554E-4	3.002E-4	3.627E-4		434.80	586.70	645.99		38.74	58.64	62.83		6	8	10		17	21	27
Q8CG45	Aflatoxin B1 aldehyde reductase member 2 OS=Rattus norvegicus GN=Akr7a2 PE=1 SV=2 - [ARK72_RAT]	40.6	1.657E-4	5.369E-4	5.905E-4	6.292E-4	250.81	717.61	756.95	688.22	19.07	53.95	60.22	56.68	6	13	15	15	11	32	29	28
Q8CGS4	Charged multivesicular body protein 3 OS=Rattus norvegicus GN=Chmp3 PE=1 SV=3 - [CHMP3_RAT]	25.0	0.000E0	4.017E-5	1.096E-5	2.187E-5		22.91	35.30	41.07		4.04	3.14	3.14		1	1	1		1	1	1
Q8CGU6	Nicastrin OS=Rattus norvegicus GN=Ncstn PE=1 SV=1 - [NICA_RAT]	78.4	8.043E-5	5.055E-5	6.756E-5	5.059E-5	66.37	75.15	134.41	82.00	6.07	4.94	4.94	4.94	3	2	2	2	4	3	3	3
Q8CIZ5	Deleted in malignant brain tumors 1 protein OS=Rattus norvegicus OX=10116 GN=Dmbt1 PE=1 SV=1 - [DMBT1_RAT]	155.6	2.396E-4	1.802E-4	1.066E-4	1.734E-4	392.65	309.94	190.36	369.35	11.07	13.12	8.11	11.14	10	12	6	11	18	17	8	19
Q8CJD3	Zymogen granule membrane protein 16 OS=Rattus norvegicus GN=Zg16 PE=2 SV=1 - [ZG16_RAT]	18.2	1.166E-4	1.298E-4	1.438E-4	1.119E-4	50.66	74.96	32.32	56.33	9.58	22.75	9.58	16.17	1	2	1	2	2	4	1	2
Q8K1P7	Transcription activator BRG1 OS=Rattus norvegicus OX=10116 GN=Smarca4 PE=1 SV=1 - [SMCA4_RAT]	181.3	0.000E0	2.045E-5	0.000E0	3.507E-5		32.48		96.45		0.74		1.18		1		2		1		3
Q8K1Q0	Glycylpeptide N-tetradecanoyltransferase 1 OS=Rattus norvegicus GN=Nmt1 PE=1 SV=1 - [NMT1_RAT]	56.8	0.000E0	3.889E-5	5.398E-5	8.958E-5		71.84	110.38	40.52		6.65	10.69	3.83		2	3	1		4	4	1
Q8K3K9	GTPase IMAP family member 4 OS=Rattus norvegicus GN=Gimap4 PE=1 SV=1 - [GIMA4_RAT]	35.8	6.071E-4	5.777E-4	1.802E-4	3.025E-4	20.97	73.39	37.28	46.02	6.13	15.16	10.00	10.00	2	4	3	3	6	9	6	8
Q8K3Y6	Zinc finger CCCH-type antiviral protein 1 OS=Rattus norvegicus OX=10116 GN=Zc3hav1 PE=1 SV=1 - [ZCCHV_RAT]	86.7	0.000E0	5.700E-5	0.000E0	0.000E0		35.40				2.45				1				1		
Q8K4F7	m7GpppX diphosphatase OS=Rattus norvegicus GN=Dcps PE=2 SV=1 - [DCPS_RAT]	38.7	2.100E-4	2.216E-4	2.492E-4	2.169E-4	419.79	466.61	392.98	358.41	39.58	41.37	35.42	39.58	10	10	8	10	22	21	16	19
Q8R424	STAM-binding protein OS=Rattus norvegicus OX=10116 GN=Stambp PE=2 SV=1 - [STABP_RAT]	48.5	3.706E-5	0.000E0	3.706E-5	0.000E0	60.66		35.76		8.49		2.83		3		1		5		2	
Q8R478	WW domain-binding protein 2 OS=Rattus norvegicus GN=Wbp2 PE=1 SV=1 - [WBP2_RAT]	28.1	0.000E0	0.000E0	0.000E0	0.000E0				20.85				3.82				1				1
Q8R4A1	ERO1-like protein alpha OS=Rattus norvegicus GN=Ero1l PE=1 SV=1 - [ERO1A_RAT]	54.0	0.000E0	3.754E-5	6.044E-5	2.698E-5		78.14	130.87	45.21		7.33	15.30	5.39		3	6	3		6	8	4
Q8VD91	Ribonuclease pancreatic alpha-type OS=Rattus fuscipes OX=10119 PE=3 SV=1 - [RNS1A_RATFU]	16.6	5.594E-5	0.000E0	0.000E0	0.000E0	47.79				16.11				2				4			
Q8VHF5	Citrate synthase, mitochondrial OS=Rattus norvegicus GN=Cs PE=1 SV=1 - [CISY_RAT]	51.8	3.109E-5	6.027E-4	6.531E-4	5.139E-4	35.22	840.85	906.86	620.09	2.58	33.48	38.84	30.90	1	12	14	11	2	31	31	25
Q8VHT6	Arsenite methyltransferase OS=Rattus norvegicus OX=10116 GN=As3mt PE=1 SV=1 - [AS3MT_RAT]	41.0	0.000E0	8.739E-5	1.086E-4	6.100E-5		92.57	79.37	41.59		7.32	17.89	7.32		2	5	2		4	5	3
Q8VHV7	Heterogeneous nuclear ribonucleoprotein H OS=Rattus norvegicus GN=Hnrnph1 PE=1 SV=2 - [HNRH1_RAT]	49.2	1.233E-3	1.907E-3	1.676E-3	1.632E-3	968.93	1360.46	1350.80	1325.10	26.73	28.73	28.73	26.95	10	11	11	10	35	43	41	35
Q8VID1	Dehydrogenase/reductase SDR family member 4 OS=Rattus norvegicus GN=Dhrs4 PE=2 SV=2 - [DHRS4_RAT]	29.8	3.066E-4	6.330E-5	9.114E-5	8.349E-5	390.43	66.85	82.44	40.32	31.18	11.11	18.64	18.64	8	3	4	4	16	4	7	6
Q8VIF7	Selenium-binding protein 1 OS=Rattus norvegicus GN=Selenbp1 PE=1 SV=1 - [SBP1_RAT]	52.5	1.363E-4	4.767E-4	4.466E-4	9.962E-4	223.10	1070.16	1190.54	1676.30	18.22	61.23	67.16	75.64	6	21	23	27	10	44	48	67
Q91V33	KH domain-containing, RNA-binding, signal transduction-associated protein 1 OS=Rattus norvegicus GN=Khdrbs1 PE=1 SV	48.3	2.969E-4	2.102E-4	2.455E-4	3.565E-4	123.36	183.54	172.65	189.13	5.42	11.96	11.96	11.96	2	4	4	4	6	9	11	10
Q91XT9	Neutral ceramidase OS=Rattus norvegicus OX=10116 GN=Asah2 PE=1 SV=1 - [ASAH2_RAT]	83.4	6.367E-4	2.029E-4	2.405E-4	3.197E-4	984.30	405.90	525.50	629.00	30.22	17.08	18.53	24.44	14	9	9	12	33	17	18	25
Q91Y78	Ubiquitin carboxyl-terminal hydrolase isozyme L3 OS=Rattus norvegicus GN=Uchl3 PE=1 SV=1 - [UCHL3_RAT]	26.1	0.000E0	7.766E-5	9.686E-5	7.439E-5		56.60	122.66	29.77		15.65	21.30	8.70		2	3	1		3	5	1
Q91Y81	Septin-2 OS=Rattus norvegicus GN=Sept2 PE=1 SV=1 - [SEPT2_RAT]	41.6	3.740E-4	2.168E-4	2.785E-4	1.136E-4	538.93	423.13	493.84	106.27	28.53	28.53	31.58	18.56	7	7	8	5	15	13	16	8
Q91ZN1	Coronin-1A OS=Rattus norvegicus GN=Coro1a PE=1 SV=3 - [COR1A_RAT]	51.0	0.000E0	3.959E-5	1.197E-4	3.914E-5		153.90	278.09	62.69		7.16	26.25	11.28		3	9	4		5	13	4
Q91ZW6	Trimethyllysine dioxygenase, mitochondrial OS=Rattus norvegicus OX=10116 GN=Tmlhe PE=1 SV=2 - [TMLH_RAT]	49.5	0.000E0	8.595E-5	7.967E-5	0.000E0		54.26	68.79			7.13	7.13			3	3			5	5	
Q920A6	Retinoid-inducible serine carboxypeptidase OS=Rattus norvegicus GN=Scpep1 PE=2 SV=1 - [RISC_RAT]	51.1	1.942E-4	5.332E-5	9.805E-5	1.249E-4	274.47	21.43	122.50	154.52	12.17	3.10	14.60	19.47	5	1	5	7	17	1	13	15
Q920D2	Dihydrofolate reductase OS=Rattus norvegicus GN=Dhfr PE=2 SV=3 - [DYR_RAT]	21.6	5.802E-5	2.839E-4	3.083E-4	3.108E-4	39.81	494.08	561.38	317.52	33.16	64.71	64.71	42.25	5	9	9	7	6	20	22	16
Q920H8	Hephaestin OS=Rattus norvegicus GN=Heph PE=1 SV=1 - [HEPH_RAT]	129.5	1.877E-4	6.898E-5	8.197E-5	0.000E0	434.05	274.00	409.14		10.54	9.25	11.58		8	7	9		19	12	15	
Q920J4	Thioredoxin-like protein 1 OS=Rattus norvegicus GN=Txnl1 PE=1 SV=3 - [TXNL1_RAT]	32.2	9.306E-5	2.650E-4	2.987E-4	1.606E-4	70.30	294.51	427.68	97.71	12.80	39.45	43.25	27.68	2	7	8	5	4	10	18	8
Q920L2	Succinate dehydrogenase [ubiquinone] flavoprotein subunit, mitochondrial OS=Rattus norvegicus GN=Sdha PE=1 SV=1 -	71.6	8.991E-5	2.487E-4	2.125E-4	1.650E-4	220.10	733.59	715.55	462.84	16.31	36.13	37.20	29.57	8	15	14	12	14	32	30	20
Q920P6	Adenosine deaminase OS=Rattus norvegicus GN=Ada PE=1 SV=3 - [ADA_RAT]	39.9	6.592E-4	4.839E-4	5.281E-4	4.241E-4	732.21	630.92	719.99	630.45	59.66	50.00	51.14	53.41	15	12	12	12	31	32	27	25
Q921A4	Cytoglobin OS=Rattus norvegicus OX=10116 GN=Cygb PE=1 SV=1 - [CYGB_RAT]	21.5	0.000E0	0.000E0	5.776E-5	6.389E-5			30.46	33.18			4.74	4.74			1	1			1	2
Q923J6	Dynein heavy chain 12, axonemal OS=Rattus norvegicus GN=Dnah12 PE=2 SV=2 - [DYH12_RAT]	357.0	2.241E-4	0.000E0	0.000E0	0.000E0	25.62				0.23				1				2			
Q923M1	Mitochondrial peptide methionine sulfoxide reductase OS=Rattus norvegicus OX=10116 GN=Msra PE=1 SV=1 - [MSRA_RA	25.8	0.000E0	4.086E-5	0.000E0	0.000E0		65.10				9.44				2				2		
Q923V4	F-box only protein 6 OS=Rattus norvegicus GN=Fbxo6 PE=1 SV=1 - [FBX6_RAT]	32.8	1.586E-5	0.000E0	2.261E-5	0.000E0	50.12		24.60		7.04		5.99		2		2		4		3	
Q923V8	Selenoprotein F OS=Rattus norvegicus OX=10116 GN=Selenof PE=1 SV=3 - [SEP15_RAT]	17.8	0.000E0	1.598E-5	0.000E0	0.000E0		36.95				7.41				1				2		
Q924S5	Lon protease homolog, mitochondrial OS=Rattus norvegicus GN=Lonp1 PE=2 SV=1 - [LONM_RAT]	105.7	9.893E-5	7.345E-5	9.848E-5	4.787E-5	25.39	76.96	102.09	22.45	9.05	6.63	10.32	5.16	6	4	6	3	9	5	10	3
Q925G0	RNA-binding protein 3 OS=Rattus norvegicus GN=Rbm3 PE=1 SV=2 - [RBM3_RAT]	16.8	0.000E0	8.571E-6	0.000E0	2.115E-5		33.51		47.32		6.45		6.45		1		1		1		2
Q99068	Alpha-2-macroglobulin receptor-associated protein OS=Rattus norvegicus OX=10116 GN=Lrpap1 PE=1 SV=2 - [AMRP_RA	42.0	0.000E0	0.000E0	3.769E-5	4.540E-5			57.93	68.53			3.61	9.44			1	3			2	3
Q99J86	Attractin OS=Rattus norvegicus GN=Atrn PE=2 SV=1 - [ATRN_RAT]	158.6	7.024E-5	0.000E0	0.000E0	0.000E0	212.63				3.70				4				7			
Q99JE6	1-phosphatidylinositol 4,5-bisphosphate phosphodiesterase beta-3 OS=Rattus norvegicus GN=Plcb3 PE=1 SV=2 - [PLCB3_	139.4	3.435E-5	3.758E-4	3.095E-4	2.281E-4	51.63	995.39	851.77	592.80	2.27	24.07	22.69	15.64	3	21	19	17	5	42	37	33
Q99M63	WD40 repeat-containing protein SMU1 OS=Rattus norvegicus GN=Smu1 PE=2 SV=1 - [SMU1_RAT]	57.5	0.000E0	4.153E-5	4.050E-5	5.334E-5	32.33	85.42	73.48	44.77	1.95	4.48	1.95	4.48	1	2	1	2	1	3	2	4
Q99MA2	Xaa-Pro aminopeptidase 2 OS=Rattus norvegicus OX=10116 GN=Xpnpep2 PE=1 SV=1 - [XPP2_RAT]	76.0	2.245E-4	0.000E0	5.281E-6	0.000E0	690.82		27.08		34.27		2.08		17		1		32		1	
Q99MI7	NEDD8-activating enzyme E1 catalytic subunit OS=Rattus norvegicus GN=Uba3 PE=1 SV=1 - [UBA3_RAT]	51.7	0.000E0	6.638E-5	7.974E-5	5.651E-5		99.28	167.15	55.99		14.07	16.88	5.63		4	5	2		6	8	3
Q99N27	Sorting nexin-1 OS=Rattus norvegicus GN=Snx1 PE=1 SV=1 - [SNX1_RAT]	59.0	7.028E-5	5.311E-5	8.599E-5	8.083E-5	101.11	80.53	115.28	104.02	2.11	2.11	2.11	4.79	1	1	1	2	2	2	2	3
Q99NA5	Isocitrate dehydrogenase [NAD] subunit alpha, mitochondrial OS=Rattus norvegicus GN=Idh3a PE=1 SV=1 - [IDH3A_RAT	39.6	0.000E0	3.191E-5	9.768E-5	8.214E-5		26.60	364.56	66.06		2.73	20.22	11.75		1	6	3		1	10	5
Q99ND9	RWD domain-containing protein 1 OS=Rattus norvegicus GN=Rwdd1 PE=2 SV=1 - [RWDD1_RAT]	27.8	7.484E-5	9.388E-5	8.436E-5	6.593E-5	57.50	51.12	67.07	59.66	3.70	3.70	3.70	3.70	1	1	1	1	2	1	2	2
Q99P99	Histone deacetylase 4 OS=Rattus norvegicus GN=Hdac4 PE=1 SV=2 - [HDAC4_RAT]	118.6	0.000E0	5.474E-3	0.000E0	7.002E-3		21.55		30.46		0.65		0.65		1		1		4		3
Q99PD4	Actin-related protein 2/3 complex subunit 1A OS=Rattus norvegicus GN=Arpc1a PE=2 SV=1 - [ARC1A_RAT]	41.6	0.000E0	1.312E-4	1.611E-4	5.369E-5		58.72	193.88	42.55		9.46	15.95	5.41		2	4	2		3	7	3
Q99PD6	Transforming growth factor beta-1-induced transcript 1 protein OS=Rattus norvegicus OX=10116 GN=Tgfb1i1 PE=1 SV=2	50.1	0.000E0	0.000E0	0.000E0	8.556E-6				20.82				1.95				1				1
Q99PF5	Far upstream element-binding protein 2 OS=Rattus norvegicus GN=Khsrp PE=1 SV=1 - [FUBP2_RAT]	74.2	0.000E0	0.000E0	4.064E-5	1.758E-4			122.23	318.94			4.72	9.57			3	6			4	12
Q99PS8	Histidine-rich glycoprotein OS=Rattus norvegicus GN=Hrg PE=1 SV=1 - [HRG_RAT]	59.0	2.189E-5	0.000E0	0.000E0	0.000E0	25.51				1.90				1				1			
Q99PW3	Sialidase-1 OS=Rattus norvegicus GN=Neu1 PE=1 SV=1 - [NEUR1_RAT]	44.7	1.287E-3	2.844E-4	4.731E-4	2.671E-4	732.77	305.94	449.01	215.75	35.94	21.03	37.41	24.69	13	7	11	7	32	14	21	12
Q9EPB1	Dipeptidyl peptidase 2 OS=Rattus norvegicus GN=Dpp7 PE=1 SV=1 - [DPP2_RAT]	55.1	2.233E-4	3.370E-4	6.598E-4	6.800E-4	213.36	610.30	900.06	736.69	13.60	33.20	33.20	27.80	5	10	11	9	12	24	27	28
Q9EPC6	Profilin-2 OS=Rattus norvegicus GN=Pfn2 PE=1 SV=3 - [PROF2_RAT]	15.0	0.000E0	5.665E-5	5.475E-5	6.042E-5		27.97	55.04	24.34		19.29	20.71	10.00		2	2	1		2	2	1
Q9EPF2	Cell surface glycoprotein MUC18 OS=Rattus norvegicus OX=10116 GN=Mcam PE=1 SV=2 - [MUC18_RAT]	71.3	1.172E-4	1.430E-4	1.530E-4	8.115E-5	87.95	141.25	335.90	111.99	4.78	6.64	15.59	6.79	2	3	6	3	4	6	12	5
Q9EPH8	Polyadenylate-binding protein 1 OS=Rattus norvegicus GN=Pabpc1 PE=1 SV=1 - [PABP1_RAT]	70.7	1.621E-3	1.894E-3	1.460E-3	1.500E-3	1968.77	2474.31	2080.20	1971.51	33.49	33.18	33.96	33.65	24	24	24	24	74	80	71	71
Q9EPJ0	Nuclear ubiquitous casein and cyclin-dependent kinase substrate 1 OS=Rattus norvegicus OX=10116 GN=Nucks1 PE=1 SV	27.1	0.000E0	8.808E-5	9.533E-5	1.706E-4		85.96	58.07	65.01		3.70	3.70	3.70		1	1	1		2	2	2
Q9EPT8	Chloride intracellular channel protein 5 OS=Rattus norvegicus GN=Clic5 PE=1 SV=1 - [CLIC5_RAT]	28.3	0.000E0	1.106E-4	1.277E-4	7.802E-5		449.65	401.58	251.85		48.61	44.22	39.04		10	8	7		14	14	9
Q9EQH5	C-terminal-binding protein 2 OS=Rattus norvegicus GN=Ctbp2 PE=1 SV=2 - [CTBP2_RAT]	49.0	0.000E0	8.410E-5	8.265E-5	8.336E-5		31.20	29.07	27.14		8.31	4.04	6.07		3	2	2		4	2	3
Q9EQS0	Transaldolase OS=Rattus norvegicus GN=Taldo1 PE=1 SV=2 - [TALDO_RAT]	37.4	7.212E-4	9.411E-4	1.110E-3	1.072E-3	933.90	987.44	1224.83	1065.37	41.25	43.62	46.59	43.62	15	16	18	16	34	41	49	41
Q9EQT5	Tubulointerstitial nephritis antigen-like OS=Rattus norvegicus GN=Tinagl1 PE=2 SV=1 - [TINAL_RAT]	52.8	0.000E0	3.263E-5	2.345E-5	2.551E-5		100.39	80.53	104.90		2.78	2.78	4.71		1	1	2		2	1	3
Q9EQV6	Tripeptidyl-peptidase 1 OS=Rattus norvegicus GN=Tpp1 PE=1 SV=1 - [TPP1_RAT]	61.3	4.117E-5	0.000E0	0.000E0	0.000E0	46.19				4.97				2				3			
Q9EQX9	Ubiquitin-conjugating enzyme E2 N OS=Rattus norvegicus GN=Ube2n PE=1 SV=1 - [UBE2N_RAT]	17.1	1.968E-4	4.074E-4	4.376E-4	6.116E-4	189.81	288.82	375.77	349.12	33.55	48.03	66.45	48.03	5	7	8	7	10	13	16	13
Q9ER24	Ataxin-10 OS=Rattus norvegicus GN=Atxn10 PE=1 SV=1 - [ATX10_RAT]	53.7	5.200E-5	0.000E0	6.169E-5	4.543E-5	49.52		20.28	25.36	2.53		6.74	2.53	1		2	1	2		2	1
Q9ER34	Aconitate hydratase, mitochondrial OS=Rattus norvegicus GN=Aco2 PE=1 SV=2 - [ACON_RAT]	85.4	1.530E-5	1.152E-3	1.461E-3	1.073E-3	37.94	2789.26	3090.38	1845.75	1.03	59.87	61.41	54.74	1	37	38	32	1	102	105	76
Q9ERB4	Versican core protein (Fragments) OS=Rattus norvegicus GN=Vcan PE=2 SV=2 - [CSPG2_RAT]	299.8	2.881E-5	6.378E-5	9.925E-5	7.346E-5	21.16	141.71	233.22	135.47	0.77	1.68	3.65	2.52	2	4	7	6	2	7	10	9
Q9ES40	PRA1 family protein 3 OS=Rattus norvegicus OX=10116 GN=Arl6ip5 PE=1 SV=1 - [PRAF3_RAT]	21.5	2.535E-5	0.000E0	0.000E0	0.000E0	24.34				5.85				1				1			
Q9ES53	Ubiquitin fusion degradation protein 1 homolog OS=Rattus norvegicus GN=Ufd1l PE=1 SV=1 - [UFD1_RAT]	34.5	3.124E-5	4.057E-5	3.664E-5	0.000E0	25.03	66.02	21.33		9.12	8.47	4.89		2	2	1		2	3	2	
Q9ES54	Nuclear protein localization protein 4 homolog OS=Rattus norvegicus GN=Nploc4 PE=1 SV=3 - [NPL4_RAT]	68.0	0.000E0	4.145E-5	6.267E-5	4.657E-5		42.75	81.26	24.55		2.63	1.64	1.64		2	1	1		3	2	1
Q9ESH6	Glutaredoxin-1 OS=Rattus norvegicus GN=Glrx PE=3 SV=3 - [GLRX1_RAT]	11.9	3.209E-4	3.444E-4	1.977E-4	1.918E-4	232.35	209.67	313.54	171.93	70.09	57.94	38.32	31.78	5	5	3	3	9	10	11	7
Q9ESS6	Basal cell adhesion molecule OS=Rattus norvegicus OX=10116 GN=Bcam PE=2 SV=1 - [BCAM_RAT]	67.5	0.000E0	1.649E-5	2.184E-5	0.000E0		56.19	30.74			5.29	3.04			2	1			2	1	
Q9EST6	Acidic leucine-rich nuclear phosphoprotein 32 family member B OS=Rattus norvegicus GN=Anp32b PE=2 SV=1 - [AN32B_	31.0	1.780E-4	1.133E-3	7.395E-4	7.993E-4	269.26	617.14	454.88	443.43	19.49	23.90	22.79	19.49	4	5	6	4	7	16	10	11
Q9ESV1	Leucine zipper protein 1 OS=Rattus norvegicus OX=10116 GN=Luzp1 PE=1 SV=1 - [LUZP1_RAT]	117.1	0.000E0	2.837E-5	3.037E-5	0.000E0		22.37	0.00			0.67	0.67			1	1			1	1	
Q9ESW0	DNA damage-binding protein 1 OS=Rattus norvegicus GN=Ddb1 PE=2 SV=1 - [DDB1_RAT]	126.8	1.421E-4	1.843E-4	2.132E-4	1.474E-4	407.33	486.07	730.95	443.83	13.77	19.30	22.72	17.89	13	17	20	15	25	27	34	26
Q9ET61	Complement component C1q receptor OS=Rattus norvegicus OX=10116 GN=Cd93 PE=1 SV=1 - [C1QR1_RAT]	68.7	5.183E-5	0.000E0	0.000E0	0.000E0	42.69				1.71				1				2			
Q9HB97	Alpha-parvin OS=Rattus norvegicus GN=Parva PE=1 SV=2 - [PARVA_RAT]	42.3	3.176E-4	1.779E-4	3.197E-5	1.051E-4	38.11	24.86	41.43	102.50	10.22	5.65	11.02	10.22	4	2	3	4	9	3	3	7
Q9JHB5	Translin-associated protein X OS=Rattus norvegicus GN=Tsnax PE=1 SV=1 - [TSNAX_RAT]	33.0	0.000E0	0.000E0	4.108E-5	5.868E-5			46.09	23.42			15.52	6.90			3	1			5	1
Q9JHL4	Drebrin-like protein OS=Rattus norvegicus GN=Dbnl PE=1 SV=1 - [DBNL_RAT]	48.6	0.000E0	4.562E-5	5.825E-5	2.378E-5		115.20	258.54	50.83		8.26	13.30	6.65		3	5	2		3	9	3
Q9JHU5	Arfaptin-1 OS=Rattus norvegicus GN=Arfip1 PE=1 SV=1 - [ARFP1_RAT]	40.8	2.379E-3	1.838E-3	2.020E-3	2.411E-3	37.00	36.93	36.62	47.13	2.73	2.73	2.73	2.73	1	1	1	1	2	2	2	2
Q9JHW0	Proteasome subunit beta type-7 OS=Rattus norvegicus GN=Psmb7 PE=1 SV=1 - [PSB7_RAT]	29.9	0.000E0	5.280E-5	1.050E-4	3.102E-5		53.02	136.91	32.04		11.19	24.55	7.94		3	5	2		4	8	2
Q9JHW1	Carboxypeptidase D OS=Rattus norvegicus GN=Cpd PE=1 SV=2 - [CBPD_RAT]	152.5	1.448E-5	0.000E0	0.000E0	0.000E0	33.35				0.73				1				1			
Q9JHY1	Junctional adhesion molecule A OS=Rattus norvegicus GN=F11r PE=1 SV=1 - [JAM1_RAT]	32.3	8.666E-5	0.000E0	8.778E-5	6.554E-5	32.24		65.57	43.21	11.67		11.67	11.67	2		2	2	3		2	2
Q9JI85	Nucleobindin-2 OS=Rattus norvegicus GN=Nucb2 PE=1 SV=1 - [NUCB2_RAT]	50.1	5.055E-5	1.920E-5	3.286E-5	2.751E-5	24.85	27.97	38.56	38.17	7.14	2.14	5.00	2.14	2	1	1	1	4	2	1	2
Q9JID2	Guanine nucleotide-binding protein subunit alpha-11 OS=Rattus norvegicus GN=Gna11 PE=2 SV=1 - [GNA11_RAT]	42.0	9.210E-5	9.656E-5	1.056E-4	3.676E-5	134.11	118.83	177.20	42.11	20.33	17.55	18.66	5.29	6	4	5	1	10	7	8	1
Q9JIK1	Cadherin-related family member 5 OS=Rattus norvegicus GN=Cdhr5 PE=1 SV=1 - [CDHR5_RAT]	90.9	1.189E-3	2.975E-4	2.357E-4	2.216E-4	652.56	351.79	375.72	243.27	17.29	17.05	13.69	17.29	12	9	8	9	36	15	16	14
Q9JIL3	Interleukin enhancer-binding factor 3 OS=Rattus norvegicus GN=Ilf3 PE=1 SV=2 - [ILF3_RAT]	95.9	8.406E-5	1.006E-4	8.698E-5	7.707E-5	25.92	201.26	261.41	106.26	2.56	8.81	8.92	6.13	2	7	7	5	3	12	11	6
Q9JIX3	Bis(5'-adenosyl)-triphosphatase OS=Rattus norvegicus OX=10116 GN=Fhit PE=1 SV=1 - [FHIT_RAT]	17.3	3.553E-5	0.000E0	0.000E0	0.000E0	99.49				8.67				1				2			
Q9JJ09	Sodium-dependent phosphate transport protein 2B OS=Rattus norvegicus OX=10116 GN=Slc34a2 PE=1 SV=1 - [NPT2B_F	75.9	6.369E-5	0.000E0	0.000E0	0.000E0	86.62				6.33				3				6			
Q9JJ19	Na(+)/H(+) exchange regulatory cofactor NHE-RF1 OS=Rattus norvegicus OX=10116 GN=Slc9a3r1 PE=1 SV=3 - [NHRF1_	38.8	1.560E-4	2.314E-4	1.621E-4	2.156E-4	59.38	76.48	106.55	137.39	3.93	3.93	7.58	19.66	1	1	2	8	2	2	3	12
Q9JJ22	Endoplasmic reticulum aminopeptidase 1 OS=Rattus norvegicus GN=Erap1 PE=2 SV=2 - [ERAP1_RAT]	106.4	2.731E-5	5.518E-5	6.015E-5	3.441E-5	36.01	175.38	307.45	126.01	4.19	8.49	9.14	7.20	3	7	8	6	3	11	14	7
Q9JJ40	Na(+)/H(+) exchange regulatory cofactor NHE-RF3 OS=Rattus norvegicus OX=10116 GN=Pdzk1 PE=1 SV=2 - [NHRF3_RA	56.8	4.655E-4	4.906E-4	5.415E-4	4.249E-4	881.98	883.68	1137.88	747.64	42.45	43.79	48.76	44.55	19	21	23	18	38	42	42	35
Q9JJ50	Hepatocyte growth factor-regulated tyrosine kinase substrate OS=Rattus norvegicus GN=Hgs PE=1 SV=1 - [HGS_RAT]	86.2	0.000E0	2.693E-5	3.850E-5	0.000E0		41.55	45.58			4.38	2.58			2	2			2	4	
Q9JJ54	Heterogeneous nuclear ribonucleoprotein D0 OS=Rattus norvegicus GN=Hnrnpd PE=1 SV=2 - [HNRPD_RAT]	38.2	6.234E-4	2.579E-3	2.107E-3	3.788E-3	439.47	1123.14	1059.74	1170.90	22.66	31.44	31.44	31.44	8	14	14	14	19	40	41	45
Q9JJP9	Ubiquilin-1 OS=Rattus norvegicus GN=Ubqln1 PE=1 SV=1 - [UBQL1_RAT]	62.0	4.505E-5	0.000E0	0.000E0	0.000E0	84.70				2.23				1				2			
Q9JJW3	Up-regulated during skeletal muscle growth protein 5 OS=Rattus norvegicus OX=10116 GN=Atp5md PE=1 SV=1 - [USMG	6.4	0.000E0	0.000E0	0.000E0	4.142E-5				26.75				25.86				1				1
Q9JK72	Copper chaperone for superoxide dismutase OS=Rattus norvegicus GN=Ccs PE=1 SV=1 - [CCS_RAT]	28.9	7.596E-5	0.000E0	5.757E-5	0.000E0	125.88		27.45		10.22		2.55		3		1		4		1	
Q9JKU6	Spermatid perinuclear RNA-binding protein OS=Rattus norvegicus GN=Strbp PE=1 SV=1 - [STRBP_RAT]	74.0	0.000E0	5.305E-5	7.018E-5	5.075E-5		62.21	53.10	24.43		4.92	2.53	4.92		3	2	3		4	2	3

Q9JLA3	UDP-glucose:glycoprotein glucosyltransferase 1 OS=Rattus norvegicus GN=Uggt1 PE=1 SV=2 - [UGGG1_RAT]	176.3	1.955E-5	8.725E-5	9.174E-5	4.647E-5	25.26	513.72	500.80	101.87	0.71	17.02	13.35	4.58	1	19	14	5	1	28	20	8
Q9JLH5	CDK5 regulatory subunit-associated protein 2 OS=Rattus norvegicus OX=10116 GN=Cdk5rap2 PE=1 SV=2 - [CK5P2_RAT]	215.3	1.095E-4	5.974E-5	8.401E-5	0.000E0	48.79	21.43	37.67		0.47	0.47	0.47		1	1	1		2	1	2	
Q9JLJ3	4-trimethylaminobutyraldehyde dehydrogenase OS=Rattus norvegicus GN=Aldh9a1 PE=1 SV=1 - [AL9A1_RAT]	53.6	1.345E-4	1.002E-3	1.165E-3	7.258E-4	225.15	1053.88	1536.57	978.55	19.03	56.88	62.75	51.82	7	21	24	19	11	41	51	39
Q9JLT0	Myosin-10 OS=Rattus norvegicus GN=Myh10 PE=1 SV=1 - [MYH10_RAT]	228.8	6.488E-4	4.874E-4	4.869E-4	8.544E-4	63.03	928.54	913.12	1008.37	0.46	8.35	8.45	10.93	1	18	17	21	2	33	32	44
Q9JLZ1	Glutaredoxin-3 OS=Rattus norvegicus GN=Glrx3 PE=1 SV=2 - [GLRX3_RAT]	37.8	6.456E-5	5.998E-5	8.460E-5	7.610E-5	72.90	142.81	270.18	117.50	5.93	13.06	22.55	15.13	2	3	5	4	4	5	8	6
Q9JM53	Apoptosis-inducing factor 1, mitochondrial OS=Rattus norvegicus GN=Aifm1 PE=1 SV=1 - [AIFM1_RAT]	66.7	4.159E-5	5.694E-5	4.647E-5	5.511E-5	54.36	170.72	109.81	107.38	8.66	11.27	8.66	5.39	4	5	4	3	6	9	6	6
Q9JMB5	Proteasomal ubiquitin receptor ADRM1 OS=Rattus norvegicus GN=Adrm1 PE=2 SV=2 - [ADRM1_RAT]	42.1	0.000E0	3.595E-5	6.645E-5	3.463E-5		49.31	53.77	51.67		3.69	5.90	3.69		2	2	2		3	4	3
Q9JMI1	Acetoacetyl-CoA synthetase OS=Rattus norvegicus OX=10116 GN=Aacs PE=1 SV=1 - [AACS_RAT]	75.0	0.000E0	4.963E-5	0.000E0	4.052E-5		21.76		48.74		2.83		1.34		2		1		2		1
Q9JMJ4	Pre-mRNA-processing factor 19 OS=Rattus norvegicus GN=Prpf19 PE=1 SV=2 - [PRP19_RAT]	55.2	6.600E-4	4.418E-4	4.459E-4	3.234E-4	467.41	366.39	375.31	194.79	36.31	30.95	24.40	29.96	12	8	7	7	27	16	15	10
Q9QUL6	Vesicle-fusing ATPase OS=Rattus norvegicus GN=Nsf PE=1 SV=1 - [NSF_RAT]	82.6	2.954E-5	5.841E-5	5.236E-5	4.047E-5	26.06	87.67	85.52	31.78	2.96	4.57	5.11	4.84	2	3	3	3	2	5	4	3
Q9QVC8	Peptidyl-prolyl cis-trans isomerase FKBP4 OS=Rattus norvegicus GN=Fkbp4 PE=1 SV=3 - [FKBP4_RAT]	51.4	2.345E-4	3.218E-4	2.334E-4	2.097E-4	447.55	730.84	619.21	505.62	23.36	45.20	36.03	39.96	8	18	12	17	16	27	23	27
Q9QWN8	Spectrin beta chain, non-erythrocytic 2 OS=Rattus norvegicus GN=Sptbn2 PE=1 SV=2 - [SPTN2_RAT]	270.9	2.184E-4	1.885E-4	1.665E-4	5.718E-5	257.09	110.10	205.87	131.51	5.07	2.76	2.76	1.72	11	7	6	4	20	10	12	6
Q9QX69	LanC-like protein 1 OS=Rattus norvegicus GN=Lancl1 PE=2 SV=2 - [LANC1_RAT]	45.2	4.437E-3	9.638E-4	7.554E-4	8.821E-4	27.92	68.17	125.17	26.47	2.01	9.77	9.77	5.01	1	3	3	2	1	4	4	4
Q9QX79	Fetuin-B OS=Rattus norvegicus GN=Fetub PE=2 SV=2 - [FETUB_RAT]	41.5	2.331E-4	3.042E-4	3.248E-4	4.206E-4	134.36	485.63	573.07	386.30	30.16	44.71	48.41	36.77	7	10	12	10	10	24	27	25
Q9QXQ0	Alpha-actinin-4 OS=Rattus norvegicus GN=Actn4 PE=1 SV=2 - [ACTN4_RAT]	104.8	5.307E-3	3.795E-3	3.682E-3	2.222E-3	10048.91	8939.41	9632.90	6974.35	75.41	74.64	76.18	72.78	65	64	64	58	303	271	274	222
Q9QY02	YTH domain-containing protein 1 OS=Rattus norvegicus OX=10116 GN=Ythdc1 PE=1 SV=2 - [YTDC1_RAT]	85.8	0.000E0	0.000E0	0.000E0	1.934E-5				20.22				0.81				1				2
Q9QY17	Protein kinase C and casein kinase substrate in neurons 2 protein OS=Rattus norvegicus GN=Pacsin2 PE=1 SV=2 - [PACN	55.9	4.167E-4	0.000E0	0.000E0	4.577E-5	33.16			37.06	1.43			2.25	1			1	2			1
Q9QYW3	MOB-like protein phocein OS=Rattus norvegicus GN=Mob4 PE=1 SV=1 - [PHOCN_RAT]	26.0	6.468E-6	4.759E-5	6.175E-5	3.903E-5	24.24	43.04	107.54	37.85	4.00	10.22	16.44	16.44	1	2	3	3	1	4	5	4
Q9QZ81	Protein argonaute-2 OS=Rattus norvegicus GN=Ago2 PE=2 SV=2 - [AGO2_RAT]	97.3	0.000E0	3.188E-5	0.000E0	0.000E0		28.61				2.79				2				2		
Q9QZ86	Nucleolar protein 58 OS=Rattus norvegicus GN=Nop58 PE=1 SV=1 - [NOP58_RAT]	60.0	0.000E0	4.182E-5	4.925E-5	6.232E-5		44.25	90.03	65.19		4.31	6.18	7.30		2	3	3		3	5	4
Q9QZA2	Programmed cell death 6-interacting protein OS=Rattus norvegicus GN=Pdcd6ip PE=1 SV=2 - [PDC6I_RAT]	96.6	1.552E-4	2.500E-4	2.571E-4	1.951E-4	375.42	1150.17	1391.67	1105.06	14.09	28.52	37.34	30.58	10	19	23	21	17	39	48	39
Q9QZA6	CD151 antigen OS=Rattus norvegicus GN=Cd151 PE=1 SV=2 - [CD151_RAT]	28.3	2.964E-5	9.893E-6	0.000E0	0.000E0	25.35	26.56			3.95	3.95			1	1			2	1		
Q9QZR6	Septin-9 OS=Rattus norvegicus GN=Sept9 PE=1 SV=1 - [SEPT9_RAT]	63.8	0.000E0	0.000E0	3.522E-5	3.389E-5			86.23	32.17			6.38	1.77			3	1			4	1
Q9R063	Peroxiredoxin-5, mitochondrial OS=Rattus norvegicus GN=Prdx5 PE=1 SV=1 - [PRDX5_RAT]	22.2	3.568E-3	1.205E-3	1.856E-3	1.055E-3	1410.44	809.34	1071.20	675.28	55.40	54.93	55.40	55.40	12	11	12	12	51	35	39	25
Q9R064	Golgi reassembly-stacking protein 2 OS=Rattus norvegicus GN=Gorasp2 PE=1 SV=3 - [GORS2_RAT]	47.2	0.000E0	3.468E-5	4.348E-5	3.609E-5		81.48	117.89	94.68		7.27	8.81	8.81		2	3	3		3	5	4
Q9R085	Ubiquitin carboxyl-terminal hydrolase 15 OS=Rattus norvegicus GN=Usp15 PE=1 SV=1 - [UBP15_RAT]	109.2	4.919E-5	4.540E-5	4.005E-5	0.000E0	60.19	106.02	32.34		5.15	6.41	4.41		5	4	3		6	6	3	
Q9R0J8	Legumain OS=Rattus norvegicus GN=Lgmn PE=1 SV=1 - [LGMN_RAT]	49.4	0.000E0	0.000E0	1.282E-4	2.241E-4			101.04	133.80			13.10	13.10			3	3			8	12
Q9R0T3	DnaJ homolog subfamily C member 3 OS=Rattus norvegicus OX=10116 GN=Dnajc3 PE=1 SV=3 - [DNJC3_RAT]	57.5	4.306E-5	4.080E-5	3.749E-5	0.000E0	33.72	116.91	53.26		5.36	9.72	7.94		2	3	2		2	4	2	
Q9R0T4	Cadherin-1 OS=Rattus norvegicus GN=Cdh1 PE=1 SV=1 - [CADH1_RAT]	98.7	4.498E-4	3.216E-4	2.556E-4	1.046E-4	583.33	490.28	296.41	107.53	13.43	9.93	12.98	8.80	8	6	7	4	20	14	12	5
Q9R1J8	Prolyl 3-hydroxylase 1 OS=Rattus norvegicus GN=P3h1 PE=1 SV=1 - [P3H1_RAT]	82.3	1.708E-5	3.329E-5	3.188E-5	0.000E0	99.22	71.36	184.94		3.16	6.59	8.65		2	4	5		4	6	7	
Q9R1T3	Cathepsin Z OS=Rattus norvegicus OX=10116 GN=Ctsz PE=1 SV=2 - [CATZ_RAT]	34.2	7.519E-4	6.367E-4	6.995E-4	5.240E-4	433.46	353.83	373.66	328.29	33.66	26.47	34.97	30.39	8	7	7	7	19	14	16	15
Q9R1Z0	Voltage-dependent anion-selective channel protein 3 OS=Rattus norvegicus OX=10116 GN=Vdac3 PE=1 SV=2 - [VDAC3_I	30.8	6.932E-5	0.000E0	6.305E-5	5.025E-5	89.70		57.01	68.86	3.53		3.53	7.42	1		1	2	2		1	3
Q9WTT6	Guanine deaminase OS=Rattus norvegicus GN=Gda PE=1 SV=1 - [GUAD_RAT]	51.0	0.000E0	6.064E-4	6.965E-4	2.352E-4		1009.95	1046.61	544.72		49.34	54.19	40.53		17	20	14		35	38	23
Q9WU06	Advillin OS=Rattus norvegicus OX=10116 GN=Avil PE=2 SV=1 - [AVIL_RAT]	93.0	0.000E0	0.000E0	0.000E0	4.748E-5			60.18	102.40			1.09	1.09			1	1			1	2
Q9WU49	Calcium-regulated heat stable protein 1 OS=Rattus norvegicus GN=Carhsp1 PE=1 SV=1 - [CHSP1_RAT]	15.9	5.534E-5	0.000E0	0.000E0	0.000E0	37.63				10.88				1				2			
Q9WU82	Catenin beta-1 OS=Rattus norvegicus GN=Ctnnb1 PE=1 SV=1 - [CTNB1_RAT]	85.4	1.939E-4	2.708E-4	2.688E-4	1.512E-4	439.05	1043.00	991.63	534.06	20.10	32.39	33.29	22.66	13	17	17	11	22	37	33	19
Q9WUC4	Copper transport protein ATOX1 OS=Rattus norvegicus GN=Atox1 PE=1 SV=1 - [ATOX1_RAT]	7.3	0.000E0	2.756E-5	0.000E0	0.000E0		22.61				11.76				1				2		
Q9WUC8	Pleiotropic regulator 1 OS=Rattus norvegicus GN=Plrg1 PE=2 SV=1 - [PLRG1_RAT]	57.2	0.000E0	3.081E-5	3.629E-5	0.000E0		64.67	51.14			5.06	7.98			2	2			3	3	
Q9WUF4	Vesicle-associated membrane protein 8 OS=Rattus norvegicus OX=10116 GN=Vamp8 PE=1 SV=1 - [VAMP8_RAT]	11.3	5.643E-5	0.000E0	2.650E-5	0.000E0	40.06		24.66		19.00		19.00		2		2		4		3	
Q9WUH4	Four and a half LIM domains protein 1 OS=Rattus norvegicus GN=Fhl1 PE=2 SV=1 - [FHL1_RAT]	31.9	0.000E0	3.521E-4	3.307E-4	3.027E-4		560.44	450.78	400.82		55.36	47.50	46.79		12	11	10		24	21	16
Q9WUJ3	Myomegalin OS=Rattus norvegicus GN=Pde4dip PE=1 SV=1 - [MYOME_RAT]	261.9	0.000E0	0.000E0	0.000E0	1.878E-4			0.00	0.00			0.30	0.30			1	1			1	3
Q9WV25	Poly(U)-binding-splicing factor PUF60 OS=Rattus norvegicus GN=Puf60 PE=2 SV=2 - [PUF60_RAT]	60.2	2.289E-4	3.444E-4	2.656E-4	2.091E-4	317.25	405.28	469.38	255.45	11.17	21.28	19.50	15.60	5	9	8	7	12	20	18	14
Q9WVB1	Ras-related protein Rab-6A OS=Rattus norvegicus GN=Rab6a PE=2 SV=2 - [RAB6A_RAT]	23.6	3.903E-4	3.976E-4	5.583E-4	4.192E-4	244.75	152.27	145.39	65.44	37.50	26.44	42.31	19.23	8	6	8	4	14	13	14	7
Q9WVC0	Septin-7 OS=Rattus norvegicus GN=Sept7 PE=1 SV=1 - [SEPT7_RAT]	50.5	2.871E-4	1.275E-4	2.004E-4	1.219E-4	63.21	138.46	230.65	172.74	2.06	10.32	21.10	17.20	1	3	6	5	2	10	16	10
Q9WVH8	Fibulin-5 OS=Rattus norvegicus GN=Fbln5 PE=2 SV=1 - [FBLN5_RAT]	50.1	9.795E-5	4.474E-5	6.205E-5	5.827E-5	207.24	102.84	109.44	96.81	15.85	8.48	11.61	8.48	6	3	4	3	12	5	6	5
Q9WVK3	Peroxisomal trans-2-enoyl-CoA reductase OS=Rattus norvegicus OX=10116 GN=Pecr PE=2 SV=1 - [PECR_RAT]	32.4	0.000E0	8.075E-5	1.112E-4	1.208E-4		76.70	220.68	194.53		18.48	30.36	26.73		3	6	5		4	10	8
Q9WVK7	Hydroxyacyl-coenzyme A dehydrogenase, mitochondrial OS=Rattus norvegicus GN=Hadh PE=2 SV=1 - [HCDH_RAT]	34.4	4.389E-4	6.286E-4	8.613E-4	5.639E-4	653.56	1015.59	1329.83	897.34	47.77	72.61	67.20	67.52	11	15	13	13	25	43	45	36
Q9WVS2	Probable tRNA N6-adenosine threonylcarbamoyltransferase OS=Rattus norvegicus GN=Osgep PE=2 SV=2 - [OSGEP_RAT]	36.3	0.000E0	5.164E-5	5.403E-5	0.000E0		52.44	106.08			5.07	17.31			1	4			1	7	
Q9Z0J5	Thioredoxin reductase 2, mitochondrial OS=Rattus norvegicus GN=Txnrd2 PE=1 SV=3 - [TRXR2_RAT]	56.5	4.695E-4	0.000E0	1.866E-4	1.153E-4	172.95		64.53	84.55	5.13		2.85	2.85	2		1	1	3		1	2
Q9Z0T0	Thiopurine S-methyltransferase OS=Rattus norvegicus GN=Tpmt PE=2 SV=1 - [TPMT_RAT]	27.7	0.000E0	6.138E-5	4.518E-5	0.000E0		28.73	22.83			3.33	3.33			1	1			1	1	
Q9Z0V5	Peroxiredoxin-4 OS=Rattus norvegicus GN=Prdx4 PE=2 SV=1 - [PRDX4_RAT]	31.0	6.689E-3	3.738E-3	3.567E-3	2.698E-3	985.77	763.20	672.05	558.05	49.45	46.89	44.32	31.50	9	9	8	6	35	27	22	18
Q9Z0V6	Thioredoxin-dependent peroxide reductase, mitochondrial OS=Rattus norvegicus GN=Prdx3 PE=1 SV=2 - [PRDX3_RAT]	28.3	8.503E-4	6.014E-4	5.265E-4	4.664E-4	627.26	557.71	519.45	351.89	40.08	45.14	33.07	45.14	8	7	6	7	21	21	17	18
Q9Z0W7	Chloride intracellular channel protein 4 OS=Rattus norvegicus GN=Clic4 PE=1 SV=3 - [CLIC4_RAT]	28.6	0.000E0	2.806E-4	3.055E-4	2.580E-4		424.78	603.98	363.22		45.06	49.80	45.06		10	10	9		20	22	18
Q9Z118	Polypyrimidine tract-binding protein 3 OS=Rattus norvegicus GN=Ptbp3 PE=2 SV=1 - [PTBP3_RAT]	56.7	6.400E-5	2.630E-4	2.157E-4	2.341E-4	44.38	252.88	280.24	207.63	8.60	14.91	15.68	18.93	4	6	6	8	4	12	11	11
Q9Z144	Galectin-2 OS=Rattus norvegicus OX=10116 GN=Lgals2 PE=2 SV=1 - [LEG2_RAT]	14.7	1.429E-3	3.370E-3	2.518E-3	1.557E-3	1115.94	1949.28	1659.65	1165.63	82.31	82.31	82.31	82.31	9	9	9	9	35	51	43	36
Q9Z1A5	NEDD8-activating enzyme E1 regulatory subunit OS=Rattus norvegicus GN=Nae1 PE=1 SV=1 - [ULA1_RAT]	60.3	0.000E0	4.949E-5	6.454E-5	6.315E-5		145.94	222.56	127.78		10.30	21.16	15.36		3	6	5		5	9	7
Q9Z1A6	Vigilin OS=Rattus norvegicus GN=Hdlbp PE=1 SV=1 - [VIGLN_RAT]	141.5	1.102E-4	1.496E-4	1.635E-4	3.053E-4	198.09	295.84	398.48	366.65	4.42	7.81	10.17	13.25	4	8	10	11	8	14	18	20
Q9Z1B2	Glutathione S-transferase Mu 5 OS=Rattus norvegicus GN=Gstm5 PE=1 SV=3 - [GSTM5_RAT]	26.6	2.652E-4	2.891E-4	3.373E-4	3.466E-4	332.12	319.27	438.20	433.60	31.11	27.11	50.67	44.89	7	6	9	9	13	12	17	17
Q9Z1N1	Fructose-1,6-bisphosphatase isozyme 2 OS=Rattus norvegicus OX=10116 GN=Fbp2 PE=1 SV=1 - [F16P2_RAT]	36.9	0.000E0	3.897E-4	2.290E-4	3.524E-4		666.74	292.02	331.73		25.37	21.83	39.53		6	5	8		17	11	14
Q9Z1N4	3'(2'),5'-bisphosphate nucleotidase 1 OS=Rattus norvegicus GN=Bpnt1 PE=1 SV=1 - [BPNT1_RAT]	33.2	0.000E0	1.079E-4	1.128E-4	4.812E-5		233.25	188.72	101.40		25.00	28.57	16.23		5	7	3		9	11	5
Q9Z1P2	Alpha-actinin-1 OS=Rattus norvegicus GN=Actn1 PE=1 SV=1 - [ACTN1_RAT]	102.9	5.133E-3	3.601E-3	3.410E-3	2.026E-3	7514.65	7648.82	7919.01	5028.99	73.88	69.96	72.42	64.69	61	55	55	50	241	226	217	167
Q9Z1Z3	Epsin-2 OS=Rattus norvegicus GN=Epn2 PE=1 SV=1 - [EPN2_RAT]	62.3	5.400E-5	1.110E-5	0.000E0	0.000E0	21.64	31.51			1.72	1.72			1	1			2	1		
Q9Z1Z9	PDZ and LIM domain protein 7 OS=Rattus norvegicus OX=10116 GN=Pdlim7 PE=1 SV=1 - [PDLI7_RAT]	49.9	6.544E-5	1.075E-4	0.000E0	0.000E0	22.33	118.61			2.19	4.60			1	2			1	3		
Q9Z254	PDZ domain-containing protein GIPC1 OS=Rattus norvegicus GN=Gipc1 PE=1 SV=2 - [GIPC1_RAT]	36.1	7.760E-5	0.000E0	1.241E-4	7.756E-5	21.82		44.77	36.83	5.11		5.11	8.71	1		1	2	1		3	2
Q9Z270	Vesicle-associated membrane protein-associated protein A OS=Rattus norvegicus OX=10116 GN=Vapa PE=1 SV=3 - [VAP	27.8	0.000E0	9.348E-5	1.138E-4	4.085E-5		200.01	158.50	56.74		7.63	4.82	7.63		2	1	2		4	2	2
Q9Z2F5	C-terminal-binding protein 1 OS=Rattus norvegicus GN=Ctbp1 PE=1 SV=3 - [CTBP1_RAT]	46.6	0.000E0	1.260E-4	1.208E-4	8.956E-5		129.69	134.25	78.69		11.16	13.02	10.47		4	5	4		6	6	6
Q9Z2G8	Nucleosome assembly protein 1-like 1 OS=Rattus norvegicus GN=Nap1l1 PE=2 SV=1 - [NP1L1_RAT]	45.3	0.000E0	1.297E-4	1.054E-4	1.167E-4		80.26	105.28	78.00		2.82	10.26	7.18		1	3	2		2	6	5
Q9Z2L0	Voltage-dependent anion-selective channel protein 1 OS=Rattus norvegicus OX=10116 GN=Vdac1 PE=1 SV=4 - [VDAC1_I	30.7	5.040E-5	4.049E-5	7.237E-5	5.084E-5	206.29	133.87	297.51	250.44	12.37	8.13	23.32	16.25	3	2	5	4	6	4	8	7
Q9Z2Q1	Protein transport protein Sec31A OS=Rattus norvegicus GN=Sec31a PE=1 SV=2 - [SC31A_RAT]	135.2	7.663E-5	2.446E-4	1.387E-4	1.994E-4	112.65	279.75	383.25	196.87	2.72	8.33	14.17	9.45	3	10	13	11	5	18	21	18
Q9Z330	DNA (cytosine-5)-methyltransferase 1 OS=Rattus norvegicus GN=Dnmt1 PE=1 SV=2 - [DNMT1_RAT]	182.7	0.000E0	0.000E0	0.000E0	2.904E-5				34.30				1.36				2				2
Q9Z339	Glutathione S-transferase omega-1 OS=Rattus norvegicus GN=Gsto1 PE=1 SV=2 - [GSTO1_RAT]	27.7	9.887E-5	1.080E-3	1.319E-3	1.063E-3	69.63	875.77	1137.99	695.64	19.50	67.63	68.46	60.17	4	13	13	13	8	38	48	33
