# Supplementary material for: Dedifferentiated fat cells administration ameliorates abnormal expressions of fatty acids metabolism-related protein expressions and intestinal tissue damage in experimental necrotizing enterocolitis
Source: Sci Rep. 2023 May 22;13:8266. doi: 10.1038/s41598-023-34156-1 (PMC10203254; doi:10.1038/s41598-023-34156-1)
Supplement: Supplementary file 4 — Supplementary Table S2. [file 41598_2023_34156_MOESM4_ESM.pdf]

Table S2. Dysregulated by NEC

Accession	Description	MW [kDa]	Area				Score				Coverage				# Peptides				# PSM			
			sham	vehicle -mild	vehicle -severe	DFAT	sham	vehicle -mild	vehicle -severe	DFAT	sham	vehicle -mild	vehicle -severe	DFAT	sham	vehicle -mild	vehicle -severe	DFAT	sham	vehicle -mild	vehicle -severe	DFAT
A0JPJ7	Obg-like ATPase 1 OS=Rattus norvegicus GN=Ola1 PE=2 SV=1 - [OLA1_RAT]	44.5	0.000E0	0.000E0	3.420E7	9.558E6			314.45	65.09			25.76	17.42			8	5			15	9
A2RUV9	Adipocyte enhancer-binding protein 1 OS=Rattus norvegicus GN=Aebp1 PE=2 SV=1 - [AEBP1_RAT]	128.0	5.074E6	0.000E0	0.000E0	0.000E0	53.72				1.24				1				2			
A2RUW1	Toll-interacting protein OS=Rattus norvegicus GN=Tollip PE=2 SV=1 - [TOLIP_RAT]	30.3	0.000E0	8.855E6	1.771E7	3.698E6		99.97	57.12	28.77		8.03	4.74	13.14		2	1	3		3	2	4
B0BN93	26S proteasome non-ATPase regulatory subunit 13 OS=Rattus norvegicus GN=Psmd13 PE=1 SV=1 - [PSD13_RAT]	42.8	5.354E6	2.042E7	3.414E7	1.888E7	84.87	157.69	688.14	281.06	11.44	25.53	60.11	46.28	4	10	19	15	5	15	34	24
B0BNA7	Eukaryotic translation initiation factor 3 subunit I OS=Rattus norvegicus GN=Eif3i PE=2 SV=1 - [EIF3I_RAT]	36.4	3.856E6	1.613E7	2.617E7	6.826E6	29.94	195.07	214.23	103.70	5.54	27.38	21.54	8.31	2	6	5	3	2	8	8	5
B0BNM1	NAD(P)H-hydrate epimerase OS=Rattus norvegicus GN=Apoa1bp PE=2 SV=1 - [NNRE_RAT]	30.9	0.000E0	7.467E6	1.070E7	9.354E6		37.40	73.88	45.48		20.57	14.54	17.38		3	2	3		4	4	4
B0K020	CDGSH iron-sulfur domain-containing protein 1 OS=Rattus norvegicus OX=10116 GN=Cisd1 PE=3 SV=1 - [CISD1_RAT]	12.1	7.571E6	0.000E0	0.000E0	0.000E0	38.52				12.04				1				1			
B1H267	Sorting nexin-5 OS=Rattus norvegicus GN=Snx5 PE=1 SV=1 - [SNX5_RAT]	46.8	2.453E7	1.520E7	8.685E6	0.000E0	258.29	57.20	36.00		11.14	3.71	3.71		4	1	1		11	2	1	
B2GUZ5	F-actin-capping protein subunit alpha-1 OS=Rattus norvegicus GN=Capza1 PE=1 SV=1 - [CAZA1_RAT]	32.9	1.730E6	7.608E7	1.213E8	4.336E7	27.30	571.07	625.85	389.43	2.80	64.69	64.69	54.20	1	12	12	11	1	24	27	18
B2GV06	Succinyl-CoA:3-ketoacid coenzyme A transferase 1, mitochondrial OS=Rattus norvegicus GN=Oxct1 PE=1 SV=1 - [SCOT1_	56.2	0.000E0	1.341E7	2.364E7	8.697E6		118.06	380.67	105.86		26.15	31.54	13.27		7	9	4		8	16	6
B2RYG6	Ubiquitin thioesterase OTUB1 OS=Rattus norvegicus GN=Otub1 PE=1 SV=1 - [OTUB1_RAT]	31.3	0.000E0	3.394E7	4.855E7	2.476E7		369.86	512.18	459.93		35.06	38.01	31.37		8	9	8		18	18	18
B5DFC8	Eukaryotic translation initiation factor 3 subunit C OS=Rattus norvegicus GN=Eif3c PE=1 SV=1 - [EIF3C_RAT]	105.4	0.000E0	1.956E7	2.941E7	1.841E7		41.34	432.81	250.78		8.23	13.61	9.77		6	11	8		10	19	13
D3ZD32	Chromodomain-helicase-DNA-binding protein 5 OS=Rattus norvegicus GN=Chd5 PE=1 SV=1 - [CHD5_RAT]	222.1	0.000E0	7.418E6	1.251E7	5.267E6		51.21	80.16	50.59		1.59	2.05	2.52		3	3	4		3	3	4
D3ZDK7	Glycerol-3-phosphate phosphatase OS=Rattus norvegicus OX=10116 GN=Pgp PE=1 SV=1 - [PGP_RAT]	34.6	0.000E0	5.336E6	9.096E6	3.891E6		43.33	155.01	25.24		9.03	16.82	11.84		2	4	3		3	6	3
D3ZZ80	Obscurin-like protein 1 OS=Rattus norvegicus GN=Obsl1 PE=2 SV=3 - [OBSL1_RAT]	197.7	5.051E6	1.926E7	3.151E7	1.317E7	36.92	0.00	0.00	0.00	2.22	0.44	0.44	0.44	2	1	1	1	3	2	2	1
E9PU28	Inosine-5'-monophosphate dehydrogenase 2 OS=Rattus norvegicus GN=Impdh2 PE=3 SV=1 - [IMDH2_RAT]	55.8	0.000E0	0.000E0	3.340E7	2.271E7			44.27	33.52			5.45	9.14			3	4			5	6
F1LMZ8	26S proteasome non-ATPase regulatory subunit 11 OS=Rattus norvegicus GN=Psmd11 PE=3 SV=2 - [PSD11_RAT]	47.4	0.000E0	6.976E6	2.015E7	9.049E6		43.59	353.27	111.55		17.06	36.97	21.09		6	13	7		8	19	10
F1LN46	Carnitine O-palmitoyltransferase 1, brain isoform OS=Rattus norvegicus OX=10116 GN=Cpt1c PE=1 SV=1 - [CPT1C_RAT]	90.1	4.642E6	0.000E0	0.000E0	0.000E0	26.00				0.87				1				2			
F1LNI5	Protein phosphatase 1G OS=Rattus norvegicus GN=Ppm1g PE=1 SV=2 - [PPM1G_RAT]	58.7	0.000E0	7.169E7	1.215E8	5.095E7		39.65	26.17	52.53		5.54	4.06	5.35		3	2	3		5	3	5
G3V6S8	Serine/arginine-rich splicing factor 6 OS=Rattus norvegicus OX=10116 GN=Srsf6 PE=1 SV=1 - [SRSF6_RAT]	39.0	0.000E0	1.622E7	7.065E7	6.129E7		53.36	336.47	387.76		2.65	19.17	25.07		1	7	9		1	12	17
G3V7P1	Syntaxin-12 OS=Rattus norvegicus GN=Stx12 PE=1 SV=1 - [STX12_RAT]	31.2	1.464E7	0.000E0	0.000E0	0.000E0	141.74				26.64				5				9			
M0RC99	Ras-related protein Rab-5A OS=Rattus norvegicus GN=Rab5a PE=2 SV=1 - [RAB5A_RAT]	23.6	6.858E7	3.515E7	2.256E7	2.133E7	173.16	72.70	76.08	80.49	10.23	5.12	18.60	5.12	2	1	3	1	4	2	4	2
O08587	Nuclear pore complex protein Nup50 OS=Rattus norvegicus GN=Nup50 PE=2 SV=2 - [NUP50_RAT]	49.8	1.025E7	0.000E0	0.000E0	0.000E0	20.90				1.50				1				2			
O08629	Transcription intermediary factor 1-beta OS=Rattus norvegicus GN=Trim28 PE=1 SV=2 - [TIF1B_RAT]	88.9	7.626E6	3.289E7	5.066E7	3.247E7	42.91	326.26	364.54	368.19	2.40	10.90	11.74	14.25	1	7	8	9	2	11	14	15
O08651	D-3-phosphoglycerate dehydrogenase OS=Rattus norvegicus GN=Phgdh PE=1 SV=3 - [SERA_RAT]	56.5	0.000E0	0.000E0	3.757E6	2.976E6		22.76	29.93	59.74		2.44	3.94	3.19		1	2	2		1	2	4
O08699	15-hydroxyprostaglandin dehydrogenase [NAD(+)] OS=Rattus norvegicus OX=10116 GN=Hpgd PE=2 SV=2 - [PGDH_RAT	28.9	0.000E0	6.275E6	8.804E6	5.725E6		88.11	58.57	53.12		9.02	9.40	9.40		2	2	2		3	3	2
O09175	Aminopeptidase B OS=Rattus norvegicus GN=Rnpep PE=1 SV=2 - [AMPB_RAT]	72.6	0.000E0	2.206E7	3.564E7	1.792E7		200.57	433.32	232.61		16.15	28.00	29.23		7	12	14		13	21	22
O35077	Glycerol-3-phosphate dehydrogenase [NAD(+)], cytoplasmic OS=Rattus norvegicus GN=Gpd1 PE=1 SV=4 - [GPDA_RAT]	37.4	5.544E6	2.651E7	3.772E7	1.522E7	61.86	237.64	350.99	87.64	3.15	37.82	39.83	29.23	1	10	9	6	1	16	16	9
O35078	D-amino-acid oxidase OS=Rattus norvegicus GN=Dao PE=2 SV=1 - [OXDA_RAT]	38.8	2.499E7	0.000E0	0.000E0	0.000E0	194.99				18.21				5				9			
O35244	Peroxiredoxin-6 OS=Rattus norvegicus GN=Prdx6 PE=1 SV=3 - [PRDX6_RAT]	24.8	6.652E6	6.069E7	9.945E7	3.323E7	120.11	593.29	857.12	466.76	26.34	62.50	70.09	68.75	4	11	12	12	5	22	29	23
O35783	Calumenin OS=Rattus norvegicus OX=10116 GN=Calu PE=1 SV=1 - [CALU_RAT]	37.0	0.000E0	0.000E0	4.531E6	1.034E7			21.69	117.52			4.13	13.33			1	3			1	5
O54975	Xaa-Pro aminopeptidase 1 OS=Rattus norvegicus GN=Xpnpep1 PE=1 SV=1 - [XPP1_RAT]	69.6	1.582E7	5.180E7	7.810E7	3.314E7	186.05	983.60	1323.85	703.27	12.52	65.01	65.65	48.15	7	25	26	22	13	48	62	37
O55012	Phosphatidylinositol-binding clathrin assembly protein OS=Rattus norvegicus GN=Picalm PE=1 SV=1 - [PICAL_RAT]	69.2	0.000E0	0.000E0	3.803E6	4.265E6		24.52	119.29	44.87		1.41	3.28	3.28		1	2	2		1	3	2
O55159	Epithelial cell adhesion molecule OS=Rattus norvegicus OX=10116 GN=Epcam PE=1 SV=1 - [EPCAM_RAT]	35.2	2.766E6	3.325E7	4.666E7	1.209E7	27.95	204.93	455.20	65.21	4.13	40.63	40.63	25.08	1	7	8	5	2	12	16	7
O55165	Kinesin-like protein KIF3C OS=Rattus norvegicus OX=10116 GN=Kif3c PE=2 SV=1 - [KIF3C_RAT]	89.8	0.000E0	2.633E7	4.116E7	1.864E7		25.18	21.86	22.70		2.01	2.01	1.13		2	2	1		4	4	1
O70196	Prolyl endopeptidase OS=Rattus norvegicus GN=Prep PE=1 SV=1 - [PPCE_RAT]	80.7	0.000E0	1.382E7	2.263E7	1.308E7		336.55	459.83	419.04		14.37	25.63	22.82		8	14	13		14	21	27
O70199	UDP-glucose 6-dehydrogenase OS=Rattus norvegicus GN=Ugdh PE=1 SV=1 - [UGDH_RAT]	54.9	0.000E0	1.224E7	3.104E7	2.645E7		272.20	830.72	733.74		17.24	33.87	41.18		6	13	15		10	27	30
O70351	3-hydroxyacyl-CoA dehydrogenase type-2 OS=Rattus norvegicus GN=Hsd17b10 PE=1 SV=3 - [HCD2_RAT]	27.2	0.000E0	0.000E0	1.276E7	5.302E6			193.56	92.27			32.95	18.01			6	3			10	4
O70352	CD82 antigen OS=Rattus norvegicus OX=10116 GN=Cd82 PE=1 SV=1 - [CD82_RAT]	29.5	9.440E6	0.000E0	0.000E0	0.000E0	111.99				12.03				3				5			
O70377	Synaptosomal-associated protein 23 OS=Rattus norvegicus OX=10116 GN=Snap23 PE=1 SV=1 - [SNP23_RAT]	23.2	3.887E6	0.000E0	0.000E0	0.000E0	34.04				6.67				1				1			
O70531	Sulfate transporter OS=Rattus norvegicus OX=10116 GN=Slc26a2 PE=2 SV=1 - [S26A2_RAT]	82.0	9.298E5	0.000E0	0.000E0	0.000E0	37.20				1.22				1				1			
O88202	60 kDa lysophospholipase OS=Rattus norvegicus OX=10116 GN=Aspg PE=1 SV=1 - [LPP60_RAT]	60.8	0.000E0	4.497E6	6.075E6	0.000E0		24.79	38.25			4.08	4.08			2	2			2	3	
O88267	Acyl-coenzyme A thioesterase 1 OS=Rattus norvegicus GN=Acot1 PE=1 SV=1 - [ACOT1_RAT]	46.0	8.508E6	3.068E7	4.612E7	2.294E7	67.30	351.64	441.52	436.85	2.63	14.56	28.64	17.66	1	5	8	6	2	10	16	15
O88656	Actin-related protein 2/3 complex subunit 1B OS=Rattus norvegicus GN=Arpc1b PE=2 SV=3 - [ARC1B_RAT]	41.0	5.408E6	2.785E7	3.938E7	1.795E7	67.72	322.41	508.41	434.56	9.14	30.38	36.29	30.65	3	8	11	9	4	12	20	15
O88761	26S proteasome non-ATPase regulatory subunit 1 OS=Rattus norvegicus GN=Psmd1 PE=2 SV=1 - [PSMD1_RAT]	105.7	0.000E0	9.193E6	2.580E7	1.215E7		130.27	473.35	229.13		14.27	24.55	10.28		9	14	7		11	24	11
O88767	Protein DJ-1 OS=Rattus norvegicus GN=Park7 PE=1 SV=1 - [PARK7_RAT]	20.0	0.000E0	3.413E7	8.530E7	4.241E7		447.79	899.65	515.87		68.78	69.31	69.31		8	9	9		15	24	21
O89046	Coronin-1B OS=Rattus norvegicus GN=Coro1b PE=1 SV=1 - [COR1B_RAT]	53.8	8.155E6	3.246E7	6.136E7	3.191E7	33.46	271.19	438.75	332.15	2.27	8.47	27.89	15.29	1	4	8	6	2	7	13	12
P00406	Cytochrome c oxidase subunit 2 OS=Rattus norvegicus OX=10116 GN=Mtco2 PE=1 SV=3 - [COX2_RAT]	25.9	3.644E6	1.204E7	5.046E7	3.586E6	46.82	55.87	65.80	69.73	4.41	7.49	7.49	4.41	1	2	2	1	2	3	3	2
P00763	Anionic trypsin-2 OS=Rattus norvegicus OX=10116 GN=Prss2 PE=1 SV=2 - [TRY2_RAT]	26.2	3.815E7	2.310E7	1.073E7	4.248E6	385.16	74.93	62.42	67.98	31.71	4.07	19.51	19.51	4	1	2	2	12	2	3	4
P00787	Cathepsin B OS=Rattus norvegicus GN=Ctsb PE=1 SV=2 - [CATB_RAT]	37.4	3.179E7	1.222E8	1.994E8	1.074E8	308.09	1215.99	2106.06	1572.01	22.71	41.59	41.59	41.59	5	12	12	12	11	35	49	43
P01174	Whey acidic protein OS=Rattus norvegicus OX=10116 GN=Wap PE=1 SV=2 - [WAP_RAT]	14.8	1.888E8	0.000E0	0.000E0	0.000E0	152.04				10.95				1				5			
P02761	Major urinary protein OS=Rattus norvegicus OX=10116 PE=1 SV=1 - [MUP_RAT]	20.7	1.415E7	0.000E0	0.000E0	0.000E0	76.53				13.81				3				5			
P04550	Parathymosin OS=Rattus norvegicus OX=10116 GN=Ptms PE=1 SV=2 - [PTMS_RAT]	11.6	0.000E0	0.000E0	4.411E6	2.189E6		69.58	86.83	44.05		20.59	20.59	11.76		2	2	1		3	2	1
P04642	L-lactate dehydrogenase A chain OS=Rattus norvegicus GN=Ldha PE=1 SV=1 - [LDHA_RAT]	36.4	5.316E7	3.768E8	5.598E8	2.790E8	519.00	1928.76	2534.48	1971.66	32.23	78.61	78.61	72.59	9	23	23	22	20	73	97	75
P04644	40S ribosomal protein S17 OS=Rattus norvegicus GN=Rps17 PE=1 SV=3 - [RS17_RAT]	15.5	0.000E0	1.149E7	2.226E7	1.549E7		29.39	100.38	126.96		8.15	24.44	24.44		1	2	2		2	3	4
P04937	Fibronectin OS=Rattus norvegicus GN=Fn1 PE=1 SV=2 - [FINC_RAT]	272.3	4.310E7	1.097E8	1.554E8	9.910E7	829.52	1109.57	740.35	863.51	17.32	20.87	17.00	20.10	31	32	28	32	49	59	44	56
P04961	Proliferating cell nuclear antigen OS=Rattus norvegicus GN=Pcna PE=1 SV=1 - [PCNA_RAT]	28.7	0.000E0	7.435E6	2.845E7	1.441E7		84.22	97.85	117.57		8.43	8.43	18.77		2	2	4		3	5	7
P05197	Elongation factor 2 OS=Rattus norvegicus GN=Eef2 PE=1 SV=4 - [EF2_RAT]	95.2	6.863E7	2.299E8	3.083E8	1.234E8	1519.58	4050.46	4963.99	3331.46	37.53	63.40	60.49	65.85	28	43	42	43	65	162	175	140
P05369	Farnesyl pyrophosphate synthase OS=Rattus norvegicus GN=Fdps PE=2 SV=2 - [FPPS_RAT]	40.8	1.786E6	4.836E7	8.537E7	5.271E7	25.42	1149.07	1332.94	1154.84	2.55	41.64	49.58	41.64	1	15	15	14	1	39	41	40
P05712	Ras-related protein Rab-2A OS=Rattus norvegicus GN=Rab2a PE=1 SV=1 - [RAB2A_RAT]	23.5	5.357E6	1.093E7	2.660E7	1.074E7	20.25	277.75	264.65	215.61	6.60	26.89	38.21	30.66	1	4	6	5	1	7	9	8
P07150	Annexin A1 OS=Rattus norvegicus GN=Anxa1 PE=1 SV=2 - [ANXA1_RAT]	38.8	4.108E6	1.274E7	1.775E7	5.232E6	49.94	209.87	264.55	95.61	5.49	21.10	25.14	12.43	2	6	7	3	2	8	10	4
P07340	Sodium/potassium-transporting ATPase subunit beta-1 OS=Rattus norvegicus OX=10116 GN=Atp1b1 PE=1 SV=1 - [AT1B	35.2	1.287E7	3.077E7	4.815E7	1.694E7	59.76	95.56	161.23	103.06	7.24	10.53	8.88	12.50	2	3	2	3	4	9	6	5
P07633	Propionyl-CoA carboxylase beta chain, mitochondrial OS=Rattus norvegicus OX=10116 GN=Pccb PE=2 SV=1 - [PCCB_RAT	58.6	0.000E0	2.286E6	4.887E6	3.406E6		20.49	65.74	33.15		2.77	9.24	5.55		1	3	2		1	4	3
P07756	Carbamoyl-phosphate synthase [ammonia], mitochondrial OS=Rattus norvegicus GN=Cps1 PE=1 SV=1 - [CPSM_RAT]	164.5	2.873E7	7.449E7	1.375E8	3.588E7	707.98	2172.05	3783.71	1449.66	16.73	46.47	48.60	31.93	20	48	53	36	39	105	131	71
P07824	Arginase-1 OS=Rattus norvegicus GN=Arg1 PE=1 SV=2 - [ARGI1_RAT]	35.0	2.561E6	0.000E0	0.000E0	0.000E0	30.16				2.17				1				1			
P07871	3-ketoacyl-CoA thiolase B, peroxisomal OS=Rattus norvegicus GN=Acaa1b PE=1 SV=2 - [THIKB_RAT]	43.8	0.000E0	1.386E7	2.732E7	1.027E7		72.00	171.28	53.96		14.62	19.81	14.39		4	6	4		6	13	8
P07872	Peroxisomal acyl-coenzyme A oxidase 1 OS=Rattus norvegicus GN=Acox1 PE=1 SV=1 - [ACOX1_RAT]	74.6	0.000E0	3.161E7	4.728E7	2.003E7		731.25	1012.73	669.79		33.89	42.36	37.37		13	17	14		26	32	28
P07943	Aldose reductase OS=Rattus norvegicus GN=Akr1b1 PE=1 SV=3 - [ALDR_RAT]	35.8	1.095E7	6.135E7	8.552E7	3.422E7	164.08	567.45	578.72	382.95	12.66	54.75	57.91	39.87	4	15	15	12	8	34	36	25
P08011	Microsomal glutathione S-transferase 1 OS=Rattus norvegicus OX=10116 GN=Mgst1 PE=1 SV=3 - [MGST1_RAT]	17.5	0.000E0	8.359E6	1.125E7	5.683E6		87.44	79.74	47.49		8.39	8.39	8.39		1	1	1		2	2	2
P08461	Dihydrolipoyllysine-residue acetyltransferase component of pyruvate dehydrogenase complex, mitochondrial OS=Rattus nc	67.1	6.236E6	0.000E0	0.000E0	0.000E0	54.09				4.75				2				3			
P08503	Medium-chain specific acyl-CoA dehydrogenase, mitochondrial OS=Rattus norvegicus GN=Acadm PE=1 SV=1 - [ACADM_R	46.5	0.000E0	3.921E7	6.430E7	1.996E7		391.39	611.29	326.66		33.02	43.47	23.04		10	14	8		16	31	17
P08592	Amyloid beta A4 protein OS=Rattus norvegicus GN=App PE=1 SV=2 - [A4_RAT]	86.6	3.654E6	0.000E0	0.000E0	0.000E0	61.00				1.56				1				2			
P09456	cAMP-dependent protein kinase type I-alpha regulatory subunit OS=Rattus norvegicus GN=Prkar1a PE=2 SV=2 - [KAP0_R	43.1	0.000E0	2.116E7	2.982E7	1.399E7		325.22	456.68	383.57		21.78	26.51	21.78		7	8	7		11	15	13
P09811	Glycogen phosphorylase, liver form OS=Rattus norvegicus GN=Pygl PE=1 SV=5 - [PYGL_RAT]	97.4	5.839E6	1.318E7	2.852E7	1.885E7	20.60	175.75	405.79	381.29	1.41	6.82	13.53	15.53	1	5	9	10	2	8	15	17
P0C219	Sarcolemmal membrane-associated protein OS=Rattus norvegicus GN=Slmap PE=3 SV=1 - [SLMAP_RAT]	98.2	1.140E7	0.000E0	0.000E0	0.000E0	20.93				1.28				1				1			
P10610	Cytochrome P450 2G1 OS=Rattus norvegicus OX=10116 GN=Cyp2g1 PE=2 SV=1 - [CP2G1_RAT]	56.7	3.710E7	0.000E0	0.000E0	0.000E0	20.98				1.42				1				1			
P10719	ATP synthase subunit beta, mitochondrial OS=Rattus norvegicus GN=Atp5b PE=1 SV=2 - [ATPB_RAT]	56.3	0.000E0	1.524E7	6.177E7	7.337E7		271.94	1119.26	1635.19		15.31	42.16	54.06		6	15	19		9	38	59
P10760	Adenosylhomocysteinase OS=Rattus norvegicus GN=Ahcy PE=1 SV=3 - [SAHH_RAT]	47.5	3.716E6	8.393E7	1.270E8	6.507E7	108.47	963.02	1188.65	693.10	3.94	58.33	66.90	52.08	2	18	20	16	4	39	48	41
P10860	Glutamate dehydrogenase 1, mitochondrial OS=Rattus norvegicus GN=Glud1 PE=1 SV=2 - [DHE3_RAT]	61.4	0.000E0	1.096E8	1.909E8	7.576E7		1192.06	1624.72	1072.66		45.16	51.97	44.98		19	22	21		43	60	45
P11442	Clathrin heavy chain 1 OS=Rattus norvegicus GN=Cltc PE=1 SV=3 - [CLH1_RAT]	191.5	7.008E7	1.677E8	2.322E8	8.019E7	1951.00	5014.57	6902.68	3227.52	32.66	54.57	63.34	47.46	41	75	85	58	82	196	249	122
P11762	Galectin-1 OS=Rattus norvegicus GN=Lgals1 PE=1 SV=2 - [LEG1_RAT]	14.8	0.000E0	6.073E7	9.853E7	3.772E7		337.98	761.56	397.23		58.52	65.93	52.59		8	8	7		16	22	16
P11980	Pyruvate kinase PKM OS=Rattus norvegicus GN=Pkm PE=1 SV=3 - [KPYM_RAT]	57.8	5.239E6	8.542E7	1.437E8	6.204E7	134.66	1310.17	2244.80	1369.74	6.03	50.28	60.26	57.82	3	21	27	24	5	46	74	54
P12007	Isovaleryl-CoA dehydrogenase, mitochondrial OS=Rattus norvegicus GN=Ivd PE=1 SV=2 - [IVD_RAT]	46.4	1.844E6	1.524E7	2.315E7	5.696E6	26.41	206.16	325.33	130.86	5.19	13.21	19.81	10.61	2	5	7	4	2	8	11	6
P12336	Solute carrier family 2, facilitated glucose transporter member 2 OS=Rattus norvegicus OX=10116 GN=Slc2a2 PE=2 SV=1	57.0	4.339E6	0.000E0	0.000E0	0.000E0	29.33				2.30				1				1			
P12749	60S ribosomal protein L26 OS=Rattus norvegicus GN=Rpl26 PE=1 SV=1 - [RL26_RAT]	17.3	0.000E0	1.728E7	3.036E7	3.029E7		25.55	68.59	110.61		4.83	10.34	22.76		1	2	4		2	4	6
P12785	Fatty acid synthase OS=Rattus norvegicus GN=Fasn PE=1 SV=3 - [FAS_RAT]	272.5	0.000E0	2.260E7	6.810E7	3.466E7		427.65	970.44	368.47		8.30	21.72	9.54		16	38	18		24	59	27
P12788	Trypsin-4 OS=Rattus norvegicus OX=10116 GN=Try4 PE=2 SV=1 - [TRY4_RAT]	26.6	5.647E6	0.000E0	0.000E0	0.000E0	28.82				11.34				2				2			
P13086	Succinyl-CoA ligase [ADP/GDP-forming] subunit alpha, mitochondrial OS=Rattus norvegicus GN=Suclg1 PE=2 SV=2 - [SUC	36.1	0.000E0	2.294E7	3.373E7	1.579E7		166.70	189.31	138.27		17.05	17.05	17.05		4	4	4		9	8	8
P13264	Glutaminase kidney isoform, mitochondrial OS=Rattus norvegicus GN=Gls PE=1 SV=2 - [GLSK_RAT]	74.0	0.000E0	0.000E0	6.206E6	0.000E0		25.27	125.71			2.52	12.61			1	5			1	7	
P13437	3-ketoacyl-CoA thiolase, mitochondrial OS=Rattus norvegicus OX=10116 GN=Acaa2 PE=2 SV=1 - [THIM_RAT]	41.8	5.360E7	1.220E8	2.076E8	8.533E7	783.54	1413.81	1922.76	1237.28	57.93	65.74	65.49	63.48	16	20	19	16	29	53	66	48
P13471	40S ribosomal protein S14 OS=Rattus norvegicus GN=Rps14 PE=2 SV=3 - [RS14_RAT]	16.2	0.000E0	1.184E7	4.171E7	3.032E7		53.22	224.36	242.57		8.61	29.80	31.13		1	3	4		2	5	8
P13676	Acylamino-acid-releasing enzyme OS=Rattus norvegicus GN=Apeh PE=1 SV=1 - [ACPH_RAT]	81.3	0.000E0	2.578E7	3.573E7	1.445E7		366.84	511.19	330.51		21.31	44.54	18.31		10	17	8		15	24	15
P13697	NADP-dependent malic enzyme OS=Rattus norvegicus GN=Me1 PE=1 SV=2 - [MAOX_RAT]	64.0	0.000E0	1.825E7	2.676E7	1.159E7		365.93	573.93	143.19		33.04	37.24	24.13		13	14	8		21	25	11
P13803	Electron transfer flavoprotein subunit alpha, mitochondrial OS=Rattus norvegicus GN=Etfa PE=1 SV=4 - [ETFA_RAT]	34.9	0.000E0	9.225E7	1.319E8	5.879E7		729.80	1019.41	519.76		53.45	64.26	48.05		12	15	11		28	39	26
P14173	Aromatic-L-amino-acid decarboxylase OS=Rattus norvegicus OX=10116 GN=Ddc PE=1 SV=1 - [DDC_RAT]	54.0	0.000E0	0.000E0	3.408E6	0.000E0		20.83	55.74			1.46	5.42			1	2			1	3	
P14604	Enoyl-CoA hydratase, mitochondrial OS=Rattus norvegicus GN=Echs1 PE=1 SV=1 - [ECHM_RAT]	31.5	0.000E0	2.271E7	3.960E7	1.212E7		243.32	469.62	85.86		27.93	31.03	14.83		6	7	3		13	22	4
P14740	Dipeptidyl peptidase 4 OS=Rattus norvegicus GN=Dpp4 PE=1 SV=2 - [DPP4_RAT]	88.0	2.897E7	8.233E7	1.100E8	6.220E7	530.02	1096.35	1253.51	1134.38	20.08	34.16	45.37	32.72	15	23	30	24	27	46	63	53
P14841	Cystatin-C OS=Rattus norvegicus GN=Cst3 PE=1 SV=2 - [CYTC_RAT]	15.4	4.704E6	0.000E0	0.000E0	0.000E0	105.95				20.00				2				4			
P15083	Polymeric immunoglobulin receptor OS=Rattus norvegicus GN=Pigr PE=1 SV=1 - [PIGR_RAT]	84.7	7.048E7	0.000E0	0.000E0	0.000E0	1133.67				20.03				14				39			
P15178	Aspartate--tRNA ligase, cytoplasmic OS=Rattus norvegicus GN=Dars PE=2 SV=1 - [SYDC_RAT]	57.1	2.207E6	1.282E7	2.241E7	1.226E7	26.44	337.23	454.27	367.27	3.99	20.56	30.54	34.73	2	9	13	14	2	15	20	22
P15205	Microtubule-associated protein 1B OS=Rattus norvegicus OX=10116 GN=Map1b PE=1 SV=3 - [MAP1B_RAT]	269.5	3.492E5	0.000E0	0.000E0	1.665E7	0.00			22.10	0.33			0.33	1			1	1			1
P15800	Laminin subunit beta-2 OS=Rattus norvegicus GN=Lamb2 PE=2 SV=1 - [LAMB2_RAT]	196.3	6.900E6	0.000E0	0.000E0	0.000E0	25.28				0.39				1				1			
P15999	ATP synthase subunit alpha, mitochondrial OS=Rattus norvegicus GN=Atp5a1 PE=1 SV=2 - [ATPA_RAT]	59.7	0.000E0	2.177E7	4.690E7	6.253E7		307.25	494.47	844.78		14.47	23.15	37.25		8	11	16		14	23	34
P16617	Phosphoglycerate kinase 1 OS=Rattus norvegicus GN=Pgk1 PE=1 SV=2 - [PGK1_RAT]	44.5	1.087E7	3.173E8	4.376E8	2.460E8	70.08	2263.88	2626.18	1655.97	14.39	70.26	69.30	71.46	4	25	24	24	7	82	84	72
P16638	ATP-citrate synthase OS=Rattus norvegicus GN=Acly PE=1 SV=1 - [ACLY_RAT]	120.6	0.000E0	2.080E7	1.103E8	7.788E7		466.21	1017.11	582.96		13.91	19.36	17.18		13	18	15		25	41	31
P17074	40S ribosomal protein S19 OS=Rattus norvegicus GN=Rps19 PE=2 SV=3 - [RS19_RAT]	16.1	0.000E0	6.913E6	3.534E7	2.745E7		242.94	428.58	464.22		28.97	37.93	44.83		4	7	8		8	13	15
P17077	60S ribosomal protein L9 OS=Rattus norvegicus GN=Rpl9 PE=1 SV=1 - [RL9_RAT]	21.9	0.000E0	4.302E7	7.938E7	4.331E7		452.81	744.19	502.63		60.42	60.94	60.42		8	10	8		18	26	22
P17078	60S ribosomal protein L35 OS=Rattus norvegicus OX=10116 GN=Rpl35 PE=1 SV=3 - [RL35_RAT]	14.5	0.000E0	0.000E0	2.285E7	2.987E7			133.49	172.23			18.70	18.70			3	3			5	6
P17164	Tissue alpha-L-fucosidase OS=Rattus norvegicus GN=Fuca1 PE=1 SV=1 - [FUCO_RAT]	53.5	1.226E7	2.731E7	4.685E7	3.572E7	91.63	243.11	244.54	314.11	6.06	9.74	18.61	20.78	2	4	6	7	4	8	10	14
P17220	Proteasome subunit alpha type-2 OS=Rattus norvegicus GN=Psma2 PE=1 SV=3 - [PSA2_RAT]	25.9	0.000E0	3.692E7	7.202E7	2.349E7		372.16	744.09	408.11		44.87	50.00	49.57		8	11	10		17	26	21
P17764	Acetyl-CoA acetyltransferase, mitochondrial OS=Rattus norvegicus GN=Acat1 PE=1 SV=1 - [THIL_RAT]	44.7	0.000E0	5.043E7	9.274E7	4.371E7		807.90	944.28	545.85		50.94	41.27	47.64		13	12	13		36	41	30
P18163	Long-chain-fatty-acid--CoA ligase 1 OS=Rattus norvegicus OX=10116 GN=Acsl1 PE=1 SV=1 - [ACSL1_RAT]	78.1	5.962E6	0.000E0	0.000E0	0.000E0	114.67				7.15				4				5			
P18297	Sepiapterin reductase OS=Rattus norvegicus GN=Spr PE=1 SV=1 - [SPRE_RAT]	28.1	0.000E0	1.066E7	1.904E7	1.045E7		243.02	317.19	215.41		18.70	24.43	21.37		3	4	4		6	7	8
P18395	Cold shock domain-containing protein E1 OS=Rattus norvegicus OX=10116 GN=Csde1 PE=2 SV=1 - [CSDE1_RAT]	88.8	7.809E5	0.000E0	0.000E0	1.367E6	23.15			23.84	0.88			1.00	1			1	1			1
P18420	Proteasome subunit alpha type-1 OS=Rattus norvegicus GN=Psma1 PE=1 SV=2 - [PSA1_RAT]	29.5	0.000E0	7.708E7	1.184E8	4.616E7		583.62	851.41	352.78		57.79	66.92	55.89		12	15	12		23	34	20
P18421	Proteasome subunit beta type-1 OS=Rattus norvegicus GN=Psmb1 PE=1 SV=3 - [PSB1_RAT]	26.5	0.000E0	2.650E7	6.188E7	1.761E7		210.57	473.60	200.40		27.92	39.17	27.92		5	7	5		8	20	8
P18422	Proteasome subunit alpha type-3 OS=Rattus norvegicus GN=Psma3 PE=1 SV=3 - [PSA3_RAT]	28.4	2.605E6	4.279E7	6.420E7	2.796E7	68.11	448.32	659.17	424.55	14.12	38.43	41.57	38.82	3	10	11	9	3	18	25	18
P18445	60S ribosomal protein L27a OS=Rattus norvegicus OX=10116 GN=Rpl27a PE=1 SV=3 - [RL27A_RAT]	16.6	0.000E0	1.786E7	3.816E7	4.933E7		115.28	130.42	186.15		15.54	20.95	27.70		2	3	4		3	4	8
P18886	Carnitine O-palmitoyltransferase 2, mitochondrial OS=Rattus norvegicus OX=10116 GN=Cpt2 PE=1 SV=1 - [CPT2_RAT]	74.1	0.000E0	0.000E0	1.052E7	4.115E6			82.20	24.42			13.68	3.65			5	2			7	2
P19218	Pancreatic secretory granule membrane major glycoprotein GP2 OS=Rattus norvegicus OX=10116 GN=Gp2 PE=1 SV=1 -	58.7	0.000E0	4.590E6	8.550E6	0.000E0		25.62	81.92			6.60	10.38			3	4			3	6	
P20070	NADH-cytochrome b5 reductase 3 OS=Rattus norvegicus OX=10116 GN=Cyb5r3 PE=1 SV=2 - [NB5R3_RAT]	34.2	4.372E6	0.000E0	0.000E0	0.000E0	28.21				8.31				2				4			
P20280	60S ribosomal protein L21 OS=Rattus norvegicus GN=Rpl21 PE=1 SV=3 - [RL21_RAT]	18.5	0.000E0	0.000E0	5.262E7	2.625E7			55.18	90.54			9.38	16.25			1	2			2	3
P20788	Cytochrome b-c1 complex subunit Rieske, mitochondrial OS=Rattus norvegicus OX=10116 GN=Uqcrfs1 PE=1 SV=2 - [UCF	29.4	6.039E6	0.000E0	0.000E0	0.000E0	165.83				10.58				3				5			
P21139	Alpha-mannosidase 2C1 OS=Rattus norvegicus GN=Man2c1 PE=1 SV=1 - [MA2C1_RAT]	115.9	0.000E0	1.221E6	7.397E6	0.000E0		25.93	37.30			1.44	5.96			1	4			1	4	
P21396	Amine oxidase [flavin-containing] A OS=Rattus norvegicus GN=Maoa PE=1 SV=1 - [AOFA_RAT]	59.5	0.000E0	2.410E6	5.645E6	7.451E6		51.59	51.39	171.11		5.13	7.79	14.45		2	3	6		2	4	10
P21531	60S ribosomal protein L3 OS=Rattus norvegicus GN=Rpl3 PE=1 SV=3 - [RL3_RAT]	46.1	0.000E0	2.510E7	7.050E7	4.746E7		284.97	828.32	722.08		13.65	44.91	30.02		6	16	12		15	37	29
P21588	5'-nucleotidase OS=Rattus norvegicus GN=Nt5e PE=1 SV=1 - [5NTD_RAT]	63.9	2.983E6	0.000E0	0.000E0	0.000E0	27.36				2.60				1				2			
P21670	Proteasome subunit alpha type-4 OS=Rattus norvegicus GN=Psma4 PE=1 SV=1 - [PSA4_RAT]	29.5	6.918E6	5.820E7	8.616E7	3.273E7	35.68	572.66	873.96	495.61	3.07	49.43	65.90	49.43	1	9	11	9	1	18	28	15
P22062	Protein-L-isoaspartate(D-aspartate) O-methyltransferase OS=Rattus norvegicus GN=Pcmt1 PE=1 SV=2 - [PIMT_RAT]	24.6	0.000E0	7.619E6	1.362E7	2.503E6		144.42	105.10	41.96		15.86	30.84	7.49		2	3	1		6	6	2
P22791	Hydroxymethylglutaryl-CoA synthase, mitochondrial OS=Rattus norvegicus OX=10116 GN=Hmgcs2 PE=1 SV=1 - [HMCS2_	56.9	0.000E0	3.825E7	6.171E7	2.216E7		377.09	623.76	325.39		27.36	30.31	20.87		13	16	10		20	28	17
P23457	3-alpha-hydroxysteroid dehydrogenase OS=Rattus norvegicus GN=Akr1c9 PE=1 SV=1 - [DIDH_RAT]	37.0	6.793E7	2.408E8	3.834E8	1.932E8	833.61	1709.88	2358.18	1493.08	49.07	60.56	73.91	62.73	13	17	20	18	32	64	74	61
P24049	60S ribosomal protein L17 OS=Rattus norvegicus OX=10116 GN=Rpl17 PE=2 SV=3 - [RL17_RAT]	21.4	0.000E0	0.000E0	1.610E7	2.595E7			99.22	178.49			18.48	30.98			3	5			4	8
P24050	40S ribosomal protein S5 OS=Rattus norvegicus GN=Rps5 PE=1 SV=3 - [RS5_RAT]	22.9	3.506E6	2.129E7	5.357E7	3.456E7	30.45	167.69	361.84	391.97	6.37	28.92	47.55	33.33	1	5	9	7	1	7	16	15
P24051	40S ribosomal protein S27-like OS=Rattus norvegicus GN=Rps27l PE=1 SV=3 - [RS27L_RAT]	9.5	0.000E0	0.000E0	2.014E7	1.303E7			120.47	62.19			28.57	28.57			2	2			6	4
P24329	Thiosulfate sulfurtransferase OS=Rattus norvegicus GN=Tst PE=1 SV=3 - [THTR_RAT]	33.4	0.000E0	3.051E7	8.362E7	4.519E7		74.30	154.81	231.77		4.04	23.57	23.91		1	6	6		2	8	11
P27657	Pancreatic triacylglycerol lipase OS=Rattus norvegicus OX=10116 GN=Pnlip PE=1 SV=1 - [LIPP_RAT]	51.4	1.595E7	0.000E0	0.000E0	0.000E0	40.04				10.97				4				6			
P27791	cAMP-dependent protein kinase catalytic subunit alpha OS=Rattus norvegicus GN=Prkaca PE=1 SV=2 - [KAPCA_RAT]	40.6	0.000E0	5.821E6	9.582E6	9.104E6		27.03	64.02	39.83		9.40	11.97	2.56		2	3	1		2	4	2
P27867	Sorbitol dehydrogenase OS=Rattus norvegicus GN=Sord PE=1 SV=4 - [DHSO_RAT]	38.2	0.000E0	3.999E6	1.150E7	4.777E6		37.29	93.12	36.89		14.01	16.25	13.17		4	5	4		4	10	5
P27952	40S ribosomal protein S2 OS=Rattus norvegicus GN=Rps2 PE=1 SV=1 - [RS2_RAT]	31.2	5.017E6	5.922E7	1.135E8	7.844E7	45.25	337.39	685.01	560.17	3.75	32.42	42.32	41.98	1	8	12	10	1	15	25	25
P28073	Proteasome subunit beta type-6 OS=Rattus norvegicus GN=Psmb6 PE=1 SV=3 - [PSB6_RAT]	25.3	0.000E0	3.021E7	5.382E7	1.155E7		255.22	249.08	207.78		25.21	37.39	12.18		6	6	3		10	11	6
P28075	Proteasome subunit beta type-5 OS=Rattus norvegicus GN=Psmb5 PE=1 SV=3 - [PSB5_RAT]	28.6	0.000E0	1.205E7	3.263E7	1.238E7		121.18	516.17	171.48		13.31	39.16	28.14		3	9	6		6	17	9
P28077	Proteasome subunit beta type-9 OS=Rattus norvegicus GN=Psmb9 PE=1 SV=2 - [PSB9_RAT]	23.3	0.000E0	4.683E6	1.433E7	0.000E0		47.95	70.73			17.35	9.59			3	1			4	2	
P28480	T-complex protein 1 subunit alpha OS=Rattus norvegicus GN=Tcp1 PE=1 SV=1 - [TCPA_RAT]	60.3	0.000E0	2.290E7	4.880E7	2.207E7		278.22	709.76	394.61		21.40	47.84	29.14		9	17	12		13	30	22
P29147	D-beta-hydroxybutyrate dehydrogenase, mitochondrial OS=Rattus norvegicus OX=10116 GN=Bdh1 PE=1 SV=2 - [BDH_R/	38.2	0.000E0	2.972E6	4.149E6	4.272E6		44.33	43.47	53.63		5.25	5.25	5.25		2	2	2		3	2	3
P29314	40S ribosomal protein S9 OS=Rattus norvegicus GN=Rps9 PE=1 SV=4 - [RS9_RAT]	22.6	3.247E6	5.162E7	1.346E8	9.905E7	29.46	205.91	429.47	496.40	4.12	30.41	36.60	45.36	1	7	10	14	1	11	22	31
P29315	Ribonuclease inhibitor OS=Rattus norvegicus GN=Rnh1 PE=1 SV=2 - [RINI_RAT]	49.9	0.000E0	2.003E7	4.678E7	4.857E7		569.23	833.00	466.80		42.11	56.36	49.12		11	16	14		18	29	23
P29410	Adenylate kinase 2, mitochondrial OS=Rattus norvegicus GN=Ak2 PE=2 SV=2 - [KAD2_RAT]	26.4	3.536E7	1.240E8	1.868E8	1.016E8	425.30	1030.07	1288.20	1075.89	54.39	70.29	70.29	65.27	11	15	15	13	22	36	48	39

P29411	GTP:AMP phosphotransferase AK3, mitochondrial OS=Rattus norvegicus GN=Ak3 PE=2 SV=2 - [KAD3_RAT]	25.4	5.194E6	1.232E7	1.777E7	9.969E6	73.77	300.10	242.36	196.64	14.54	24.23	14.54	14.54	3	5	3	3	4	10	8	7
P29476	Nitric oxide synthase, brain OS=Rattus norvegicus OX=10116 GN=Nos1 PE=1 SV=1 - [NOS1_RAT]	160.5	3.617E6	0.000E0	0.000E0	0.000E0	21.45				0.63				1				1			
P30349	Leukotriene A-4 hydrolase OS=Rattus norvegicus GN=Lta4h PE=2 SV=2 - [LKHA4_RAT]	69.1	8.624E6	1.092E8	1.480E8	6.046E7	49.25	1592.86	2046.65	1697.19	1.97	43.28	46.07	46.07	1	21	22	22	2	56	71	68
P30835	ATP-dependent 6-phosphofructokinase, liver type OS=Rattus norvegicus GN=Pfkl PE=2 SV=3 - [PFKAL_RAT]	85.3	0.000E0	9.560E6	2.257E7	1.083E7		80.64	240.74	78.53		6.67	10.90	10.26		3	6	5		5	11	7
P31053	Leukocyte antigen CD37 OS=Rattus norvegicus OX=10116 GN=Cd37 PE=2 SV=1 - [CD37_RAT]	31.7	2.337E6	0.000E0	0.000E0	0.000E0	20.98				2.85				1				1			
P32362	Uroporphyrinogen decarboxylase (Fragment) OS=Rattus norvegicus GN=Urod PE=1 SV=1 - [DCUP_RAT]	40.4	2.456E6	2.164E7	3.345E7	1.951E7	32.07	277.64	364.43	273.46	1.92	21.98	21.98	21.98	1	5	5	5	1	11	16	10
P34064	Proteasome subunit alpha type-5 OS=Rattus norvegicus GN=Psma5 PE=2 SV=1 - [PSA5_RAT]	26.4	1.893E7	7.759E7	1.060E8	2.966E7	118.02	482.49	565.85	294.40	13.28	46.89	46.89	25.31	2	8	8	4	3	13	20	9
P34067	Proteasome subunit beta type-4 OS=Rattus norvegicus GN=Psmb4 PE=1 SV=2 - [PSB4_RAT]	29.2	0.000E0	3.052E7	5.145E7	1.539E7		368.43	604.35	208.43		28.14	37.26	31.18		5	6	6		17	20	11
P35171	Cytochrome c oxidase subunit 7A2, mitochondrial OS=Rattus norvegicus OX=10116 GN=Cox7a2 PE=1 SV=1 - [CX7A2_RA	9.3	4.320E6	0.000E0	0.000E0	5.481E6	38.43	51.41		67.02	12.05	12.05		12.05	1	1		1	1	1		1
P35435	ATP synthase subunit gamma, mitochondrial OS=Rattus norvegicus GN=Atp5c1 PE=1 SV=2 - [ATPG_RAT]	30.2	0.000E0	0.000E0	5.827E6	6.241E6			32.44	37.99			4.03	7.69			1	2			1	2
P36876	Serine/threonine-protein phosphatase 2A 55 kDa regulatory subunit B alpha isoform OS=Rattus norvegicus GN=Ppp2r2a P	51.6	0.000E0	4.767E6	1.030E7	0.000E0		38.68	91.38			8.05	8.05			3	3			5	5	
P37805	Transgelin-3 OS=Rattus norvegicus GN=Tagln3 PE=1 SV=2 - [TAGL3_RAT]	22.5	0.000E0	1.607E7	3.084E7	1.534E7		58.34	55.88	48.99		11.06	4.02	4.02		2	1	1		3	2	2
P38650	Cytoplasmic dynein 1 heavy chain 1 OS=Rattus norvegicus GN=Dync1h1 PE=1 SV=1 - [DYHC1_RAT]	531.9	0.000E0	1.508E7	2.282E7	9.473E6		225.31	1117.67	392.64		3.47	12.19	6.01		11	42	22		16	66	33
P38652	Phosphoglucomutase-1 OS=Rattus norvegicus GN=Pgm1 PE=1 SV=2 - [PGM1_RAT]	61.4	0.000E0	1.050E7	1.715E7	1.105E7		567.70	690.58	647.04		27.40	37.90	30.25		11	14	12		20	24	21
P38983	40S ribosomal protein SA OS=Rattus norvegicus GN=Rpsa PE=1 SV=3 - [RSSA_RAT]	32.8	6.641E7	1.475E8	2.280E8	1.660E8	543.54	615.32	1422.74	1334.82	30.85	31.86	39.66	51.86	6	7	8	11	16	15	31	37
P39032	60S ribosomal protein L36 OS=Rattus norvegicus OX=10116 GN=Rpl36 PE=1 SV=2 - [RL36_RAT]	12.3	0.000E0	0.000E0	2.234E7	1.141E7			96.28	129.95			12.38	20.95			2	3			4	4
P39052	Dynamin-2 OS=Rattus norvegicus GN=Dnm2 PE=1 SV=1 - [DYN2_RAT]	98.2	0.000E0	0.000E0	6.255E6	3.696E6			41.77	23.24			5.52	4.37			4	3			6	4
P40112	Proteasome subunit beta type-3 OS=Rattus norvegicus GN=Psmb3 PE=1 SV=1 - [PSB3_RAT]	22.9	5.799E6	3.321E7	5.739E7	2.003E7	34.13	246.89	426.63	199.57	8.78	27.32	38.54	20.00	1	4	7	3	2	13	17	10
P40307	Proteasome subunit beta type-2 OS=Rattus norvegicus GN=Psmb2 PE=1 SV=1 - [PSB2_RAT]	22.9	4.279E6	2.749E7	5.264E7	2.056E7	41.33	247.46	286.51	217.16	5.47	20.90	36.32	24.38	1	3	4	4	2	10	13	10
P40329	Arginine--tRNA ligase, cytoplasmic OS=Rattus norvegicus GN=Rars PE=1 SV=2 - [SYRC_RAT]	75.8	0.000E0	3.127E7	4.502E7	2.052E7		516.95	855.52	562.98		35.76	36.06	28.79		19	21	14		26	39	25
P41123	60S ribosomal protein L13 OS=Rattus norvegicus GN=Rpl13 PE=1 SV=2 - [RL13_RAT]	24.3	0.000E0	4.761E6	1.504E7	8.483E6		64.65	71.78	143.41		5.21	5.21	14.69		1	1	3		2	1	6
P41350	Caveolin-1 OS=Rattus norvegicus OX=10116 GN=Cav1 PE=1 SV=3 - [CAV1_RAT]	20.5	0.000E0	0.000E0	7.388E6	2.341E6			37.27	40.57			4.49	4.49			1	1			1	2
P41562	Isocitrate dehydrogenase [NADP] cytoplasmic OS=Rattus norvegicus GN=Idh1 PE=1 SV=1 - [IDHC_RAT]	46.7	2.170E6	1.534E8	2.557E8	1.311E8	22.53	1472.10	2159.55	1606.42	3.14	53.38	64.73	62.56	1	21	23	22	1	57	75	68
P42123	L-lactate dehydrogenase B chain OS=Rattus norvegicus GN=Ldhb PE=1 SV=2 - [LDHB_RAT]	36.6	1.816E7	1.243E8	1.897E8	9.159E7	219.68	442.20	546.35	378.11	6.29	25.15	29.04	17.96	2	8	10	7	3	15	22	13
P43244	Matrin-3 OS=Rattus norvegicus GN=Matr3 PE=1 SV=2 - [MATR3_RAT]	94.4	0.000E0	2.941E7	4.673E7	3.757E7		141.36	201.21	248.28		6.39	6.63	9.82		3	4	6		5	10	12
P43424	Galactose-1-phosphate uridylyltransferase OS=Rattus norvegicus OX=10116 GN=Galt PE=2 SV=1 - [GALT_RAT]	43.3	0.000E0	0.000E0	1.122E7	4.031E6			41.47	37.42			7.12	6.33			2	2			3	4
P45953	Very long-chain specific acyl-CoA dehydrogenase, mitochondrial OS=Rattus norvegicus OX=10116 GN=Acadvl PE=1 SV=1	70.7	7.870E6	0.000E0	0.000E0	0.000E0	127.92				12.82				6				9			
P46101	Dipeptidyl aminopeptidase-like protein 6 OS=Rattus norvegicus GN=Dpp6 PE=1 SV=1 - [DPP6_RAT]	97.2	2.382E6	0.000E0	0.000E0	0.000E0	28.65				2.33				2				2			
P46462	Transitional endoplasmic reticulum ATPase OS=Rattus norvegicus GN=Vcp PE=1 SV=3 - [TERA_RAT]	89.3	0.000E0	1.363E8	2.377E8	1.277E8		2658.22	4137.70	3085.88		45.53	59.80	67.74		28	38	40		83	125	110
P46844	Biliverdin reductase A OS=Rattus norvegicus GN=Blvra PE=1 SV=1 - [BIEA_RAT]	33.5	3.099E6	1.680E7	2.489E7	1.316E7	54.10	331.29	381.95	279.53	14.58	45.08	50.85	41.36	3	10	10	9	3	18	20	15
P47245	Nardilysin OS=Rattus norvegicus GN=Nrd1 PE=1 SV=1 - [NRDC_RAT]	132.9	0.000E0	3.897E6	6.834E6	3.370E6		93.31	92.39	46.21		2.15	2.24	2.33		3	3	3		4	4	4
P47942	Dihydropyrimidinase-related protein 2 OS=Rattus norvegicus GN=Dpysl2 PE=1 SV=1 - [DPYL2_RAT]	62.2	0.000E0	1.131E8	1.577E8	5.620E7		1485.17	1996.86	1129.71		59.09	56.29	48.78		22	21	19		54	59	41
P48004	Proteasome subunit alpha type-7 OS=Rattus norvegicus GN=Psma7 PE=1 SV=1 - [PSA7_RAT]	28.3	0.000E0	7.383E7	1.253E8	5.105E7		397.93	567.66	393.52		44.49	44.49	44.49		9	9	9		14	19	18
P48284	Carbonic anhydrase 4 OS=Rattus norvegicus OX=10116 GN=Ca4 PE=1 SV=1 - [CAH4_RAT]	35.1	1.081E7	0.000E0	0.000E0	0.000E0	45.32				7.77				2				2			
P48508	Glutamate--cysteine ligase regulatory subunit OS=Rattus norvegicus GN=Gclm PE=1 SV=1 - [GSH0_RAT]	30.5	0.000E0	0.000E0	5.699E6	2.991E6			33.15	53.31			5.47	11.31			1	2			1	3
P49242	40S ribosomal protein S3a OS=Rattus norvegicus GN=Rps3a PE=1 SV=2 - [RS3A_RAT]	29.9	4.643E6	7.789E7	1.363E8	1.122E8	33.38	322.37	503.29	448.32	3.03	33.71	39.02	35.61	1	8	10	9	1	17	24	25
P49432	Pyruvate dehydrogenase E1 component subunit beta, mitochondrial OS=Rattus norvegicus GN=Pdhb PE=1 SV=2 - [ODPB	39.0	0.000E0	5.091E6	1.066E7	6.561E6		51.00	161.32	73.04		11.14	16.16	11.70		3	5	4		4	8	6
P50116	Protein S100-A9 OS=Rattus norvegicus OX=10116 GN=S100a9 PE=1 SV=3 - [S10A9_RAT]	13.1	0.000E0	3.495E6	1.673E7	1.095E7		25.17	32.75	25.99		6.19	6.19	6.19		1	1	1		2	2	2
P50878	60S ribosomal protein L4 OS=Rattus norvegicus GN=Rpl4 PE=1 SV=3 - [RL4_RAT]	47.2	0.000E0	4.973E7	1.072E8	7.284E7		525.30	968.71	735.96		22.33	34.92	34.92		9	13	12		20	40	31
P51574	Solute carrier family 15 member 1 OS=Rattus norvegicus OX=10116 GN=Slc15a1 PE=2 SV=1 - [S15A1_RAT]	78.9	8.084E6	0.000E0	0.000E0	0.000E0	86.96				3.94				2				4			
P51607	N-acylglucosamine 2-epimerase OS=Rattus norvegicus GN=Renbp PE=2 SV=2 - [RENBP_RAT]	49.6	3.715E6	1.071E7	3.012E7	1.298E7	34.03	109.07	387.18	209.58	4.88	13.49	30.70	24.88	2	4	14	11	3	6	21	18
P51650	Succinate-semialdehyde dehydrogenase, mitochondrial OS=Rattus norvegicus GN=Aldh5a1 PE=1 SV=2 - [SSDH_RAT]	56.1	0.000E0	4.370E6	9.166E6	9.970E6		52.13	70.01	62.01		3.82	7.27	3.82		2	3	2		2	4	4
P52020	Squalene monooxygenase OS=Rattus norvegicus OX=10116 GN=Sqle PE=2 SV=1 - [ERG1_RAT]	64.0	4.567E6	0.000E0	0.000E0	0.000E0	20.43				1.22				1				1			
P52296	Importin subunit beta-1 OS=Rattus norvegicus GN=Kpnb1 PE=1 SV=1 - [IMB1_RAT]	97.1	3.410E6	3.037E7	4.742E7	1.395E7	90.10	494.51	1034.80	486.73	4.57	19.20	29.83	17.49	3	11	18	11	4	20	42	21
P52631	Signal transducer and activator of transcription 3 OS=Rattus norvegicus GN=Stat3 PE=1 SV=1 - [STAT3_RAT]	88.0	0.000E0	8.580E6	1.429E7	0.000E0		56.30	234.10			4.94	12.73			3	7			3	13	
P53534	Glycogen phosphorylase, brain form (Fragment) OS=Rattus norvegicus GN=Pygb PE=1 SV=3 - [PYGB_RAT]	96.1	5.839E6	2.238E7	4.282E7	2.348E7	20.60	643.43	881.06	711.21	1.43	22.32	35.80	33.29	1	14	23	21	2	22	40	35
P53790	Sodium/glucose cotransporter 1 OS=Rattus norvegicus OX=10116 GN=Slc5a1 PE=2 SV=1 - [SC5A1_RAT]	73.0	1.372E7	0.000E0	0.000E0	0.000E0	135.85				5.11				4				6			
P55091	Chymotrypsin-C OS=Rattus norvegicus OX=10116 GN=Ctrc PE=1 SV=1 - [CTRC_RAT]	29.4	0.000E0	0.000E0	8.667E6	7.515E6			46.28	34.91			14.93	4.10			3	1			3	2
P55213	Caspase-3 OS=Rattus norvegicus GN=Casp3 PE=2 SV=2 - [CASP3_RAT]	31.5	0.000E0	1.248E7	2.890E7	1.069E7	28.36	139.10	160.98	108.41	2.89	11.19	23.10	6.86	1	3	5	2	2	4	6	5
P56571	ES1 protein homolog, mitochondrial OS=Rattus norvegicus PE=1 SV=2 - [ES1_RAT]	28.2	0.000E0	1.634E7	2.393E7	2.305E7		22.09	32.43	26.97		9.40	15.04	9.40		1	2	1		2	4	2
P56574	Isocitrate dehydrogenase [NADP], mitochondrial OS=Rattus norvegicus GN=Idh2 PE=1 SV=2 - [IDHP_RAT]	50.9	0.000E0	1.016E8	1.714E8	9.216E7		1270.17	1467.68	972.27		47.35	54.20	46.46		20	22	21		51	59	49
P60123	RuvB-like 1 OS=Rattus norvegicus GN=Ruvbl1 PE=1 SV=1 - [RUVB1_RAT]	50.2	0.000E0	2.810E6	1.700E7	6.811E6		37.19	225.87	123.96		5.92	21.49	15.79		2	7	5		4	11	7
P60711	Actin, cytoplasmic 1 OS=Rattus norvegicus GN=Actb PE=1 SV=1 - [ACTB_RAT]	41.7	2.439E8	1.494E9	2.861E9	1.702E9	2031.99	5706.83	8737.00	7377.28	67.73	72.53	71.73	73.07	20	22	24	24	76	240	356	326
P60868	40S ribosomal protein S20 OS=Rattus norvegicus GN=Rps20 PE=3 SV=1 - [RS20_RAT]	13.4	0.000E0	9.005E6	4.572E7	2.994E7		72.98	191.89	194.08		15.13	28.57	25.21		2	4	3		2	8	6
P60892	Ribose-phosphate pyrophosphokinase 1 OS=Rattus norvegicus GN=Prps1 PE=1 SV=2 - [PRPS1_RAT]	34.8	0.000E0	6.299E6	9.162E6	4.342E6		130.42	170.85	38.88		14.47	19.18	10.69		3	4	2		5	6	2
P60901	Proteasome subunit alpha type-6 OS=Rattus norvegicus GN=Psma6 PE=1 SV=1 - [PSA6_RAT]	27.4	1.228E7	6.782E7	1.093E8	4.034E7	63.93	617.28	844.59	581.03	4.88	48.78	48.78	47.97	1	10	11	10	2	19	26	18
P61023	Calcineurin B homologous protein 1 OS=Rattus norvegicus OX=10116 GN=Chp1 PE=1 SV=2 - [CHP1_RAT]	22.4	6.846E6	0.000E0	0.000E0	0.000E0	46.61				6.15				1				1			
P61203	COP9 signalosome complex subunit 2 OS=Rattus norvegicus GN=Cops2 PE=2 SV=1 - [CSN2_RAT]	51.6	0.000E0	8.935E6	1.637E7	6.253E6		70.29	169.45	86.33		7.00	19.64	9.93		3	8	4		6	11	6
P61314	60S ribosomal protein L15 OS=Rattus norvegicus GN=Rpl15 PE=1 SV=2 - [RL15_RAT]	24.1	0.000E0	6.831E7	1.123E8	7.187E7		509.41	664.42	640.79		45.59	57.35	52.94		9	13	12		23	29	28
P61515	Putative 60S ribosomal protein L37a OS=Rattus norvegicus OX=10116 GN=Rpl37a-ps1 PE=5 SV=2 - [RL37P_RAT]	10.3	0.000E0	3.359E6	1.080E7	1.101E7		51.68	64.66	155.73		8.70	28.26	41.30		1	2	3		2	3	5
P62193	26S protease regulatory subunit 4 OS=Rattus norvegicus GN=Psmc1 PE=2 SV=1 - [PRS4_RAT]	49.2	0.000E0	0.000E0	6.085E6	2.944E6			102.59	20.73			11.14	4.77			3	1			5	1
P62243	40S ribosomal protein S8 OS=Rattus norvegicus GN=Rps8 PE=1 SV=2 - [RS8_RAT]	24.2	0.000E0	1.061E7	8.596E7	8.504E7		222.66	684.68	568.82		41.35	41.35	50.96		7	7	9		9	16	21
P62250	40S ribosomal protein S16 OS=Rattus norvegicus GN=Rps16 PE=1 SV=2 - [RS16_RAT]	16.4	4.882E6	3.788E7	1.315E8	7.684E7	43.74	210.44	390.13	402.54	6.85	26.03	45.21	47.26	1	4	7	7	2	10	15	16
P62268	40S ribosomal protein S23 OS=Rattus norvegicus GN=Rps23 PE=1 SV=3 - [RS23_RAT]	15.8	0.000E0	0.000E0	1.436E7	2.164E7			78.67	187.24			18.88	26.57			1	2			2	4
P62271	40S ribosomal protein S18 OS=Rattus norvegicus GN=Rps18 PE=1 SV=3 - [RS18_RAT]	17.7	0.000E0	2.440E7	1.050E8	6.443E7		297.41	549.44	494.17		25.00	40.13	36.18		4	7	6		8	17	16
P62278	40S ribosomal protein S13 OS=Rattus norvegicus GN=Rps13 PE=1 SV=2 - [RS13_RAT]	17.2	6.667E6	2.728E7	4.603E7	4.769E7	49.81	70.57	166.73	235.68	4.64	4.64	33.77	40.40	1	1	6	7	2	2	10	12
P62282	40S ribosomal protein S11 OS=Rattus norvegicus GN=Rps11 PE=1 SV=3 - [RS11_RAT]	18.4	0.000E0	0.000E0	1.644E7	2.395E7			83.63	223.29			20.25	36.71			3	6			5	12
P62329	Thymosin beta-4 OS=Rattus norvegicus OX=10116 GN=Tmsb4x PE=1 SV=2 - [TYB4_RAT]	5.0	5.351E6	0.000E0	0.000E0	0.000E0	21.81				29.55				1				1			
P62703	40S ribosomal protein S4, X isoform OS=Rattus norvegicus GN=Rps4x PE=2 SV=2 - [RS4X_RAT]	29.6	3.891E6	3.675E7	8.244E7	4.968E7	44.54	502.07	667.83	441.81	7.98	36.88	46.39	40.30	2	9	12	10	4	20	34	20
P62738	Actin, aortic smooth muscle OS=Rattus norvegicus GN=Acta2 PE=2 SV=1 - [ACTA_RAT]	42.0	1.997E8	9.862E8	1.968E9	1.334E9	1210.40	3554.48	4854.25	4726.31	53.85	70.56	71.09	70.03	16	21	23	21	52	159	207	213
P62752	60S ribosomal protein L23a OS=Rattus norvegicus GN=Rpl23a PE=2 SV=1 - [RL23A_RAT]	17.7	0.000E0	0.000E0	1.365E7	1.758E7			134.53	238.49			14.74	20.51			2	4			3	11
P62755	40S ribosomal protein S6 OS=Rattus norvegicus OX=10116 GN=Rps6 PE=1 SV=1 - [RS6_RAT]	28.7	0.000E0	0.000E0	2.145E7	3.248E7			98.31	240.85			10.84	20.88			2	4			2	9
P62815	V-type proton ATPase subunit B, brain isoform OS=Rattus norvegicus GN=Atp6v1b2 PE=1 SV=1 - [VATB2_RAT]	56.5	0.000E0	6.635E6	2.481E7	9.622E6		22.51	434.48	127.12		8.22	38.55	10.76		2	12	4		2	20	7
P62832	60S ribosomal protein L23 OS=Rattus norvegicus GN=Rpl23 PE=2 SV=1 - [RL23_RAT]	14.9	0.000E0	5.878E6	1.749E7	2.301E7		38.08	174.46	159.32		20.00	30.71	25.71		2	3	3		3	6	7
P62836	Ras-related protein Rap-1A OS=Rattus norvegicus GN=Rap1a PE=1 SV=1 - [RAP1A_RAT]	21.0	0.000E0	3.529E6	8.072E6	4.794E6		95.81	197.07	140.63		10.33	15.76	15.76		2	3	3		3	8	6
P62845	40S ribosomal protein S15 OS=Rattus norvegicus GN=Rps15 PE=1 SV=2 - [RS15_RAT]	17.0	0.000E0	2.449E7	4.305E7	1.662E7		56.35	74.66	61.27		40.00	40.69	22.07		3	4	3		6	12	8
P62850	40S ribosomal protein S24 OS=Rattus norvegicus GN=Rps24 PE=2 SV=1 - [RS24_RAT]	15.4	0.000E0	7.248E6	2.252E7	2.255E7		49.98	178.71	343.61		11.28	11.28	20.30		1	1	2		1	3	7
P62853	40S ribosomal protein S25 OS=Rattus norvegicus GN=Rps25 PE=2 SV=1 - [RS25_RAT]	13.7	6.234E6	6.000E7	1.468E8	8.809E7	76.42	298.36	460.44	452.45	15.20	29.60	29.60	29.60	2	4	5	5	3	10	15	14
P62856	40S ribosomal protein S26 OS=Rattus norvegicus GN=Rps26 PE=3 SV=3 - [RS26_RAT]	13.0	0.000E0	3.823E6	1.797E7	2.329E7		43.63	135.65	52.29		7.83	20.87	20.87		1	2	2		2	4	4
P62864	40S ribosomal protein S30 OS=Rattus norvegicus OX=10116 GN=Fau PE=1 SV=1 - [RS30_RAT]	6.6	0.000E0	0.000E0	6.867E6	3.734E7			22.28	78.79			16.95	16.95			1	1			1	2
P62898	Cytochrome c, somatic OS=Rattus norvegicus GN=Cycs PE=1 SV=2 - [CYC_RAT]	11.6	1.041E7	6.088E6	4.554E6	5.548E6	93.50	70.78	74.04	80.49	18.10	18.10	18.10	18.10	2	2	2	2	4	3	3	3
P62902	60S ribosomal protein L31 OS=Rattus norvegicus OX=10116 GN=Rpl31 PE=2 SV=1 - [RL31_RAT]	14.5	0.000E0	0.000E0	1.086E7	1.446E7			79.52	77.22			17.60	17.60			2	2			5	4
P62909	40S ribosomal protein S3 OS=Rattus norvegicus GN=Rps3 PE=1 SV=1 - [RS3_RAT]	26.7	1.308E7	6.827E7	1.429E8	8.107E7	309.96	934.57	1126.18	1074.78	33.74	61.73	61.73	61.73	8	14	14	14	13	34	38	35
P62912	60S ribosomal protein L32 OS=Rattus norvegicus OX=10116 GN=Rpl32 PE=1 SV=2 - [RL32_RAT]	15.8	0.000E0	0.000E0	3.897E7	2.794E7			262.57	229.53			33.33	33.33			4	4			9	11
P62914	60S ribosomal protein L11 OS=Rattus norvegicus GN=Rpl11 PE=1 SV=2 - [RL11_RAT]	20.2	0.000E0	1.665E7	4.591E7	5.307E7		52.36	210.69	317.86		16.85	21.35	21.35		3	4	4		3	8	11
P62919	60S ribosomal protein L8 OS=Rattus norvegicus GN=Rpl8 PE=2 SV=2 - [RL8_RAT]	28.0	0.000E0	5.258E7	8.098E7	5.468E7		264.52	303.58	316.82		35.80	42.41	45.14		8	9	10		16	17	19
P62961	Nuclease-sensitive element-binding protein 1 OS=Rattus norvegicus OX=10116 GN=Ybx1 PE=2 SV=3 - [YBOX1_RAT]	35.7	0.000E0	0.000E0	5.062E6	5.944E6			51.14	59.77			5.90	5.90			1	1			1	2
P62963	Profilin-1 OS=Rattus norvegicus GN=Pfn1 PE=1 SV=2 - [PROF1_RAT]	14.9	1.269E8	8.274E8	1.162E9	6.248E8	1062.24	2358.91	2715.85	2051.98	63.57	65.00	73.57	73.57	8	10	11	11	38	81	90	66
P62997	Transformer-2 protein homolog beta OS=Rattus norvegicus OX=10116 GN=Tra2b PE=1 SV=1 - [TRA2B_RAT]	33.6	2.411E6	2.347E7	4.111E7	3.081E7	69.98	284.66	410.94	438.65	8.68	15.97	15.97	26.74	2	4	4	6	3	9	11	13
P63036	DnaJ homolog subfamily A member 1 OS=Rattus norvegicus GN=Dnaja1 PE=1 SV=1 - [DNJA1_RAT]	44.8	0.000E0	1.126E8	1.845E8	9.182E7		35.43	39.60	21.78		10.08	13.35	6.80		3	4	2		7	8	5
P63039	60 kDa heat shock protein, mitochondrial OS=Rattus norvegicus GN=Hspd1 PE=1 SV=1 - [CH60_RAT]	60.9	0.000E0	0.000E0	1.932E7	8.937E6			446.27	187.10			24.08	14.14			10	6			18	12
P63155	Crooked neck-like protein 1 OS=Rattus norvegicus GN=Crnkl1 PE=2 SV=1 - [CRNL1_RAT]	83.4	0.000E0	3.440E6	2.025E7	0.000E0		57.73	22.04			6.96	3.62			4	2			4	3	
P63312	Thymosin beta-10 OS=Rattus norvegicus OX=10116 GN=Tmsb10 PE=1 SV=2 - [TYB10_RAT]	5.0	3.646E6	0.000E0	0.000E0	2.845E6	65.74			52.05	18.18			18.18	1			1	2			1
P63324	40S ribosomal protein S12 OS=Rattus norvegicus GN=Rps12 PE=1 SV=2 - [RS12_RAT]	14.5	0.000E0	2.885E6	4.547E7	1.013E7		26.49	262.14	130.08		7.58	42.42	35.61		1	5	4		1	11	9
P63326	40S ribosomal protein S10 OS=Rattus norvegicus GN=Rps10 PE=2 SV=1 - [RS10_RAT]	18.9	0.000E0	1.015E7	2.520E7	2.303E7		50.08	216.17	266.28		5.45	22.42	22.42		1	4	4		1	10	14
P67779	Prohibitin OS=Rattus norvegicus OX=10116 GN=Phb PE=1 SV=1 - [PHB_RAT]	29.8	0.000E0	0.000E0	3.270E6	5.112E6			43.23	52.30			4.04	7.35			1	2			1	3
P68182	cAMP-dependent protein kinase catalytic subunit beta OS=Rattus norvegicus GN=Prkacb PE=1 SV=2 - [KAPCB_RAT]	40.7	0.000E0	4.947E6	7.240E6	6.239E6		42.66	106.67	97.35		5.98	8.55	5.98		2	3	2		2	4	4
P69060	N-acylneuraminate cytidylyltransferase OS=Rattus norvegicus GN=Cmas PE=2 SV=1 - [NEUA_RAT]	48.1	0.000E0	0.000E0	5.441E6	4.999E6			44.29	72.90			5.56	5.56			2	2			3	4
P70473	Alpha-methylacyl-CoA racemase OS=Rattus norvegicus OX=10116 GN=Amacr PE=1 SV=3 - [AMACR_RAT]	41.8	6.965E6	0.000E0	0.000E0	0.000E0	54.65				2.36				1				2			
P70490	Lactadherin OS=Rattus norvegicus GN=Mfge8 PE=2 SV=1 - [MFGM_RAT]	47.4	1.523E7	0.000E0	0.000E0	0.000E0	282.49				17.56				6				11			
P70552	GTP cyclohydrolase 1 feedback regulatory protein OS=Rattus norvegicus GN=Gchfr PE=1 SV=3 - [GFRP_RAT]	9.7	1.318E7	0.000E0	0.000E0	0.000E0	224.46				52.38				3				6			
P70564	Serpin B5 OS=Rattus norvegicus OX=10116 GN=Serpinb5 PE=2 SV=1 - [SPB5_RAT]	42.0	0.000E0	1.037E7	1.955E7	1.316E7		82.48	160.18	153.62		14.93	23.47	26.13		4	7	8		7	10	11
P70618	Mitogen-activated protein kinase 14 OS=Rattus norvegicus GN=Mapk14 PE=2 SV=3 - [MK14_RAT]	41.3	3.984E6	2.667E7	4.041E7	2.035E7	25.67	177.66	178.52	133.21	2.22	6.39	6.39	10.56	1	2	2	3	1	6	7	8
P70645	Bleomycin hydrolase OS=Rattus norvegicus GN=Blmh PE=1 SV=1 - [BLMH_RAT]	52.3	0.000E0	3.529E7	5.644E7	2.692E7		402.35	464.84	237.27		21.81	38.99	27.53		7	11	8		13	20	14
P81718	Tyrosine-protein phosphatase non-receptor type 6 OS=Rattus norvegicus OX=10116 GN=Ptpn6 PE=1 SV=1 - [PTN6_RAT]	69.5	0.000E0	0.000E0	7.042E6	2.755E6			149.45	25.37			13.54	6.04			6	3			9	3
P81799	N-acetyl-D-glucosamine kinase OS=Rattus norvegicus GN=Nagk PE=1 SV=4 - [NAGK_RAT]	37.2	6.460E6	1.595E7	2.727E7	9.224E6	213.17	224.46	391.20	193.69	15.74	28.86	32.94	13.70	5	6	8	3	10	8	14	6
P82450	Sialate O-acetylesterase OS=Rattus norvegicus OX=10116 GN=Siae PE=1 SV=2 - [SIAE_RAT]	60.4	5.198E6	0.000E0	0.000E0	0.000E0	90.09				12.73				4				7			
P83645	Glutathione peroxidase 2 OS=Rattus norvegicus OX=10116 GN=Gpx2 PE=1 SV=3 - [GPX2_RAT]	22.0	2.733E6	2.658E7	4.212E7	2.009E7	35.83	423.93	423.79	453.85	10.00	52.63	71.58	47.37	2	9	12	10	2	15	22	19
P83883	60S ribosomal protein L36a OS=Rattus norvegicus OX=10116 GN=Rpl36a PE=1 SV=2 - [RL36A_RAT]	12.4	0.000E0	0.000E0	9.536E6	1.673E7			75.96	76.85			16.04	16.04			2	2			3	3
P84092	AP-2 complex subunit mu OS=Rattus norvegicus GN=Ap2m1 PE=1 SV=1 - [AP2M1_RAT]	49.6	0.000E0	1.128E7	1.584E7	6.820E6		117.34	216.80	83.95		11.26	15.63	11.26		4	6	4		7	10	5
P84100	60S ribosomal protein L19 OS=Rattus norvegicus OX=10116 GN=Rpl19 PE=1 SV=1 - [RL19_RAT]	23.5	0.000E0	6.449E6	3.776E7	2.553E7		102.29	370.29	306.14		13.27	13.27	13.78		2	2	3		2	6	7
P84817	Mitochondrial fission 1 protein OS=Rattus norvegicus GN=Fis1 PE=1 SV=1 - [FIS1_RAT]	17.0	4.819E6	0.000E0	0.000E0	0.000E0	79.41				23.03				3				5			
P84855	Major urinary protein (Fragments) OS=Rattus rattus OX=10117 PE=1 SV=2 - [MUP_RATRT]	5.3	3.425E6	0.000E0	0.000E0	0.000E0	59.65				36.96				2				4			
P85515	Alpha-centractin OS=Rattus norvegicus GN=Actr1a PE=1 SV=1 - [ACTZ_RAT]	42.6	0.000E0	3.363E6	2.014E7	8.494E6		33.37	167.41	67.36		4.26	22.07	19.95		1	5	4		2	10	9
P85845	Fascin OS=Rattus norvegicus GN=Fscn1 PE=1 SV=2 - [FSCN1_RAT]	54.5	0.000E0	3.330E7	6.000E7	2.907E7		419.51	505.86	364.89		19.68	29.61	17.24		7	10	6		13	19	11
P85970	Actin-related protein 2/3 complex subunit 2 OS=Rattus norvegicus GN=Arpc2 PE=1 SV=1 - [ARPC2_RAT]	34.4	0.000E0	4.387E7	6.935E7	3.347E7		282.94	405.72	195.19		28.67	42.67	28.33		9	11	8		17	20	14
P97519	Hydroxymethylglutaryl-CoA lyase, mitochondrial OS=Rattus norvegicus OX=10116 GN=Hmgcl PE=2 SV=1 - [HMGCL_RAT]	34.2	2.289E6	1.766E7	2.605E7	7.935E6	30.44	348.01	389.77	208.66	4.31	23.08	25.85	19.08	1	5	6	4	1	11	12	9
P97532	3-mercaptopyruvate sulfurtransferase OS=Rattus norvegicus OX=10116 GN=Mpst PE=1 SV=3 - [THTM_RAT]	32.9	0.000E0	9.046E6	1.508E7	1.126E7		82.96	208.32	223.97		8.42	18.86	12.46		2	5	3		2	9	6
P97694	Cytohesin-1 OS=Rattus norvegicus GN=Cyth1 PE=1 SV=1 - [CYH1_RAT]	46.2	0.000E0	0.000E0	4.421E6	2.933E6			41.95	30.63			2.76	2.76			1	1			2	2
P97834	COP9 signalosome complex subunit 1 OS=Rattus norvegicus GN=Gps1 PE=1 SV=1 - [CSN1_RAT]	53.4	0.000E0	4.213E6	9.790E6	4.753E6		84.45	131.44	35.64		4.46	9.77	1.49		2	5	1		3	7	2
Q01986	Dual specificity mitogen-activated protein kinase kinase 1 OS=Rattus norvegicus GN=Map2k1 PE=1 SV=2 - [MP2K1_RAT]	43.4	0.000E0	0.000E0	1.114E7	5.703E6			71.47	45.82			7.63	5.60			2	1			2	2
Q02253	Methylmalonate-semialdehyde dehydrogenase [acylating], mitochondrial OS=Rattus norvegicus GN=Aldh6a1 PE=1 SV=1 -	57.8	0.000E0	0.000E0	1.295E7	4.466E6			249.10	65.55			9.16	7.66			4	3			6	4
Q04631	Protein farnesyltransferase/geranylgeranyltransferase type-1 subunit alpha OS=Rattus norvegicus GN=Fnta PE=1 SV=1 - [	44.0	0.000E0	5.425E6	8.290E6	0.000E0		47.36	89.11			9.02	10.61			3	3			4	6	
Q07205	Eukaryotic translation initiation factor 5 OS=Rattus norvegicus GN=Eif5 PE=1 SV=1 - [IF5_RAT]	48.9	0.000E0	1.144E7	1.684E7	9.079E6		142.07	103.11	137.46		15.15	5.59	11.66		5	2	4		7	6	6
Q07969	Platelet glycoprotein 4 OS=Rattus norvegicus OX=10116 GN=Cd36 PE=1 SV=3 - [CD36_RAT]	52.7	1.756E7	0.000E0	0.000E0	0.000E0	98.06				14.19				5				7			
Q08849	Syntaxin-3 OS=Rattus norvegicus OX=10116 GN=Stx3 PE=1 SV=1 - [STX3_RAT]	33.2	1.436E7	4.817E6	0.000E0	2.290E6	217.70	54.84		42.56	12.46	7.27		7.61	4	2		2	7	3		2
Q09167	Serine/arginine-rich splicing factor 5 OS=Rattus norvegicus OX=10116 GN=Srsf5 PE=1 SV=1 - [SRSF5_RAT]	30.9	0.000E0	0.000E0	5.903E7	5.766E7			180.89	246.99			14.13	21.19			3	5			5	9
Q10728	Protein phosphatase 1 regulatory subunit 12A OS=Rattus norvegicus GN=Ppp1r12a PE=1 SV=2 - [MYPT1_RAT]	115.2	0.000E0	3.330E7	6.315E7	3.701E7		73.17	133.94	37.31		3.88	6.69	3.39		4	6	4		6	11	7
Q1JU68	Eukaryotic translation initiation factor 3 subunit A OS=Rattus norvegicus GN=Eif3a PE=2 SV=2 - [EIF3A_RAT]	163.1	2.247E6	1.092E7	2.329E7	8.349E6	29.15	151.02	392.67	138.41	1.11	6.57	8.71	4.58	2	7	9	5	3	10	18	8
Q2A865	Sodium-dependent neutral amino acid transporter B(0)AT1 OS=Rattus norvegicus OX=10116 GN=Slc6a19 PE=1 SV=2 - [S	71.2	1.064E7	0.000E0	0.000E0	0.000E0	38.77				3.15				2				2			
Q2LAP6	Testin OS=Rattus norvegicus GN=Tes PE=1 SV=1 - [TES_RAT]	47.6	0.000E0	1.065E7	2.449E7	1.615E7		156.00	290.13	216.36		10.26	21.24	12.65		3	6	4		7	11	9
Q32PX2	Aminoacyl tRNA synthase complex-interacting multifunctional protein 2 OS=Rattus norvegicus GN=Aimp2 PE=2 SV=1 - [A	35.4	0.000E0	0.000E0	8.113E6	4.793E6			137.82	27.54			14.37	2.81			3	1			5	1
Q3B7D1	Ubiquitin-conjugating enzyme E2 Z OS=Rattus norvegicus GN=Ube2z PE=2 SV=2 - [UBE2Z_RAT]	38.3	0.000E0	0.000E0	6.377E6	1.784E6			114.53	45.65			7.02	5.06			3	2			4	3
Q3KR86	MICOS complex subunit Mic60 (Fragment) OS=Rattus norvegicus GN=Immt PE=1 SV=1 - [MIC60_RAT]	67.1	2.640E7	1.624E7	1.163E7	6.297E6	567.14	396.05	382.58	236.96	11.17	11.33	7.06	7.06	5	5	3	3	12	8	6	4
Q3KRC6	Volume-regulated anion channel subunit LRRC8E OS=Rattus norvegicus OX=10116 GN=Lrrc8e PE=1 SV=1 - [LRC8E_RAT]	90.4	4.276E7	0.000E0	0.000E0	0.000E0	23.84				3.52				1				1			
Q3T1K5	F-actin-capping protein subunit alpha-2 OS=Rattus norvegicus GN=Capza2 PE=1 SV=1 - [CAZA2_RAT]	32.9	5.009E6	6.125E7	9.148E7	3.820E7	33.83	557.18	865.91	728.63	9.79	58.74	65.38	54.20	2	10	11	9	2	16	24	21
Q3T1L0	Aldehyde dehydrogenase family 16 member A1 OS=Rattus norvegicus GN=Aldh16a1 PE=2 SV=1 - [A16A1_RAT]	85.4	0.000E0	7.898E6	1.837E7	6.709E6		374.45	560.54	351.18		6.23	16.83	10.72		4	8	6		7	15	10
Q498M4	WD repeat-containing protein 5 OS=Rattus norvegicus GN=Wdr5 PE=1 SV=1 - [WDR5_RAT]	36.6	0.000E0	1.745E7	2.796E7	4.533E6		89.20	80.00	27.27		9.88	5.69	7.78		3	2	2		4	3	2
Q4FZT9	26S proteasome non-ATPase regulatory subunit 2 OS=Rattus norvegicus GN=Psmd2 PE=2 SV=1 - [PSMD2_RAT]	100.1	0.000E0	1.680E7	3.688E7	1.793E7		352.38	755.56	468.55		15.97	26.10	18.94		11	17	13		19	36	24
Q4FZV0	Beta-mannosidase OS=Rattus norvegicus GN=Manba PE=2 SV=1 - [MANBA_RAT]	101.2	7.097E6	0.000E0	0.000E0	0.000E0	145.46				6.13				6				11			
Q4KLH4	Paraspeckle component 1 OS=Rattus norvegicus OX=10116 GN=Pspc1 PE=1 SV=1 - [PSPC1_RAT]	58.7	0.000E0	6.630E6	8.968E6	9.195E6		21.98	75.20	79.82		4.41	8.05	7.47		2	4	4		3	4	6
Q4KLJ8	Phosducin-like protein 3 OS=Rattus norvegicus GN=Pdcl3 PE=1 SV=1 - [PDCL3_RAT]	27.7	0.000E0	1.539E7	3.509E7	8.360E6		64.56	69.22	50.20		9.58	12.92	11.25		2	3	3		2	5	5
Q4KLN6	Ribonucleoside-diphosphate reductase subunit M2 OS=Rattus norvegicus GN=Rrm2 PE=2 SV=1 - [RIR2_RAT]	45.0	7.238E6	0.000E0	0.000E0	0.000E0	72.35				12.05				4				5			
Q4KLZ6	Triokinase/FMN cyclase OS=Rattus norvegicus GN=Tkfc PE=1 SV=1 - [TKFC_RAT]	59.4	6.473E6	2.923E7	4.014E7	6.825E6	147.64	362.91	365.18	92.63	18.86	35.29	25.95	3.46	7	12	9	2	8	21	19	3
Q4KM35	Proteasome subunit beta type-10 OS=Rattus norvegicus GN=Psmb10 PE=2 SV=1 - [PSB10_RAT]	29.0	1.604E6	9.472E6	1.376E7	0.000E0	37.24	173.57	172.75		12.45	20.15	20.15		2	4	4		2	6	7	
Q4KM62	Palmdelphin OS=Rattus norvegicus GN=Palmd PE=1 SV=1 - [PALMD_RAT]	62.4	5.696E6	0.000E0	0.000E0	0.000E0	33.74				1.45				1				2			
Q4KM65	Cleavage and polyadenylation specificity factor subunit 5 OS=Rattus norvegicus GN=Nudt21 PE=2 SV=1 - [CPSF5_RAT]	26.2	8.098E6	1.646E7	2.276E7	1.980E7	83.54	100.12	160.85	147.30	23.35	23.35	30.40	43.17	3	3	5	7	5	7	10	10
Q4KMA2	UV excision repair protein RAD23 homolog B OS=Rattus norvegicus GN=Rad23b PE=1 SV=1 - [RD23B_RAT]	43.5	1.420E7	0.000E0	0.000E0	0.000E0	115.70				9.16				4				9			
Q4V7C7	Actin-related protein 3 OS=Rattus norvegicus GN=Actr3 PE=1 SV=1 - [ARP3_RAT]	47.3	0.000E0	2.969E7	6.190E7	3.411E7		232.40	645.12	536.00		31.10	56.46	38.04		9	15	11		16	30	21
Q4V8H8	EH domain-containing protein 2 OS=Rattus norvegicus GN=Ehd2 PE=1 SV=1 - [EHD2_RAT]	61.2	0.000E0	0.000E0	1.778E7	6.250E7			53.63	49.58			3.31	3.31			2	2			3	4
Q4V8K2	Beta-catenin-like protein 1 OS=Rattus norvegicus GN=Ctnnbl1 PE=1 SV=1 - [CTBL1_RAT]	64.9	0.000E0	7.816E6	1.339E7	5.778E6		65.83	80.41	74.53		2.66	2.66	2.66		2	2	2		4	4	3

Q562B5	Serine/threonine-protein phosphatase PGAM5, mitochondrial OS=Rattus norvegicus OX=10116 GN=Pgam5 PE=2 SV=1 - [	32.0	4.200E6	0.000E0	0.000E0	0.000E0	45.54			26.46	3.13			3.47	1			1	2			1
Q562C4	Methyltransferase-like protein 7B OS=Rattus norvegicus OX=10116 GN=Mettl7b PE=1 SV=1 - [MET7B_RAT]	27.9	0.000E0	6.283E6	9.804E6	4.283E6		39.79	85.61	24.39		15.98	26.23	7.79		2	5	2		3	6	3
Q562C9	1,2-dihydroxy-3-keto-5-methylthiopentene dioxygenase OS=Rattus norvegicus OX=10116 GN=Adi1 PE=2 SV=1 - [MTND_	21.4	2.119E7	0.000E0	0.000E0	0.000E0	89.64				41.34				6				10			
Q566Q8	UPF0696 protein C11orf68 homolog OS=Rattus norvegicus OX=10116 GN=Bles03 PE=1 SV=2 - [CK068_RAT]	31.4	0.000E0	0.000E0	8.202E6	5.604E6			117.93	130.69			10.27	14.73			2	3			3	4
Q568Z6	IST1 homolog OS=Rattus norvegicus GN=Ist1 PE=2 SV=1 - [IST1_RAT]	39.9	0.000E0	3.450E6	7.003E6	6.613E6		56.22	161.29	204.63		9.56	12.30	12.30		3	4	4		5	7	7
Q5BJQ6	Cleavage stimulation factor subunit 1 OS=Rattus norvegicus GN=Cstf1 PE=2 SV=1 - [CSTF1_RAT]	48.4	0.000E0	8.313E6	1.145E7	3.392E6		90.82	118.70	85.17		6.96	11.83	11.83		2	3	3		3	5	4
Q5BK06	Cyclin-D1-binding protein 1 OS=Rattus norvegicus OX=10116 GN=Ccndbp1 PE=2 SV=1 - [CCDB1_RAT]	39.0	9.805E5	0.000E0	0.000E0	0.000E0	36.98				1.97				1				1			
Q5EGY4	Synaptobrevin homolog YKT6 OS=Rattus norvegicus GN=Ykt6 PE=1 SV=1 - [YKT6_RAT]	22.4	3.266E6	0.000E0	0.000E0	0.000E0	27.98				4.04				1				1			
Q5EZ72	Ectonucleotide pyrophosphatase/phosphodiesterase family member 7 OS=Rattus norvegicus OX=10116 GN=Enpp7 PE=1	49.8	3.902E6	0.000E0	0.000E0	0.000E0	31.83				2.28				1				1			
Q5HZW7	Placenta-expressed transcript 1 protein OS=Rattus norvegicus OX=10116 GN=Plet1 PE=2 SV=1 - [PLET1_RAT]	26.8	3.596E7	0.000E0	0.000E0	0.000E0	80.41				6.67				2				4			
Q5HZY2	GTP-binding protein SAR1b OS=Rattus norvegicus GN=Sar1b PE=2 SV=1 - [SAR1B_RAT]	22.4	1.824E7	0.000E0	0.000E0	0.000E0	90.24				25.76				3				4			
Q5I0G4	Glycine--tRNA ligase (Fragment) OS=Rattus norvegicus GN=Gars PE=1 SV=1 - [SYG_RAT]	72.0	0.000E0	1.359E7	2.691E7	1.071E7		171.80	405.35	260.26		14.91	21.51	11.46		7	12	6		11	21	11
Q5M827	Pirin OS=Rattus norvegicus GN=Pir PE=1 SV=1 - [PIR_RAT]	32.2	0.000E0	1.425E7	2.957E7	1.183E7		62.44	233.36	85.59		16.84	16.84	12.71		4	4	3		7	11	7
Q5PPJ4	Deoxyhypusine hydroxylase OS=Rattus norvegicus OX=10116 GN=Dohh PE=2 SV=1 - [DOHH_RAT]	33.1	0.000E0	3.015E6	4.466E6	0.000E0		20.19	44.74			6.62	7.95			2	2			2	2	
Q5RJR8	Leucine-rich repeat-containing protein 59 OS=Rattus norvegicus GN=Lrrc59 PE=1 SV=1 - [LRC59_RAT]	34.8	0.000E0	7.755E6	1.145E7	3.372E6		61.88	134.34	61.95		16.61	19.87	13.36		4	5	4		4	8	5
Q5RKH0	Putative oxidoreductase GLYR1 OS=Rattus norvegicus OX=10116 GN=Glyr1 PE=1 SV=1 - [GLYR1_RAT]	60.4	0.000E0	3.548E6	1.194E7	4.947E6		35.85	83.40	30.89		4.35	3.08	4.71		2	1	2		2	2	3
Q5RKH7	Solute carrier family 35 member F6 OS=Rattus norvegicus OX=10116 GN=Slc35f6 PE=2 SV=1 - [S35F6_RAT]	41.1	2.941E6	0.000E0	0.000E0	0.000E0	55.10				6.45				2				2			
Q5U216	ATP-dependent RNA helicase DDX39A OS=Rattus norvegicus GN=Ddx39a PE=2 SV=1 - [DX39A_RAT]	49.1	2.915E7	7.554E7	1.328E8	1.104E8	237.85	491.21	824.04	815.71	25.29	30.44	37.47	41.92	9	11	14	15	14	28	37	40
Q5U2V4	Phospholipase B-like 1 OS=Rattus norvegicus OX=10116 GN=Plbd1 PE=2 SV=1 - [PLBL1_RAT]	63.0	1.921E7	1.122E7	7.737E6	6.059E6	349.99	271.82	183.44	165.03	14.55	11.27	9.27	10.36	7	5	4	4	15	10	5	7
Q5U2Z3	Nucleosome assembly protein 1-like 4 OS=Rattus norvegicus GN=Nap1l4 PE=1 SV=1 - [NP1L4_RAT]	43.9	0.000E0	0.000E0	7.836E6	5.727E6			149.25	69.19			17.36	7.77			4	3			6	5
Q5XI29	Cleavage and polyadenylation specificity factor subunit 7 OS=Rattus norvegicus OX=10116 GN=Cpsf7 PE=1 SV=1 - [CPSF	51.0	0.000E0	3.950E6	6.139E6	0.000E0		43.51	20.99			4.11	1.73			2	1			3	1	
Q5XI96	Ribonuclease H2 subunit B OS=Rattus norvegicus GN=Rnaseh2b PE=2 SV=1 - [RNH2B_RAT]	34.6	0.000E0	0.000E0	1.776E6	2.015E6			0.00	14.59			3.58	3.58			1	1			2	1
Q5XIE3	39S ribosomal protein L11, mitochondrial OS=Rattus norvegicus OX=10116 GN=Mrpl11 PE=2 SV=1 - [RM11_RAT]	22.4	0.000E0	0.000E0	3.427E6	3.104E6			28.08	41.06			4.43	4.43			1	1			1	2
Q5XIG6	N-acetylgalactosamine kinase OS=Rattus norvegicus OX=10116 GN=Galk2 PE=2 SV=1 - [GALK2_RAT]	50.2	0.000E0	0.000E0	6.648E6	5.015E6			69.52	40.21			5.46	5.46			2	2			3	4
Q5XIM7	Lysine--tRNA ligase OS=Rattus norvegicus OX=10116 GN=Kars PE=1 SV=1 - [SYK_RAT]	71.6	0.000E0	5.776E7	7.940E7	2.345E7		337.80	491.09	400.19		14.70	28.12	26.20		8	13	12		15	23	26
Q5XIM9	T-complex protein 1 subunit beta OS=Rattus norvegicus GN=Cct2 PE=1 SV=3 - [TCPB_RAT]	57.4	1.129E7	2.434E7	6.026E7	2.142E7	161.18	355.52	1196.02	712.80	9.53	28.97	43.55	33.27	4	10	19	14	6	16	43	30
Q62698	Cytoplasmic dynein 1 light intermediate chain 2 OS=Rattus norvegicus GN=Dync1li2 PE=1 SV=1 - [DC1L2_RAT]	54.7	0.000E0	3.916E6	7.927E6	0.000E0		21.68	74.19			4.43	4.43			2	2			2	3	
Q62745	CD81 antigen OS=Rattus norvegicus GN=Cd81 PE=1 SV=1 - [CD81_RAT]	25.9	0.000E0	0.000E0	1.654E7	1.028E7			20.30	39.52			3.39	3.39			1	1			1	2
Q62780	Probable ATP-dependent RNA helicase DDX46 OS=Rattus norvegicus OX=10116 GN=Ddx46 PE=1 SV=1 - [DDX46_RAT]	117.3	0.000E0	0.000E0	1.246E6	1.801E6			20.99	35.46			0.97	3.20			1	3			1	3
Q62786	Prostaglandin F2 receptor negative regulator OS=Rattus norvegicus GN=Ptgfrn PE=1 SV=1 - [FPRP_RAT]	98.7	4.836E6	0.000E0	0.000E0	0.000E0	84.98				2.73				2				3			
Q62839	Golgin subfamily A member 2 OS=Rattus norvegicus OX=10116 GN=Golga2 PE=1 SV=4 - [GOGA2_RAT]	112.8	9.396E5	0.000E0	0.000E0	0.000E0	21.68				1.50				1				1			
Q62847	Gamma-adducin OS=Rattus norvegicus GN=Add3 PE=1 SV=2 - [ADDG_RAT]	78.8	0.000E0	4.613E6	7.498E6	4.817E6		27.16	52.57	48.98		1.42	1.42	3.69		1	1	2		1	2	3
Q62848	ADP-ribosylation factor GTPase-activating protein 1 OS=Rattus norvegicus GN=Arfgap1 PE=1 SV=1 - [ARFG1_RAT]	45.4	1.649E6	0.000E0	0.000E0	0.000E0	30.45				6.75				2				2			
Q62915	Peripheral plasma membrane protein CASK OS=Rattus norvegicus GN=Cask PE=1 SV=1 - [CSKP_RAT]	103.2	1.226E6	0.000E0	0.000E0	0.000E0	28.02				0.77				1				2			
Q62952	Dihydropyrimidinase-related protein 3 OS=Rattus norvegicus GN=Dpysl3 PE=1 SV=2 - [DPYL3_RAT]	61.9	0.000E0	3.490E7	5.725E7	2.368E7		861.56	1033.22	643.70		28.77	42.11	36.67		12	16	14		28	35	26
Q62975	Protein Z-dependent protease inhibitor OS=Rattus norvegicus GN=Serpina10 PE=2 SV=2 - [ZPI_RAT]	50.2	1.136E7	0.000E0	0.000E0	0.000E0	304.08				11.01				4				8			
Q63060	Glycerol kinase OS=Rattus norvegicus OX=10116 GN=Gk PE=2 SV=1 - [GLPK_RAT]	57.4	0.000E0	8.672E6	1.393E7	4.908E6		103.49	145.94	48.32		14.69	17.37	7.44		6	7	3		9	10	4
Q63083	Nucleobindin-1 OS=Rattus norvegicus OX=10116 GN=Nucb1 PE=1 SV=1 - [NUCB1_RAT]	53.5	0.000E0	0.000E0	6.859E6	5.310E6			143.34	123.15			8.71	7.63			3	3			7	6
Q63347	26S protease regulatory subunit 7 OS=Rattus norvegicus GN=Psmc2 PE=1 SV=3 - [PRS7_RAT]	48.5	0.000E0	0.000E0	4.973E6	3.727E6			68.48	95.11			9.93	8.31			3	3			4	5
Q63362	NADH dehydrogenase [ubiquinone] 1 alpha subcomplex subunit 5 OS=Rattus norvegicus OX=10116 GN=Ndufa5 PE=1 SV	13.4	2.612E6	0.000E0	0.000E0	0.000E0	52.85				17.24				1				2			
Q63413	Spliceosome RNA helicase Ddx39b OS=Rattus norvegicus GN=Ddx39b PE=1 SV=3 - [DX39B_RAT]	49.0	3.191E7	7.850E7	1.328E8	1.104E8	607.73	979.18	1236.15	1318.44	39.02	45.79	48.36	48.36	14	16	18	18	24	46	52	55
Q63569	26S protease regulatory subunit 6A OS=Rattus norvegicus GN=Psmc3 PE=2 SV=1 - [PRS6A_RAT]	49.1	0.000E0	1.167E7	4.175E7	6.312E6		191.69	352.09	232.36		15.03	29.61	12.07		5	10	4		8	18	6
Q63570	26S protease regulatory subunit 6B OS=Rattus norvegicus GN=Psmc4 PE=1 SV=1 - [PRS6B_RAT]	47.4	0.000E0	0.000E0	1.084E7	5.582E6			78.74	56.14			9.09	10.53			3	4			5	4
Q63644	Rho-associated protein kinase 1 OS=Rattus norvegicus GN=Rock1 PE=1 SV=1 - [ROCK1_RAT]	159.5	0.000E0	0.000E0	1.473E6	0.000E0	23.73	26.40	16.32		0.73	0.73	0.73		1	1	1		1	1	1	
Q63661	Mucin-4 OS=Rattus norvegicus GN=Muc4 PE=1 SV=2 - [MUC4_RAT]	247.9	1.067E7	0.000E0	0.000E0	0.000E0	144.43				1.71				4				7			
Q64057	Alpha-aminoadipic semialdehyde dehydrogenase OS=Rattus norvegicus GN=Aldh7a1 PE=1 SV=2 - [AL7A1_RAT]	58.7	0.000E0	2.954E7	5.374E7	1.700E7		419.27	823.55	490.14		33.77	45.27	39.52		11	16	14		22	30	25
Q64060	Probable ATP-dependent RNA helicase DDX4 OS=Rattus norvegicus OX=10116 GN=Ddx4 PE=1 SV=1 - [DDX4_RAT]	77.9	0.000E0	0.000E0	3.071E6	2.385E6			25.89	26.78			1.68	1.68			1	1			2	1
Q641X8	Eukaryotic translation initiation factor 3 subunit E OS=Rattus norvegicus GN=Eif3e PE=2 SV=1 - [EIF3E_RAT]	52.2	0.000E0	5.273E6	1.807E7	8.435E6		55.27	330.13	91.93		8.54	19.78	15.06		4	7	6		7	12	9
Q641Z6	EH domain-containing protein 1 OS=Rattus norvegicus GN=Ehd1 PE=1 SV=1 - [EHD1_RAT]	60.6	0.000E0	0.000E0	1.066E7	5.186E6			59.62	44.65			4.49	7.68			2	3			3	5
Q64268	Heparin cofactor 2 OS=Rattus norvegicus GN=Serpind1 PE=1 SV=1 - [HEP2_RAT]	54.5	5.014E6	0.000E0	0.000E0	0.000E0	37.11				4.18				2				3			
Q64350	Translation initiation factor eIF-2B subunit epsilon OS=Rattus norvegicus GN=Eif2b5 PE=1 SV=2 - [EI2BE_RAT]	80.2	0.000E0	6.677E6	8.970E6	0.000E0		58.80	36.82			6.28	2.51			3	2			4	2	
Q64428	Trifunctional enzyme subunit alpha, mitochondrial OS=Rattus norvegicus GN=Hadha PE=1 SV=2 - [ECHA_RAT]	82.6	0.000E0	3.965E6	1.157E7	4.115E6		53.11	95.38	85.23		3.01	18.87	9.57		2	8	6		3	12	8
Q64560	Tripeptidyl-peptidase 2 OS=Rattus norvegicus GN=Tpp2 PE=2 SV=3 - [TPP2_RAT]	138.2	0.000E0	5.397E6	9.992E6	4.207E6		56.70	162.44	73.58		2.56	8.33	7.05		2	7	6		3	9	8
Q64591	2,4-dienoyl-CoA reductase, mitochondrial OS=Rattus norvegicus GN=Decr1 PE=1 SV=2 - [DECR_RAT]	36.1	0.000E0	0.000E0	2.587E7	8.902E6			25.90	127.38			6.57	13.13			1	3			1	5
Q66H61	Glutamine--tRNA ligase OS=Rattus norvegicus OX=10116 GN=Qars PE=1 SV=1 - [SYQ_RAT]	87.6	0.000E0	1.544E7	2.501E7	1.113E7		506.13	462.87	358.55		27.61	23.61	24.52		16	12	12		25	21	17
Q66H68	RNA-binding protein 47 OS=Rattus norvegicus GN=Rbm47 PE=2 SV=1 - [RBM47_RAT]	64.1	0.000E0	2.165E7	4.031E7	1.129E7		83.39	166.00	149.64		9.32	15.25	13.39		5	7	6		10	13	10
Q66HD0	Endoplasmin OS=Rattus norvegicus GN=Hsp90b1 PE=1 SV=2 - [ENPL_RAT]	92.7	1.392E8	2.866E8	4.235E8	2.109E8	511.63	1138.48	2109.64	1893.29	10.32	24.38	44.53	45.27	8	19	34	36	15	46	87	81
Q66HD3	Nuclear autoantigenic sperm protein OS=Rattus norvegicus GN=Nasp PE=1 SV=1 - [NASP_RAT]	84.1	0.000E0	0.000E0	1.570E7	4.199E6			87.52	39.03			5.80	4.51			3	2			5	4
Q66X93	Staphylococcal nuclease domain-containing protein 1 OS=Rattus norvegicus GN=Snd1 PE=2 SV=1 - [SND1_RAT]	101.9	0.000E0	3.146E7	4.519E7	2.020E7		529.81	607.26	432.96		41.36	37.51	33.22		23	23	20		38	40	35
Q68FQ0	T-complex protein 1 subunit epsilon OS=Rattus norvegicus GN=Cct5 PE=1 SV=1 - [TCPE_RAT]	59.5	4.734E6	1.728E7	5.131E7	2.035E7	147.56	266.08	832.79	304.25	4.25	11.09	40.11	17.38	3	6	17	8	5	9	38	14
Q68FT1	Ubiquinone biosynthesis protein COQ9, mitochondrial OS=Rattus norvegicus OX=10116 GN=Coq9 PE=1 SV=2 - [COQ9_R	35.1	1.112E7	4.396E6	0.000E0	0.000E0	85.72	32.34			6.41	9.62			2	3			3	3		
Q68FT3	Pyridine nucleotide-disulfide oxidoreductase domain-containing protein 2 OS=Rattus norvegicus OX=10116 GN=Pyroxd2 P	62.8	3.643E6	0.000E0	0.000E0	0.000E0	36.94				2.24				1				1			
Q68FU3	Electron transfer flavoprotein subunit beta OS=Rattus norvegicus GN=Etfb PE=2 SV=3 - [ETFB_RAT]	27.7	0.000E0	8.569E7	1.185E8	5.323E7		1037.28	1329.90	990.38		49.02	49.02	45.49		11	11	10		27	35	27
Q68FV6	Glycosylated lysosomal membrane protein OS=Rattus norvegicus OX=10116 GN=Glmp PE=2 SV=1 - [GLMP_RAT]	43.8	1.970E7	7.871E6	0.000E0	0.000E0	114.64	65.41			4.95	4.95			1	1			2	1		
Q68FX0	Isocitrate dehydrogenase [NAD] subunit beta, mitochondrial OS=Rattus norvegicus GN=Idh3B PE=1 SV=1 - [IDH3B_RAT]	42.3	0.000E0	0.000E0	7.690E6	4.815E6			49.20	60.02			4.16	4.16			1	1			2	2
Q6AYD3	Proliferation-associated protein 2G4 OS=Rattus norvegicus GN=Pa2g4 PE=1 SV=1 - [PA2G4_RAT]	43.6	1.068E7	7.937E7	1.151E8	5.679E7	160.08	1030.94	977.66	723.60	19.04	55.33	54.82	55.33	5	17	17	17	8	36	41	30
Q6AYK1	RNA-binding protein with serine-rich domain 1 OS=Rattus norvegicus OX=10116 GN=Rnps1 PE=2 SV=1 - [RNPS1_RAT]	34.2	0.000E0	8.215E6	1.331E7	1.088E7		79.77	189.51	128.64		4.92	11.48	11.48		1	3	3		2	6	6
Q6AYQ2	Aldo-keto reductase family 1 member C21 OS=Rattus norvegicus GN=Akr1c21 PE=2 SV=1 - [AK1CL_RAT]	36.1	1.013E7	3.421E7	5.826E7	2.487E7	147.04	138.12	97.77	141.98	8.18	8.18	7.86	8.18	4	3	3	3	7	8	6	8
Q6AYR5	Clarin-3 OS=Rattus norvegicus OX=10116 GN=Clrn3 PE=2 SV=1 - [CLRN3_RAT]	25.3	1.029E7	0.000E0	0.000E0	0.000E0	69.23				5.31				1				2			
Q6AYS4	Plasma alpha-L-fucosidase OS=Rattus norvegicus OX=10116 GN=Fuca2 PE=2 SV=1 - [FUCO2_RAT]	53.2	3.521E6	8.842E6	1.190E7	8.521E6	36.16	75.97	41.84	44.32	6.97	15.47	15.69	10.24	3	6	6	4	5	8	9	6
Q6AYT3	tRNA-splicing ligase RtcB homolog OS=Rattus norvegicus GN=Rtcb PE=2 SV=1 - [RTCB_RAT]	55.2	0.000E0	2.716E7	3.976E7	1.455E7		398.52	396.51	309.02		28.71	30.30	22.97		11	11	9		17	17	15
Q6BBI8	Ubiquitin-fold modifier-conjugating enzyme 1 OS=Rattus norvegicus GN=Ufc1 PE=2 SV=1 - [UFC1_RAT]	19.5	0.000E0	6.425E6	1.419E7	0.000E0		21.04	20.71			4.79	10.18			1	2			1	2	
Q6GQP4	Ras-related protein Rab-31 OS=Rattus norvegicus GN=Rab31 PE=1 SV=2 - [RAB31_RAT]	21.4	8.200E6	0.000E0	0.000E0	0.000E0	26.37				5.67				1				1			
Q6IMY8	Heterogeneous nuclear ribonucleoprotein U OS=Rattus norvegicus OX=10116 GN=Hnrnpu PE=1 SV=1 - [HNRPU_RAT]	87.7	4.649E6	1.054E7	3.228E7	8.977E7	25.50	94.01	278.82	1087.25	1.63	4.64	12.66	28.32	2	2	8	19	4	3	13	48
Q6IUU3	Sulfhydryl oxidase 1 OS=Rattus norvegicus GN=Qsox1 PE=1 SV=1 - [QSOX1_RAT]	82.4	2.099E6	0.000E0	0.000E0	0.000E0	22.12				1.47				1				2			
Q6JE36	Protein NDRG1 OS=Rattus norvegicus GN=Ndrg1 PE=1 SV=1 - [NDRG1_RAT]	42.9	0.000E0	0.000E0	2.498E7	9.259E6			24.38	55.44			4.31	16.50			1	4			2	7
Q6MG08	ATP-binding cassette sub-family F member 1 OS=Rattus norvegicus OX=10116 GN=Abcf1 PE=1 SV=1 - [ABCF1_RAT]	95.2	0.000E0	0.000E0	1.507E6	2.404E6			43.52	37.06			1.19	1.19			1	1			1	2
Q6P502	T-complex protein 1 subunit gamma OS=Rattus norvegicus GN=Cct3 PE=1 SV=1 - [TCPG_RAT]	60.6	0.000E0	1.153E7	6.071E7	2.338E7		368.33	792.12	551.44		11.38	44.40	30.64		6	17	13		12	30	22
Q6P6T4	Echinoderm microtubule-associated protein-like 2 OS=Rattus norvegicus GN=Eml2 PE=1 SV=1 - [EMAL2_RAT]	70.7	0.000E0	5.455E6	1.230E7	3.716E6		92.71	257.28	123.04		7.24	18.03	12.02		4	8	6		5	12	10
Q6P767	Pituitary tumor-transforming gene 1 protein-interacting protein OS=Rattus norvegicus GN=Pttg1ip PE=2 SV=1 - [PTTG_R	19.9	1.369E7	0.000E0	0.000E0	5.790E6	54.15			35.69	4.60			4.60	1			1	2			1
Q6P799	Serine--tRNA ligase, cytoplasmic OS=Rattus norvegicus GN=Sars PE=1 SV=3 - [SYSC_RAT]	58.6	0.000E0	4.902E7	6.611E7	2.927E7		620.13	748.57	508.23		36.13	37.89	28.52		13	14	12		24	28	24
Q6P7B0	Tryptophan--tRNA ligase, cytoplasmic OS=Rattus norvegicus GN=Wars PE=1 SV=2 - [SYWC_RAT]	54.1	0.000E0	0.000E0	1.063E7	6.827E6			25.58	23.95			7.07	3.33			2	1			3	1
Q6P9U8	Eukaryotic translation initiation factor 3 subunit H OS=Rattus norvegicus GN=Eif3h PE=1 SV=1 - [EIF3H_RAT]	39.9	0.000E0	4.902E6	1.493E7	3.567E6		70.10	160.25	32.78		7.10	14.20	3.13		2	3	1		3	6	2
Q6PDU1	Serine/arginine-rich splicing factor 2 OS=Rattus norvegicus GN=Srsf2 PE=1 SV=3 - [SRSF2_RAT]	25.5	9.839E6	2.373E7	6.379E7	6.341E7	79.64	120.62	388.01	372.72	3.62	11.76	19.46	19.46	1	3	4	4	2	5	10	12
Q6PDV7	60S ribosomal protein L10 OS=Rattus norvegicus GN=Rpl10 PE=1 SV=3 - [RL10_RAT]	24.6	0.000E0	1.259E7	2.829E7	1.921E7		113.56	286.30	143.58		26.17	38.32	28.50		4	6	5		7	12	12
Q6Q0N1	Cytosolic non-specific dipeptidase OS=Rattus norvegicus GN=Cndp2 PE=1 SV=1 - [CNDP2_RAT]	52.7	3.608E7	2.473E8	3.869E8	1.695E8	405.36	1822.14	3067.26	1935.17	21.05	62.11	81.26	62.11	7	25	29	25	13	85	113	87
Q6RY07	Acidic mammalian chitinase OS=Rattus norvegicus OX=10116 GN=Chia PE=2 SV=1 - [CHIA_RAT]	51.9	5.966E6	4.804E7	1.294E8	1.160E8	34.58	312.00	790.20	872.73	1.90	27.27	38.90	35.73	1	9	11	10	2	17	32	32
Q6T3U3	NPC1-like intracellular cholesterol transporter 1 OS=Rattus norvegicus OX=10116 GN=Npc1l1 PE=1 SV=1 - [NPCL1_RAT]	146.3	4.673E6	0.000E0	0.000E0	0.000E0	101.75				2.03				3				4			
Q7M0E3	Destrin OS=Rattus norvegicus GN=Dstn PE=1 SV=3 - [DEST_RAT]	18.5	7.971E6	1.860E8	3.084E8	2.085E8	169.04	1336.91	1502.40	1546.78	30.30	75.76	75.15	75.15	5	16	15	15	8	51	58	59
Q7TP17	Splicing factor U2AF 26 kDa subunit OS=Rattus norvegicus OX=10116 GN=U2af1l4 PE=2 SV=1 - [U2AF4_RAT]	25.8	0.000E0	3.757E6	7.574E6	8.074E6		32.81	61.71	87.41		4.09	4.09	7.73		1	1	2		1	2	4
Q7TPB1	T-complex protein 1 subunit delta OS=Rattus norvegicus GN=Cct4 PE=1 SV=3 - [TCPD_RAT]	58.1	0.000E0	1.580E7	4.160E7	1.706E7		161.70	874.52	308.19		11.69	42.30	32.28		5	16	12		10	36	23
Q7TQ94	Deaminated glutathione amidase OS=Rattus norvegicus OX=10116 GN=Nit1 PE=2 SV=2 - [NIT1_RAT]	36.1	0.000E0	1.390E7	1.912E7	9.269E6		202.37	278.74	163.25		28.75	41.59	24.77		7	9	6		13	15	9
Q7TQM5	Keratinocyte proline-rich protein OS=Rattus norvegicus OX=10116 GN=Kprp PE=2 SV=1 - [KPRP_RAT]	76.3	1.810E7	0.000E0	0.000E0	0.000E0	63.94				1.43				1				2			
Q80U96	Exportin-1 OS=Rattus norvegicus GN=Xpo1 PE=1 SV=1 - [XPO1_RAT]	123.0	3.556E6	9.634E6	2.137E7	1.314E7	39.70	165.78	516.48	232.74	0.65	8.78	16.06	11.67	1	6	12	9	2	10	24	15
Q811A3	Procollagen-lysine,2-oxoglutarate 5-dioxygenase 2 OS=Rattus norvegicus GN=Plod2 PE=2 SV=1 - [PLOD2_RAT]	84.5	0.000E0	6.024E6	1.013E7	3.719E6		163.43	186.39	65.75		5.43	12.21	1.63		3	6	1		5	8	2
Q811U3	ELKS/Rab6-interacting/CAST family member 1 OS=Rattus norvegicus GN=Erc1 PE=1 SV=1 - [RB6I2_RAT]	108.8	3.404E6	0.000E0	0.000E0	0.000E0	0.00				0.95				1				1			
Q811X6	Lambda-crystallin homolog OS=Rattus norvegicus OX=10116 GN=Cryl1 PE=1 SV=3 - [CRYL1_RAT]	35.3	0.000E0	1.223E7	2.225E7	1.177E7		163.49	312.81	118.41		31.97	34.80	21.63		7	8	5		10	15	8
Q8K1Q0	Glycylpeptide N-tetradecanoyltransferase 1 OS=Rattus norvegicus GN=Nmt1 PE=1 SV=1 - [NMT1_RAT]	56.8	0.000E0	6.151E6	9.202E6	9.895E6		71.84	110.38	40.52		6.65	10.69	3.83		2	3	1		4	4	1
Q8R4A1	ERO1-like protein alpha OS=Rattus norvegicus GN=Ero1l PE=1 SV=1 - [ERO1A_RAT]	54.0	0.000E0	5.938E6	1.030E7	2.981E6		78.14	130.87	45.21		7.33	15.30	5.39		3	6	3		6	8	4
Q8VD91	Ribonuclease pancreatic alpha-type OS=Rattus fuscipes OX=10119 PE=3 SV=1 - [RNS1A_RATFU]	16.6	6.491E6	0.000E0	0.000E0	0.000E0	47.79				16.11				2				4			
Q8VHT6	Arsenite methyltransferase OS=Rattus norvegicus OX=10116 GN=As3mt PE=1 SV=1 - [AS3MT_RAT]	41.0	0.000E0	1.382E7	1.852E7	6.738E6		92.57	79.37	41.59		7.32	17.89	7.32		2	5	2		4	5	3
Q91Y78	Ubiquitin carboxyl-terminal hydrolase isozyme L3 OS=Rattus norvegicus GN=Uchl3 PE=1 SV=1 - [UCHL3_RAT]	26.1	0.000E0	1.228E7	1.651E7	8.217E6		56.60	122.66	29.77		15.65	21.30	8.70		2	3	1		3	5	1
Q91ZN1	Coronin-1A OS=Rattus norvegicus GN=Coro1a PE=1 SV=3 - [COR1A_RAT]	51.0	0.000E0	6.262E6	2.040E7	4.323E6		153.90	278.09	62.69		7.16	26.25	11.28		3	9	4		5	13	4
Q921A4	Cytoglobin OS=Rattus norvegicus OX=10116 GN=Cygb PE=1 SV=1 - [CYGB_RAT]	21.5	0.000E0	0.000E0	9.847E6	7.057E6			30.46	33.18			4.74	4.74			1	1			1	2
Q923J6	Dynein heavy chain 12, axonemal OS=Rattus norvegicus GN=Dnah12 PE=2 SV=2 - [DYH12_RAT]	357.0	2.600E7	0.000E0	0.000E0	0.000E0	25.62				0.23				1				2			
Q99068	Alpha-2-macroglobulin receptor-associated protein OS=Rattus norvegicus OX=10116 GN=Lrpap1 PE=1 SV=2 - [AMRP_RA	42.0	0.000E0	0.000E0	6.425E6	5.015E6			57.93	68.53			3.61	9.44			1	3			2	3
Q99J86	Attractin OS=Rattus norvegicus GN=Atrn PE=2 SV=1 - [ATRN_RAT]	158.6	8.150E6	0.000E0	0.000E0	0.000E0	212.63				3.70				4				7			
Q99NA5	Isocitrate dehydrogenase [NAD] subunit alpha, mitochondrial OS=Rattus norvegicus GN=Idh3a PE=1 SV=1 - [IDH3A_RAT	39.6	0.000E0	5.047E6	1.665E7	9.072E6		26.60	364.56	66.06		2.73	20.22	11.75		1	6	3		1	10	5
Q99PF5	Far upstream element-binding protein 2 OS=Rattus norvegicus GN=Khsrp PE=1 SV=1 - [FUBP2_RAT]	74.2	0.000E0	0.000E0	6.929E6	1.942E7			122.23	318.94			4.72	9.57			3	6			4	12
Q99PS8	Histidine-rich glycoprotein OS=Rattus norvegicus GN=Hrg PE=1 SV=1 - [HRG_RAT]	59.0	2.540E6	0.000E0	0.000E0	0.000E0	25.51				1.90				1				1			
Q9EPB1	Dipeptidyl peptidase 2 OS=Rattus norvegicus GN=Dpp7 PE=1 SV=1 - [DPP2_RAT]	55.1	2.591E7	5.331E7	1.125E8	7.511E7	213.36	610.30	900.06	736.69	13.60	33.20	33.20	27.80	5	10	11	9	12	24	27	28
Q9EQV6	Tripeptidyl-peptidase 1 OS=Rattus norvegicus GN=Tpp1 PE=1 SV=1 - [TPP1_RAT]	61.3	4.777E6	0.000E0	0.000E0	0.000E0	46.19				4.97				2				3			
Q9ER34	Aconitate hydratase, mitochondrial OS=Rattus norvegicus GN=Aco2 PE=1 SV=2 - [ACON_RAT]	85.4	1.775E6	1.822E8	2.491E8	1.185E8	37.94	2789.26	3090.38	1845.75	1.03	59.87	61.41	54.74	1	37	38	32	1	102	105	76
Q9ERB4	Versican core protein (Fragments) OS=Rattus norvegicus GN=Vcan PE=2 SV=2 - [CSPG2_RAT]	299.8	3.342E6	1.009E7	1.692E7	8.114E6	21.16	141.71	233.22	135.47	0.77	1.68	3.65	2.52	2	4	7	6	2	7	10	9
Q9ES40	PRA1 family protein 3 OS=Rattus norvegicus OX=10116 GN=Arl6ip5 PE=1 SV=1 - [PRAF3_RAT]	21.5	2.941E6	0.000E0	0.000E0	0.000E0	24.34				5.85				1				1			
Q9ES54	Nuclear protein localization protein 4 homolog OS=Rattus norvegicus GN=Nploc4 PE=1 SV=3 - [NPL4_RAT]	68.0	0.000E0	6.557E6	1.068E7	5.144E6		42.75	81.26	24.55		2.63	1.64	1.64		2	1	1		3	2	1
Q9ESS6	Basal cell adhesion molecule OS=Rattus norvegicus OX=10116 GN=Bcam PE=2 SV=1 - [BCAM_RAT]	67.5	0.000E0	2.608E6	3.724E6	0.000E0		56.19	30.74			5.29	3.04			2	1			2	1	
Q9ET61	Complement component C1q receptor OS=Rattus norvegicus OX=10116 GN=Cd93 PE=1 SV=1 - [C1QR1_RAT]	68.7	6.014E6	0.000E0	0.000E0	0.000E0	42.69				1.71				1				2			
Q9JHB5	Translin-associated protein X OS=Rattus norvegicus GN=Tsnax PE=1 SV=1 - [TSNAX_RAT]	33.0	0.000E0	0.000E0	7.003E6	6.482E6			46.09	23.42			15.52	6.90			3	1			5	1
Q9JHL4	Drebrin-like protein OS=Rattus norvegicus GN=Dbnl PE=1 SV=1 - [DBNL_RAT]	48.6	0.000E0	7.216E6	9.931E6	2.626E6		115.20	258.54	50.83		8.26	13.30	6.65		3	5	2		3	9	3
Q9JHW0	Proteasome subunit beta type-7 OS=Rattus norvegicus GN=Psmb7 PE=1 SV=1 - [PSB7_RAT]	29.9	0.000E0	8.352E6	1.790E7	3.426E6		53.02	136.91	32.04		11.19	24.55	7.94		3	5	2		4	8	2
Q9JHW1	Carboxypeptidase D OS=Rattus norvegicus GN=Cpd PE=1 SV=2 - [CBPD_RAT]	152.5	1.680E6	0.000E0	0.000E0	0.000E0	33.35				0.73				1				1			
Q9JIX3	Bis(5'-adenosyl)-triphosphatase OS=Rattus norvegicus OX=10116 GN=Fhit PE=1 SV=1 - [FHIT_RAT]	17.3	4.123E6	0.000E0	0.000E0	0.000E0	99.49				8.67				1				2			
Q9JJ09	Sodium-dependent phosphate transport protein 2B OS=Rattus norvegicus OX=10116 GN=Slc34a2 PE=1 SV=1 - [NPT2B_R	75.9	7.390E6	0.000E0	0.000E0	0.000E0	86.62				6.33				3				6			
Q9JJ50	Hepatocyte growth factor-regulated tyrosine kinase substrate OS=Rattus norvegicus GN=Hgs PE=1 SV=1 - [HGS_RAT]	86.2	0.000E0	4.259E6	6.563E6	0.000E0		41.55	45.58			4.38	2.58			2	2			2	4	
Q9JJP9	Ubiquilin-1 OS=Rattus norvegicus GN=Ubqln1 PE=1 SV=1 - [UBQL1_RAT]	62.0	5.227E6	0.000E0	0.000E0	0.000E0	84.70				2.23				1				2			
Q9JKU6	Spermatid perinuclear RNA-binding protein OS=Rattus norvegicus GN=Strbp PE=1 SV=1 - [STRBP_RAT]	74.0	0.000E0	8.392E6	1.196E7	5.606E6		62.21	53.10	24.43		4.92	2.53	4.92		3	2	3		4	2	3
Q9JMB5	Proteasomal ubiquitin receptor ADRM1 OS=Rattus norvegicus GN=Adrm1 PE=2 SV=2 - [ADRM1_RAT]	42.1	0.000E0	5.687E6	1.133E7	3.825E6		49.31	53.77	51.67		3.69	5.90	3.69		2	2	2		3	4	3
Q9QY17	Protein kinase C and casein kinase substrate in neurons 2 protein OS=Rattus norvegicus GN=Pacsin2 PE=1 SV=2 - [PACN	55.9	4.836E7	0.000E0	0.000E0	5.055E6	33.16			37.06	1.43			2.25	1			1	2			1
Q9QYW3	MOB-like protein phocein OS=Rattus norvegicus GN=Mob4 PE=1 SV=1 - [PHOCN_RAT]	26.0	7.505E5	7.527E6	1.053E7	4.311E6	24.24	43.04	107.54	37.85	4.00	10.22	16.44	16.44	1	2	3	3	1	4	5	4
Q9QZA6	CD151 antigen OS=Rattus norvegicus GN=Cd151 PE=1 SV=2 - [CD151_RAT]	28.3	3.439E6	1.565E6	0.000E0	0.000E0	25.35	26.56			3.95	3.95			1	1			2	1		
Q9QZR6	Septin-9 OS=Rattus norvegicus GN=Sept9 PE=1 SV=1 - [SEPT9_RAT]	63.8	0.000E0	0.000E0	6.005E6	3.743E6			86.23	32.17			6.38	1.77			3	1			4	1
Q9R064	Golgi reassembly-stacking protein 2 OS=Rattus norvegicus GN=Gorasp2 PE=1 SV=3 - [GORS2_RAT]	47.2	0.000E0	5.485E6	7.413E6	3.986E6		81.48	117.89	94.68		7.27	8.81	8.81		2	3	3		3	5	4
Q9R0J8	Legumain OS=Rattus norvegicus GN=Lgmn PE=1 SV=1 - [LGMN_RAT]	49.4	0.000E0	0.000E0	2.185E7	2.475E7			101.04	133.80			13.10	13.10			3	3			8	12
Q9WU49	Calcium-regulated heat stable protein 1 OS=Rattus norvegicus GN=Carhsp1 PE=1 SV=1 - [CHSP1_RAT]	15.9	6.421E6	0.000E0	0.000E0	0.000E0	37.63				10.88				1				2			
Q9WVK3	Peroxisomal trans-2-enoyl-CoA reductase OS=Rattus norvegicus OX=10116 GN=Pecr PE=2 SV=1 - [PECR_RAT]	32.4	0.000E0	1.277E7	1.895E7	1.334E7		76.70	220.68	194.53		18.48	30.36	26.73		3	6	5		4	10	8
Q9Z1A5	NEDD8-activating enzyme E1 regulatory subunit OS=Rattus norvegicus GN=Nae1 PE=1 SV=1 - [ULA1_RAT]	60.3	0.000E0	7.828E6	1.100E7	6.975E6		145.94	222.56	127.78		10.30	21.16	15.36		3	6	5		5	9	7
Q9Z1Z3	Epsin-2 OS=Rattus norvegicus GN=Epn2 PE=1 SV=1 - [EPN2_RAT]	62.3	6.266E6	1.756E6	0.000E0	0.000E0	21.64	31.51			1.72	1.72			1	1			2	1		
