# Supplementary material for: Dedifferentiated fat cells administration ameliorates abnormal expressions of fatty acids metabolism-related protein expressions and intestinal tissue damage in experimental necrotizing enterocolitis
Source: Sci Rep. 2023 May 22;13:8266. doi: 10.1038/s41598-023-34156-1 (PMC10203254; doi:10.1038/s41598-023-34156-1)
Supplement: Supplementary file 5 — Supplementary Table S3. [file 41598_2023_34156_MOESM5_ESM.pdf]

Table S3. Dysregulated by NEC and ameliolated by DFAT

Accession	Description	MW [kDa]	Area				Score				Coverage				# Peptides				# PSM			
			sham	vehicle -mild	vehicle -severe	DFAT	sham	vehicle -mild	vehicle -severe	DFAT	sham	vehicle -mild	vehicle -severe	DFAT	sham	vehicle -mild	vehicle -severe	DFAT	sham	vehicle -mild	vehicle -severe	DFAT
A2RUW1	Toll-interacting protein OS=Rattus norvegicus GN=Tollip PE=2 SV=1 - [TOLIP_RAT]	30.3	0.000E0	8.855E6	1.771E7	3.698E6		99.97	57.12	28.77		8.03	4.74	13.14		2	1	3		3	2	4
B0BNA7	Eukaryotic translation initiation factor 3 subunit I OS=Rattus norvegicus GN=Eif3i PE=2 SV=1 - [EIF3I_RAT]	36.4	3.856E6	1.613E7	2.617E7	6.826E6	29.94	195.07	214.23	103.70	5.54	27.38	21.54	8.31	2	6	5	3	2	8	8	5
B2GUZ5	F-actin-capping protein subunit alpha-1 OS=Rattus norvegicus GN=Capza1 PE=1 SV=1 - [CAZA1_RAT]	32.9	1.730E6	7.608E7	1.213E8	4.336E7	27.30	571.07	625.85	389.43	2.80	64.69	64.69	54.20	1	12	12	11	1	24	27	18
B2GV06	Succinyl-CoA:3-ketoacid coenzyme A transferase 1, mitochondrial OS=Rattus norvegicus GN=Oxct1 PE=1 SV=1 - [SCOT1_	56.2	0.000E0	1.341E7	2.364E7	8.697E6		118.06	380.67	105.86		26.15	31.54	13.27		7	9	4		8	16	6
O35077	Glycerol-3-phosphate dehydrogenase [NAD(+)], cytoplasmic OS=Rattus norvegicus GN=Gpd1 PE=1 SV=4 - [GPDA_RAT]	37.4	5.544E6	2.651E7	3.772E7	1.522E7	61.86	237.64	350.99	87.64	3.15	37.82	39.83	29.23	1	10	9	6	1	16	16	9
O35244	Peroxiredoxin-6 OS=Rattus norvegicus GN=Prdx6 PE=1 SV=3 - [PRDX6_RAT]	24.8	6.652E6	6.069E7	9.945E7	3.323E7	120.11	593.29	857.12	466.76	26.34	62.50	70.09	68.75	4	11	12	12	5	22	29	23
O54975	Xaa-Pro aminopeptidase 1 OS=Rattus norvegicus GN=Xpnpep1 PE=1 SV=1 - [XPP1_RAT]	69.6	1.582E7	5.180E7	7.810E7	3.314E7	186.05	983.60	1323.85	703.27	12.52	65.01	65.65	48.15	7	25	26	22	13	48	62	37
O55159	Epithelial cell adhesion molecule OS=Rattus norvegicus OX=10116 GN=Epcam PE=1 SV=1 - [EPCAM_RAT]	35.2	2.766E6	3.325E7	4.666E7	1.209E7	27.95	204.93	455.20	65.21	4.13	40.63	40.63	25.08	1	7	8	5	2	12	16	7
O88202	60 kDa lysophospholipase OS=Rattus norvegicus OX=10116 GN=Aspg PE=1 SV=1 - [LPP60_RAT]	60.8	0.000E0	4.497E6	6.075E6	0.000E0		24.79	38.25			4.08	4.08			2	2			2	3	
O88656	Actin-related protein 2/3 complex subunit 1B OS=Rattus norvegicus GN=Arpc1b PE=2 SV=3 - [ARC1B_RAT]	41.0	5.408E6	2.785E7	3.938E7	1.795E7	67.72	322.41	508.41	434.56	9.14	30.38	36.29	30.65	3	8	11	9	4	12	20	15
P00406	Cytochrome c oxidase subunit 2 OS=Rattus norvegicus OX=10116 GN=Mtco2 PE=1 SV=3 - [COX2_RAT]	25.9	3.644E6	1.204E7	5.046E7	3.586E6	46.82	55.87	65.80	69.73	4.41	7.49	7.49	4.41	1	2	2	1	2	3	3	2
P05197	Elongation factor 2 OS=Rattus norvegicus GN=Eef2 PE=1 SV=4 - [EF2_RAT]	95.2	6.863E7	2.299E8	3.083E8	1.234E8	1519.58	4050.46	4963.99	3331.46	37.53	63.40	60.49	65.85	28	43	42	43	65	162	175	140
P07150	Annexin A1 OS=Rattus norvegicus GN=Anxa1 PE=1 SV=2 - [ANXA1_RAT]	38.8	4.108E6	1.274E7	1.775E7	5.232E6	49.94	209.87	264.55	95.61	5.49	21.10	25.14	12.43	2	6	7	3	2	8	10	4
P07340	Sodium/potassium-transporting ATPase subunit beta-1 OS=Rattus norvegicus OX=10116 GN=Atp1b1 PE=1 SV=1 - [AT1B	35.2	1.287E7	3.077E7	4.815E7	1.694E7	59.76	95.56	161.23	103.06	7.24	10.53	8.88	12.50	2	3	2	3	4	9	6	5
P07756	Carbamoyl-phosphate synthase [ammonia], mitochondrial OS=Rattus norvegicus GN=Cps1 PE=1 SV=1 - [CPSM_RAT]	164.5	2.873E7	7.449E7	1.375E8	3.588E7	707.98	2172.05	3783.71	1449.66	16.73	46.47	48.60	31.93	20	48	53	36	39	105	131	71
P07872	Peroxisomal acyl-coenzyme A oxidase 1 OS=Rattus norvegicus GN=Acox1 PE=1 SV=1 - [ACOX1_RAT]	74.6	0.000E0	3.161E7	4.728E7	2.003E7		731.25	1012.73	669.79		33.89	42.36	37.37		13	17	14		26	32	28
P07943	Aldose reductase OS=Rattus norvegicus GN=Akr1b1 PE=1 SV=3 - [ALDR_RAT]	35.8	1.095E7	6.135E7	8.552E7	3.422E7	164.08	567.45	578.72	382.95	12.66	54.75	57.91	39.87	4	15	15	12	8	34	36	25
P08503	Medium-chain specific acyl-CoA dehydrogenase, mitochondrial OS=Rattus norvegicus GN=Acadm PE=1 SV=1 - [ACADM_R	46.5	0.000E0	3.921E7	6.430E7	1.996E7		391.39	611.29	326.66		33.02	43.47	23.04		10	14	8		16	31	17
P09456	cAMP-dependent protein kinase type I-alpha regulatory subunit OS=Rattus norvegicus GN=Prkar1a PE=2 SV=2 - [KAP0_R	43.1	0.000E0	2.116E7	2.982E7	1.399E7		325.22	456.68	383.57		21.78	26.51	21.78		7	8	7		11	15	13
P11442	Clathrin heavy chain 1 OS=Rattus norvegicus GN=Cltc PE=1 SV=3 - [CLH1_RAT]	191.5	7.008E7	1.677E8	2.322E8	8.019E7	1951.00	5014.57	6902.68	3227.52	32.66	54.57	63.34	47.46	41	75	85	58	82	196	249	122
P11762	Galectin-1 OS=Rattus norvegicus GN=Lgals1 PE=1 SV=2 - [LEG1_RAT]	14.8	0.000E0	6.073E7	9.853E7	3.772E7		337.98	761.56	397.23		58.52	65.93	52.59		8	8	7		16	22	16
P12007	Isovaleryl-CoA dehydrogenase, mitochondrial OS=Rattus norvegicus GN=Ivd PE=1 SV=2 - [IVD_RAT]	46.4	1.844E6	1.524E7	2.315E7	5.696E6	26.41	206.16	325.33	130.86	5.19	13.21	19.81	10.61	2	5	7	4	2	8	11	6
P13264	Glutaminase kidney isoform, mitochondrial OS=Rattus norvegicus GN=Gls PE=1 SV=2 - [GLSK_RAT]	74.0	0.000E0	0.000E0	6.206E6	0.000E0		25.27	125.71			2.52	12.61			1	5			1	7	
P13676	Acylamino-acid-releasing enzyme OS=Rattus norvegicus GN=Apeh PE=1 SV=1 - [ACPH_RAT]	81.3	0.000E0	2.578E7	3.573E7	1.445E7		366.84	511.19	330.51		21.31	44.54	18.31		10	17	8		15	24	15
P13697	NADP-dependent malic enzyme OS=Rattus norvegicus GN=Me1 PE=1 SV=2 - [MAOX_RAT]	64.0	0.000E0	1.825E7	2.676E7	1.159E7		365.93	573.93	143.19		33.04	37.24	24.13		13	14	8		21	25	11
P13803	Electron transfer flavoprotein subunit alpha, mitochondrial OS=Rattus norvegicus GN=Etfa PE=1 SV=4 - [ETFA_RAT]	34.9	0.000E0	9.225E7	1.319E8	5.879E7		729.80	1019.41	519.76		53.45	64.26	48.05		12	15	11		28	39	26
P14173	Aromatic-L-amino-acid decarboxylase OS=Rattus norvegicus OX=10116 GN=Ddc PE=1 SV=1 - [DDC_RAT]	54.0	0.000E0	0.000E0	3.408E6	0.000E0		20.83	55.74			1.46	5.42			1	2			1	3	
P14604	Enoyl-CoA hydratase, mitochondrial OS=Rattus norvegicus GN=Echs1 PE=1 SV=1 - [ECHM_RAT]	31.5	0.000E0	2.271E7	3.960E7	1.212E7		243.32	469.62	85.86		27.93	31.03	14.83		6	7	3		13	22	4
P15205	Microtubule-associated protein 1B OS=Rattus norvegicus OX=10116 GN=Map1b PE=1 SV=3 - [MAP1B_RAT]	269.5	3.492E5	0.000E0	0.000E0	1.665E7	0.00			22.10	0.33			0.33	1			1	1			1
P17220	Proteasome subunit alpha type-2 OS=Rattus norvegicus GN=Psma2 PE=1 SV=3 - [PSA2_RAT]	25.9	0.000E0	3.692E7	7.202E7	2.349E7		372.16	744.09	408.11		44.87	50.00	49.57		8	11	10		17	26	21
P18395	Cold shock domain-containing protein E1 OS=Rattus norvegicus OX=10116 GN=Csde1 PE=2 SV=1 - [CSDE1_RAT]	88.8	7.809E5	0.000E0	0.000E0	1.367E6	23.15			23.84	0.88			1.00	1			1	1			1
P18420	Proteasome subunit alpha type-1 OS=Rattus norvegicus GN=Psma1 PE=1 SV=2 - [PSA1_RAT]	29.5	0.000E0	7.708E7	1.184E8	4.616E7		583.62	851.41	352.78		57.79	66.92	55.89		12	15	12		23	34	20
P18421	Proteasome subunit beta type-1 OS=Rattus norvegicus GN=Psmb1 PE=1 SV=3 - [PSB1_RAT]	26.5	0.000E0	2.650E7	6.188E7	1.761E7		210.57	473.60	200.40		27.92	39.17	27.92		5	7	5		8	20	8
P18422	Proteasome subunit alpha type-3 OS=Rattus norvegicus GN=Psma3 PE=1 SV=3 - [PSA3_RAT]	28.4	2.605E6	4.279E7	6.420E7	2.796E7	68.11	448.32	659.17	424.55	14.12	38.43	41.57	38.82	3	10	11	9	3	18	25	18
P19218	Pancreatic secretory granule membrane major glycoprotein GP2 OS=Rattus norvegicus OX=10116 GN=Gp2 PE=1 SV=1 -	58.7	0.000E0	4.590E6	8.550E6	0.000E0		25.62	81.92			6.60	10.38			3	4			3	6	
P21139	Alpha-mannosidase 2C1 OS=Rattus norvegicus GN=Man2c1 PE=1 SV=1 - [MA2C1_RAT]	115.9	0.000E0	1.221E6	7.397E6	0.000E0		25.93	37.30			1.44	5.96			1	4			1	4	
P21670	Proteasome subunit alpha type-4 OS=Rattus norvegicus GN=Psma4 PE=1 SV=1 - [PSA4_RAT]	29.5	6.918E6	5.820E7	8.616E7	3.273E7	35.68	572.66	873.96	495.61	3.07	49.43	65.90	49.43	1	9	11	9	1	18	28	15
P22062	Protein-L-isoaspartate(D-aspartate) O-methyltransferase OS=Rattus norvegicus GN=Pcmt1 PE=1 SV=2 - [PIMT_RAT]	24.6	0.000E0	7.619E6	1.362E7	2.503E6		144.42	105.10	41.96		15.86	30.84	7.49		2	3	1		6	6	2
P22791	Hydroxymethylglutaryl-CoA synthase, mitochondrial OS=Rattus norvegicus OX=10116 GN=Hmgcs2 PE=1 SV=1 - [HMCS2_	56.9	0.000E0	3.825E7	6.171E7	2.216E7		377.09	623.76	325.39		27.36	30.31	20.87		13	16	10		20	28	17
P28073	Proteasome subunit beta type-6 OS=Rattus norvegicus GN=Psmb6 PE=1 SV=3 - [PSB6_RAT]	25.3	0.000E0	3.021E7	5.382E7	1.155E7		255.22	249.08	207.78		25.21	37.39	12.18		6	6	3		10	11	6
P28077	Proteasome subunit beta type-9 OS=Rattus norvegicus GN=Psmb9 PE=1 SV=2 - [PSB9_RAT]	23.3	0.000E0	4.683E6	1.433E7	0.000E0		47.95	70.73			17.35	9.59			3	1			4	2	
P30349	Leukotriene A-4 hydrolase OS=Rattus norvegicus GN=Lta4h PE=2 SV=2 - [LKHA4_RAT]	69.1	8.624E6	1.092E8	1.480E8	6.046E7	49.25	1592.86	2046.65	1697.19	1.97	43.28	46.07	46.07	1	21	22	22	2	56	71	68
P34064	Proteasome subunit alpha type-5 OS=Rattus norvegicus GN=Psma5 PE=2 SV=1 - [PSA5_RAT]	26.4	1.893E7	7.759E7	1.060E8	2.966E7	118.02	482.49	565.85	294.40	13.28	46.89	46.89	25.31	2	8	8	4	3	13	20	9
P34067	Proteasome subunit beta type-4 OS=Rattus norvegicus GN=Psmb4 PE=1 SV=2 - [PSB4_RAT]	29.2	0.000E0	3.052E7	5.145E7	1.539E7		368.43	604.35	208.43		28.14	37.26	31.18		5	6	6		17	20	11
P35171	Cytochrome c oxidase subunit 7A2, mitochondrial OS=Rattus norvegicus OX=10116 GN=Cox7a2 PE=1 SV=1 - [CX7A2_RA	9.3	4.320E6	0.000E0	0.000E0	5.481E6	38.43	51.41		67.02	12.05	12.05		12.05	1	1		1	1	1		1
P36876	Serine/threonine-protein phosphatase 2A 55 kDa regulatory subunit B alpha isoform OS=Rattus norvegicus GN=Ppp2r2a P	51.6	0.000E0	4.767E6	1.030E7	0.000E0		38.68	91.38			8.05	8.05			3	3			5	5	
P38650	Cytoplasmic dynein 1 heavy chain 1 OS=Rattus norvegicus GN=Dync1h1 PE=1 SV=1 - [DYHC1_RAT]	531.9	0.000E0	1.508E7	2.282E7	9.473E6		225.31	1117.67	392.64		3.47	12.19	6.01		11	42	22		16	66	33
P40112	Proteasome subunit beta type-3 OS=Rattus norvegicus GN=Psmb3 PE=1 SV=1 - [PSB3_RAT]	22.9	5.799E6	3.321E7	5.739E7	2.003E7	34.13	246.89	426.63	199.57	8.78	27.32	38.54	20.00	1	4	7	3	2	13	17	10
P40329	Arginine--tRNA ligase, cytoplasmic OS=Rattus norvegicus GN=Rars PE=1 SV=2 - [SYRC_RAT]	75.8	0.000E0	3.127E7	4.502E7	2.052E7		516.95	855.52	562.98		35.76	36.06	28.79		19	21	14		26	39	25
P47942	Dihydropyrimidinase-related protein 2 OS=Rattus norvegicus GN=Dpysl2 PE=1 SV=1 - [DPYL2_RAT]	62.2	0.000E0	1.131E8	1.577E8	5.620E7		1485.17	1996.86	1129.71		59.09	56.29	48.78		22	21	19		54	59	41
P52296	Importin subunit beta-1 OS=Rattus norvegicus GN=Kpnb1 PE=1 SV=1 - [IMB1_RAT]	97.1	3.410E6	3.037E7	4.742E7	1.395E7	90.10	494.51	1034.80	486.73	4.57	19.20	29.83	17.49	3	11	18	11	4	20	42	21
P52631	Signal transducer and activator of transcription 3 OS=Rattus norvegicus GN=Stat3 PE=1 SV=1 - [STAT3_RAT]	88.0	0.000E0	8.580E6	1.429E7	0.000E0		56.30	234.10			4.94	12.73			3	7			3	13	
P60901	Proteasome subunit alpha type-6 OS=Rattus norvegicus GN=Psma6 PE=1 SV=1 - [PSA6_RAT]	27.4	1.228E7	6.782E7	1.093E8	4.034E7	63.93	617.28	844.59	581.03	4.88	48.78	48.78	47.97	1	10	11	10	2	19	26	18
P63155	Crooked neck-like protein 1 OS=Rattus norvegicus GN=Crnkl1 PE=2 SV=1 - [CRNL1_RAT]	83.4	0.000E0	3.440E6	2.025E7	0.000E0		57.73	22.04			6.96	3.62			4	2			4	3	
P63312	Thymosin beta-10 OS=Rattus norvegicus OX=10116 GN=Tmsb10 PE=1 SV=2 - [TYB10_RAT]	5.0	3.646E6	0.000E0	0.000E0	2.845E6	65.74			52.05	18.18			18.18	1			1	2			1
P81799	N-acetyl-D-glucosamine kinase OS=Rattus norvegicus GN=Nagk PE=1 SV=4 - [NAGK_RAT]	37.2	6.460E6	1.595E7	2.727E7	9.224E6	213.17	224.46	391.20	193.69	15.74	28.86	32.94	13.70	5	6	8	3	10	8	14	6
P84092	AP-2 complex subunit mu OS=Rattus norvegicus GN=Ap2m1 PE=1 SV=1 - [AP2M1_RAT]	49.6	0.000E0	1.128E7	1.584E7	6.820E6		117.34	216.80	83.95		11.26	15.63	11.26		4	6	4		7	10	5
P97519	Hydroxymethylglutaryl-CoA lyase, mitochondrial OS=Rattus norvegicus GN=Hmgcl PE=2 SV=1 - [HMGCL_RAT]	34.2	2.289E6	1.766E7	2.605E7	7.935E6	30.44	348.01	389.77	208.66	4.31	23.08	25.85	19.08	1	5	6	4	1	11	12	9
Q04631	Protein farnesyltransferase/geranylgeranyltransferase type-1 subunit alpha OS=Rattus norvegicus GN=Fnta PE=1 SV=1 - [	44.0	0.000E0	5.425E6	8.290E6	0.000E0		47.36	89.11			9.02	10.61			3	3			4	6	
Q08849	Syntaxin-3 OS=Rattus norvegicus GN=Stx3 PE=1 SV=1 - [STX3_RAT]	33.2	1.436E7	4.817E6	0.000E0	2.290E6	217.70	54.84		42.56	12.46	7.27		7.61	4	2		2	7	3		2
Q3T1K5	F-actin-capping protein subunit alpha-2 OS=Rattus norvegicus GN=Capza2 PE=1 SV=1 - [CAZA2_RAT]	32.9	5.009E6	6.125E7	9.148E7	3.820E7	33.83	557.18	865.91	728.63	9.79	58.74	65.38	54.20	2	10	11	9	2	16	24	21
Q498M4	WD repeat-containing protein 5 OS=Rattus norvegicus GN=Wdr5 PE=1 SV=1 - [WDR5_RAT]	36.6	0.000E0	1.745E7	2.796E7	4.533E6		89.20	80.00	27.27		9.88	5.69	7.78		3	2	2		4	3	2
Q4KLJ8	Phosducin-like protein 3 OS=Rattus norvegicus GN=Pdcl3 PE=1 SV=1 - [PDCL3_RAT]	27.7	0.000E0	1.539E7	3.509E7	8.360E6		64.56	69.22	50.20		9.58	12.92	11.25		2	3	3		2	5	5
Q4KLZ6	Triokinase/FMN cyclase OS=Rattus norvegicus GN=Tkfc PE=1 SV=1 - [TKFC_RAT]	59.4	6.473E6	2.923E7	4.014E7	6.825E6	147.64	362.91	365.18	92.63	18.86	35.29	25.95	3.46	7	12	9	2	8	21	19	3
Q4KM35	Proteasome subunit beta type-10 OS=Rattus norvegicus GN=Psmb10 PE=2 SV=1 - [PSB10_RAT]	29.0	1.604E6	9.472E6	1.376E7	0.000E0	37.24	173.57	172.75		12.45	20.15	20.15		2	4	4		2	6	7	
Q5BJQ6	Cleavage stimulation factor subunit 1 OS=Rattus norvegicus GN=Cstf1 PE=2 SV=1 - [CSTF1_RAT]	48.4	0.000E0	8.313E6	1.145E7	3.392E6		90.82	118.70	85.17		6.96	11.83	11.83		2	3	3		3	5	4
Q5PPJ4	Deoxyhypusine hydroxylase OS=Rattus norvegicus OX=10116 GN=Dohh PE=2 SV=1 - [DOHH_RAT]	33.1	0.000E0	3.015E6	4.466E6	0.000E0		20.19	44.74			6.62	7.95			2	2			2	2	
Q5RJR8	Leucine-rich repeat-containing protein 59 OS=Rattus norvegicus GN=Lrrc59 PE=1 SV=1 - [LRC59_RAT]	34.8	0.000E0	7.755E6	1.145E7	3.372E6		61.88	134.34	61.95		16.61	19.87	13.36		4	5	4		4	8	5
Q5XI29	Cleavage and polyadenylation specificity factor subunit 7 OS=Rattus norvegicus OX=10116 GN=Cpsf7 PE=1 SV=1 - [CPSF	51.0	0.000E0	3.950E6	6.139E6	0.000E0		43.51	20.99			4.11	1.73			2	1			3	1	
Q5XIM7	Lysine--tRNA ligase OS=Rattus norvegicus OX=10116 GN=Kars PE=1 SV=1 - [SYK_RAT]	71.6	0.000E0	5.776E7	7.940E7	2.345E7		337.80	491.09	400.19		14.70	28.12	26.20		8	13	12		15	23	26
Q62698	Cytoplasmic dynein 1 light intermediate chain 2 OS=Rattus norvegicus GN=Dync1li2 PE=1 SV=1 - [DC1L2_RAT]	54.7	0.000E0	3.916E6	7.927E6	0.000E0		21.68	74.19			4.43	4.43			2	2			2	3	
Q63060	Glycerol kinase OS=Rattus norvegicus OX=10116 GN=Gk PE=2 SV=1 - [GLPK_RAT]	57.4	0.000E0	8.672E6	1.393E7	4.908E6		103.49	145.94	48.32		14.69	17.37	7.44		6	7	3		9	10	4
Q63569	26S protease regulatory subunit 6A OS=Rattus norvegicus GN=Psmc3 PE=2 SV=1 - [PRS6A_RAT]	49.1	0.000E0	1.167E7	4.175E7	6.312E6		191.69	352.09	232.36		15.03	29.61	12.07		5	10	4		8	18	6
Q63644	Rho-associated protein kinase 1 OS=Rattus norvegicus GN=Rock1 PE=1 SV=1 - [ROCK1_RAT]	159.5	0.000E0	0.000E0	1.473E6	0.000E0	23.73	26.40	16.32		0.73	0.73	0.73		1	1	1		1	1	1	
Q64057	Alpha-aminoadipic semialdehyde dehydrogenase OS=Rattus norvegicus GN=Aldh7a1 PE=1 SV=2 - [AL7A1_RAT]	58.7	0.000E0	2.954E7	5.374E7	1.700E7		419.27	823.55	490.14		33.77	45.27	39.52		11	16	14		22	30	25
Q64350	Translation initiation factor eIF-2B subunit epsilon OS=Rattus norvegicus GN=Eif2b5 PE=1 SV=2 - [EI2BE_RAT]	80.2	0.000E0	6.677E6	8.970E6	0.000E0		58.80	36.82			6.28	2.51			3	2			4	2	
Q66H68	RNA-binding protein 47 OS=Rattus norvegicus GN=Rbm47 PE=2 SV=1 - [RBM47_RAT]	64.1	0.000E0	2.165E7	4.031E7	1.129E7		83.39	166.00	149.64		9.32	15.25	13.39		5	7	6		10	13	10
Q66X93	Staphylococcal nuclease domain-containing protein 1 OS=Rattus norvegicus GN=Snd1 PE=2 SV=1 - [SND1_RAT]	101.9	0.000E0	3.146E7	4.519E7	2.020E7		529.81	607.26	432.96		41.36	37.51	33.22		23	23	20		38	40	35
Q68FU3	Electron transfer flavoprotein subunit beta OS=Rattus norvegicus GN=Etfb PE=2 SV=3 - [ETFB_RAT]	27.7	0.000E0	8.569E7	1.185E8	5.323E7		1037.28	1329.90	990.38		49.02	49.02	45.49		11	11	10		27	35	27
Q6AYT3	tRNA-splicing ligase RtcB homolog OS=Rattus norvegicus GN=Rtcb PE=2 SV=1 - [RTCB_RAT]	55.2	0.000E0	2.716E7	3.976E7	1.455E7		398.52	396.51	309.02		28.71	30.30	22.97		11	11	9		17	17	15
Q6BBI8	Ubiquitin-fold modifier-conjugating enzyme 1 OS=Rattus norvegicus GN=Ufc1 PE=2 SV=1 - [UFC1_RAT]	19.5	0.000E0	6.425E6	1.419E7	0.000E0		21.04	20.71			4.79	10.18			1	2			1	2	
Q6P767	Pituitary tumor-transforming gene 1 protein-interacting protein OS=Rattus norvegicus GN=Pttg1ip PE=2 SV=1 - [PTTG_R/	19.9	1.369E7	0.000E0	0.000E0	5.790E6	54.15			35.69	4.60			4.60	1			1	2			1
Q6P799	Serine--tRNA ligase, cytoplasmic OS=Rattus norvegicus GN=Sars PE=1 SV=3 - [SYSC_RAT]	58.6	0.000E0	4.902E7	6.611E7	2.927E7		620.13	748.57	508.23		36.13	37.89	28.52		13	14	12		24	28	24
Q811A3	Procollagen-lysine,2-oxoglutarate 5-dioxygenase 2 OS=Rattus norvegicus GN=Plod2 PE=2 SV=1 - [PLOD2_RAT]	84.5	0.000E0	6.024E6	1.013E7	3.719E6		163.43	186.39	65.75		5.43	12.21	1.63		3	6	1		5	8	2
Q8R4A1	ERO1-like protein alpha OS=Rattus norvegicus GN=Ero1l PE=1 SV=1 - [ERO1A_RAT]	54.0	0.000E0	5.938E6	1.030E7	2.981E6		78.14	130.87	45.21		7.33	15.30	5.39		3	6	3		6	8	4
Q8VHT6	Arsenite methyltransferase OS=Rattus norvegicus OX=10116 GN=As3mt PE=1 SV=1 - [AS3MT_RAT]	41.0	0.000E0	1.382E7	1.852E7	6.738E6		92.57	79.37	41.59		7.32	17.89	7.32		2	5	2		4	5	3
Q9ER34	Aconitate hydratase, mitochondrial OS=Rattus norvegicus GN=Aco2 PE=1 SV=2 - [ACON_RAT]	85.4	1.775E6	1.822E8	2.491E8	1.185E8	37.94	2789.26	3090.38	1845.75	1.03	59.87	61.41	54.74	1	37	38	32	1	102	105	76
Q9ESS6	Basal cell adhesion molecule OS=Rattus norvegicus OX=10116 GN=Bcam PE=2 SV=1 - [BCAM_RAT]	67.5	0.000E0	2.608E6	3.724E6	0.000E0		56.19	30.74			5.29	3.04			2	1			2	1	
Q9JHL4	Drebrin-like protein OS=Rattus norvegicus GN=Dbnl PE=1 SV=1 - [DBNL_RAT]	48.6	0.000E0	7.216E6	9.931E6	2.626E6		115.20	258.54	50.83		8.26	13.30	6.65		3	5	2		3	9	3
Q9JHW0	Proteasome subunit beta type-7 OS=Rattus norvegicus GN=Psmb7 PE=1 SV=1 - [PSB7_RAT]	29.9	0.000E0	8.352E6	1.790E7	3.426E6		53.02	136.91	32.04		11.19	24.55	7.94		3	5	2		4	8	2
Q9JJ50	Hepatocyte growth factor-regulated tyrosine kinase substrate OS=Rattus norvegicus GN=Hgs PE=1 SV=1 - [HGS_RAT]	86.2	0.000E0	4.259E6	6.563E6	0.000E0		41.55	45.58			4.38	2.58			2	2			2	4	
Q9QY17	Protein kinase C and casein kinase substrate in neurons 2 protein OS=Rattus norvegicus GN=Pacsin2 PE=1 SV=2 - [PACN	55.9	4.836E7	0.000E0	0.000E0	5.055E6	33.16			37.06	1.43			2.25	1			1	2			1
Q9QYW3	MOB-like protein phocein OS=Rattus norvegicus GN=Mob4 PE=1 SV=1 - [PHOCN_RAT]	26.0	7.505E5	7.527E6	1.053E7	4.311E6	24.24	43.04	107.54	37.85	4.00	10.22	16.44	16.44	1	2	3	3	1	4	5	4
